# Intermolecular 2+2 imine-olefin photocycloadditions enabled by Cu(I)-alkene MLCT

**DOI:** 10.1038/s41467-022-30393-6

**Published:** 2022-05-19

**Authors:** Daniel M. Flores, Michael L. Neville, Valerie A. Schmidt

**Affiliations:** 1 University of California San Diego, Department of Chemistry and Biochemistry, 9500 Gilman Drive, La Jolla, CA 92093 USA; 2 Present Address: Element Biosciences, 9880 Campus Point Drive #210, San Diego, CA 92121 USA

**Keywords:** Photocatalysis, Organic chemistry, Catalysis

## Abstract

2 + 2 Photocycloadditions are idealized, convergent construction approaches of 4-membered heterocyclic rings, including azetidines. However, methods of direct excitation are limited by the unfavorable photophysical properties of imines and electronically unbiased alkenes. Here, we report copper-catalyzed photocycloadditions of non-conjugated imines and alkenes to produce a variety of substituted azetidines. Design principles allow this base metal-catalyzed method to achieve 2 + 2 imine-olefin photocycloaddition via selective alkene activation through a coordination-MLCT pathway supported by combined experimental and computational mechanistic studies.

## Abstract (web summary)

An ideal method to construct azetidines would be through a [2 + 2] photocycloaddition that joins an olefin and an imine, shown only rarely in the literature, partially due to competitive photochemical processes of the imine. Here, the authors report copper-catalyzed photocycloadditions of imines and alkenes to produce a variety of substituted azetidines, via activation of the olefin.

## Introduction

**Fig. 1:**
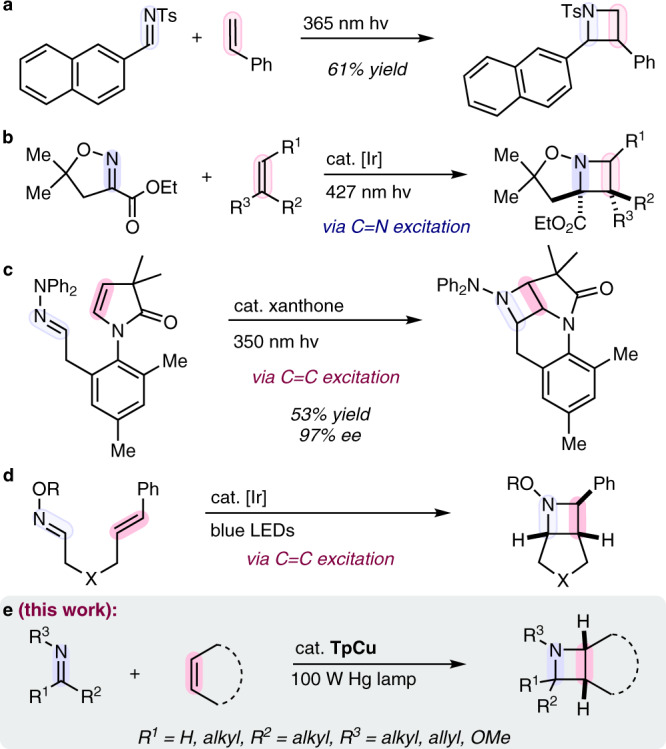
Previous strategies of azetidine forming 2 + 2 photocycloadditions and this approach. **a** Use of substrate π-stacking pre-organization^13^. **b** Activation via cyclic glyoxylate imine sensitization^16^. **c** Xanthone sensitization^17^. **d** Activation via Ir-mediated sensitization^18^. **e** Alkene activation via Cu-coordination and excitation. Tp = hydrotris(pyrazolyl)borate.

Nitrogen heterocycles are prevalent motifs in small molecule pharmaceuticals and their rapid construction continues to drive new reaction development^1^. While 3-, 5- and 6-membered *N*-heterocycles are exceptionally common, made possible by a variety of synthetically accessible routes for their construction, 4-membered heterocycles are scarce by comparison despite the promising biological activity displayed by many azetidine-containing compounds^2–7^. The 2 + 2 photocycloaddition of two π-components is a prototypical approach to 4-membered ring construction. While limited in generality, the Paternò-Büchi reaction forms oxetanes through direct irradiation of a carbonyl C = O double bond followed by C = C double bond capture^8–10^. Aza-Paternò-Büchi^11^ analogs using imines in place of aldehydes or ketones do not generally result in the formation of analogous azetidines due in part to the low barrier *E/Z* isomerization available upon excitation of the imine C = N double bond^12^. Maruoka reported a rare intermolecular azetidine-forming 2 + 2 photocycloaddition example using naphthaldehyde derived *N*-sulfonyl aldimines and styrene or benzofuran (Fig. 1a)^13^. However, this reactivity was exclusive to these substrate pairs, enabled by a π-stacking association of the substrates prior to excitation. Photosensitization of cyclic C = N double bonds which cannot undergo *E/Z* isomerization is one successful strategy for azetidine formation^14–16^. A recent report from Schindler and co-workers used *fac*-Ir(dFppy)_3_ (*fac* = facial; dFppy = 2-(2,4-difluorophenyl)-pyridine) and blue light irradiation as a photocatalyst system to selectively activate the C = N double bond of cyclic glyoxylate imines which coupled to a wide array of alkenes (Fig. 1b)^16^. Preferential excitation of the alkene component resulted in recent advancements in intramolecular azetidine-forming 2 + 2 photocycloadditions. Sivaguru and co-workers developed an enantioselective xanthone-mediated 2 + 2 photocycloaddition of atropisomeric enamides tethered to imines (Fig. 1c)^17^. Schindler and co-workers also developed an intramolecular process using [Ir[dF(CF_3_)ppy]_2_(dtbbpy)](PF_6_) (dF(CF_3_)ppy = 2-(2,4-difluorophenyl)-3-trifluoromethylpyridine; dtbbpy = 4,4′-di-*tert*-butyl-2,2′-bipyridine) as a photosensitizer that selectively activated vinyl arenes, leading to fused azetidine products (Fig. 1d)^18^. Despite these substrate-controlled advances, catalyst-controlled approaches to 2 + 2 imine-olefin photocycloaddition (IOPC) remain limited as general strategies for azetidine construction.

Transition metal compounds capable of photoexcitation via metal to ligand charge transfer (MLCT) to achieve intermolecular electron or energy transfer have gained significant recognition for their use in chemical synthesis^19–22^. While the majority of these reports use photocatalysts that access the needed excited states in the absence of any substrate, the formation of the active photocatalytic species in situ through selective substrate engagement is a comparatively underdeveloped approach. Seminal contributions from Salomon, Kochi, and co-workers identified that cyclobutane formation from alkene photodimerization was significantly improved by the addition of Cu(I)-trifluoromethanesulfonate (CuOTf) due to the in situ formation of a Cu(I)-olefin compound serving as the active chromophore^23^. Meggers and co-workers reported photochemical cyclobutane synthesis by in situ formation of the active chromophore as a chiral at metal, O-bound Rh-enone compound^24^. We recently reported a Cu-catalyzed 2 + 2 carbonyl-olefin photocycloaddition (COPC) approach for oxetane formation by inverting conventional Paternò-Büchi reactivity and achieved alkene activation via Cu(I)-coordination followed by MLCT^25^.

In this work we show that azetidines may be accessed by substrate coordination and selective activation via MLCT with the alkene π-component in a 2 + 2 imine-olefin photocycloaddition (Fig. 1e).

## Results

### Reaction optimization

We irradiated a 1.1:1 mixture of *N*-butyl-2-methylpropan-1-imine (**1**) and norbornene with catalytic hydrotris(pyrazolyl)borate copper(I) (TpCu) using a 100 W Hg lamp for 24 h resulting in formation of the corresponding azetidine **1A** in 55% yield with exclusive *exo* selectivity (Table 1, entry 1). Removing either TpCu or light resulted in no formation of **1A** (entries 2 and 3). Using a threefold excess of **1**, produced **1A** in 79% yield (entry 4). Application of a 300 nm cut-on filter resulted in a significant decrease in the yield of **1A** to 39% even after a prolonged 36 h reaction time suggesting that product forming irradiation occurred at wavelengths shorter than 300 nm (entry 5). Using CuCl or CuOTf in place of TpCu did not result in any detectable quantities of **1A** (entries 6 and 7), underscoring the importance of the coordination environment of the Cu(I) ion source.

**Table 1:** Summary of reaction optimization studies.


Entry	Deviation from standard	Yield of 1A^a^
1	None	55%
2	No TpCu	n.d.
3	No light	n.d.
4	3 equiv **1**	79%
5	Entry 4 + 300 nm cut-on filter	39%
6	CuCl in place of TpCu	n.d.
7	CuOTf in place of TpCu	n.d.

*n.d.* not detected.

^a^**1A** was detected as a >95:5 *exo*:*endo* mixture and 62:48 diastereomer mixture at C2; yields determined by ^1^H NMR analysis of crude reaction mixtures using durene as an internal standard. Tp = hydrotris(pyrazolyl)borate, OTf = trifluoromethanesulfonate.

### Reaction scope

**Fig. 2:**
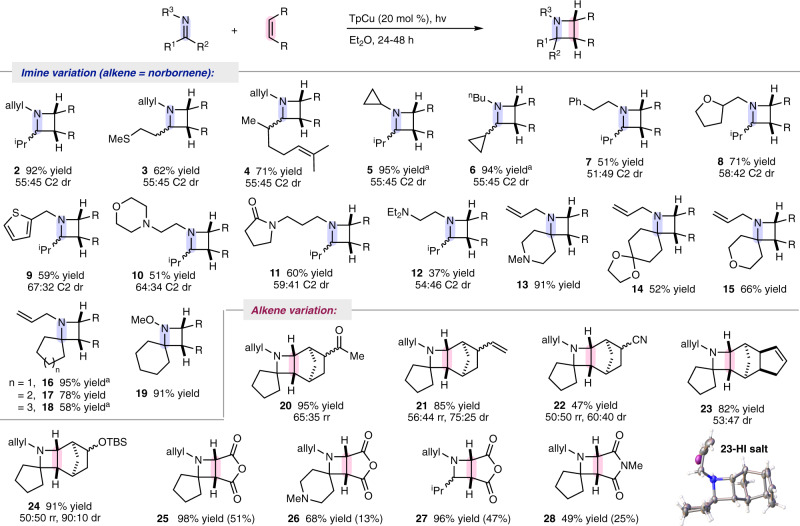
2 + 2 Imine-olefin photocycloaddition reaction scope. Summary of imine and alkene substrate variation. All reactions carried out with 1 equiv alkene, 1.1 equiv imine, 20 mol % TpCu, in diethyl ether (0.15 M) and irradiated with a 100 W Hg lamp in borosilicate tubes; all yields are of isolated material following purification via basic alumina chromatography and diastereomeric ratios were determined via gas chromatography of crude reaction mixtures; all products detected as >95:5 *exo*:*endo* diastereomers. Yield in parentheses obtained in the absence of TpCu. ^*a*^3 equiv of imine used.

In exploration of imines that undergo Cu-catalyzed 2 + 2 IOPC with norbornene, we found that aldimines from *N*-allyl amine condensation with isobutyraldehyde, 3-(methylthio)propanal, or melonal were all successfully converted to their corresponding azetidines, **2**, **3**, and **4**, respectively, in good to excellent yields (Fig. 2). Imines bearing cyclopropyl groups participated in this 2 + 2 IOPC to produce azetidines exclusively with no detection of cyclopropyl ring-opened products (**5** and **6**)^26, 27^. While imines derived from acetophenone, benzaldehyde, or aniline did not participate in this 2 + 2 IOPC, *N*-phenethylamine isobutyraldimine, produced azetidine **7**, suggesting that arenes are tolerated when not conjugated to the imine C = N double bond. Heterocycles including tetrahydrofuran (**8**), thiophene (**9**), and morpholine (**10**) as well as an acyclic 3° amine (**12**) were all well incorporated in this process. Productive 2 + 2 IOPC was particularly notable in the presence of a γ-lactam (**11**), showcasing the chemoselectivity of olefin activation even in the presence of other potentially competitive chromophores. Imines derived from ketones also reacted efficiently to produce the corresponding azetidines even when the imine also contained Lewis-basic 3° amine (**13**), acetal (**14**), or ether (**15**) functionalities.

Biologically active azetidines such as penaresidin A, nicotianamine, medicanine, and mugineic acid bear *N*-alkyl or no substitution, but access to 2° azetidines can be challenging using existing cyclization methods that require *N*-substituents that are stubborn or not amenable to cleavage. This Cu-catalyzed 2 + 2 IOPC is uniquely capable of engaging *N*-alkyl imines directly as well as N-substituents that are easily removed. *N*-Allyl azetidine **13** underwent deallylation to reveal the corresponding 2° azetidine in 82% yield using 2 mol % Pd(PPh_3_)_4_ and 2-mercaptobenzoic acid. Alternatively, *O*-methyl oxime-derived azetidine **19** underwent facile reductive N-O bond cleavage using activated Zn dust in aqueous acetic acid to provide the corresponding unsubstituted azetidine in 93% yield.

Diversification of the olefin component allowed norbornene derivatives containing acetyl, vinyl, cyano, and *tert*-butyltrimethyl silyl ether groups to be successfully coupled with *N*-allyl cyclopentanimine (Fig. 2, **20**–**24**). The identity of azetidine **23** was unambiguously confirmed through determination of the solid-state structure of its hydroiodic salt using X-ray diffraction (**23**-HI salt).

While maleic anhydride is known to undergo selective cyclobutane forming 2 + 2 photocycloaddition with ethylene using acetophenone as a photosensitizer^28^, direct excitation of maleic anhydride with *N*-allyl cyclopentanimine resulted in the formation of azetidine **25** in 51% yield along with a complex mixture of unidentified byproducts. Subjecting the same reactant pair to irradiation in the presence of 20 mol % TpCu resulted in the exclusive formation of **25** in 98% yield. Similarly, other imines were converted to the corresponding maleic anhydride derived azetidines (**26** and **27**) and use of TpCu uniformly increased both reaction efficiency and selectivity compared to uncatalyzed reactivity. *N*-Methyl maleimide was also successfully converted to the corresponding azetidine **28** with *N*-allyl cyclopentanimine in 49% yield.

### Mechanism investigations

**Fig. 3:**
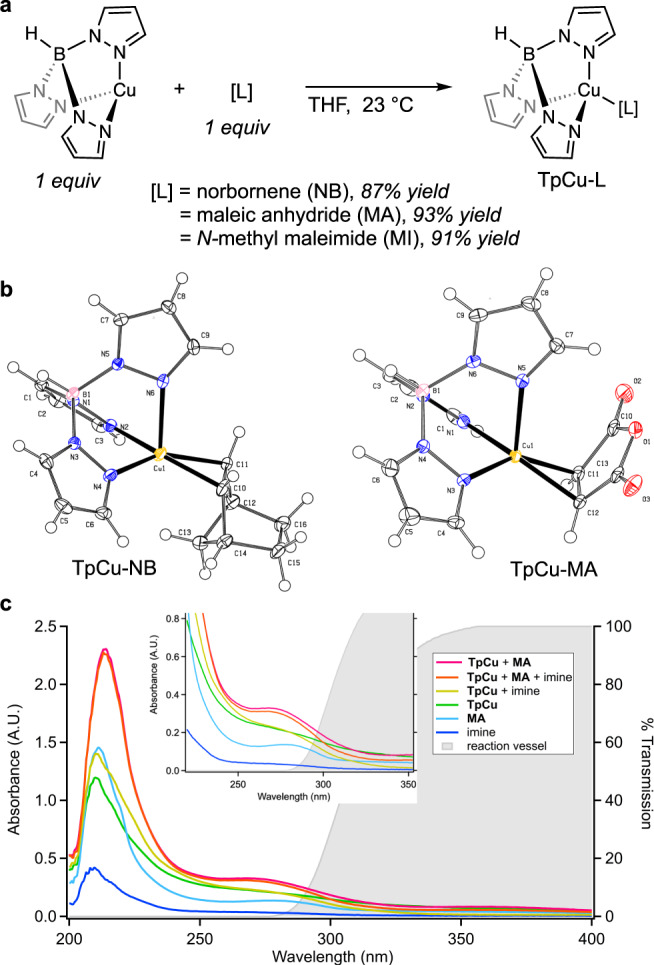
Spectroscopic investigations of 2 + 2 IOPC reaction intermediates. **a** Synthesis of hydrotris(pyrazolyl)borate copper olefin compounds. **b** X-ray structures of the norbornene and maleic anhydride complexes TpCu-NB and TpCu-MA. **c** Electronic absorption spectra of reaction components (TpCu, MA = maleic anhydride, imine = **1**) and transmission spectrum of reaction vessels used. Pink line = stoichiometric mixture of TpCu and maleic anhydride; orange line = stoichiometric mixture of TpCu, maleic anhydride, and **1**; gold line = stoichiometric mixture of TpCu and **1**; green line = TpCu; light blue line = maleic anhydride; dark blue line = 1. Gray shaded area indicates transmission spectrum of the borosilicate glass reaction vessels used. A.U. = arbitrary units.

We initially hypothesized that 2 + 2 IOPC could occur through activation of either the imine or alkene π-bond. Through a combination of ^1^H NMR and electronic absorption spectroscopies we observed the requirement of alkene coordination to TpCu for successful azetidine formation. Three olefin adducts of TpCu were successfully prepared and isolated through equimolar mixing experiments (Fig. 3a). TpCu-NB was isolated as a white crystalline solid and the solid-state structure determined by single crystal X-ray diffraction (Fig. 3b). The coordination geometry is best described as pseudo-tetrahedral about the Cu-ion with the carbon-carbon bond distance of the coordinated olefin (1.387 Å) in the range of other Cu(I)-norbornene compounds (1.36-1.39 Å) indicative of a predominating σ-bonding, rather than π-back-bonding, metal-olefin coordination interaction^29–32^. Examination of the Cu-N(pyrazolyl) bond distances show that while two were nearly equivalent (Cu-N(2) = 1.999(2), Cu-N(4) = 2.010(2)), the third was significantly longer (Cu-N(6) = 2.274(2)), breaking the C_3_ symmetry axis of the hydrotris(pyrazolyl)borate chelate.

The solid-state structure of TpCu-MA shows an analogous coordination geometry to TpCu-NB and while the carbon-carbon bond length of the coordinated maleic anhydride (1.390 Å) is elongated compared to unligated maleic anhydride (1.3032 Å)^33^, it is significantly shortened compared to the only other structurally characterized Cu(I)-maleic anhydride compound supported by a strongly donating iminophosphanamide chelate (1.49 Å)^34^. This suggests that despite the electronic disparities between maleic anhydride and norbornene, the bonding in TpCu-MA is analogous to that observed in TpCu-NB. While ligand exchange was observed by ^1^H NMR spectroscopy from TpCu-NB in the presence of the imines used in our study, isolation of a TpCu-imine coordination compound was unsuccessful likely due to the observed rapid, reversible ligand exchange. However, TpCu-MA did not undergo ligand exchange when subjected to imines of this study. We observed that irradiating equimolar amounts of TpCu-MA, *N*-allyl cyclopentanimine, and norbornene for 24 h resulted in the exclusive formation of azetidine **25** in 95% yield. This suggests that olefin coordination is necessary for 2 + 2 IOPC to proceed and that the coordinated alkene is selectively incorporated into the cycloadduct produced. We noted throughout our investigations that when additional strained, cyclic alkenes were assayed, azetidine formation was not observed when TpCu-alkene coordination was not detected by ^1^H NMR.

The transmission spectrum of the borosilicate glass reaction vessels used in this study showed that light below 280 nm is effectively filtered from reaching the reaction mixture (Fig. 3c, gray area) and the electronic absorption spectra of ethereal solutions of most individual 2 + 2 IOPC reaction components showed no distinct absorption features 250–400 nm (Fig. 3c). While neither TpCu (green line), **1** (dark blue line), nor norbornene (not pictured) showed significant absorptions above 250 nm, maleic anhydride did (light blue line), explaining the small amount of non-selective background photocycloaddition observed with this alkene. Conversely, a stoichiometric solution mixture of TpCu and maleic anhydride (pink line) resulted in an electronic absorption spectrum with a distinctive broad feature with a maximum at 263 nm, which is similar to the spectrum observed of TpCu-NB which shows a maximum at 272 nm^25^, and is nearly identical to the spectrum of a stoichiometric mixture of TpCu, maleic anhydride, and imine **1** (orange line). In combination with the diminished azetidine formation when a 300 nm cut-on filter was applied (Table 1, entry 6), these data support azetidine formation occurring via excitation between 280–300 nm.

**Fig. 4:**
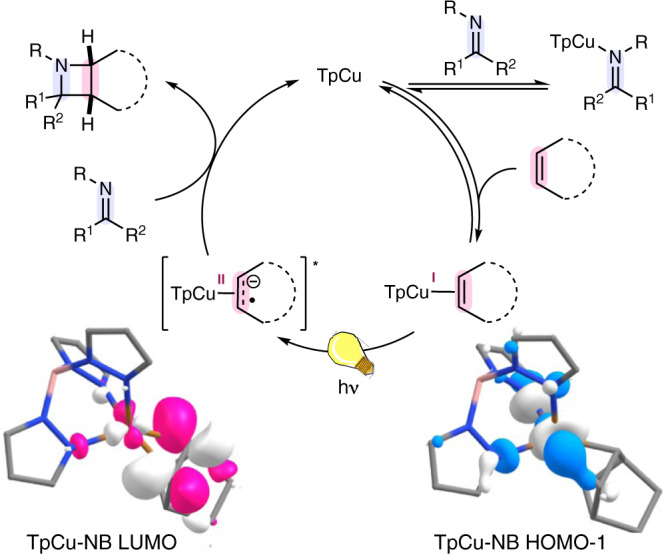
Mechanistic proposal of the title reaction. The proposed 2 + 2 IOPC mechanism commences with olefin substrate coordination to TpCu. Ligand exchange experiments indicate that imine substrates undergo rapid and reversible coordination to TpCu. The active TpCu-olefin chromophore then undergoes a MLCT from computationally visualized TpCu-NB HOMO-1 which is primarily Cu-based to TpCu-NB LUMO which is olefin(π*)-based. Capture by the imine C = N double bond leads to azetidine formation and release of TpCu.

A calculated electronic absorption spectrum of TpCu-NB best approximated the experimentally observed spectrum using time-dependent density functional theory (DFT) calculations of at the M06 level of theory with the Tamm-Dancoff approximation. This enabled the construction of qualitative molecular orbital diagrams for TpCu-NB at the observed absorption feature 250–300 nm linked to azetidine formation (see the Supporting Information for additional details). The highest occupied molecular orbitals (HOMO-1) and the lowest unoccupied molecular orbital (LUMO) of TpCu-NB are primarily TpCu-based and olefin(π*)-based, respectively (Fig. 4). This is supported by previous DFT calculations which concluded an MLCT of idealized cationic Cu(I)-ethylene compounds was responsible for cyclobutane forming 2 + 2 photocycloadditions^35^ and recent optical and X-ray transient absorption spectroscopic insights of Cu(I)-mediated photodimerization of norbornene^36^. The selective imine C = N capture of this excited state affords the cycloadduct azetidine. Chemoselectivity of azetidine formation over alkene dimerization pathways is attributed to the rapid rates of C-centered radical addition to C = N double bonds^37^ and preference over alkene addition^26^.

Analogous to our observations in 2 + 2 COPC, luminescence data collected from excitation between 280–300 nm did not indicate linear relationships between TpCu and the concentration of added norbornene or imine **1**. However, a quenching relationship was observed with maleic anhydride, indicating its potential as a sensitizing agent in this process. While photosensitization cannot be ruled out for all substrates, the impact of olefin coordination ability in azetidine formation and chemoselectivity suggests that a shared sensitization process in all examples is unlikely to be operative. Stereochemical probes often used to infer 2 + 2 photocycloaddition spin states are not applicable in this 2 + 2 IOPC system as identical stereoisomers would be produced irrespective of the proceeding spin state due to the geometric constraints of the cyclic alkenes used.

## Discussion

In conclusion, we report herein an intermolecular 2 + 2 photocycloaddition of aliphatic imines and alkenes to form azetidines. The use of hydrotris(pyrazolyl)borate copper(I) as a pre-catalyst allows for coordination of the olefin component and selective absorption of light 280–300 nm. A combination of solution ^1^H NMR and electronic absorption spectroscopies along with DFT calculations from solid-state structural data supports a Cu(*d*) to olefin(π*) MLCT pathway to azetidine formation. This work highlights a catalyst controlled 2 + 2 imine-olefin photocycloaddition strategy that selectively activates π-components at red-shifted wavelengths compared to uncoordinated alkenes to access novel azetidine containing compounds.

## Methods

### Representative procedure

In a nitrogen filled glovebox a borosilicate culture tube was charged with imine (1.1 equiv), TpCu (0.2 equiv), alkene (1.0 equiv), and diethyl ether (0.15 M), capped then sealed with electrical tape and irradiated with a UVP Blak-Ray B-100A UV lamp in a fume hood. After the indicated time, the reaction mixture was quenched by exposure to air for 15 min, then concentrated under reduced pressure. The residue was taken up in methanol and purified by passage through a portion of basic alumina before solvent removal under reduced pressure to afford pure azetidine compounds.

## Supplementary information

Supplentary Information

## Data Availability

Experimental data as well as ^1^H and ^13^C NMR spectra for all new compounds prepared in the course of these studies are provided in the Supplementary Information of this manuscript. The X-ray crystallographic coordinates for compounds **23**-HI, TpCu-NB, TpCu-MA have been deposited at the Cambridge Crystallographic Data Centre with the accession codes 1986944 [10.5517/ccdc.csd.cc24pkzd], 1986945 [10.5517/ccdc.csd.cc24pl0g], 1986946 [10.5517/ccdc.csd.cc24pl1h], respectively. These data can be obtained free of charge from The Cambridge Crystallographic Data Center via www.ddcd.cam.ac.uk/data_request/cif. All other data including synthetic procedures, spectroscopic, crystallographic, and computational data are available in the Supplementary Information Files.
