# Supplementary material for: Intermolecular 2+2 imine-olefin photocycloadditions enabled by Cu(I)-alkene MLCT
Source: Nat Commun. 2022 May 19;13:2764. doi: 10.1038/s41467-022-30393-6 (PMC9120151; doi:10.1038/s41467-022-30393-6)
Supplement: Supplementary file 1 — Supplentary Information [file 41467_2022_30393_MOESM1_ESM.pdf]

Intermolecular 2+2 Imine-Olefin Photocycloadditions Enabled by Cu(I)-Alkene MLCT

Daniel M. Flores, Michael L. Neville, and Valerie A. Schmidt
Department of Chemistry and Biochemistry
University of California San Diego, La Jolla, CA 92093, United States

Supplementary Information

I. Supplementary Methods

A. General Considerations. All air- and moisture-sensitive manipulations were carried out using standard high vacuum line, Schlenk or cannula techniques or in an M. Braun inert atmosphere drybox containing an atmosphere of purified nitrogen. Solvents for air- and moisture-sensitive manipulations were dried and deoxygenated using literature procedures.[1]

^{1}H and ^{13}C NMR were recorded on Bruker 300 MHz or Varian 500 MHz spectrometers at 300 and 126 MHz, respectively. All chemical shifts are reported relative to $SiMe_4$ using ^{1}H (residual) chemical shifts of the solvent as a secondary standard. GC analyses were performed using an Agilent Technologies 7890B gas chromatograph equipped with an Agilent 7693 autosampler and Agilent HP-5 capillary column (30 m x 0.320mm x 250μm). Standard method parameters: 1.2 mL/min flow rate with oven program 80 – 250 °C with a ramp rate of 25 °C/min and hold time of 8.7 minutes at 250 °C. High-resolution mass spectra were measured using a Thermo LCQdeca APCI-MS.

Photophysical Methods. Electronic absorption and fluorescence experiments were conducted using sealable 1-cm path length fused quartz cuvettes (Starna Cells, catalog number 3-Q-10-GL14-C, 3.5 mL volume) using a Shimadzu UV-2450 UV-Vis spectrometer and a HORIBA Scientific Fluoromax-4 fluorometer. Samples were prepared in a dry nitrogen glove box. Filter experiments were conducted using a long pass UV filter with a cut-on wavelength of 300 nm (Asahi Spectra XUL0300).

Photochemical Reactions. Photochemical reactions were carried out using two 100-W Blak-Ray Long Wave Ultraviolet Lamps (Hg) in a fume hood. The light source was placed approximately 20 cm from the sample and the reaction mixture was stirred vigorously using a magnetic stir bar. All reactions were performed in VWR 13 x 100 mm borosilicate culture tubes that were capped and sealed with electrical tape.

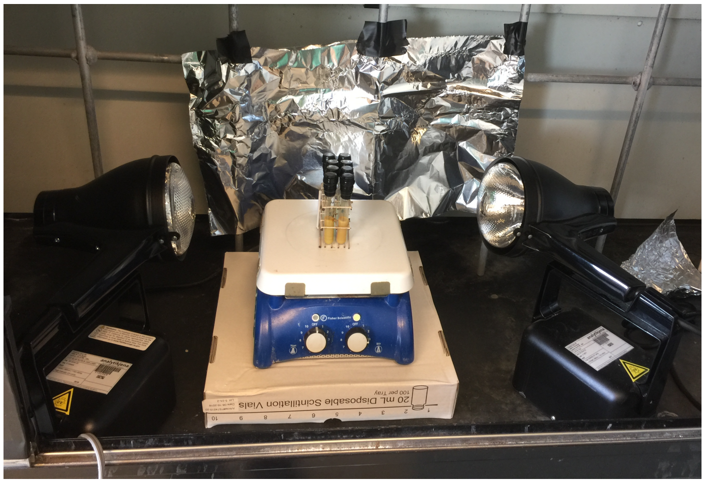

Supplementary Figure 1. Photoreactor set-up for 2+2 IOPC reactions

B. Preparation of TpCu

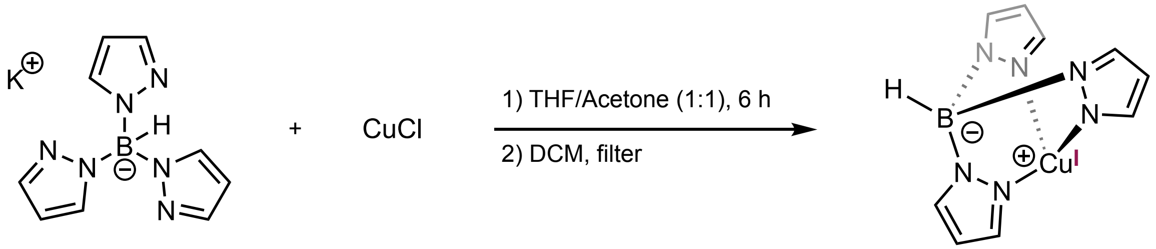

In a nitrogen filled glovebox, a 20 mL vial was charged with CuCl (0.125g, 1 equiv), potassium trispyrazolylborate (0.318 g, 1 equiv) and a magnetic stir bar. A mixture of THF (4 mL) and dried, degassed acetone (4 mL) was added and the mixture was stirred vigorously for 6 h. The solvent was removed under reduced pressure, the resultant solid extracted with dichloromethane (5 mL), and the suspension filtered through a thin pad of celite on a fritted funnel. Removal of the solvent under reduced pressure provided TpCu (0.349 g, 98% yield) as a faint blue solid. Analytical data for TpCu:

^{1}H NMR (500 MHz, C_6D_6): δ 7.58 (d, J=1.8 Hz, 3H), 7.09 (d, J=1.2 Hz 3H), 5.96 (t, J=1.8 Hz, 3H); ^{13}C NMR (126 MHz; C_6D_6): δ 141.8, 135.9, 104.9; HRMS (ESI-TOFMS): [M]+ calcd. for $[C_9H_{10}BCuN_6]^+$ = 276.0352, found = 276.0354.

C. Preparation of Substrates

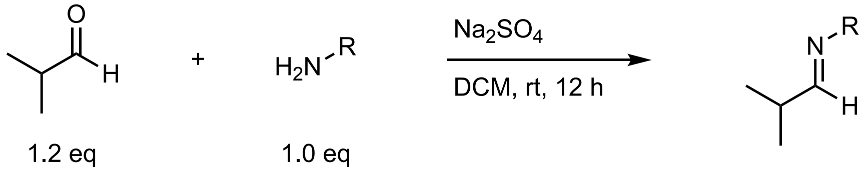

General Procedure A: To a round bottom flask charged with a magnetic stir bar was added isobutryaldehyde (1.2 equiv), sodium sulfate (2.0 equiv), DCM (0.50 M) and placed under a nitrogen atmosphere. A solution of amine (1.0 equiv) in DCM (10 mL) was added via syringe over the course of 5 min and the reaction mixture was allowed to stir for 12 h. The reaction was then filtered, and solvent was removed under reduced pressure to provide the desired imine as an oil. Imines were then freeze-pump-thawed, and vacuum transferred before being brought into a dry nitrogen filled glovebox and stored in the freezer.

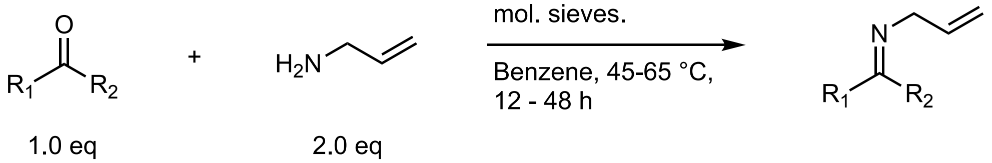

General Procedure B: To a round bottom flask charged with a magnetic stir bar was added carbonyl (1.0 equiv), *N*-allyl amine (2.0 equiv), crushed molecular sieves (1.0 g), benzene (0.50 M) and placed under a nitrogen atmosphere. The reaction mixture was heated to 45-65 °C and allowed to stir vigorously for at least 12 h. Upon consumption of the carbonyl as monitored by ^{1}H NMR the reaction was then filtered through a medium frit funnel, and solvent was removed under reduced pressure to provide the desired imine as an oil. Imines were then freeze-pump-thawed, and vacuum transferred before being brought into a dry nitrogen filled glovebox and stored in the freezer.

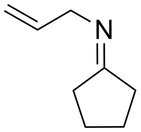

***N*-Allylcyclopentylideneamine** was synthesized by general procedure B. Physical and spectral data was in accordance with literature data.[2]

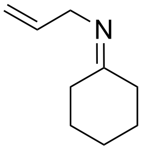

***N*-Allylcyclohexylideneamine** was synthesized by general procedure B. Physical and spectral data was in accordance with literature data.[2]

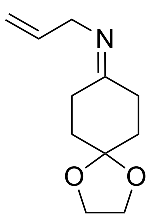

***N*-Allylcyclohexylideneamine-4-monoethylene acetal** was synthesized by general procedure B. Physical and spectral data was in accordance with literature data.[3]

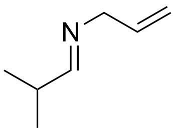

***N*-allyl-2-methylpropan-1-imine** was synthesized by general procedure A. Physical and spectral data was in accordance with literature data.[4]

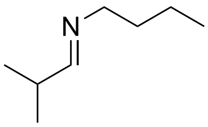

***N*-isobutylidenebutylamine** was synthesized by general procedure A. Physical and spectral data was in accordance with literature data.[5]

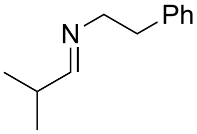

***N*-(2-methylpropylidene)-2-phenethylamine** was synthesized by general procedure A. Physical and spectral data was in accordance with literature data.[6]

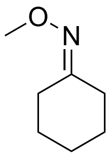

Cyclohexanone O-methyl oxime was synthesized by general procedure A. Physical and spectral data was in accordance with literature data.[7]

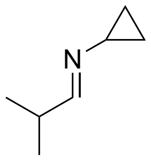

***N*-isobutylidenecyclopropylamine** was synthesized by general procedure A. Physical and spectral data was in accordance with literature data.[8]

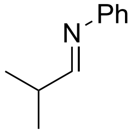

***N*-(2-methylpropylidene)aniline** was synthesized by general procedure A. Physical and spectral data was in accordance with literature data.[9]

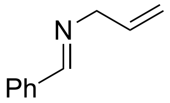

Benzylidene allylamine was synthesized by general procedure A. Physical and spectral data was in accordance with literature data.[10]

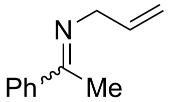

N-(1-phenylethylidene)prop-2-en-1-amine was synthesized by general procedure B. Physical and spectral data was in accordance with literature data.[11]

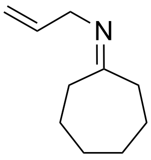

***N*-Allylcycloheptylideneamine** was synthesized by general procedure B in 87% yield (2.36 g) as a colorless oil. Analytical data:

^{1}H NMR (300 MHz; C_6D_6): δ 6.16 (m, 1H), 5.35 (dq, *J* = 17.2, 2.0 Hz, 1H), 5.11 (dq, *J* = 10.3, 2.0 Hz, 1H), 3.78 (d, *J* = 5.3 Hz, 2H), 2.49-2.45 (m, 2H), 2.17 (d, *J* = 9.0 Hz, 2H), 1.92 (d, *J* = 6.0 Hz, 2H), 1.46 (m, 2H), 1.37-1.23 (m, 11H); ^{13}C NMR (126 MHz; C_6D_6): δ 174.5, 137.4, 113.9, 52.8, 43.3, 40.9, 31.4, 30.1, 26.9, 24.8, 24.0; HRMS (ESI-TOFMS) Calcd. for $[C_{10}H_{17}N+H^+]^+$ = 152.1434, Found = 152.1434

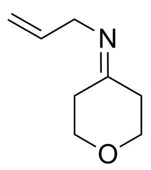

4-*N*-allyl-tetrahydro-4H-pyran was synthesized by general procedure B in 92% (2.57 g) yield as a colorless oil. Analytical data:

^{1}H NMR (300 MHz; C_6D_6): δ 6.60-5.939 (m, 1H), 5.244-5.023 (m, 2H), 3.82-3.79 (m, 2H), 3.54 (t, J=6 Hz, 2H), 3.36 (t, J=6 Hz, 2H), 2.28 (t, J=6 Hz, 2H), 1.87 (t, J=6 Hz, 2H); ^{13}C NMR (126 MHz; C_6D_6): δ 167.2, 136.8, 114.1, 68.6, 67.0, 42.5, 39.9, 30.1; HRMS (ESI-TOFMS) Calc. for $[C_8H_{13}NO+H^+]^+$ = 140.1070, Found = 140.1070

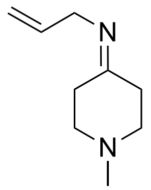

***N*-allyl-1-methyl-4-piperidinylidene** was synthesized by general procedure B in 89% (2.41 g) yield as a colorless oil. Analytical data:

^{1}H NMR (300 MHz; C_6D_6): δ 5.91-5.78 (m, 1H), 5.03-4.94 (m, 2H), 3.86 (d, J = 5.6 Hz, 2H), 2.58 (t, J = 5.9 Hz, 1H), 2.46 (t, J = 6.1 Hz, 2H), 2.40-2.27 (m, 9H), 2.20 (s, 4H), 2.17-2.14 (m, 1H); ^{13}C NMR (126 MHz; C_6D_6): δ 168.9, 136.9, 114.0, 56.4, 55.3, 55.0, 45.5, 40.9, 28.3; HRMS (ESI-TOFMS) Calcd. for $[C_9H_{16}N_2+H]^+$ = 153.1386, Found = 153.1388

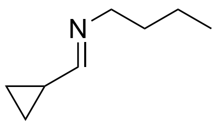

***N*-cyclopropylidenebutylamine** was synthesized by general procedure A in 93% yield (3.35 g) as a colorless oil. Analytical data:

^{1}H NMR (300 MHz; $CDCl_3$): δ 6.87 (d, J = 6.8 Hz, 1H), 3.27 (td, J = 6.8, 1.3 Hz, 2H), 1.61-1.47 (m, 3H), 1.39-1.26 (m, 2H), 0.86 (t, J = 7.3 Hz, 3H), 0.51-0.45 (m, 4H); ^{13}C NMR (126 MHz; $CDCl_3$): δ 164.7, 60.8, 33.3, 20.4, 15.7, 13.7, 5.7; HRMS (ESI-TOFMS) Calcd. for $[C_8H_{15}N+H^+]^+$ = 126.1282, Found = 126.1284

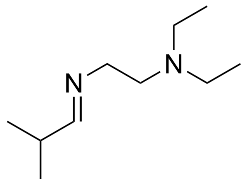

***N'*-isobutylidene-*N*,*N*-diethyl-1,2-ethanediamine** was synthesized by general procedure A in 92% yield (3.57 g) as a colorless oil. Analytical data:

^{1}H NMR (300 MHz; $CDCl_3$): δ 7.36 (dt, *J* = 4.1, 1.3 Hz, 1H), 3.47-3.42 (m, 2H), 2.67 (t, *J* = 6.8 Hz, 2H), 2.44 (q, *J* = 7.1 Hz, 4H), 2.34-2.23 (m, 1H), 1.00-0.92 (m, 12H); ^{13}C NMR (126 MHz; $CDCl_3$): δ 168.3, 60.0, 53.8, 47.4, 33.7, 18.9, 12.2; HRMS (ESI-TOFMS) Calcd. for $[C_{10}H_{22}N_2+H^+]^+$ = 171.1856, Found = 171.1856

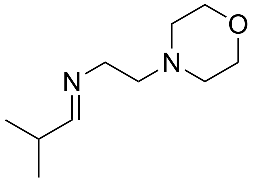

***N*-isobutylidene-1,2-ethanemorpholine** was synthesized by general procedure A in 95% yield (2.7 g) as a colorless oil. Analytical data:

^{1}H NMR (300 MHz; $CDCl_3$): δ 7.30 (dt, *J* = 4.2, 1.3 Hz, 1H), 3.58-3.55 (m, 4H), 3.42-3.37 (m, 2H), 2.46 (t, *J* = 6.8 Hz, 2H), 2.26-2.22 (m, 5H), 0.97 (d, *J* = 6.9 Hz, 6H); ^{13}C-NMR (126 MHz; $CDCl_3$): δ 168.9, 66.7, 59.3, 58.7, 54.0, 33.7, 18.9; HRMS (ESI-TOFMS) Calcd. for $[C_{10}H_{20}N_2O+H^+]^+$ = 185.1648, Found = 185.1649

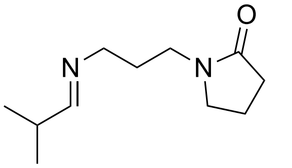

***N*-isobutylidene-1,3-propanepyrrolidone** was synthesized by general procedure A in 93% yield (2.57 g) as a colorless oil. Analytical data:

^{1}H NMR (300 MHz; $CDCl_3$): δ 7.34 (d, *J* = 2.9 Hz, 1H), 3.20 (q, *J* = 7.2 Hz, 4H), 2.67 (t, *J* = 7.0 Hz, 2H), 2.33-2.22 (m, 1H), 1.98 (t, *J* = 8.1 Hz, 2H), 1.65 (quintet, *J* = 6.9 Hz, 2H), 1.28 (dt, *J* = 15.2, 7.5 Hz, 3H), 0.98 (d, *J* = 6.9 Hz, 6H); ^{13}C NMR (126 MHz; $CDCl_3$): δ 173.2, 168.8, 58.4, 46.0, 40.0, 33.7, 30.5, 28.5, 19.0, 17.6; HRMS (ESI-TOFMS) Calcd. for $[C_{11}H_{20}N_2O+H^+]^+$ = 197.1648, Found = 197.1649

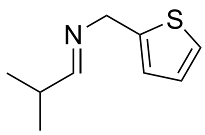

***N*-isobutylidene-methylenethiophene** was synthesized by general procedure A in 88% yield (2.97 g) as a colorless oil. Analytical data:

^{1}H NMR (300 MHz; $CDCl_3$): δ 7.23 (m, 1H), 6.85-6.73 (m, 3H), 4.49 (s, 2H), 2.21 (m, 1H), 0.90 (d, *J* = 6.8 Hz, 6H); ^{13}C NMR (126 MHz; $CDCl_3$): δ 170.0, 143.4, 126.8, 124.53, 124.43, 59.5, 34.1, 19.1; HRMS (ESI-TOFMS) Calcd. for $[C_9H_{13}NS+H]^+$ = 168.0841, Found = 168.0839

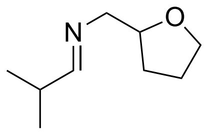

***N*-isobutylidene-methylenefuran** was synthesized by general procedure A in 81% yield (2.50 g) as a colorless oil. Analytical data:

^{1}H NMR (300 MHz; $CDCl_3$): δ 7.38 (dt, *J* = 4.1, 1.3 Hz, 1H), 4.13-4.05 (m, 1H), 3.74-3.67 (m, 1H), 3.59-3.39 (m, 3H), 2.27 (m, 1H), 1.73-1.65 (m, 1H), 1.60-1.50 (m, 3H), 0.96 (dd, *J* = 6.9, 2.0 Hz, 6H); ^{13}C NMR (126 MHz; $CDCl_3$): δ 169.6, 78.2, 67.7, 65.6, 33.8, 29.2, 25.7, 18.95, 18.92; HRMS (ESI-TOFMS) Calcd. for $[C_9H_{17}NO+H]^+$ = 156.1388, Found = 156.1387

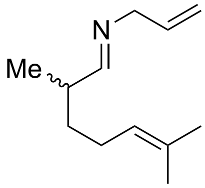

***N*-isobutylidene-melonal** was synthesized by general procedure A in 89% yield (2.29 g) as a colorless oil. Analytical data:

^{1}H NMR (300 MHz; $CDCl_3$): δ 7.27 (d, *J* = 4.8 Hz, 1H), 6.00-5.93 (m, 1H), 5.19-5.12 (m, 2H), 5.01 (dt, *J* = 10.3, 1.6 Hz, 1H), 3.87 (d, *J* = 5.3 Hz, 2H), 2.27 (dt, *J* = 12.6, 6.4 Hz, 1H), 2.00 (m, 2H), 1.64 (s, 3H), 1.52 (s, 3H), 1.28 (m, 1H), 0.97 (d, *J* = 6.9 Hz, 3H); ^{13}C NMR (126 MHz; $CDCl_3$): δ 168.8, 136.7, 131.1, 124.5, 114.6, 63.3, 38.7, 34.0, 25.64, 25.51, 17.4, 17.0; HRMS (ESI-TOFMS) Calcd. for $[C_{12}H_{21}N+H]^+$ = 180.1752, Found = 180.1753

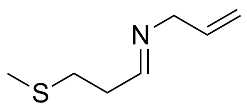

***N*-isobutylidene-methional** was synthesized by general procedure A in 91% yield (2.52 g) as a colorless oil. Analytical data:

^{1}H NMR (300 MHz; $CDCl_3$): δ 7.25 (d, *J* = 1.1 Hz, 1H), 5.94-5.86 (m, 1H), 5.12 (m, 1H), 4.98 (m, 1H), 3.81 (d, *J* = 5.4 Hz, 2H), 2.44 (t, *J* = 7.3 Hz, 2H), 2.24 (m, 2H), 1.73 (d, *J* = 1.8 Hz, 3H); ^{13}C NMR (126 MHz; $CDCl_3$): δ 162.7, 136.3, 114.8, 63.2, 35.2, 29.9, 14.8; HRMS (ESI-TOFMS) Calcd. for $[C_7H_{13}NS+H]^+$ = 144.0846, Found = 144.0845

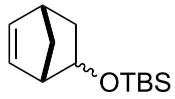

***exo*-(bicyclo[2.2.1]hept-5-en-2-yl) *tert*-butyldimethylsilyl ether** was synthesized via the following procedure. To a dry 100 mL round bottom flask was added a mixture of endo and *exo*-5-norbornen-2-ol (1.0 g, 9.1 mmol), imidazole (1.3 equiv, 0.816 g, 12.1 mmol), and DMF (15 mL). Then *tert*-butyldimethylsiyl chloride (1.0 equiv, 1.4 g, 9.1 mmol) was added in one portion and the reaction kept under nitrogen atmosphere. The reaction was allowed to stir 16 hours before quenching with water (45 mL) and extraction with DCM/Hexane (9:1) three times (25 mL). Combined organic fractions were washed with brine (3 x 25 mL), dried over Na_2SO_4, filtered, and concentrated under reduced pressure. The crude material was purified via flash chromatography using hexanes (R_f = 0.6) as eluent to provide the desired product as a colorless oil (1.4 g, 70% yield). Physical and spectral data was in accordance with literature data.[12]

D. General IOPC Procedures.

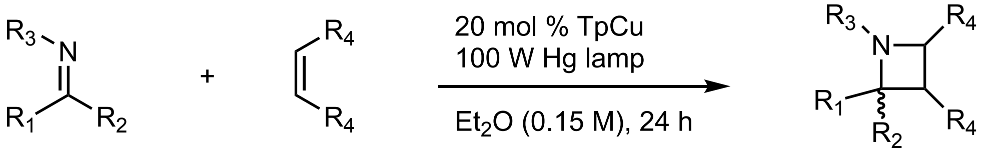

In a glovebox to a borosilicate culture tube charged with a magnetic stir bar was added imine (1.1 equiv), TpCu (0.2 equiv), alkene (30 mg, 1.0 equiv), and diethyl ether (0.15 M). The vial was capped, sealed with electrical tape, and irradiated with a UVP Blak-Ray B-100A UV lamp in a fume hood. After 24 h, or otherwise indicated, the reaction mixture was opened to air for 15 min, then concentrated under reduced pressure. The residue was taken up in methanol and purified by a short plug of basic alumina. If the paramagnetic byproduct Tp_2Cu still remains (indicated by a dark blue coloring), the azetidine product can be extracted with methanol.

E. Characterization of IOPC Products.

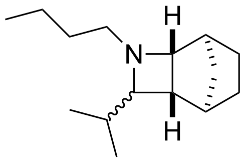

1A was synthesized via general IOPC procedure (24 h reaction time) in 79% yield (55 mg, 55:45 diastereomeric mixture at C2 as determined by GC-MS, >95:5 *exo:endo* as determined by analogy to **16**) as a colorless oil. Analytical data for **1A**:

^{1}H NMR (300 MHz; C_6D_6): δ 3.44 (d, *J* = 4.4 Hz, 1H), 2.83 (d, *J* = 11.2 Hz, 1H), 2.71 (d, *J* = 5.4 Hz, 1H), 2.58-2.46 (m, 3H), 2.34 (m, 3H), 2.06 (s, 1H), 1.95 (m, 4H), 1.78 (d, *J* = 9.6 Hz, 1H), 1.69 (m, 1H), 1.36 (m, 12H), 1.13 (m, 2H), 0.90 (m, 18H), 0.72 (d, *J* = 6.3 Hz, 2H); ^{13}C NMR (126 MHz; C_6D_6): δ 73.9, 73.5, 69.0, 64.5, 59.0, 48.3, 42.6, 39.9, 38.6, 37.9, 37.0, 34.89, 34.85, 34.6, 34.0, 31.9, 30.6, 29.3, 28.7, 27.7, 25.5, 24.1, 20.66, 20.52, 19.2, 19.0, 18.4, 17.3, 14.11, 14.09; HRMS (ESI-TOFMS) Calcd. for $[C_{15}H_{27}N+H]^+$ = 222.2216, Found = 222.2216; R_f (1% MeOH in DCM): 0.2

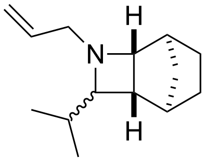

2 was synthesized via general IOPC procedure (24 h reaction time) in 92% yield (60 mg, 55:45 diastereomeric mixture at C2 as determined by GC-MS, >95:5 *exo:endo*, as determined by analogy to **16**) as a colorless oil. Analytical data for **2**:

^{1}H NMR (300 MHz; C_6D_6): δ 5.83 (m, 1H), 5.21 (dd, *J* = 28.5, 13.7 Hz, 1H), 5.00 (t, *J* = 7.0 Hz, 1H), 3.45 (d, *J* = 3.3 Hz, 1H), 3.37 (dd, *J* = 11.6, 4.5 Hz, 1H), 3.24 (d, *J* = 10.8 Hz, 1H), 2.94-2.85 (m, 1H), 2.76 (d, *J* = 4.7 Hz, 1H), 2.53 (t, *J* = 7.2 Hz, 1H), 2.46 (s, 1H), 2.26 (s, 1H), 2.02 (s, 1H), 1.93 (m, 1H), 1.85 (m, 1H), 1.68 (m, 1H), 1.37-1.22 (m, 3H), 1.09 (m, 2H), 0.82 (dd, *J* = 16.4, 3.8 Hz, 6H), 0.66 (d, *J* = 4.3 Hz, 1H); ^{13}C NMR (126 MHz; C_6D_6): δ 137.9, 136.9, 116.1, 114.9, 74.03, 73.96, 69.1, 65.2, 62.6, 52.0, 43.3, 40.0, 39.0, 38.2, 37.4, 35.27, 35.11, 34.96, 34.3, 29.6, 29.0, 27.9, 25.7, 24.4, 19.45, 19.30, 18.7, 17.7; HRMS (ESI-TOFMS) Calcd. for $[C_{14}H_{23}N+H]^+$ = 206.1903, Found = 206.1905; R_f (1% MeOH in DCM): 0.2

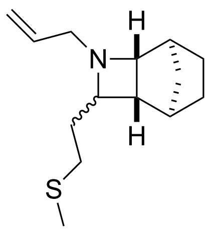

3 was synthesized via general IOPC procedure (24 h reaction time) in 62% yield (47 mg, 51:49 diastereomeric mixture at C2 as determined by GC-MS, >95:5 *exo:endo* as determined by analogy to **16**) as colorless oil. Analytical data for **3**:

^{1}H NMR (300 MHz; C_6D_6): δ 5.86-5.74 (m, 2H), 5.17 (m, 2H), 4.97 (dd, *J* = 9.0, 5.7 Hz, 2H), 3.38 (d, *J* = 5.1 Hz, 2H), 3.27 (dd, *J* = 14.5, 4.9 Hz, 2H), 3.11 (td, *J* = 16.4, 6.2 Hz, 2H), 2.96 (d, *J* = 13.5 Hz, 2H), 2.79 (d, *J* = 6.0 Hz, 1H), 2.45 (d, *J* = 8.9 Hz, 1H), 2.36-2.19 (m, 6H), 1.98 (d, *J* = 12.4 Hz, 2H), 1.88 (s, 2H), 1.80-1.70 (m, 12H), 1.36-1.25 (m, 6H), 1.10 (dd, *J* = 12.7, 8.2 Hz, 2H), 0.79 (m, 4H); ^{13}C NMR (126 MHz; C_6D_6): δ 137.4, 136.5, 115.9, 114.9, 69.3, 66.10, 66.06, 65.8, 61.2, 51.5, 44.5, 39.2, 39.0, 37.6, 37.4, 36.1, 34.57, 34.49, 33.8, 31.0, 30.0, 29.8, 29.1, 27.5, 25.0, 23.9, 15.12, 15.07; HRMS (ESI-TOFMS) Calcd. for $[C_{14}H_{23}NS +H]^+$ = 238.1629, Found = 238.1630; R_f (1% MeOH in DCM): 0.25

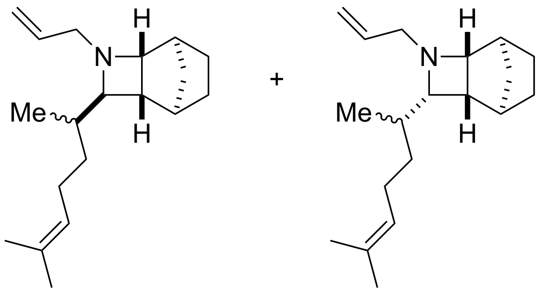

4 was synthesized via general IOPC procedure (24 h reaction time) in 73% yield (63 mg, 61:39 diastereomeric mixture at C2 as determined by GC-MS, >95:5 *exo:endo* as determined by analogy to **16**) as colorless oil. This is a non-separable mixture of diastereomers, and the following spectral lists are a mix of both. Analytical data for **4**:

^{1}H NMR (300 MHz; C_6D_6): δ 5.83 (ddt, *J* = 21.7, 10.7, 5.3 Hz, 2H), 5.27-5.15 (m, 4H), 5.00 (q, *J* = 9.6 Hz, 2H), 3.46 (d, *J* = 4.8 Hz, 1H), 3.38 (dt, *J* = 14.4, 7.4 Hz, 1H), 3.27 (t, *J* = 14.0 Hz, 1H), 2.95 (t, *J* = 12.8 Hz, 1H), 2.89 (d, *J* = 8.7 Hz, 1H), 2.78 (d, *J* = 6.1 Hz, 1H), 2.66 (t, *J* = 8.5 Hz, 1H), 2.58 (t, *J* = 3.3 Hz, 1H), 2.48 (d, *J* = 8.8 Hz, 1H), 2.27 (s, 2H), 2.12 (d, *J* = 10.3 Hz, 2H), 2.00 (dt, *J* = 23.7, 11.2 Hz, 4H), 1.90 (dd, *J* = 24.2, 4.7 Hz, 4H), 1.70 (d, *J* = 9.2 Hz, 2H), 1.66 (d, *J* = 9.8 Hz, 6H), 1.56 (d, *J* = 6.7 Hz, 6H), 1.33-1.27 (m, 4H), 1.12 (dd, *J* = 21.7, 7.9 Hz, 2H), 1.06-1.01 (m, 2H), 0.90-0.80 (m, 8H), 0.70 (d, *J* = 6.4 Hz, 2H); ^{13}C NMR (126 MHz; C_6D_6): δ 137.5, 136.50, 136.38, 130.70, 130.57, 130.46, 125.32, 125.30, 125.15, 115.71, 115.64, 114.54, 114.46, 72.72, 72.54, 72.41, 72.34, 68.87, 68.72, 65.08, 65.00, 62.36, 62.31, 51.60, 51.56, 43.3, 42.6, 39.80, 39.60, 39.09, 38.95, 38.7, 37.92, 37.87, 37.13, 37.06, 34.91, 34.76, 34.62, 34.60, 34.00, 33.95, 33.75, 33.4, 33.16, 33.01, 32.7, 32.2, 29.21, 29.17, 27.60, 27.57, 25.89, 25.73, 25.58, 25.53, 25.51, 25.45, 25.36, 25.33, 24.1, 17.46, 17.43, 17.38, 15.4, 14.1; HRMS (ESI-TOFMS) Calcd. for $[C_{19}H_{31}N + H]^{+}$ =274. 2534 Found = 274. 2532; R_f (1% MeOH in DCM): 0.25

Supplementary Figure 2. Gas chromatograph of diastereomers of **4**

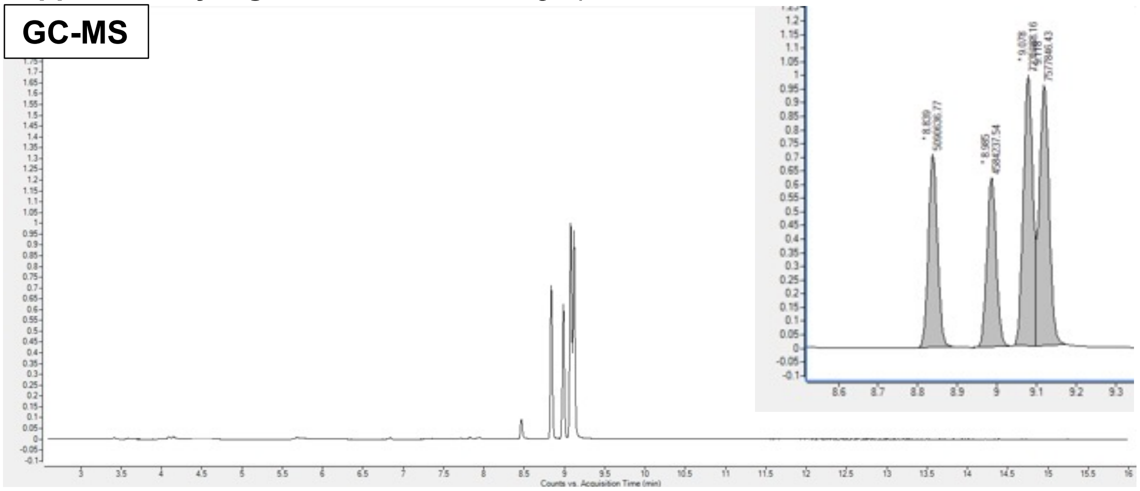

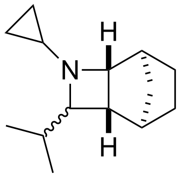

5 was synthesized via general IOPC procedure in 95% yield (62 mg, 55:45 diastereomeric mixture at C2 as determined by GC-MS, >95:5 *exo:endo* as determined by analogy to **16**) as a colorless oil. Analytical data for **5**:

^{1}H NMR (300 MHz; C_6D_6): δ 3.34 (d, *J* = 4.2 Hz, 1H), 3.04 (d, *J* = 5.1 Hz, 1H), 2.78-2.71 (m, 2H), 2.42 (s, 1H), 2.29 (d, *J* = 7.4 Hz, 1H), 2.01 (m, 2H), 1.96 (t, *J* = 5.8 Hz, 1H), 1.88 (m, 5H), 1.78 (m, 1H), 1.66 (m, 1H), 1.29 (m, 6H), 1.16 (d, *J* = 8.1 Hz, 2H), 1.06 (d, *J* = 7.6 Hz, 1H), 0.94-0.80 (m, 14H), 0.72 (d, *J* = 5.2 Hz, 2H), 0.40-0.25 (m, 7H); ^{13}C NMR (126 MHz; C_6D_6): δ 75.6, 74.3, 68.4, 65.8, 42.0, 39.6, 39.08, 38.89, 38.3, 38.1, 34.6, 34.4, 34.1, 33.5, 29.5, 29.3, 28.3, 27.7, 25.3, 24.0, 20.4, 19.09, 18.99, 17.5, 8.64, 8.47, 4.7, 4.0; HRMS (ESI-TOFMS) Calcd. for $[C_{14}H_{24}N + H]^+$ = 206.1904, Found = 206.1903; R_f (1% MeOH in DCM): 0.2

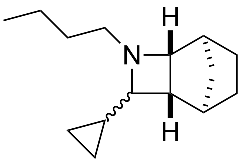

6 was synthesized via general IOPC procedure (24 h reaction time) in 94% yield (65 mg, 55:45 diastereomeric mixture at C2 as determined by GC-MS, >95:5 *exo:endo* as determined by analogy to **16**) as a colorless oil. Analytical data for **6**:

^{1}H NMR (300 MHz; C_6D_6): δ 3.29 (d, *J* = 5.4 Hz, 1H), 2.75 (d, *J* = 5.5 Hz, 2H), 2.70-2.59 (m, 1H), 2.48-2.41 (m, 3H), 2.23 (m, 2H), 2.06-1.94 (m, 4H), 1.37-1.31 (m, 10H), 1.24-1.12 (m, 3H), 0.93-0.86 (m, 10H), 0.42-0.40 (m, 2H), 0.28-0.24 (m, 3H), 0.06-0.05 (m, 2H), -0.13 (m, 1H); ^{13}C NMR (126 MHz; C_6D_6): δ 73.7, 70.3, 68.8, 66.6, 57.5, 48.4, 44.6, 39.96, 39.77, 38.4, 37.3, 35.0, 34.6, 33.6, 31.7, 30.9, 29.3, 27.8, 24.8, 24.0, 20.58, 20.57, 15.0, 14.08, 14.01, 10.7, 4.2, 3.2, 1.2, 0.7; HRMS (ESI-TOFMS) Calcd. for $[C_{15}H_{25}N + H]^+$ = 220.2060, Found = 220.2060; R_f (1% MeOH in DCM): 0.2

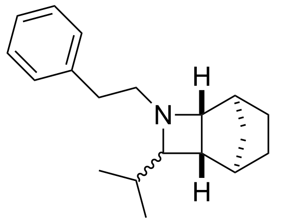

7 was synthesized via general IOPC procedure (72 h reaction time) in 51% yield (44 mg, 51:49 diastereomeric mixture at C2 as determined by GC-MS, >95:5 *exo:endo* as determined by analogy to **16**) as colorless oil. Analytical data for **7**:

^{1}H NMR (300 MHz; C_6D_6): δ 7.19-7.18 (m, 1H), 7.12-7.02 (m, 4H), 3.51 (t, *J* = 6.8 Hz, 1H), 3.43 (d, *J* = 5.5 Hz, 1H), 3.09-3.01 (m, 1H), 2.85 (t, *J* = 7.1 Hz, 1H), 2.78-2.75 (m, 1H), 2.67-2.56 (m, 4H), 2.53-2.44 (m, 3H), 2.25-2.24 (m, 1H), 2.05-2.04 (m, 1H), 2.01-1.97 (m, 1H), 1.96 (s, 1H), 1.88 (d, *J* = 9.1 Hz, 2H), 1.71 (m, 1H), 1.64 (m, 1H), 1.36-1.27 (m, 3H), 1.17-1.07 (m, 2H), 0.92 (d, *J* = 6.9 Hz, 3H), 0.81 (q, *J* = 7.0 Hz, 9H), 0.68 (d, *J* = 6.6 Hz, 2H); ^{13}C NMR (126 MHz; C_6D_6): δ 141.32, 141.12, 128.84, 128.79, 125.86, 125.75, 73.74, 73.51, 69.27, 64.46, 61.24, 50.85, 42.65, 39.87, 38.59, 37. 85, 36.92, 36.50, 35.24, 34.86, 34.83, 34.59, 33.91, 29.25, 28.63, 27.60, 25.41, 24.07, 18.91, 18.89, 18.28, 17.24; HRMS (ESI-TOFMS) Calcd. for $[C_{14}H_{24}N +H]^+$ = 270.2216, Found = 270.2217; R_f (1% MeOH in DCM): 0.2.

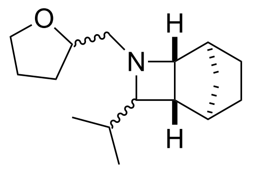

8 was synthesized via general IOPC procedure (24 h reaction time) in 73% yield (36 mg, 55:45 diastereomeric mixture at C2 as determined by GC-MS, >95:5 *exo:endo* as determined by analogy to **16**) as colorless oil. Analytical data for **8**:

^{1}H NMR (300 MHz; C_6D_6): δ 3.85 (d, J = 5.4 Hz, 1H), 3.75 (dt, J = 10.9, 5.7 Hz, 2H), 3.58-3.49 (m, 2H), 3.12 (dd, J = 12.5, 5.6 Hz,), 2.98 (ddd, J = 17.9, 12.7, 5.5 Hz, 1H), 2.73 (dd, J = 12.6, 4.4 Hz, 1H), 2.66-2.60 (m, 1H), 2.53-2.43 (m, 2H), 2.22 (dd, J = 12.5, 5.9 Hz,), 2.12 (s, 1H), 2.04 (d, J = 7.5 Hz,), 1.97 (td, J = 12.5, 5.6 Hz, 1H), 1.90 (s, 1H), 1.85 (s, 1H), 1.75-1.67 (m, 3H), 1.60-1.55 (m, 2H), 1.48 (dd, J = 13.7, 6.5 Hz, 2H), 1.38-1.27 (m, 3H), 1.16-1.08 (m, 1H), 0.86 (qd, J = 14.2, 6.7 Hz, 9H), 0.68 (dd, J = 6.3, 3.5 Hz, 1H); ^{13}C NMR (126 MHz; C_6D_6): δ 79.3, 79.0, 78.8, 78.1, 74.4, 74.0, 73.7, 71.6, 70.1, 67.61, 67.60, 67.50, 66.3, 65.6, 63.7, 62.5, 53.4, 52.5, 43.1, 42.5, 39.63, 39.55, 39.24, 39.06, 38.04, 37.99, 36.87, 36.85, 34.77, 34.75, 34.74, 34.72, 34.69, 34.2, 33.6, 33.3, 30.3, 29.8, 29.33, 29.24, 29.17, 29.0, 28.74, 28.68, 27.61, 27.54, 25.90, 25.82, 25.39, 25.26, 25.11, 24.05, 24.04, 19.11, 18.96, 18.90, 18.86, 18.28, 18.16, 17.25, 17.05; HRMS (ESI-TOFMS) Calc. for $[C_9H_{17}NO+H]^+$ = 156.1388, Found = 156.1387; R_f (1% MeOH in DCM): 0.2.

Supplementary Figure 3. Gas chromatograph of diastereomers of **8**

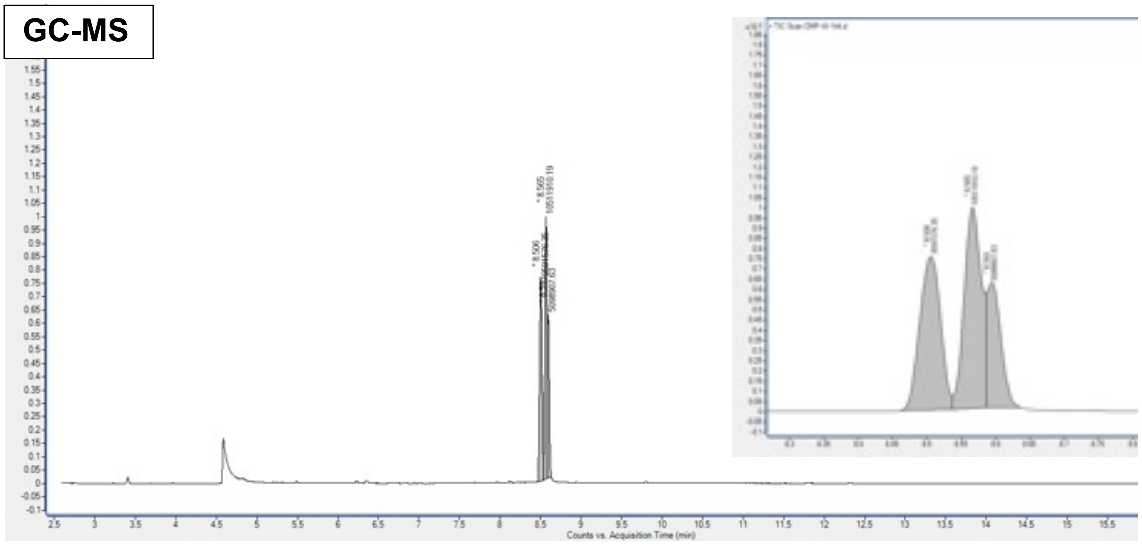

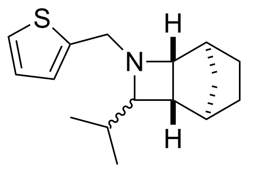

9 was synthesized via general IOPC procedure (48 h reaction time) in 59% yield (49 mg, 51:49 diastereomeric mixture at C2 as determined by GC-MS, >95:5 *exo:endo* as determined by analogy to **16**) as colorless oil. Analytical data for **9**:

^{1}H NMR (300 MHz; C_6D_6): δ 6.89-6.73 (m, 10H), 4.11 (d, *J* = 11.7 Hz, 2H), 3.77 (d, *J* = 11.1 Hz, 1H), 3.57 (d, *J* = 11.1 Hz, 1H), 3.48 (m, 3H), 2.80 (d, *J* = 5.0 Hz, 1H), 2.59 (t, *J* = 7.5 Hz, 1H), 2.53-2.47 (m, 3H), 2.22-2.18 (m, 2H), 1.99 (m, 3H), 1.90 (t, *J* = 5.9 Hz, 1H), 1.85 (s, 2H), 1.82 (s, 1H), 1.66 (m, 3H), 1.24 (m, 2H), 1.19 (m, 4H), 1.08 (m, 4H), 0.91 (dd, *J* = 5.5, 1.2 Hz, 5H), 0.85 (dd, *J* = 5.3, 1.3 Hz, 5H), 0.81-0.79 (m, 10H), 0.67 (m, 5H), 0.57 (s, 1H); ^{13}C NMR (126 MHz; C_6D_6): δ 146.43, 144.40, 126.64, 126.51, 124.67, 124.36, 124.20, 123.33, 73.89, 73.75, 69.79, 64.77, 59.60, 57.92, 48.14, 43.04, 39.85, 39.05, 38.19, 37.13, 35.06, 35.03, 34.98, 34.17, 29.59, 28.94, 27.80, 25.60, 24.31, 19.26, 19.22, 19.13, 18.45, 17.65; HRMS (ESI-TOFMS) Calcd. for $[C_{16}H_{23}NS + H]^+$ = 262.1624, Found = 262.1623; R_f (1% MeOH in DCM): 0.2.

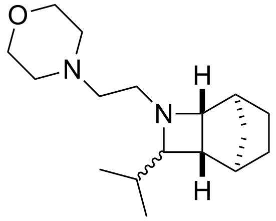

10 was synthesized via general IOPC procedure (24 h reaction time) in 51% yield (45 mg, 64:36 diastereomeric mixture at C2 as determined by GC-MS, >95:5 *exo:endo* as determined by analogy to **16**) as a colorless oil. Analytical data for **10**:

^{1}H NMR (300 MHz; C_6D_6): δ 3.57 (s, 8H), 3.39 (s, 1H), 2.92 (s, 1H), 2.71 (m, 1H), 2.54 (s, 1H), 2.47-2.38 (m, 4H), 2.24 (m, 11H), 2.01 (d, *J* = 11.6 Hz, 1H), 1.86 (m, 3H), 1.72 (s, 1H), 1.64 (s, 1H), 1.30 (d, *J* = 8.1 Hz, 3H), 1.12 (m, 2H), 0.94 (s, 1H), 0.83 (m, 8H), 0.67 (s, 2H); ^{13}C NMR (126 MHz; C_6D_6): δ 73.9, 73.7, 69.72, 66.88, 64.66, 58.54, 57.66, 56.90, 54.33, 46.61, 4282, 39.96, 38.84, 37.88, 36.96, 34.89, 34.79, 34.65, 33.89, 29.22, 28.66, 27.58, 25.33, 24.02, 19.12, 18.90, 18.31, 17.23; HRMS (ESI-TOFMS) Calcd. for $[C_{17}H_{30}N_2O + H]^+$ = 279.2431, Found = 279.2431; R_f (3% MeOH in DCM): 0.2.

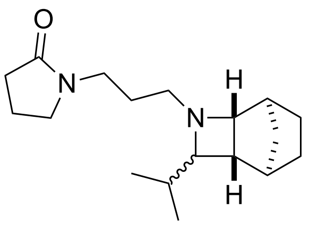

11 was synthesized via general IOPC procedure (48 h reaction time) in 60% yield (55 mg, 59:41 diastereomeric mixture at C2 as determined by GC-MS, >95:5 *exo:endo* as determined by analogy to **16**) as a colorless oil. Analytical data for **11**:

^{1}H NMR (300 MHz; C_6D_6): δ 3.41 (d, J = 5.4 Hz, 1H), 3.24 (m, 3H), 2.72 (m, 6H), 2.52-2.42 (m, 3H), 2.31 (m, 1H), 2.25-2.16 (m, 2H), 2.02 (m, 5H), 1.90 (m, 3H), 1.66 (m, 2H), 1.45 (m, 4H), 1.28 (m, 8H), 1.12 (dd, J = 17.7, 10.0 Hz, 2H), 0.83 (m, 12H), 0.69 (d, J = 6.5 Hz, 2H); ^{13}C NMR (126 MHz; C_6D_6): δ 173.23, 173.16, 73.8, 73.4, 68.9, 64.4, 56.5, 46.15, 46.05, 42.5, 40.6, 39.8, 38.5, 37.8, 36.9, 34.80, 34.61, 34.58, 34.0, 30.54, 30.52, 29.2, 28.6, 27.5, 27.3, 26.1, 25.4, 24.1, 19.1, 18.9, 18.4, 17.6, 17.2; HRMS (ESI-TOFMS) Calcd. for $[C_{18}H_{30}N_2O +H]^+$ = 291.2431, Found = 291.2430; R_f (3% MeOH in DCM): 0.1.

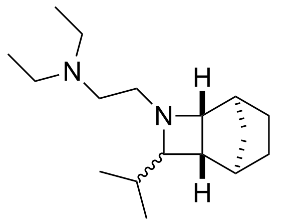

12 was synthesized via general IOPC procedure (24 h reaction time) in 37% yield (31 mg, 54:46 diastereomeric mixture at C2 as determined by GC-MS, >95:5 *exo:endo* as determined by analogy to **16**) as a colorless oil. Analytical data for **12**:

^{1}H NMR (300 MHz; C_6D_6): δ 3.46 (d, J = 5.3 Hz, 1H), 3.02 (m, 1H), 2.75 (m, 1H), 2.59-2.39 (m, 15H), 2.35 (d, J = 3.0 Hz, 1H), 2.03 (s, 1H), 1.96 (t, J = 7.0 Hz, 1H), 1.90 (d, J = 4.3 Hz, 1H), 1.86 (s, 1H), 1.77 (d, J = 10.2 Hz, 1H), 1.69 (m, 1H), 1.30 (m, 4H), 1.12 (t, J = 9.0 Hz, 2H), 0.98 (m, 10H), 0.90 (d, J = 6.5 Hz, 3H), 0.86 (d, J = 6.5 Hz, 2H), 0.82 (d, J = 6.5 Hz, 3H), 0.68 (d, J = 6.5 Hz, 2H); ^{13}C NMR (126 MHz; C_6D_6): δ 73.88, 73.84, 69.9, 64.7, 58.2, 53.2, 52.2, 47.87, 47.71, 47.62, 42.9, 40.0, 38.9, 37.9, 37.0, 35.0, 34.83, 34.63, 33.9, 29.2, 28.7, 27.6, 25.4, 19.05, 18.91, 18.3, 17.3, 12.37, 12.26; HRMS (ESI-TOFMS) Calcd. for $[C_{17}H_{32}N_2 +H]^+$ = 265.2638, Found = 265.2637; R_f (3% MeOH in DCM): 0.2.

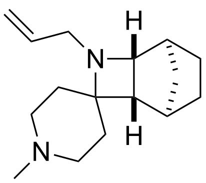

13 was synthesized via general IOPC procedure (24 h reaction time, 3 equiv. of imine) in 91% yield (71 mg, >95:5 *exo:endo* as determined by analogy to **16**) as a colorless oil. Analytical data for **13**:

^{1}H NMR (300 MHz; C_6D_6): δ 5.77 (m, 1H), 5.13 (d, *J* = 17.0 Hz, 1H), 4.93 (d, *J* = 9.9 Hz, 1H), 3.19 (dd, *J* = 13.1, 4.8 Hz, 1H), 2.88 (m, 2H), 2.54 (m, 3H), 2.07 (s, 3H), 2.02 (d, *J* = 21.6 Hz, 2H), 1.89 (d, *J* = 12.3 Hz, 1H), 1.82 (m, 2H), 1.70 (m, 1H), 1.55 (m, 1H), 1.49 (d, *J* = 6.2 Hz, 1H), 1.45 (d, *J* = 8.6 Hz, 1H), 1.32 (m, 3H), 1.12 (d, *J* = 9.2 Hz, 1H), 0.87-0.75 (m, 2H); ^{13}C NMR (126 MHz; C_6D_6): δ 137.8, 115.2, 67.5, 63.9, 54.0, 52.1, 51.8, 46.1, 45.6, 38.7, 34.6, 34.4, 32.3, 31.8, 29.5, 23.9; HRMS (ESI-TOFMS) Calcd. for [$C_{16}H_{26}N_2$ +H]$^+$ = 247.2169, Found = 247.2171; R_f (3% MeOH in DCM): 0.2.

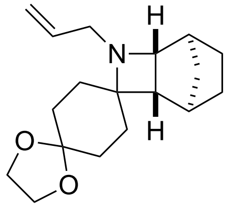

14 was synthesized via general IOPC procedure (72 h reaction time) in 52% yield (48 mg, >95:5 *exo:endo* as determined by analogy to **16**) as a colorless oil. Analytical data for **14**:

^{1}H NMR (300 MHz; C_6D_6): δ 5.99 (m, 1H), 5.21-4.99 (m, 2H), 3.85 (d, *J* = 6.5 Hz, 1H), 3.56 (m, 1H), 3.49 (s, 3H), 3.43 (s, 1H), 2.58 (t, *J* = 8.9 Hz, 2H), 2.29 (t, *J* = 9.3 Hz, 1H), 2.15 (dd, *J* = 20.0, 10.7 Hz, 2H), 2.06 (s, 1H), 1.76 (t, *J* = 8.9 Hz, 2H), 1.68-1.60 (m, 3H); ^{13}C NMR (126 MHz; C_6D_6): δ 137.7, 115.1, 108.2, 106.9, 67.5, 64.0, 63.8, 51.8, 44.9, 38.7, 35.1, 34.5, 33.7, 32.8, 31.1, 29.5, 29.4, 29.0, 23.8; HRMS (ESI-TOFMS) Calcd. for [$C_{18}H_{27}NO_2$ + H]$^+$ = 290.2115, Found = 290.2113; R_f (1% MeOH in DCM): 0.2.

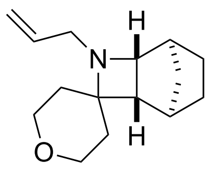

15 was synthesized via general IOPC procedure (48 h reaction time) in 66% yield (49 mg, >95:5 *exo:endo* as determined by analogy to **16**) as a colorless oil. Analytical data for **15**:

^{1}H NMR (300 MHz; C_6D_6): δ 5.76 (m, 1H), 5.13 (d, *J* = 18.0, 1H), 4.95 (d, *J* = 12.0, 1H), 3.73 (m, 2H), 3.16 (m, 3H), 2.84 (m, 2H), 2.45 (d, *J* = 9.0, 1H), 1.99 (s, 1H), 1.90 (s, 1H), 1.70 (m, 2H), 1.46 (d, *J* = 6.0, 1H), 1.43-1.22 (m, 5H), 1.10 (d, *J* = 6.0, 1H), 0.96-0.75 (m, 3H); ^{13}C NMR (126 MHz; C_6D_6): δ 137.6, 115.3, 67.4, 65.8, 63.8, 51.6, 45.2, 38.6, 34.7, 34.3, 33.5, 32.9, 29.3, 23.8, 14.0; HRMS (ESI-TOFMS) Calcd. for $[C_{15}H_{23}NO +H]^+$ = 234.1852, Found = 234.1853; R_f (1% MeOH in DCM): 0.25.

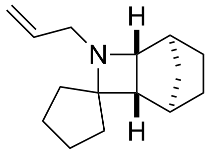

16 was synthesized via general IOPC procedure (48 h reaction time) in 66% yield (49 mg, >95:5 *exo:endo* as determined by a series of multidimensional NMR experiments. See S87-88) as a colorless oil. Analytical data for **16**:

^{1}H NMR (300 MHz; C_6D_6): δ 5.82 (m, 1H), 5.18 (d, *J* = 16.5 Hz, 1H), 4.98 (d, *J* = 8.6 Hz, 1H), 3.18 (d, *J* = 11.0 Hz, 1H), 2.96 (d, *J* = 7.0 Hz, 1H), 2.91 (d, *J* = 3.5 Hz, 1H), 2.55 (d, *J* = 7.0 Hz, 1H), 2.05 (s, 1H), 1.98 (s, 1H), 1.89 (s, 1H), 1.77 (s, 1H), 1.53 (d, *J* = 3.5 Hz, 1H), 1.46-1.35 (m, 8H), 1.16 (d, *J* = 7.0 Hz, 1H), 0.85 (m, 2H); ^{13}C NMR (126 MHz; C_6D_6): δ 137.7, 115.2, 74.1, 67.7, 53.3, 48.3, 39.4, 35.4, 33.8, 32.7, 31.9, 28.9, 24.1, 22.4, 22.1; HRMS (ESI-TOFMS) Calcd. for $[C_{15}H_{23}N +H]^+$ = 218.1903 Found = 218.1905; R_f (1% MeOH in DCM): 0.2.

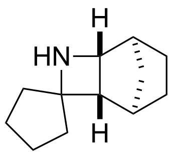

16a was synthesized from **16** according to a known procedure[13] in 82% yield (20 mg, >95:5 *exo:endo*)**.** Analytical data for **16a**:

^{1}H NMR (300 MHz; C_6D_6): δ 3.77 (d, *J* = 4.2 Hz, 1H), 2.40 (d, *J* = 8.8 Hz, 1H), 2.32 (t, *J* = 4.3 Hz, 2H), 2.17 (s, 2H), 2.11 (dd, *J* = 10.6, 6.1 Hz, 1H), 1.75 (d, *J* = 2.4 Hz, 1H), 1.65-1.58 (m, 3H), 1.45-1.32 (m, 4H), 1.28-1.23 (m, 1H), 1.13-1.06 (m, 3H), 0.59-0.54 (m, 2H); ^{13}C NMR (126 MHz; C_6D_6): δ 74.2, 59.3, 48.9, 40.7, 37.5, 36.0, 33.4, 30.8, 27.2, 24.1, 23.2, 22.5; HRMS (ESI-TOFMS) Calcd. for $[C_{12}H_{19}N +H]^+$ = 178.1595, Found = 178.1596; R_f (3% MeOH in DCM): 0.3.

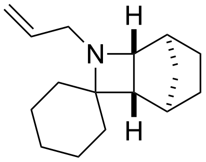

17 was synthesized via general IOPC procedure (24 h reaction time) in 78% yield (57 mg, >95:5 *exo:endo* as determined by analogy to **16**) as a colorless oil. Analytical data for **17**:

^{1}H NMR (300 MHz; C_6D_6): δ 5.85 (m, 1H), 5.19 (d, *J* = 18.0 Hz, 1H), 4.98 (d, *J* = 9.3 Hz, 1H), 3.27 (dd, *J* = 4.5 Hz, *J* = 4.5 Hz , 1H), 2.93 (d, *J* = 6 Hz, 1H), 2.63 (d, *J* = 3.5 Hz, 1H), 2.07-1.94 (m, 3H), 1.66 (d, *J* = 12.0 Hz, 1H), 1.52-0.789 (m, 15H); ^{13}C NMR (126 MHz; C_6D_6): δ 138.2, 114.9, 67.6, 66.0, 51.7, 45.7, 38.8, 34.9, 34.6, 32.8, 32.4, 29.6, 25.8, 24.3, 23.9, 22.5; HRMS (ESI-TOFMS) Calc. for $[C_{16}H_{25}N+H]^+$ = 232.2060, Found = 232.2062; R_f (1% MeOH in DCM): 0.2.

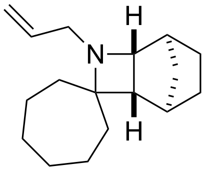

18 was synthesized via general IOPC procedure (24 h reaction time) in 58% yield (45 mg, >95:5 exo:endo as determined by analogy to **16**) as a colorless oil. Analytical data for **18**:

^{1}H NMR (300 MHz; C_6D_6): δ 5.72 (m, 1H), 5.12 (d, *J* = 23.1 Hz, 1H), 4.96 (d, *J* = 13.2 Hz, 1H), 3.25 (dd, *J* = 17.7, 6.1 Hz, 1H), 3.01-2.92 (m, 2H), 2.33 (d, *J* = 11.5 Hz, 1H), 2.24 (s, 1H), 2.12 (s, 1H), 2.00 (dd, *J* = 18.7, 10.2 Hz, 2H), 1.91 (s, 1H), 1.84-1.72 (m, 3H), 1.63 (d, *J* = 8.5 Hz, 1H), 1.50-1.26 (m, 15H), 1.12 (d, *J* = 12.1 Hz, 1H), 0.88 (td, *J* = 25.8, 15.7 Hz, 3H); ^{13}C NMR (126 MHz; C_6D_6): δ 138.2, 115.0, 88.5, 81.0, 68.9, 67.4, 52.3, 47.6, 38.7, 35.7, 35.4, 35.2, 34.5, 29.5, 29.2, 24.0, 22.5; HRMS (ESI-TOFMS) Calcd. for $[C_{17}H_{27}N+H\ ]^+$ = 246.2216, Found = 246.2218; R_f (3% MeOH in DCM): 0.2.

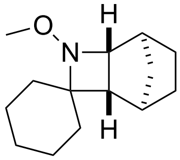

19 was synthesized via general IOPC procedure (48 h reaction time) in 91% yield (64 mg, >95:5 *exo:endo* as determined by analogy to **16**) as a colorless oil. Analytical data for **19**:

^{1}H NMR (300 MHz; C_6D_6): δ 3.53 (d, *J* = 7.5 Hz, 1H), 3.42 (s, 3H), 2.28 (d, *J* = 9.3 Hz, 1H), 2.21 (s, 1H), 1.97-1.85 (m, 4H), 1.64 (d, *J* = 13.3 Hz, 1H), 1.55 (d, *J* = 7.6 Hz, 1H), 1.46 (m, 2H), 1.37-1.22 (m, 5H), 1.10 (m, 3H), 0.86-0.73 (m, 2H); ^{13}C NMR (126 MHz; C_6D_6): δ 72.1, 71.2, 61.9, 44.3, 38.4, 34.9, 34.2, 29.4, 25.9, 23.9, 23.7, 22.3; HRMS (ESI-TOFMS) Calcd. for $[C_{14}H_{23}NO +H]^+$ = 221.852, Found = 221.854; R_f (3% MeOH in DCM): 0.25.

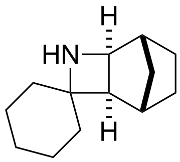

19a was synthesized from **19** according to a known procedure[14] in 93% yield (24 mg, >95:5 *exo:endo*).

^{1}H NMR (300 MHz; C_6D_6): δ 3.50 (d, *J* = 3.1 Hz, 1H), 3.50 (br s, 1H), 2.57 (d, *J* = 7.6 Hz, 1H), 2.12 (s, 1H), 1.98 (s, 1H), 1.65 (s, 1H), 1.54 (m, 2H), 1.40 (s, 4H), 1.29-1.24 (m, 5H), 1.14 (m, 3H), 0.76 (dd, *J* = 19.9, 8.0 Hz, 2H); ^{13}C NMR (126 MHz; C_6D_6): δ 63.86, 60.0, 49.0, 41.0, 39.1, 35.4, 34.2, 32.5, 29.1, 25.9, 24.5, 23.3, 22.8; HRMS (ESI-TOFMS) Calcd. for $[C_{13}H_{21}N +H]^+$ = 192.1752, Found = 192.1751; R_f (3% MeOH in DCM): 0.3.

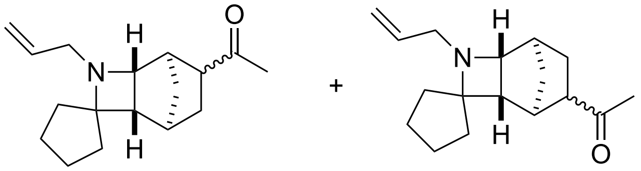

20 was synthesized via general IOPC procedure (48 h reaction time) in 95% yield (54 mg, 65:35 regioisomeric mixture as determined by GC-MS, >95:5 *exo:endo* as determined by analogy to **2**) as a colorless oil. Analytical data for **20**:

^{1}H NMR (300 MHz; C_6D_6): δ 5.72 (m, 1H), 5.17-4.89 (m, 2H), 3.14-2.74 (m, 3H), 2.58 (d, J = 9.0 Hz, 1H), 2.23 (m, 1H), 2.17 (m, 1H), 2.08 (s, 1H), 1.99 (d, 1H), 1.93 (d, J = 3.2 Hz, 1H), 1.85 (d, J = 12.1 Hz, 1H), 1.76 (s, 2H), 1.67 (s, 1H), 1.63-1.57 (m, 2H), 1.44-1.20 (m, 8H), 1.14 (m, 1H); ^{13}C NMR (126 MHz; C_6D_6): δ 206.44, 206.40, 137.3, 115.55, 115.49, 74.6, 73.9, 66.9, 63.6, 54.1, 53.08, 53.06, 51.5, 47.5, 43.04, 42.88, 40.2, 39.1, 36.3, 35.94, 35.81, 32.70, 32.64, 31.82, 31.62, 28.62, 28.57, 28.43, 24.4, 22.31, 22.30, 22.09, 22.05; HRMS (ESI-TOFMS) Calcd. for $[C_{17}H_{25}NO +H]^+$ = 260.2009, Found = 260.2010; R_f (3% MeOH in DCM): 0.2.

Supplementary Figure 4. Gas chromatograph of diastereomers of **20**

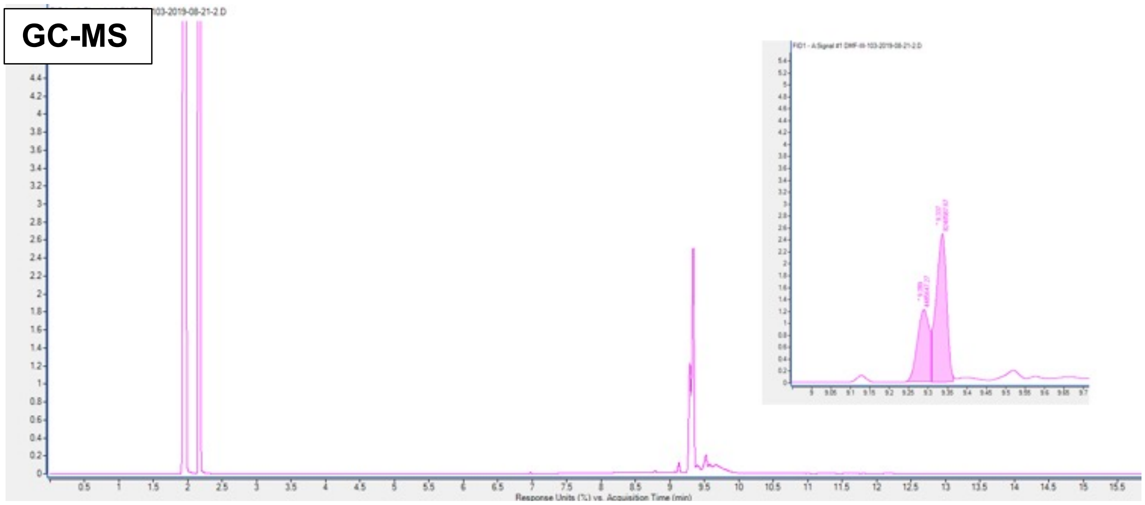

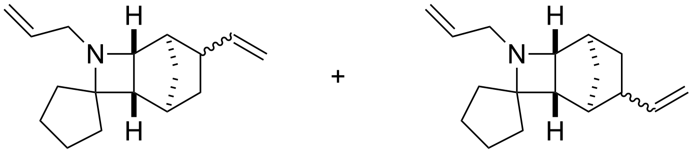

21 was synthesized via general IOPC procedure (48 h reaction time) in 85% yield (52 mg, 56:44 regiomeric mixture as determined by GC-MS, 75:25 diastereomeric ratio as determined by GC-MS, >95:5 *exo:endo* as determined by analogy to **2**) as a colorless oil. Analytical data for **21**:

^{1}H NMR (300 MHz; C_6D_6): δ 5.73 (m, 2H), 5.13 (t, *J* = 15.5 Hz, 1H), 4.93 (m, 3H), 3.33 (d, 1H), 3.14 (m, 1H), 2.94-2.86 (m, 1H), 2.60 (t, *J* = 9.4 Hz, 1H), 2.40 (m, 1H), 2.10 (s, 1H), 2.01-1.90 (m, 2H), 1.82 (m, 1H), 1.74 (m, 1H), 1.65-1.49 (m, 2H), 1.36-1.15 (m, 9H), 1.10 (m, 1H), 0.76 (d, *J* = 11.0 Hz, 1H), 0.63 (d, *J* = 7.2 Hz, 1H); ^{13}C NMR (126 MHz; C_6D_6): δ 143.58, 143.46, 140.94, 140.82, 137.63, 137.53, 115.32, 115.27, 114.2, 113.6, 111.9, 111.6, 74.5, 73.98, 73.85, 73.67, 67.6, 67.33, 67.20, 63.7, 53.38, 53.24, 53.23, 53.18, 48.60, 48.54, 48.1, 45.5, 45.13, 44.97, 44.3, 42.09, 42.04, 41.4, 41.1, 40.4, 39.8, 36.66, 36.52, 36.0, 35.53, 35.43, 34.3, 32.99, 32.88, 32.68, 32.63, 31.82, 31.73, 31.55, 31.43, 30.9, 29.9, 29.5, 22.49, 22.39, 22.34, 22.21, 22.17, 22.13, 22.07; HRMS (ESI-TOFMS) Calcd. for $[C_{17}H_{25}N + H]^+$ = 244.2060, Found = 244.2062; R_f (3% MeOH in DCM): 0.25.

Supplementary Figure 5. Gas chromatograph of diastereomers of **21**

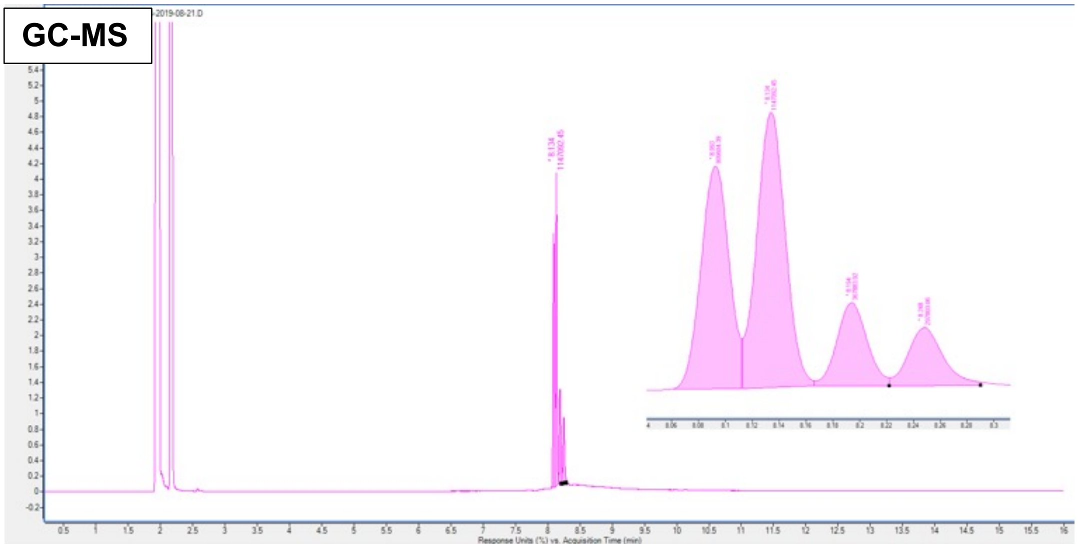

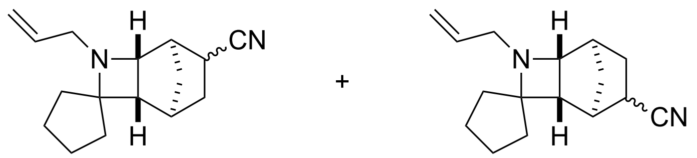

22 was synthesized via general IOPC procedure (48 h reaction time) in 47% yield (29 mg, 50:50 regiomeric mixture as determined by GC-MS, 60:40 diastereomeric ratio as determined by GC-MS, >95:5 *exo*:*endo* as determined by analogy to **2**) as a colorless oil. Analytical data for **22**:

^{1}H NMR (300 MHz; C_6D_6): δ 5.63 (m 1H), 5.07 (m, 1H), 4.92 (m, 1H), 3.61 (d, 1H), 2.99 (m, 1H), 2.76 (m, 1H); ^{13}C NMR (126 MHz; C_6D_6): δ 137.05, 137.04, 136.89, 136.82, 122.74, 122.66, 121.5, 121.2, 115.91, 115.79, 115.63, 115.60, 74.4, 74.02, 73.93, 73.7, 66.1, 65.8, 65.5, 63.7, 52.87, 52.83, 52.71, 47.1, 46.7, 44.4, 43.3, 41.7, 40.7, 39.3, 38.8, 35.5, 35.12, 35.05, 34.3, 33.94, 33.92, 32.75, 32.60, 32.56, 32.53, 32.48, 32.43, 32.1, 31.85, 31.71, 31.67, 31.59, 30.3, 29.93, 29.87, 29.6, 29.16, 29.12, 25.9, 25.6, 22.45, 22.25, 22.21, 22.19, 22.14, 21.99, 21.96, 21.91; HRMS (ESI-TOFMS) Calcd. for $[C_{16}H_{23}N + H]^+$ = 243.1856, Found = 243.1858; R_f (3% MeOH in DCM): 0.2.

Supplementary Figure 6. Gas chromatograph of diastereomers of **22**

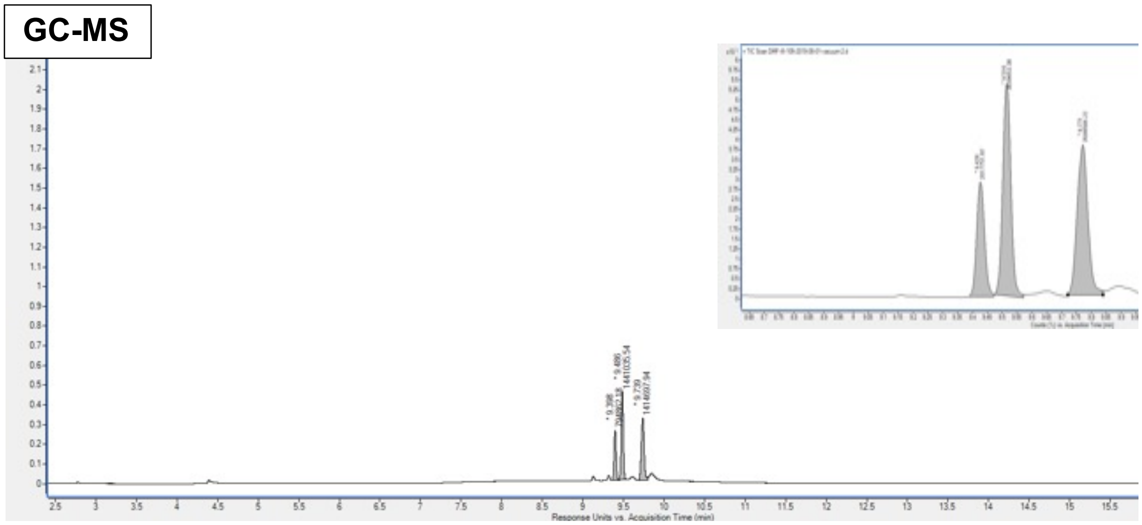

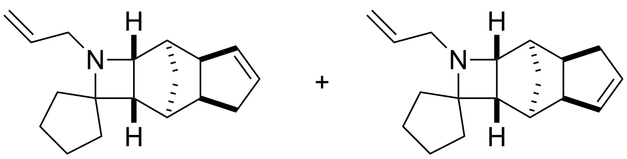

23 was synthesized via general IOPC procedure (60 h reaction time) in 82% yield (47 mg, 53:47 diastereomeric ratio as determined by GC-MS, >95:5 *exo:endo* at azetidine juncture as determined by X-ray crystallography) as a colorless oil. Analytical data for **23**:

^{1}H NMR (300 MHz; C_6D_6): δ 5.79 (m, 1H), 5.46 (m, 2H), 5.14 (dd, *J* = 17.1, 6.4 Hz, 1H), 4.95 (d, *J* = 7.1 Hz, 1H), 3.18 (m, 2H), 3.03 (m, 1H), 2.92 (m, 2H), 2.71-2.69 (m, 1H), 2.45 (m, 1H), 2.18-2.00 (m, 4H), 1.91-1.77 (m, 4H), 1.35 (m, 8H); ^{13}C NMR (126 MHz; C_6D_6): δ 137.77, 137.72, 131.4, 131.10, 131.03, 130.2, 115.20, 115.11, 74.53, 74.38, 65.1, 63.0, 53.49, 53.41, 53.31, 51.3, 44.4, 43.9, 42.22, 42.08, 41.1, 40.4, 39.9, 38.15, 38.04, 37.1, 33.16, 33.00, 32.12, 32.07, 31.3, 31.0, 22.57, 22.45, 22.38, 22.1; HRMS (ESI-TOFMS) Calcd. for $[C_{18}H_{25}N + H]^+$ = 256.2060, Found = 256.2061; R_f (3 % MeOH in DCM): 0.3.

Supplementary Figure 7. Gas chromatograph of diastereomers of **23**

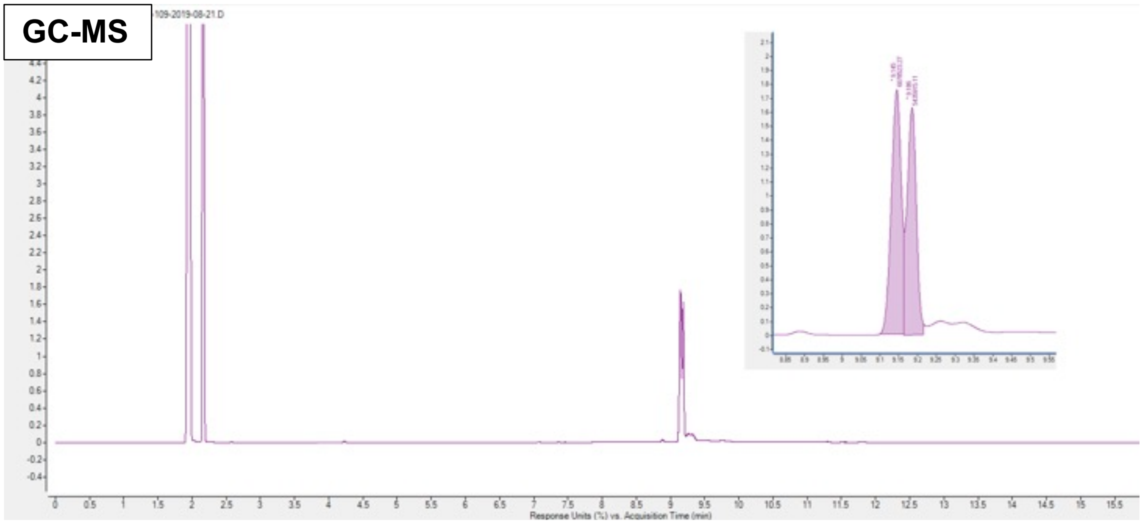

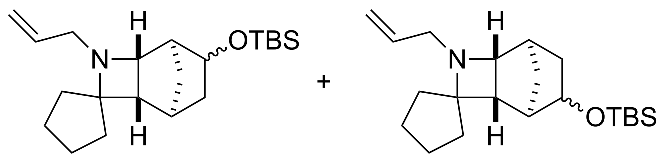

24 was synthesized via general IOPC procedure (192 h reaction time) in 82% yield (28 mg, 50:50 regiomeric mixture as determined by GC-MS, 90:10 diastereomeric ratio as determined by GC-MS, >95:5 *exo:endo* as determined by analogy to **2**) as a colorless oil. Analytical data for **24**:

^{1}H NMR (300 MHz; C_6D_6): δ 5.79 (m, 1H), 5.46 (m, 2H), 5.14 (dd, *J* = 17.1, 6.4 Hz, 1H), 4.95 (d, *J* = 7.1 Hz, 1H), 3.18 (m, 2H), 3.03 (m, 1H), 2.92 (m, 2H), 2.71-2.69 (m, 1H), 2.45 (m, 1H), 2.18-2.00 (m, 4H), 1.91-1.77 (m, 4H), 1.35 (m, 8H); ^{13}C NMR (126 MHz; C_6D_6): δ 137.77, 137.72, 131.4, 131.10, 131.03, 130.2, 115.20, 115.11, 74.53, 74.38, 65.1, 63.0, 53.49, 53.41, 53.31, 51.3, 44.4, 43.9, 42.22, 42.08, 41.1, 40.4, 39.9, 38.15, 38.04, 37.1, 33.16, 33.00, 32.12, 32.07, 31.3, 31.0, 22.57, 22.45, 22.38, 22.1; HRMS (ESI-TOFMS) Calcd. for $[C_{18}H_{25}N + H]^+$ = 256.2060, Found = 256.2061; R_f (2% MeOH in DCM): 0.25.

Supplementary Figure 8. Gas chromatograph of diastereomers of **24**

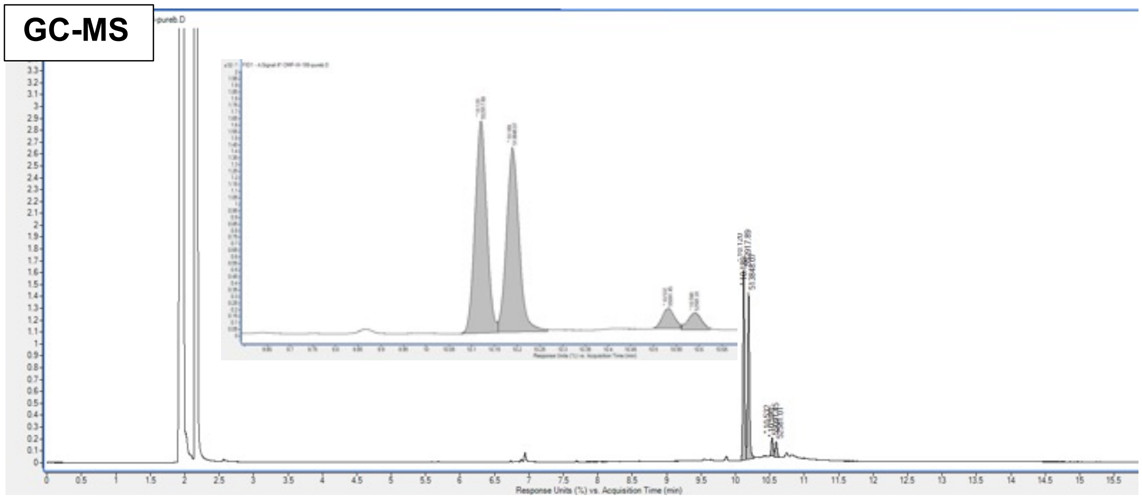

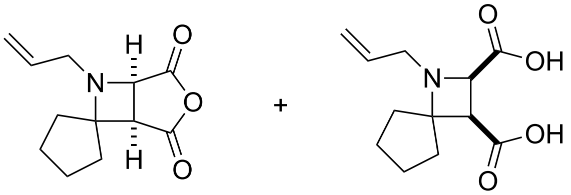

25 was synthesized via general IOPC procedure (24 h reaction time) in 98% combined yield (67 mg, >95:5 *exo:endo*) as a colorless oil. Without TpCu, 51% yield (39 mg). Both the anhydride and di-acid are present in the sample and the following spectral lists are of the mixture. Characterization data for **25**:

^{1}H NMR (300 MHz; C_6D_6): δ 8.48 (s, 1H), 5.76 (m, 1H), 5.17-5.07 (m, 2H), 4.96 (s, 1H), 4.33-4.24 (m, 1H), 4.04 (m, 1H), 3.31 (s, 1H), 2.96 (m, 1H), 2.86 (m, 1H), 2.75 (m, 1H), 2.63-2.54 (m, 2H), 2.39 (m, 2H), 2.29 (t, *J* = 8.6 Hz, 1H), 1.98 (m, 1H), 1.65 (quintet, *J* = 10.4 Hz, 1H); ^{13}C NMR (126 MHz; C_6D_6): δ 168.8, 167.9, 141.0, 139.0, 133.2, 131.8, 117.0, 115.7, 111.4, 105.7, 46.0, 45.2, 44.0, 34.6, 33.5, 32.1, 30.8, 29.7, 29.2, 21.3; HRMS (ESI-TOFMS) Calcd. for $[C_{12}H_{15}NO_3 - H]^-$ = 220.0979, Found = 220.0981; Calcd. for $[C_{12}H_{16}NO_4 - H]^-$ = 238.1082, Found = 238.1085.

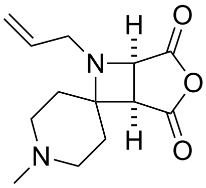

26 was synthesized via general IOPC procedure (24 h reaction time) in 68% yield (52 mg, >95:5 *exo:endo*) as a colorless oil. Without TpCu, 13% yield (10 mg). Analytical data for **26**:

^{1}H NMR (300 MHz; C_6D_6): δ 6.04 (d, *J* = 9.6 Hz, 1H), 5.78 (d, *J* = 9.8 Hz, 2H), 5.29 (br s, 3H), 4.56 (s, 1H), 3.84 (m, 1H), 3.13 (m, 1H), 2.77-2.73 (m, 1H), 1.43 (s, 2H), 1.32 (s, 1H), 1.22-1.02 (m, 5H), 0.78-0.63 (m, 7H), 0.38 (s, 1H), 0.26 (m, 1H), 0.17-0.10 (m, 2H), -0.13 (m, 1H); ^{13}C NMR (126 MHz; C_6D_6): δ 165.1, 164.4, 152.8, 139.8, 135.2, 105.1, 89.4, 41.8, 32.3, 20.5, 13.9, 11.9, 4.5, 2.7; HRMS (ESI-TOFMS) Calcd. for $[C_{13}H_{18}N_2O_3 + H]^+$ = 251.1395, Found = 251.1393.

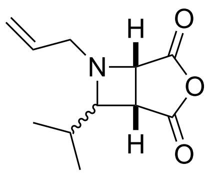

27 was synthesized via general IOPC procedure (24 h reaction time) in 96% yield (47 mg, 75:25 diastereomeric mixture at C2 as determined by GC-MS, >95:5 *exo:endo*) as a colorless oil. Without TpCu**,** 47% yield. Analytical data for **27**:

^{1}H NMR (300 MHz; C_6D_6): δ 5.76-5.63 (m, 1H), 5.30-5.22 (m, 2H), 4.99 (d, *J* = 1.7 Hz, 1H), 4.28 (dd, *J* = 15.1, 5.6 Hz,), 4.18 (dd, *J* = 15.1, 5.2 Hz, 1H), 3.84 (dd, *J* = 15.0, 7.3 Hz,), 3.69 (dd, *J* = 15.0, 7.8 Hz, 1H), 2.88 (dd, *J* = 19.2, 5.1 Hz, 1H), 2.80 (d, *J* = 1.3 Hz, 1H), 2.74-2.67 (m,), 2.61 (d, *J* = 1.4 Hz,), 2.51 (d, *J* = 1.8 Hz,), 2.36-2.35 (m, 1H), 1.23-1.14 (m, 6H); ^{13}C NMR (126 MHz; C_6D_6): δ 176.3, 175.6, 166.5, 166.0, 132.6, 132.1, 118.7, 117.5, 93.4, 48.2, 46.7, 45.9, 43.0, 41.3, 39.2, 33.4, 32.9, 23.7, 23.1, 17.42, 17.28; HRMS (ESI-TOFMS) Calcd. for $[C_{11}H_{15}N +H]^{+}$ = 162.1282, Found = 162.1283.

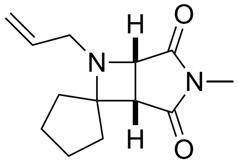

28 was synthesized via general IOPC procedure (24 h reaction time) in 49% yield (33 mg, 95:5 *exo:endo*) as a colorless oil. Without TpCu, 25% yield. Analytical data for **28**:

^{1}H NMR (300 MzHz; C_6D_6): 5.88 (m, 1H), 5.18-5.02 (m, 2H), 3.56-3.43 (m, 2H), 2.86 (m, 3H), 2.72 (s, 1H), 2.63 (m, 1H), 2.45 (m, 1H), 2.38 (s, 1H), 2.29 (m, 1H), 2.03 (m, 1H), 1.71-1.59 (m, 1H), 1.37 (m, 4H), 1.05 (m, 1H), 0.76 (m, 1H); ^{13}C NMR (126 MHz; C_6D_6): δ 179.3, 179.1, 178.1, 176.60, 176.52, 176.33, 136.62, 136.58, 114.69, 114.62, 55.70, 55.65, 47.4, 47.2, 40.4, 40.1, 33.0, 31.4, 28.88, 28.82, 28.5, 27.0, 24.6, 24.3, 22.43, 22.33; HRMS (ESI-Orbitrap) Calcd. for $[C_{13}H_{21}N +H]^{+}$ = 221.1441, Found = 235.1443.

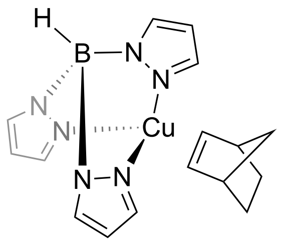

TpCu-NB was synthesized via the equimolar combination of TpCu (294 mg) and norbornene (100 mg) in THF (6 mL). After allowing the solution to stir for 15 min the solution was filtered solvent was removed and the title compound was obtained in 87% yield (344 mg) as a white solid. Recrystallization from DCM/pentane via vapor diffusion at -30 °C led to crystals suitable for X-ray diffraction. Analytical data for TpCu-NB:

^{1}H NMR (300 MHz; C_6D_6): 7.52 (s, 3H), 7.39 (s, 3H), 5.95 (s, 3H), 4.95 (br s, 2H), 2.79 (s, 2H), 1.62 (br s, 1H), 1.37 (d, J = 7.4 Hz, 2H), 0.89-0.83 (m, 3H), 0.67 (d, J = 9.0 Hz, 1H); ^{13}C NMR (126 MHz; C_6D_6): δ 139.4, 134.2, 104.0, 42.3, 25.0; Analysis (calcd., found for C16H20N6BCu): C (51.84, 47.36), H (5.44, 4.62), N (22.67, 2053).

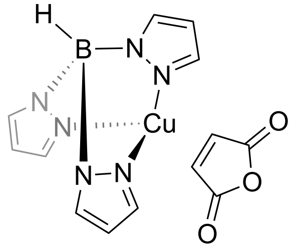

TpCu-MA was synthesized via the equimolar combination of TpCu (282 mg) and maleic anhydride (100 mg) in THF (6 mL). After allowing the solution to stir for 15 min the solution was filtered, and solvent was removed and the title compound was obtained in 93% yield (354 mg) as a yellow-orange solid. Recrystallization from DCM/pentane via vapor diffusion at -30 °C led to crystals suitable for X-ray diffraction Analytical data for TpCu-MA:

^{1}H NMR (300 MHz; C_6D_6): 7.39 (s, 3H), 7.33 (s, 3H), 5.81 (s, 3H), 4.90 (s, 2H); ^{13}C NMR (126 MHz; C_6D_6): δ 165.4, 140.2, 135.4, 105.3; analysis (calcd., found for C13H12N6O3BCu): C (41.68, 42.41), H (3.23, 3.35), N (22.43, 21.94).

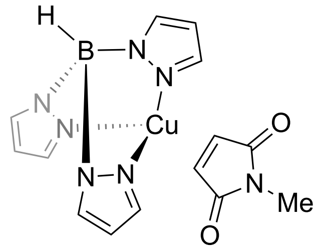

TpCu-MI was synthesized via the equimolar combination of TpCu (142 mg) and *N*-methyl maleimide (50 mg) in THF (6 mL). After allowing the solution to stir for 15 min the solution was filtered and solvent was removed and the title compound was obtained in in 91% yield (175 mg) as a yellow-orange solid. Analytical data for TpCu-MI:

^{1}H NMR (300 MHz; C_6D_6): 7.57 (s, 3H), 7.36 (s, 3H), 5.86 (s, 3H), 5.03 (s, 2H), 2.55 (s, 3H); ^{13}C NMR (126 MHz; C_6D_6): δ 171.0, 139.6, 134.8, 104.6; analysis (calcd., found for C14H15N7O2BCu): C (41.79, 40.52), H (3.51, 3.36), N (26.24, 22.57)

F. Stern-Volmer Quenching Studies.

Stern-Volmer luminescence quenching experiments were run with freshly prepared solutions of 3.0 x 10^{-4} M TpCu in diethyl ether inside of a dry nitrogen filled glovebox. The solutions were irradiated at 294 nm and luminescence was measured at 350 nm. Each sample was prepared three times, the luminescence was acquired and averaged. The average of the results was used for the graphical representation and determination of K_{SV}. The data show that norbornene and *N*-isobutylidene butylamine are unable to quench the excited state of the photocatalyst, however there was an observed concentration dependence of maleic anhydride and luminescence quenching. For all tabular and graphical data, see **Supplementary Tables 1- 4** and **Supplementary Figures 9-16** below.

Supplementary Table 1. Fluorescence quenching data with solution of TpCu and norbornene. See **Supplementary Figures 9-10.**

Sample	1	2	3	4	5	6
**Molarity**	0	3.00E-04	6.00E-04	1.20E-03	2.40E-03	4.80E-03
**Trial 1**	1425270	2151096	1913457	2038849	2014523	1995782
**Trial 2**	1520302	1931018	1987233	2001444	2038714	2043452
**Trial 3**	1462478	1986729	1891140	2186087	2078013	2038556
**Average**	1469350	2023281	1930610	2075460	2043750	2025930

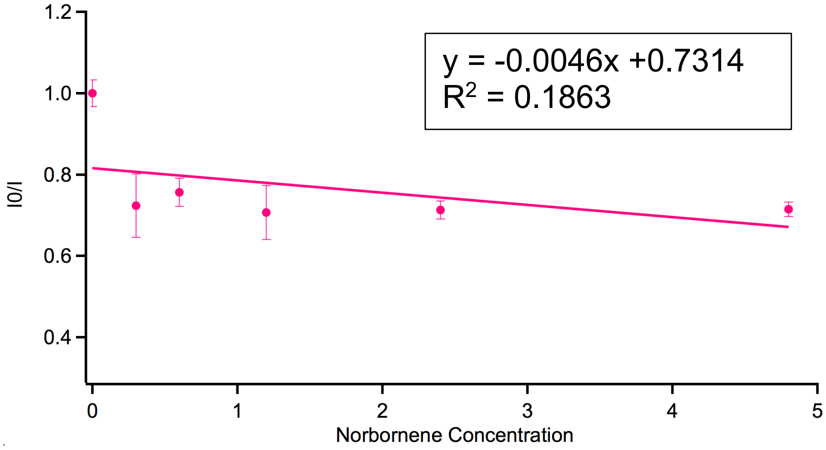

Supplementary Figure 9. Stern-Volmer plot of TpCu quenching with varying concentration of norbornene. Error bars define ± one standard deviation.

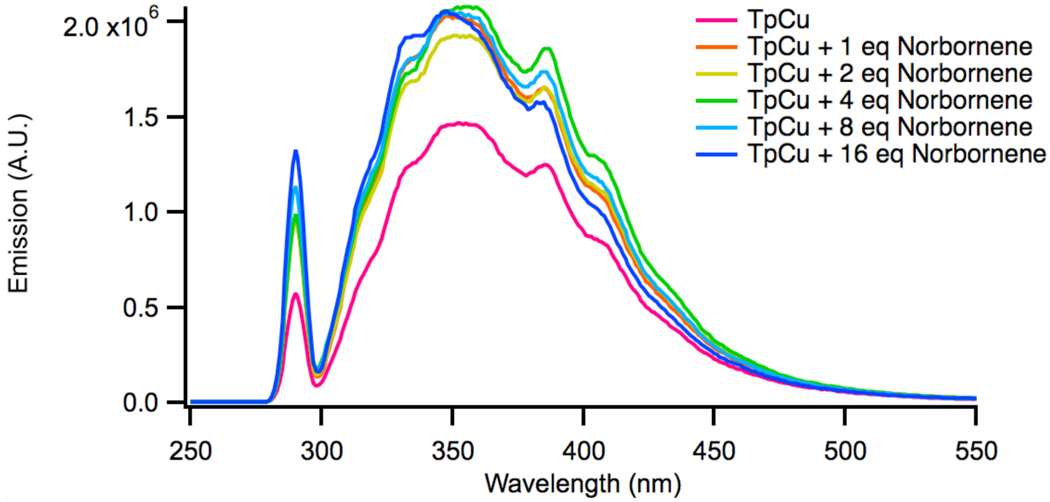

Supplementary Figure 10. Emission plot of TpCu with varying concentration of norbornene. A. U. = arbitrary units.

Supplementary Table 2. Fluorescence quenching data with solution of TpCu and *N*-isobutylidene butylamine. See **Supplementary Figures 11-12.**

Sample	1	2	3	4	5	6
**Molarity**	0	3.00E-04	6.00E-04	1.20E-03	2.40E-03	4.80E-03
**Trial 1**	1425270	1602578	1630246	1685493	1856721	1882367
**Trial 2**	1520302	1649876	1648736	1734569	1803564	1934873
**Trial 3**	1462478	1642331	1641348	1741330	1809105	1888850
**Average**	1469350	1631595	1640110	1720464	1823130	1902030

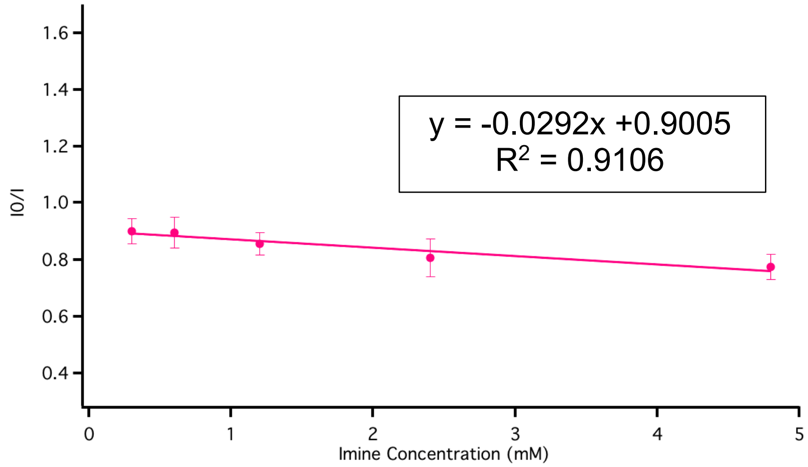

Supplementary Figure 11. Stern-Volmer plot of TpCu quenching with varying concentration of *N*- isobutylidene butylamine. Error bars define ± one standard deviation.

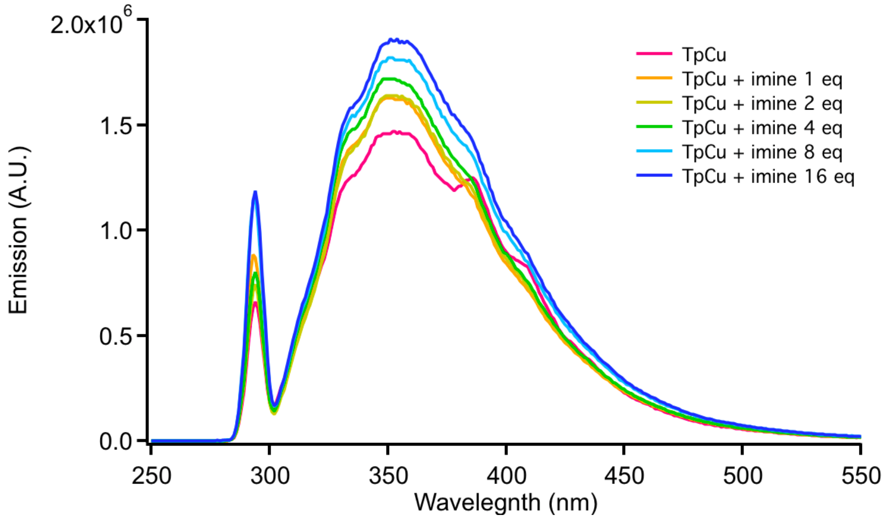

Supplementary Figure 12. Emission plot of TpCu with varying concentration of *N*-isobutylidene butylamine. A. U. = arbitrary units.

Supplementary Table 3. Fluorescence quenching data with solution of TpCu and maleic anhydride. See **Supplementary Figures 13-14.**

Sample	1	2	3	4	5	6
Molarity (M)	0	3.00E-04	6.00E-04	1.20E-03	2.40E-03	4.80E-03
Trial 1	1425270	1035387	740256	369987	209871	87652
Trial 2	1520302	1005987	743572	376548	235783	91245
Trial 3	1462478	1048686	742622	380475	232894	93023
Average	1469350	1030020	742150	375670	233140	90640
Correction factor*	1	1.024	1.049	1.100	1.210	1.463
Corrected Average*	1469350	1054833	778338	413199	282048	132657

*A correction factor due to the primary inner-filter effect was calculated following the method reported by Albinsson *et. al*.[15]

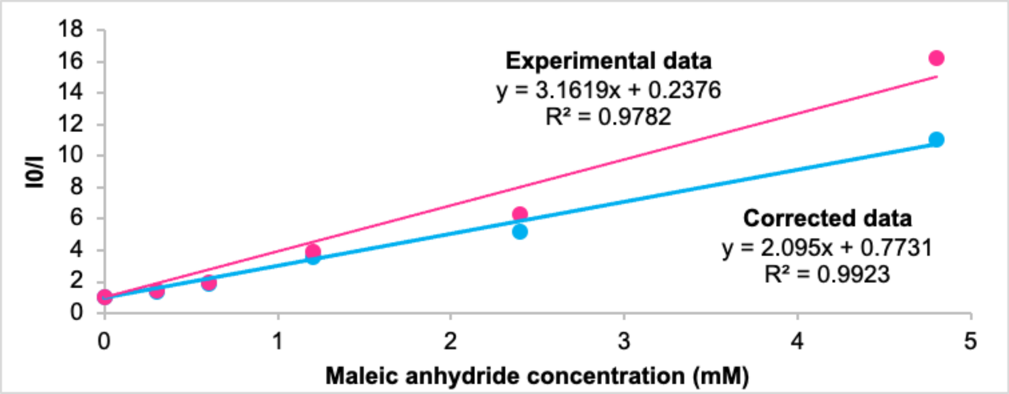

Supplementary Figure 13. Stern-Volmer plot of TpCu quenching with varying concentration of maleic anhydride.

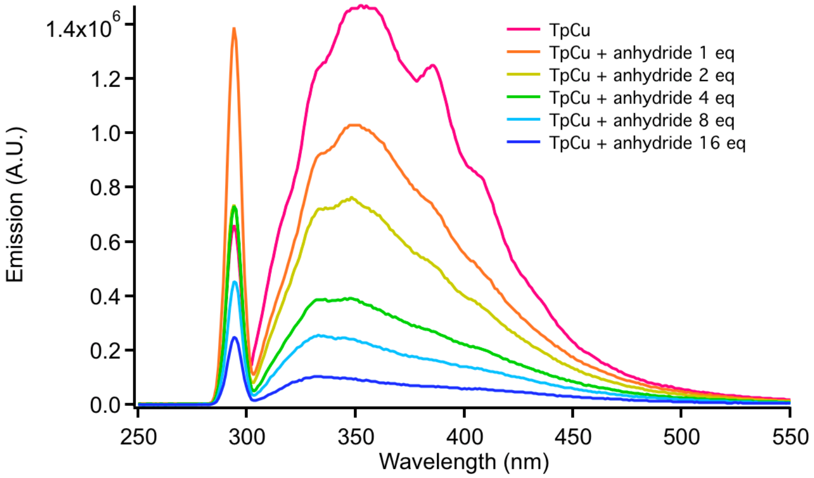

Supplementary Figure 14. Stern-Volmer plot of TpCu quenching with varying concentration of maleic anhydride. A. U. = arbitrary units.

Supplementary Table 4. Fluorescence quenching data with solution of TpCu-NB and *N*-isobutylidene butylamine. See **Supplementary Figures 15-16**.

Sample	1	2	3	4	5	6
**Molarity**	0	3.00E-04	6.00E-04	1.20E-03	2.40E-03	4.80E-03
**Trial 1**	2003568	1880000	1824000	1952000	1927600	1800000
**Trial 2**	2053482	1930000	1887000	1889000	1883600	1850000
**Trial 3**	2012793	1950000	1878000	1859000	1875000	1870000
**Average**	2023281	1920000	1863000	1900000	1895400	1840000

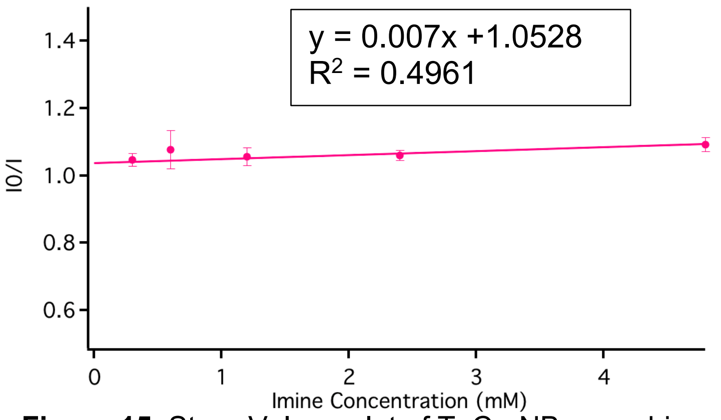

Supplementary Figure 15. Stern-Volmer plot of TpCu-NB quenching with varying concentration of *N*-isobutylidene butylamine.

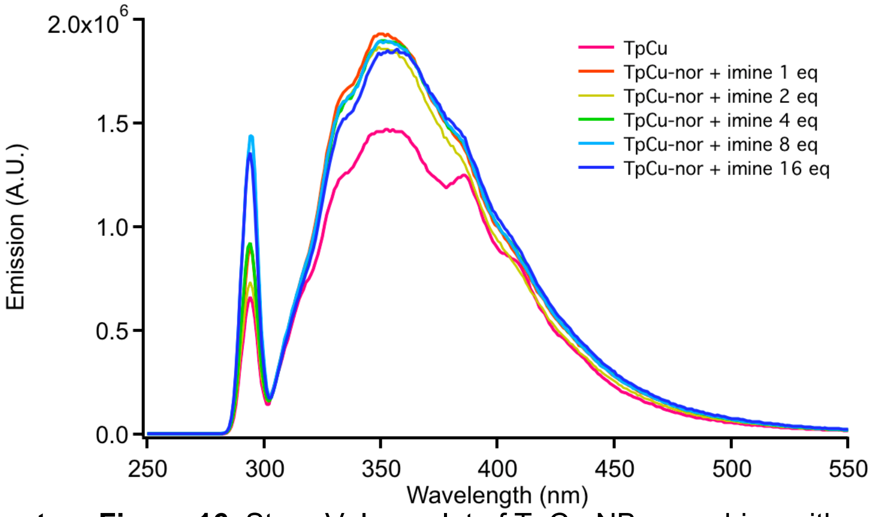

Supplementary Figure 16. Stern-Volmer plot of TpCu-NB quenching with varying concentration of *N*-isobutylidene butylamine. A. U. = arbitrary units.

G. Electronic Absorption Spectra.

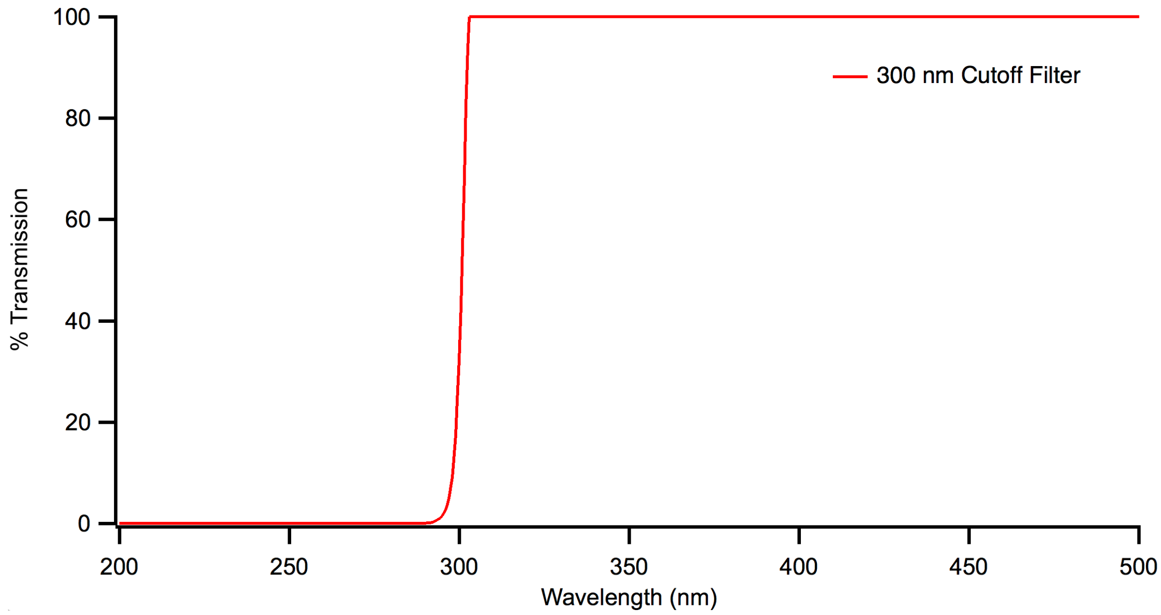

Supplementary Figure 17. Transmission spectrum of Asahi Spectra long pass 300 nm cut-on filter.

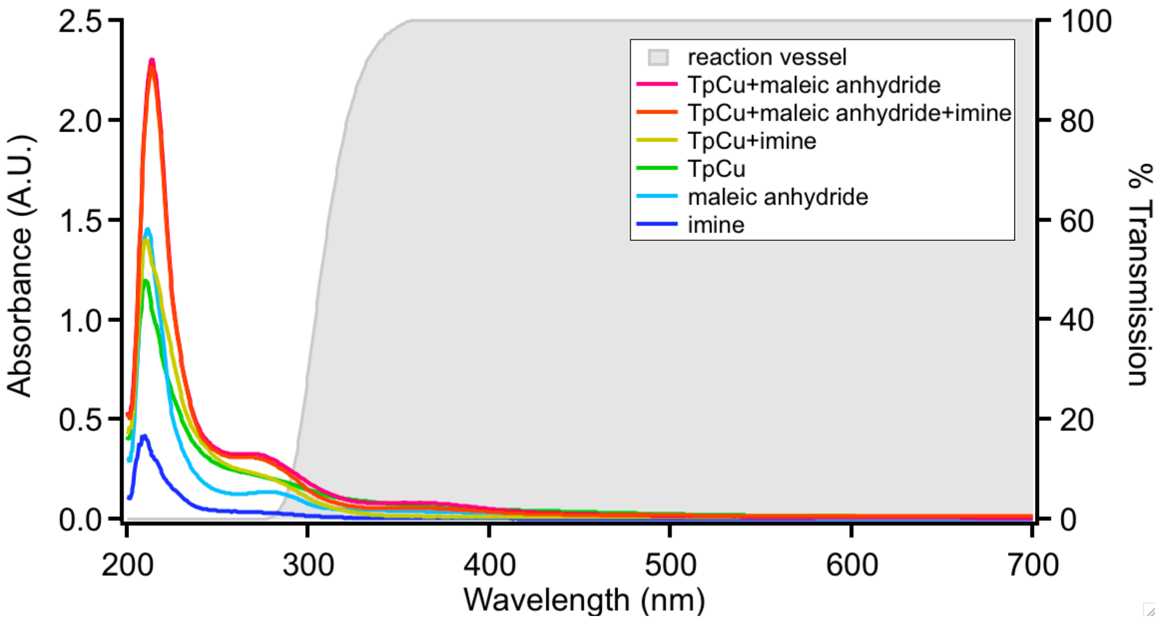

Supplementary Figure 18. Electronic absorption spectra of TpCu and reagents. Imine used was *N*-isobutylidene butylamine. A. U. = arbitrary units.

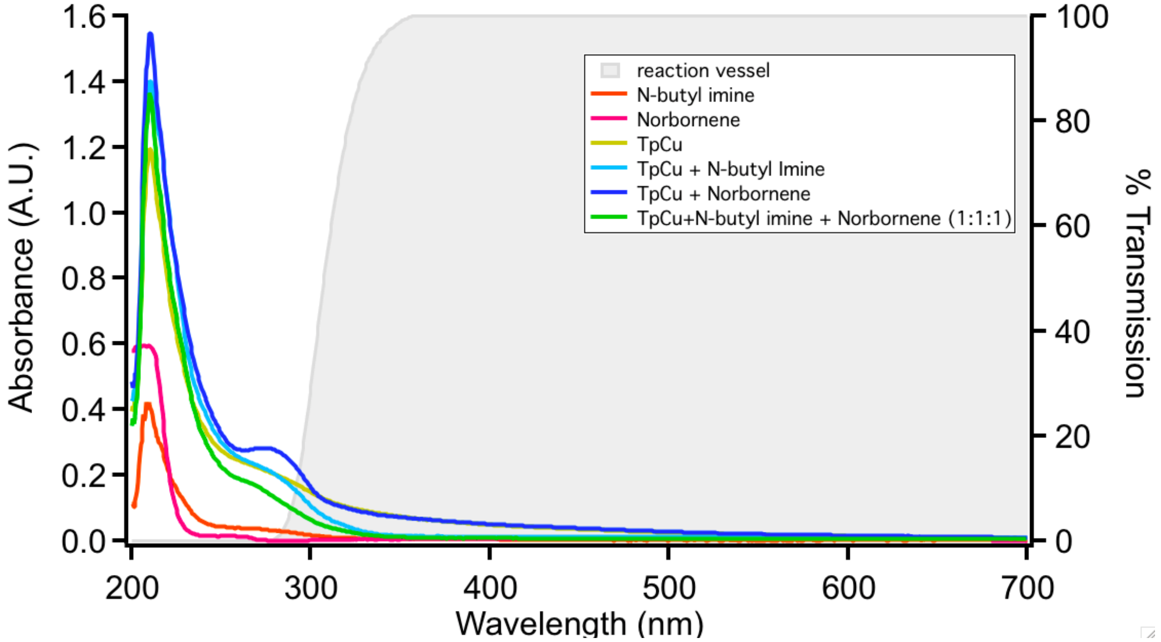

Supplementary Figure 19. Electronic absorption spectra of TpCu and reagents. Imine used was *N*-isobutylidene butylamine. A. U. = arbitrary units.

H. NMR Studies.

NMR coordination experiments were conducted with freshly prepared solutions of 0.11 M TpCu in dry, degassed benzene-d_6 at room temperature inside a dry nitrogen filled glovebox. From a stock solution of *N*-isobutylidene butylamine, maleic anhydride, or norbornene were added into the NMR tube. The sample was sealed with electrical tape, and spectrum obtained. Samples were brought back into the glove box and the process was repeated. For all graphical data see **Supplementary Figure 20** and **21**.

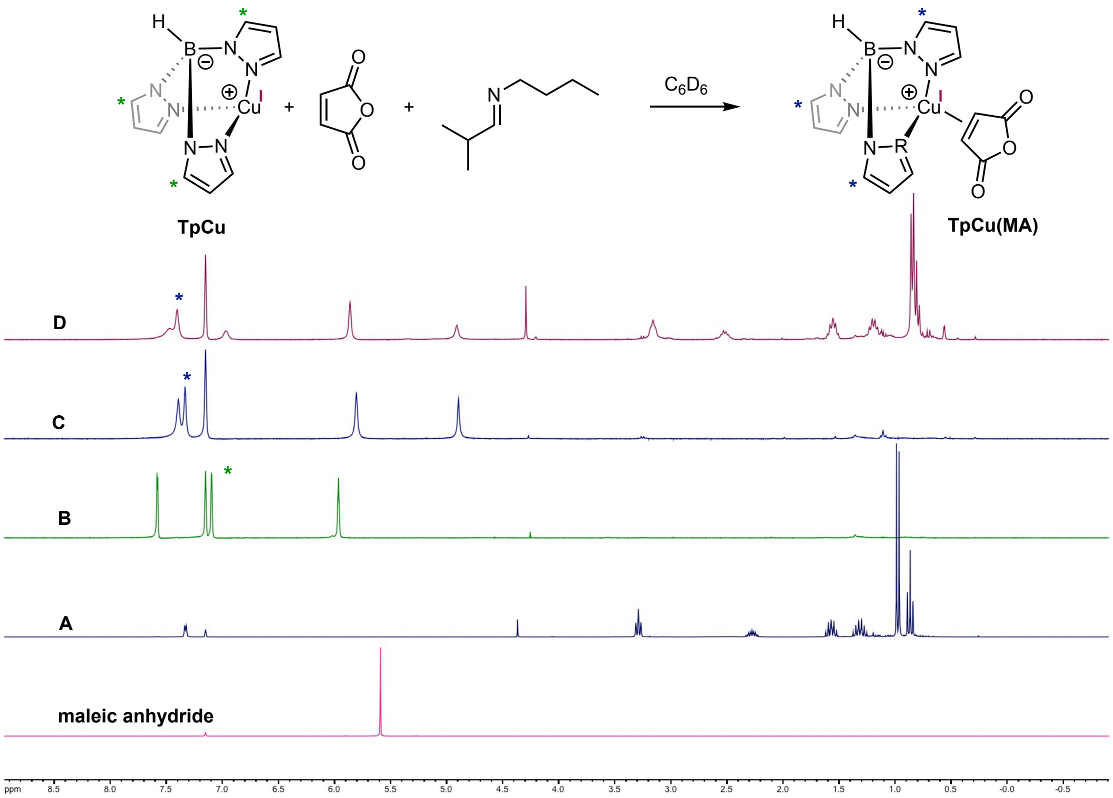

Supplementary Figure 20. ^{1}H NMR spectra of TpCu with 1.0 equivalents of maleic anhydride and/or *N*-isobutylidene butylamine. Spectrum A = *N*-isobutylidene butylamine only (0.11 M in benzene-d_6 with respect to imine). Spectrum B = 1 equiv TpCu (0.11 M in benzene-d_6 with respect to TpCu). Spectrum C = 1 equiv TpCu + 1.0 equiv maleic anhydride (0.11 M in benzene-d_6 with respect to TpCu). Spectrum D = 1 equiv TpCu + 1 equiv maleic anhydride (0.11 M in benzene-d_6 with respect to TpCu) + 1 equiv imine (0.11 M in benzene-d_6 with respect to TpCu).

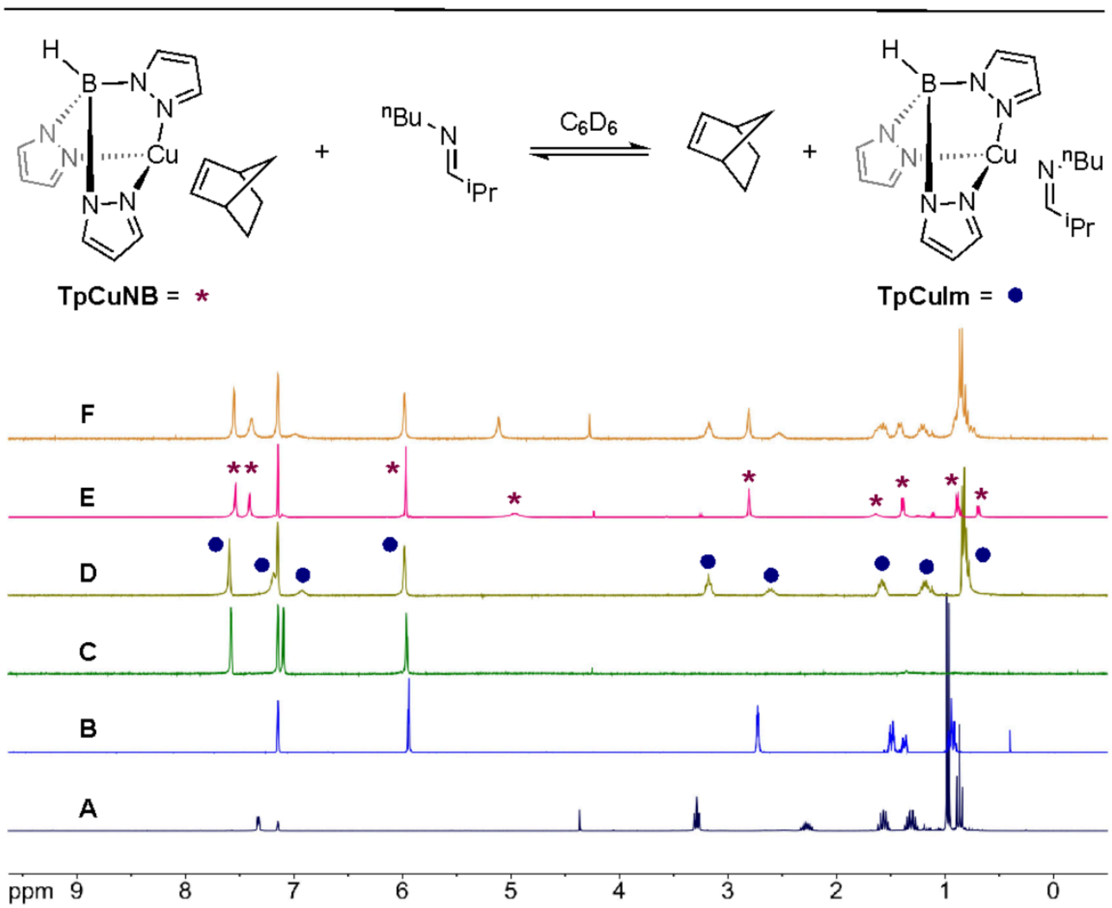

Supplementary Figure 21. ^{1}H NMR spectra. Spectrum A = *N*-isobutylidene butylamine only (0.11 M in benzene-d_6 with respect to imine). Spectrum B = Norbornene (0.11 M in benzene-d_6 with respect to norbornene). Spectrum C = TpCu (0.11 M in benzene-d_6 with respect to TpCu). Spectrum D = 1 equiv TpCu + 1 equiv *N*-isobutylidene butylamine (0.11 M in benzene-d_6 with respect to TpCu). Spectrum E = 1 equiv TpCu + 1 equiv norbornene (0.11 M in benzene-d_6 with respect to TpCu). Spectrum F = 1 equiv TpCu + 1 equiv norbornene (0.11 M in benzene-d_6 with respect to TpCu) + 1 equiv imine (0.11 M in benzene-d_6 with respect to TpCu).

I. Computational Details

Geometry optimizations for TpCu-NB were done starting from the crystallographic atomic coordinates obtained from the solved X-ray crystal structure. Ground state geometry optimizations and frequency calculations were performed using the M06 density functional [16, 17] with Ahlrichs' def2-tzvpp basis set [18] using the Gaussian09 software package. This research was supported in part by the W. M. Keck Foundation through computing resources at the W. M. Keck Laboratory for Integrated Biology II.

TpCu-NB and its excited states were analyzed via time-dependent density functional theory (TD-DFT) calculations (first ten excited states) using the M06 density functional[16,17] with Ahlrichs' def2-tzvpp provided a theoretical electronic absorption spectrum that most closely resembled the experimental spectra (see **Supplementary Figure 22**). From this an absorption band at 265 nm, which corresponds to an excitation from the HOMO - 1 (MO 95) to the LUMO + 1 (MO 97) could be computed. Further absorption bands were detected at 254 nm, and 221 nm. MO 95 was chosen to visualize because it contributes (44.0%) the most significantly in the excitation at 265 nm. While there are various HOMOs contributing to the possible transitions to the excited state each is primarily metal based (see below images). Therefore, the observed excitation can be interpreted as a metal to ligand charge transfer (MLCT). The HOMO shows significant contributions from the Cu d_{yz} orbital, and the LUMO shows contribution from the Cu d_{xy} orbital and norbornene π* orbital.

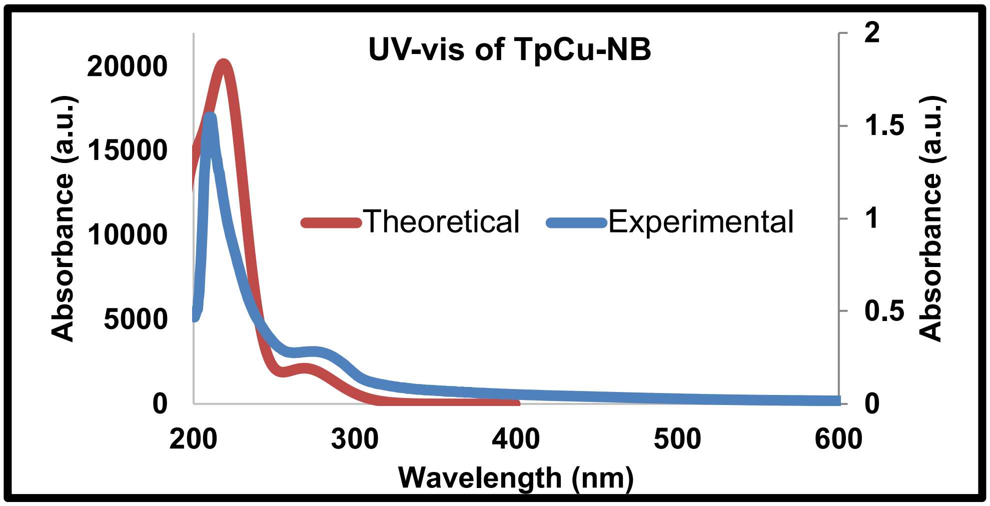

Supplementary Figure 22. Theoretical (TD-DFT M06/def2-tzvpp) and experimental electronic absorption spectra of TpCu-NB. a. u. = arbitrary units.

TpCu-NB was analyzed via TDA-DFT using other functionals such as B3LYP, PBE, ωb97x-D, TPSS, and M06 with various basis sets including 6-311+g(2d,p), def2-svp, and def2-tzvp gave theoretical UV-vis spectra that poorly corresponded to the experimental data (see **Supplementary Figure 23**).

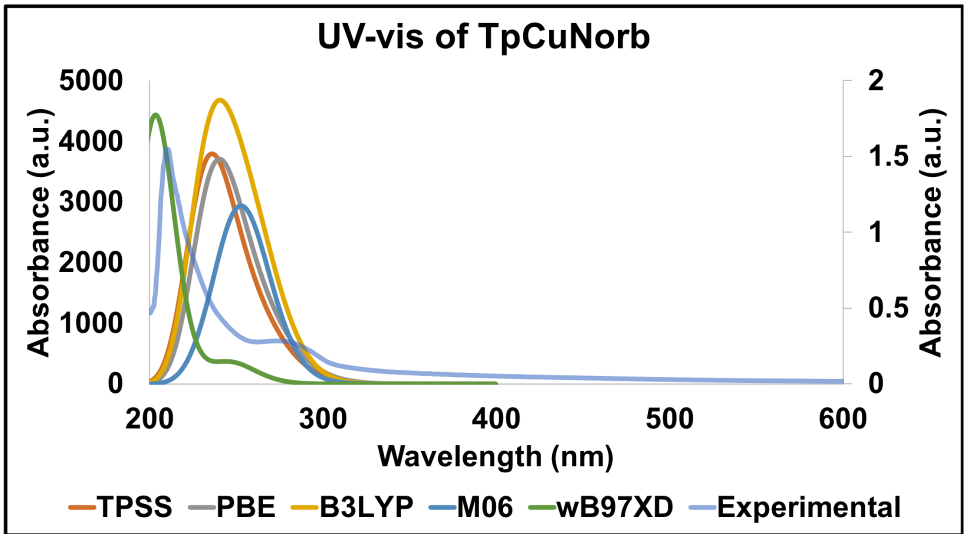

Supplementary Figure 23. Theoretical electronic absorption spectra of TpCu-NB using various functionals with the def2-tzvpp basis set. Experimental spectrum is overlaid for comparison. a. u. = arbitrary units.

Selected MOs for TpCu-NB calculated using TD-DFT M06/def2-tzvpp

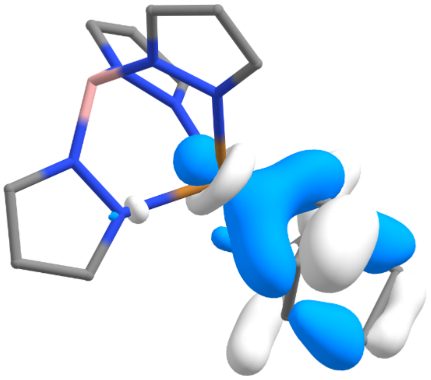

HOMO-11 - MO 85

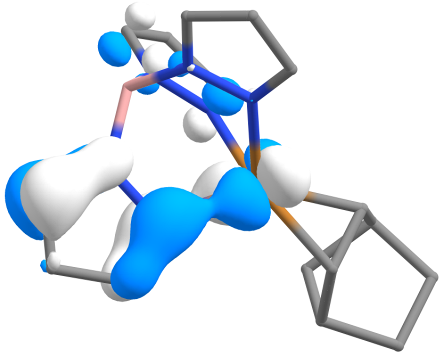

HOMO-10 - MO 86

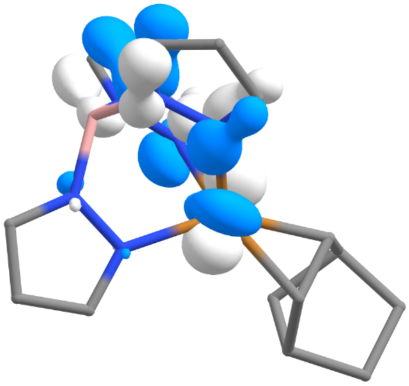

HOMO-9 - MO 87

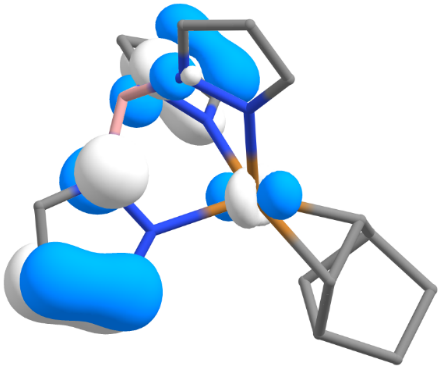

HOMO-8 - MO 88

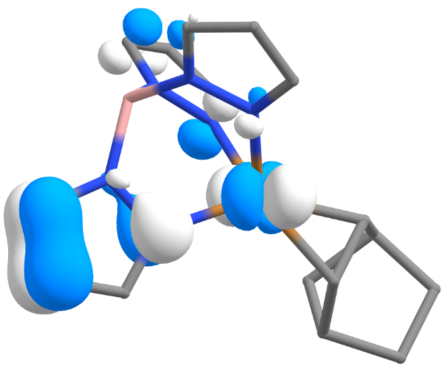

HOMO-4 - MO 92

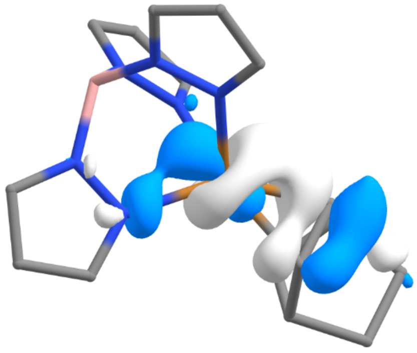

HOMO-2 - MO 94

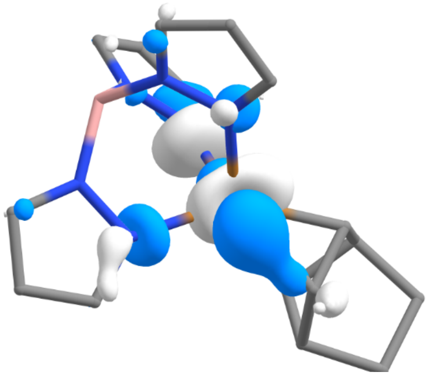

HOMO-1 - MO 95

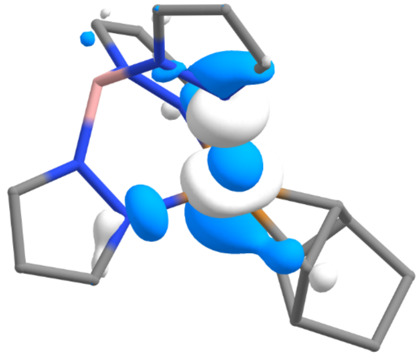

HOMO - MO 96

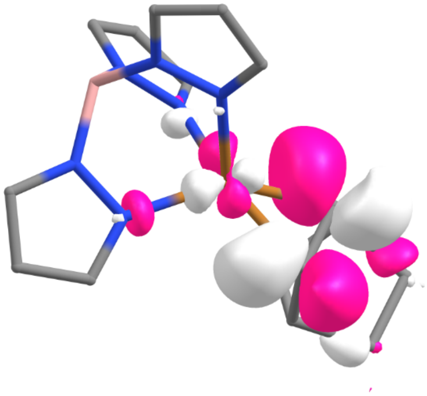

LUMO - MO 97

Excited State 1: 4.3085 eV,
287.76 nm f=0.0061

92 -> 97	0.10970
95 -> 97	-0.36822
96 -> 97	0.57070

Excited State 2: 4.6721 eV,
265.37 nm f=0.0201

85 -> 97	-0.14949
87 -> 97	0.19205
89 -> 97	0.13043
91 -> 97	0.20779
94 -> 97	-0.27178
95 -> 97	0.44013
96 -> 97	0.30363

Excited State 3: 4.8174 eV
257.37 nm f=0.0261

85 -> 97	0.21325
87 -> 97	0.23662
89 -> 97	0.14724
91 -> 97	0.23208
94 -> 97	0.53159
95 -> 97	0.11827

Excited State 4: 4.8775 eV
254.20 nm f=0.0009 <S**2>=0.000

86 -> 97	0.29578
88 -> 97	-0.22759
92 -> 97	0.57348

Excited State 5: 5.4660 eV
226.83 nm f=0.0061

95 -> 98	0.49315
95 -> 99	-0.17448
96 -> 98	0.33660
96 -> 99	0.30884

Excited State 6: 5.4968 eV
225.56 nm f=0.0662

95 -> 98	0.49315
95 -> 99	-0.17448
96 -> 98	0.33660
96 -> 99	0.30884

Excited State 7: 5.5906 eV
221.77 nm f=0.0308

95 -> 98	-0.30867
95 -> 99	-0.26602
96 -> 98	-0.17092
96 -> 99	0.52944

Excited State 8: 5.7255 eV
216.55 nm f=0.1013

91 -> 97	-0.11879
95 -> 99	0.59017
96 -> 99	0.31573

Excited State 9: 5.8585 eV
211.63 nm f=0.3554

87 -> 97	0.13381
89 -> 97	0.10708
91 -> 97	0.37843
91 -> 99	-0.13496
92 -> 98	-0.21918
94 -> 97	-0.15685
94 -> 98	0.13483
94 ->100	0.12360
95 -> 97	-0.24461
95 -> 98	0.12842
96 -> 97	-0.15129
96 -> 98	-0.15939
96 ->100	-0.17250

Excited State 10: 5.8958 eV,
211.63 nm f=0.3554

93 -> 97	0.69823

Geometry optimizations for TpCu-MA were done starting from the crystallographic atomic coordinates obtained from the solved X-ray crystal structure. Ground state geometry optimizations and frequency calculations were performed using the M11 density functional[16,17] with Ahlrichs' def2-tzvpp basis set[18] using the Gaussian09 software package.

TpCu-MA and its excited states were analyzed via Tam-Dancoff approximation density functional theory (TDA-DFT) calculations (first ten excited states). This provided a theoretical electronic absorption spectrum that most closely resembled the experimental spectra (see **Supplementary Figure 24**). Calculations using the same TD-DFT M06 density functional that was identified as optimal for TpCu-NB, did not result in a closely matched theoretical spectrum as compared to the experimental (**Supplementary Figure 24**).

From this, an absorption band at 341.9 nm, which corresponds to an excitation from the HOMO - 7 (MO 88) to the LUMO (MO 96) could be computed. Further absorption bands were detected at 308.2 nm, and 224 nm. While there are various HOMOs contributing to the possible transitions to the excited state each is primarily metal based (see images below). Therefore, the observed excitation can be interpreted as a metal to ligand charge transfer (MLCT). The HOMO shows significant contributions from the Cu d_{z^2} orbital, and the LUMO shows contribution from the Cu d_{xy} orbital and maleic anhydride π^* orbital.

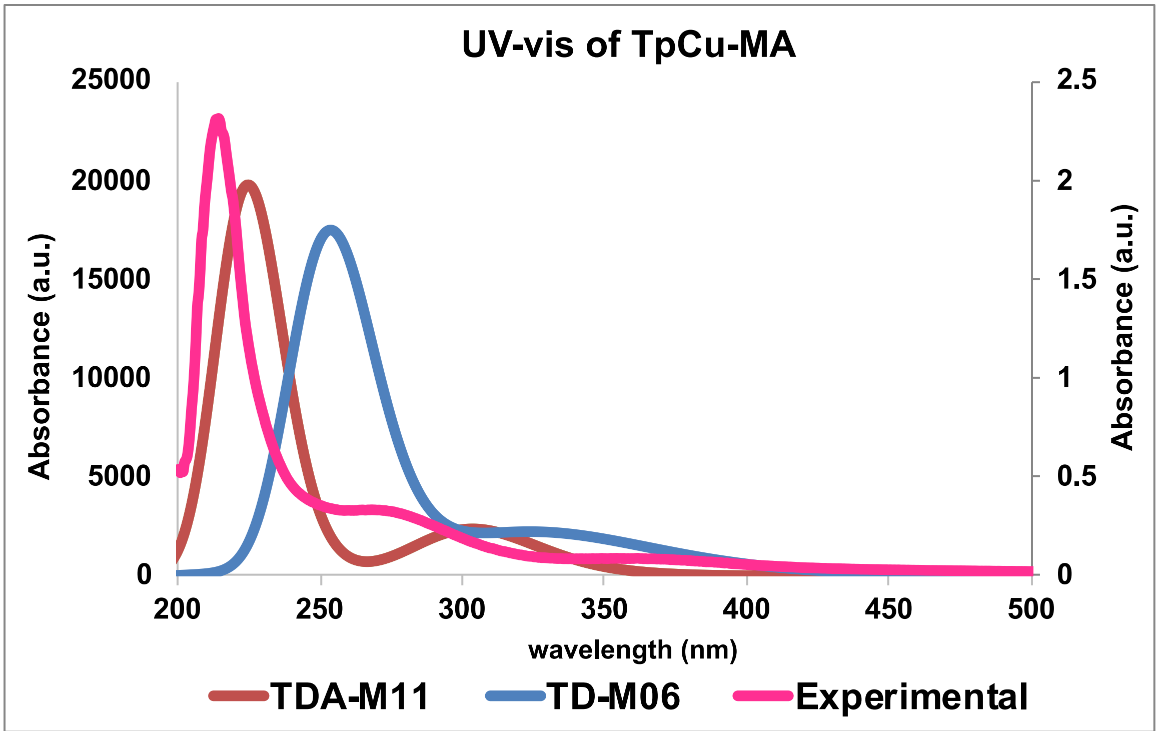

Supplementary Figure 24. Theoretical electronic absorption spectra of TpCu-MA using TDA-DFT M11 and TD-DFT M06 functionals with the def2-tzvpp basis set. Experimental spectrum is overlaid for comparison. a. u. = arbitrary units.

Selected MOs for TpCu-MA calculated using TDA-DFT M11/def2-tzvpp

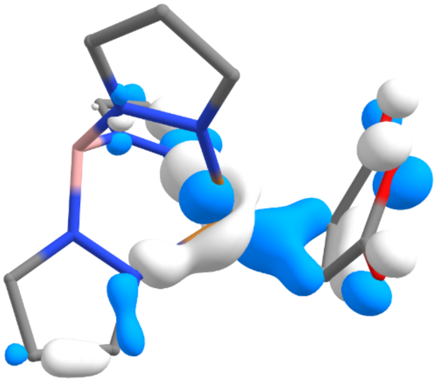

HOMO-16 - MO 79

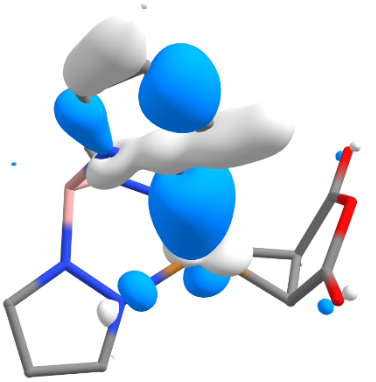

HOMO-15 - MO 80

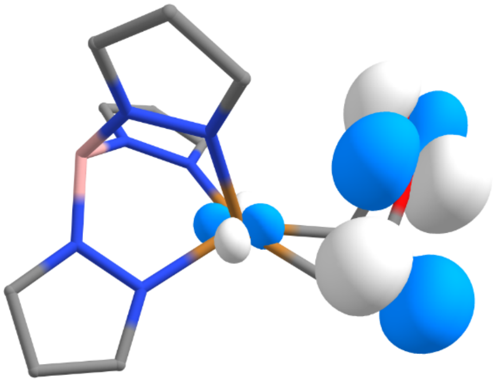

HOMO-14 - MO 81

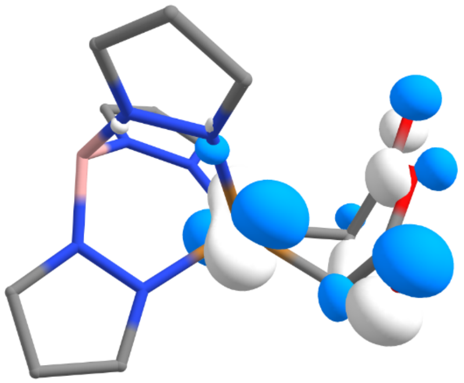

HOMO-13 - MO 82

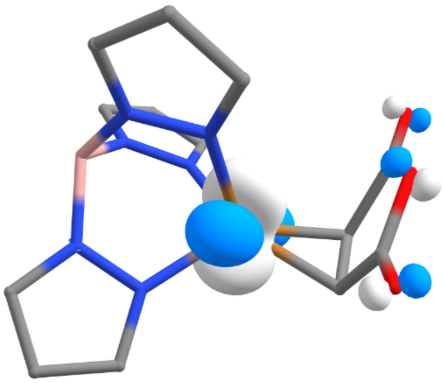

HOMO-12 - MO 83

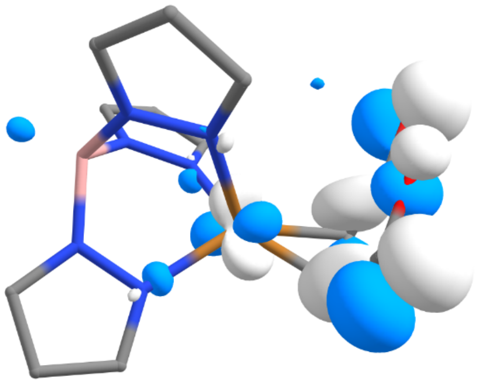

HOMO-10 - MO 85

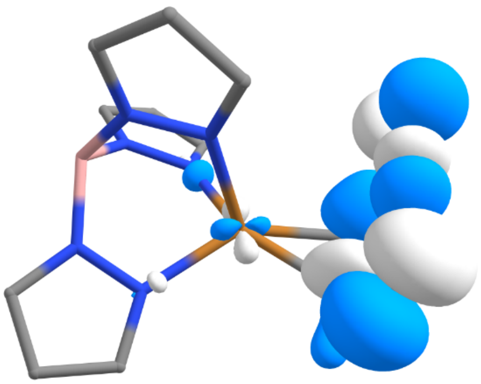

HOMO-9 - MO 86

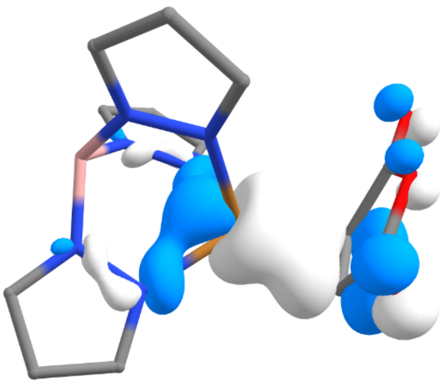

HOMO-8 - MO 87

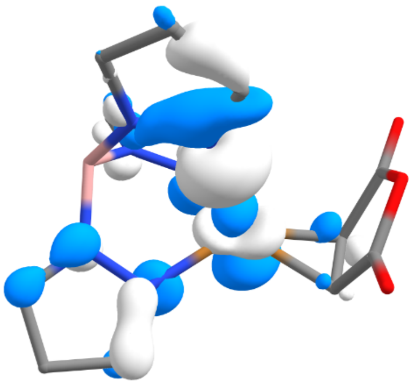

HOMO-7 - MO 88

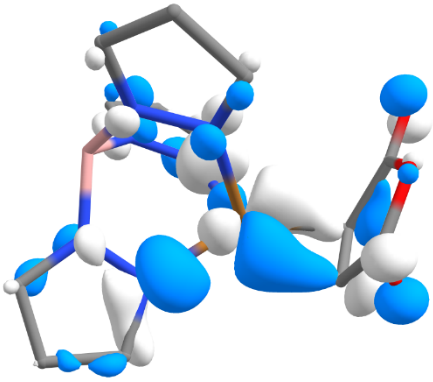

HOMO-6 - MO 89

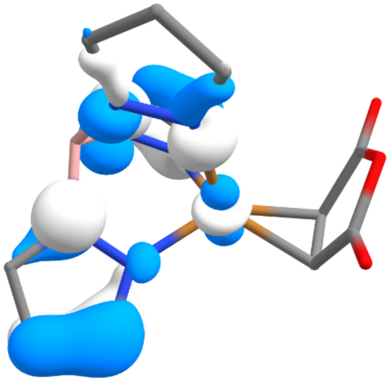

HOMO-5 - MO 90

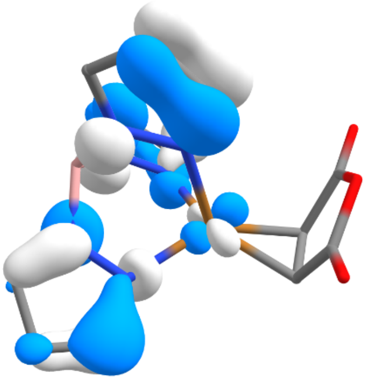

HOMO-4 - MO 91

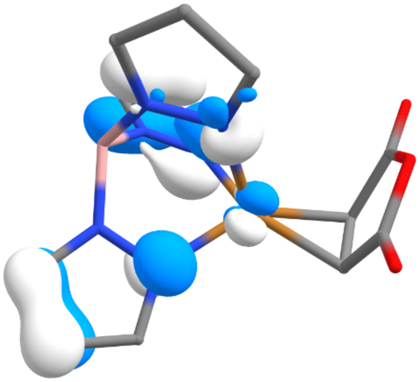

HOMO-3 - MO 92

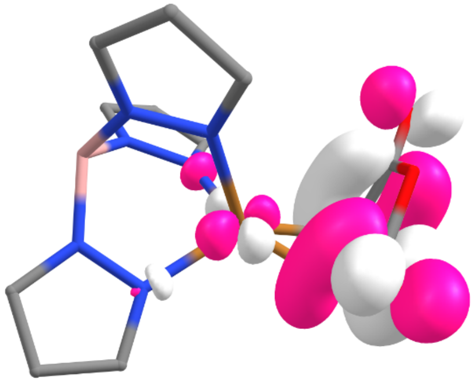

LUMO - MO 96

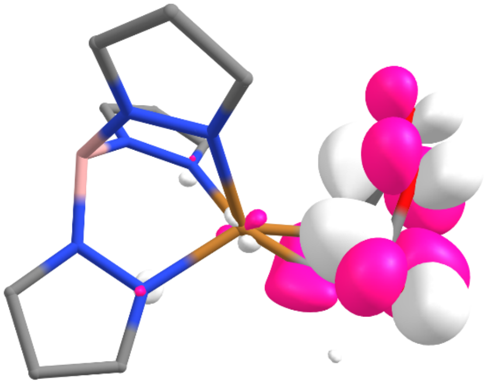

LUMO+5 - MO 101

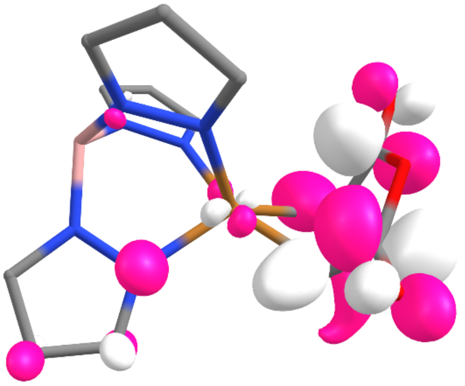

LUMO+8 - MO 104

Excited State 1: 3.6255 eV,
341.97 nm f=0.0016

79 -> 96	-0.10830
80 -> 96	0.19795
88 -> 96	0.47863
90 -> 96	0.30710
92 -> 96	0.24187

Excited State 2: 4.0229 eV,
308.20 nm f=0.0280

79 -> 96	0.22606
80 -> 96	0.11565
81 -> 96	0.12023
82 -> 96	-0.12166
87 -> 96	0.55614
88 -> 96	0.12809

Excited State 3: 4.1218 eV,
300.80 nm f=0.0298
83 -> 96 0.48127
83 ->104 -0.10970
86 -> 96 -0.14972
89 -> 96 -0.13427
91 -> 96 0.20555
93 -> 96 -0.19785
94 -> 96 -0.27229

Excited State 4: 4.2248 eV,
293.47 nm f=0.0003
82 -> 96 0.42312
82 ->104 -0.10128
85 -> 96 -0.31625
86 -> 96 0.13088
87 -> 96 0.17622
88 -> 96 -0.16593
92 -> 96 0.27629

Excited State 5: 4.5501 eV,
272.49 nm f=0.0045
83 -> 96 0.18695
85 ->101 0.12456
86 -> 96 0.57533
86 ->104 0.11288
89 -> 96 0.19876

Excited State 6: 5.2446 eV,
236.40 nm f=0.0042
79 -> 96 0.19264
82 -> 96 0.33533
85 -> 96 0.46184
85 ->104 0.10529
86 ->101 0.23047

Excited State 7: 5.3037 eV,
233.77 nm f=0.0020
93 -> 96 0.12953
94 -> 96 -0.19401
95 -> 96 0.65438

Excited State 8: 5.3307 eV,
232.59 nm f=0.0248
83 -> 96 0.14102
91 -> 96 -0.11250
92 -> 96 -0.10250
93 -> 96 -0.41000
94 -> 96 0.45831
95 -> 96 0.24788

Excited State 9: 5.5199 eV,
224.61 nm f=0.4618
83 -> 96 0.22462
86 -> 96 -0.22512
89 -> 96 0.44330
91 -> 96 -0.31997
93 -> 96 0.15464

Excited State 10: 5.9332 eV,
208.97 nm f=0.0060
83 -> 96 0.24290
89 -> 96 -0.15101
91 -> 96 0.14825
92 -> 96 0.16978
93 -> 96 0.44283
94 -> 96 0.36904

While the TDA-DFT theoretical electronic absorption spectrum for TpCu-NB generated using the M11 functional (see **Supplementary Figure 25**) was reasonably well-matched to the experimental spectrum, we've elected to primarily report the results using the M06 functional as it more closely matched the experimental λ_{max} by 17 nm. From this an absorption band at 341.9 nm, which corresponds to an excitation from the HOMO - 7 (MO 88) to the LUMO (MO 96) could be computed. Further absorption bands were detected at 308.2 nm, and 224 nm. While there are various HOMOs contributing to the possible transitions to the excited state each is primarily metal based (see images below). This analysis provides analogous conclusions to those obtained using the TD-DFT M06 functional, albeit with a change in sign (see below images), while TD-DFT M06 analysis resulted in a stronger theoretical spectrum match to experimental.

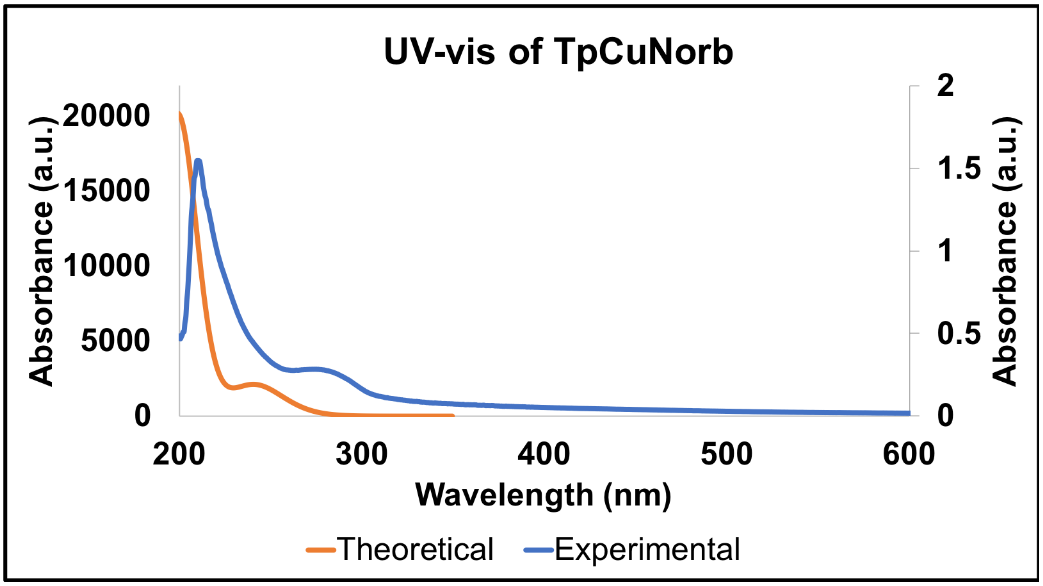

Supplementary Figure 25. Theoretical (TDA-DFT M11/def2-tzvpp) and experimental electronic absorption spectra of TpCu-NB. a. u. = arbitrary units.

Selected MOs for TpCu-NB calculated using TDA-DFT M11/def2-tzvpp

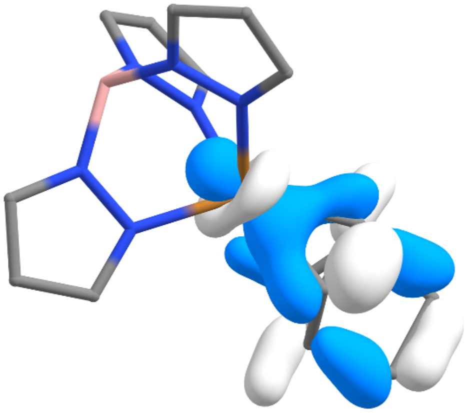

HOMO-11 - MO 85

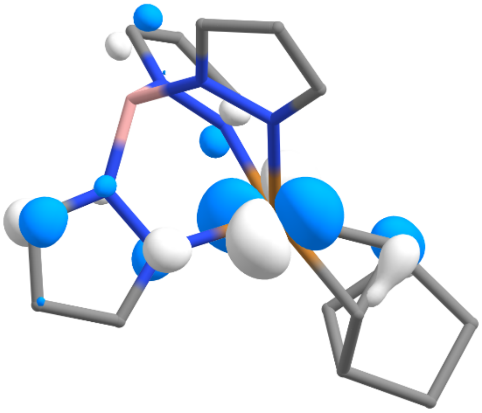

HOMO-10 - MO 86

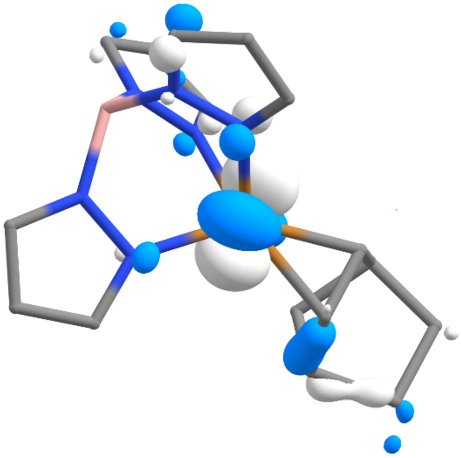

HOMO-9 - MO 87

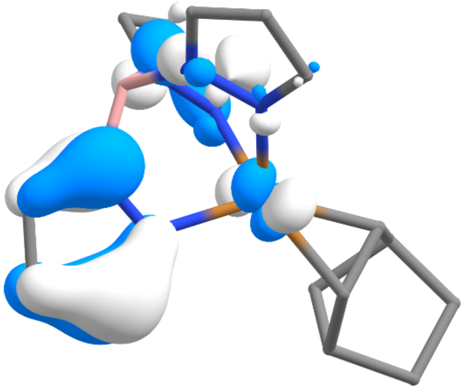

HOMO-8 - MO 88

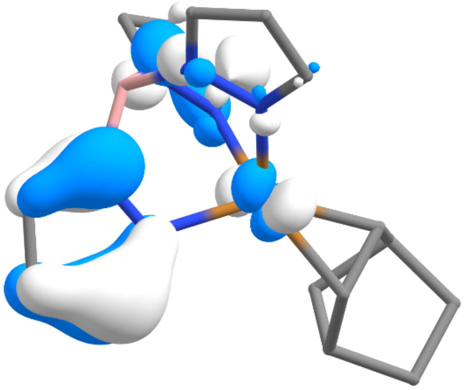

HOMO-6 - MO 90

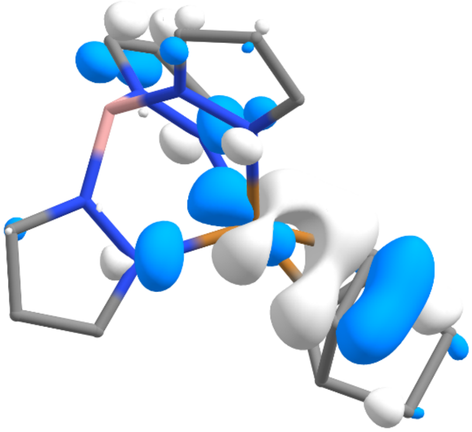

HOMO-3 - MO 93

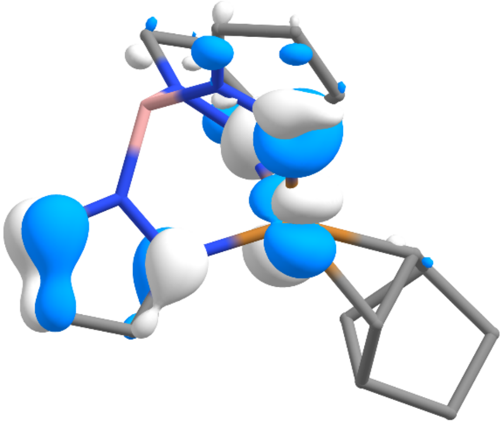

HOMO-2 - MO 94

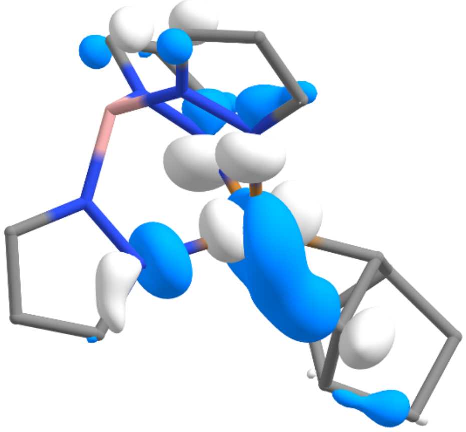

HOMO - MO 96

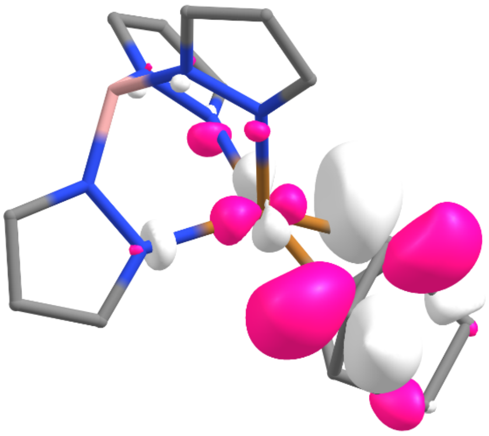

LUMO+1 - MO 98

Excited State 1: 4.8525 eV,
255.51 nm f=0.0055

80 ->	98	-0.18415
88 ->	98	-0.20707
90 ->	98	-0.32933
94 ->	98	0.49866
96 ->	98	-0.10063

Excited State 2: 5.0822 eV,
243.96 nm f=0.0298

85 ->	98	0.33925
86 ->	98	0.11018
90 ->	98	0.11157
93 ->	98	0.42799
96 ->	98	-0.32348

Excited State 3: 5.2697 eV,
235.28 nm f=0.0189

86 ->	98	0.24132
87 ->	98	0.44226
93 ->	98	0.21481
96 ->	98	0.34039

Excited State 4: 5.3399 eV,
232.18 nm f=0.0029

84 ->	98	-0.11371
85 ->	98	-0.11158
86 ->	98	0.46131
87 ->	98	-0.19323
88 ->	98	-0.21389
90 ->	98	-0.26130
94 ->	98	-0.24180

Excited State 5: 6.1830 eV,
200.52 nm f=0.4611

87 ->	98	0.22185
89 ->	98	0.23278
91 ->	98	0.35237
92 ->	98	-0.14788
94 ->	99	0.18026
96 ->	98	-0.32325

Excited State 6: 6.5356 eV,
189.70 nm f=0.0050

90 ->	100	0.19282
91 ->	99	-0.10624
94 ->	99	0.11863
94 ->	100	-0.28128
96 ->	99	0.48280

Excited State 7: 6.6926 eV, 185.26 nm f=0.0286
90 -> 104 0.18349
91 -> 99 -0.11900
93 -> 100 0.10890
94 -> 99 -0.23895
94 -> 100 -0.20069
95 -> 99 0.31710
96 -> 97 0.17555
96 -> 99 -0.11861
96 -> 100 0.27251

Excited State 8: 6.7216 eV, 184.46 nm f=0.0096
91 -> 104 0.17563
93 -> 99 0.13494
94 -> 99 -0.13370
94 -> 100 0.29948
95 -> 98 0.15715
95 -> 100 0.34976
96 -> 97 0.12104
96 -> 99 0.15263
96 -> 100 0.10690

Excited State 9: 6.8071 eV, 182.14 nm f=0.1081
88 -> 104 -0.12511
90 -> 99 -0.19764
90 -> 100 -0.18753
91 -> 99 -0.13794
91 -> 100 0.10152
92 -> 99 0.17293
94 -> 99 0.15543
94 -> 100 0.12556
94 -> 104 0.18399
95 -> 99 0.31418
96 -> 99 0.12056
96 -> 100 -0.21366

Excited State 10: 6.8250 eV, 181.66 nm f=0.1592
89 -> 104 -0.12170
90 -> 99 -0.23099
90 -> 100 0.16674
91 -> 99 0.14895
91 -> 100 0.13487
94 -> 100 -0.21593
95 -> 98 0.10523
95 -> 100 0.28672
96 -> 99 -0.20542
96 -> 100 -0.17522
96 -> 104 0.17499

```
%mem=20GB
%nprocshared=8
%chk=TpCuNorb-M11.chk
# opt freq m11/def2tzvpp scrf=(solvent=diethylether) integral=ultrafinegrid pop=full

Title Card Required

0 1
xyz
```

Supplementary Figure 26. Example input file for geometry optimization and frequency calculations.

```
%mem=20GB
%nprocshared=8
%chk=TpCuNorb-TDA-M11.chk
# tda=nstates=10 m11/def2tzvpp scrf=(solvent=diethylether) integral=ultrafinegrid

Title Card Required

0 1
xyz
```

Supplementary Figure 27. Example input file for TDA-DFT calculations.

J. Crystallographic Structure Determination

Single crystal X-ray structure determinations were carried out at low temperature on Bruker Kappa diffractometers equipped with a Mo sealed tube or rotating anode or Cu rotating anode radiation source and a Bruker APEX-II, or Proteum Pt135 detector. All structures were solved via intrinsic methods with SHELXT and refined by full-matrix least squares procedures using SHELXL within the Olex2 small-molecule solution, refinement and analysis software package.

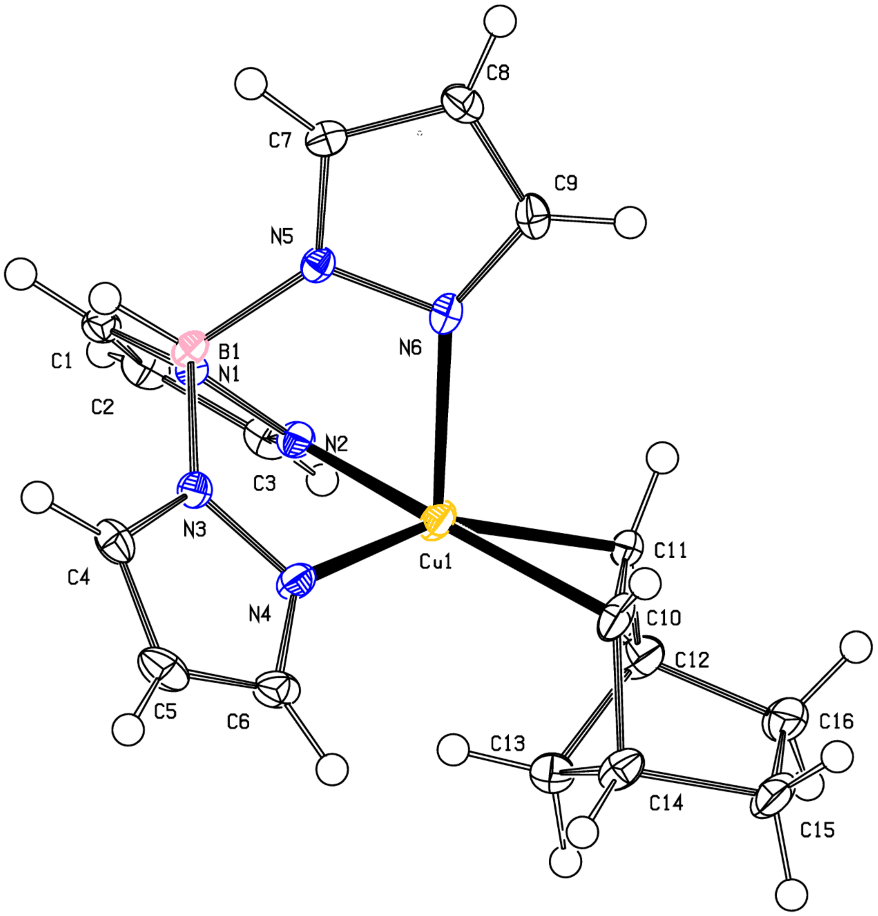

Supplementary Figure 28. Molecular structure of TpCu-NB**.**

Selected Bond Distances (Å)		Selected Bond Angles (°)	
Cu-N2	2.010(2)	Cu–N2–N1	115.4(1)
Cu-N4	1.999(2)	Cu–N4–N3	116.8(1)
Cu-N6	2.274(2)	Cu–N6–N5	111.6(1)
Cu-C10	2.016(2)	N4–Cu–C10	108.97(8)
Cu-C11	2.029(2)	N2–Cu–C11	105.57(8)
N1-B	1.543(3)	N6–Cu–C10	106.91(8)
N3-B	1.549(3)	Cu–C10–C14	116.4
N5-B	1.545(3)	Cu–C10–H10	105.7
C10-C11	1.381(3)	H10–C10–C11	126.5
		H11–C11–C10	125.7

Supplementary Table 5. Selected bond distances and angles for TpCu-NB**.**

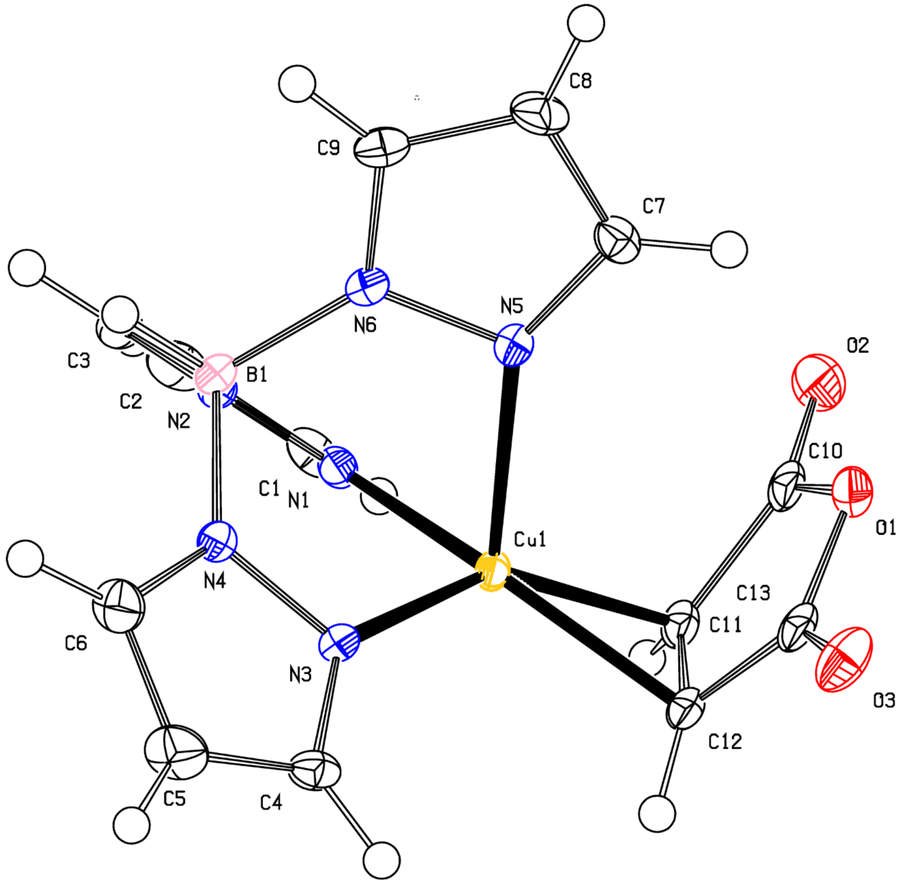

Supplementary Figure 29. Molecular structure of TpCu-MA**.**

**Selected Bond Distances (Å)**		**Selected Bond Angles (°)**	
Cu-N1	1.967(2)	Cu–N1–N2	116.0(1)
Cu-N3	1.977(2)	Cu–N3–N4	115.3(1)
Cu-N5	2.218(2)	Cu–N5–N6	110.4(1)
Cu-C11	2.000(2)	N1–Cu–C11	106.66(7)
Cu-C12	2.006(2)	N3–Cu–C12	109.46(7)
N2-B	1.543(3)	N5–Cu–C12	114.91(7)
N4-B	1.551(3)	Cu–C11–H11	121.2
N6-B	1.542(3)	Cu–C12–H12	121
C11-C12	1.390(3)	C12–C11–H11	121.2
		C11–C12–H12	121

Supplementary Table 6. Selected bond distances and angles for TpCu-MA**.**

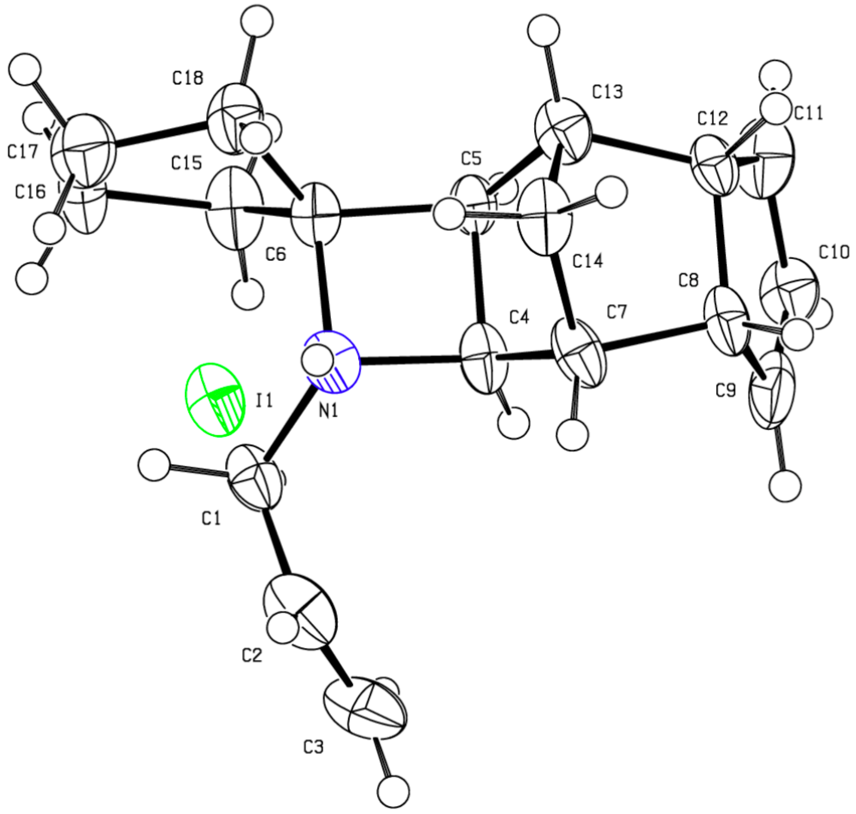

Supplementary Figure 30. Molecular structure of **22** as an HI salt. Azetidine was reacted with excess methyl iodide, upon standing and slow evaporation from THF colorless crystals were obtained.

**Selected Bond Distances (Å)**		**Selected Bond Angles (°)**	
N1-C1	1.472(1)	N1-C4-C5	91.0(7)
N1-C4	1.516(1)	C4-N1-C6	89.7(7)
N1-C6	1.544(1)	C1-N1-C4	115.6
N1-H	1.000(0)	C4-C5-C6	88.6(7)
C4-C5	1.548(1)	C5-C6-N1	90.2(7)
C5-C6	1.542(1)	N1-C4-H4	114.9
		C4-C5-H5	113.6

Supplementary Table 7. Selected bond distances and angles for **22** HI salt.

K. ^{1}H, ^{13}C, and 2-D NMR Spectra

a

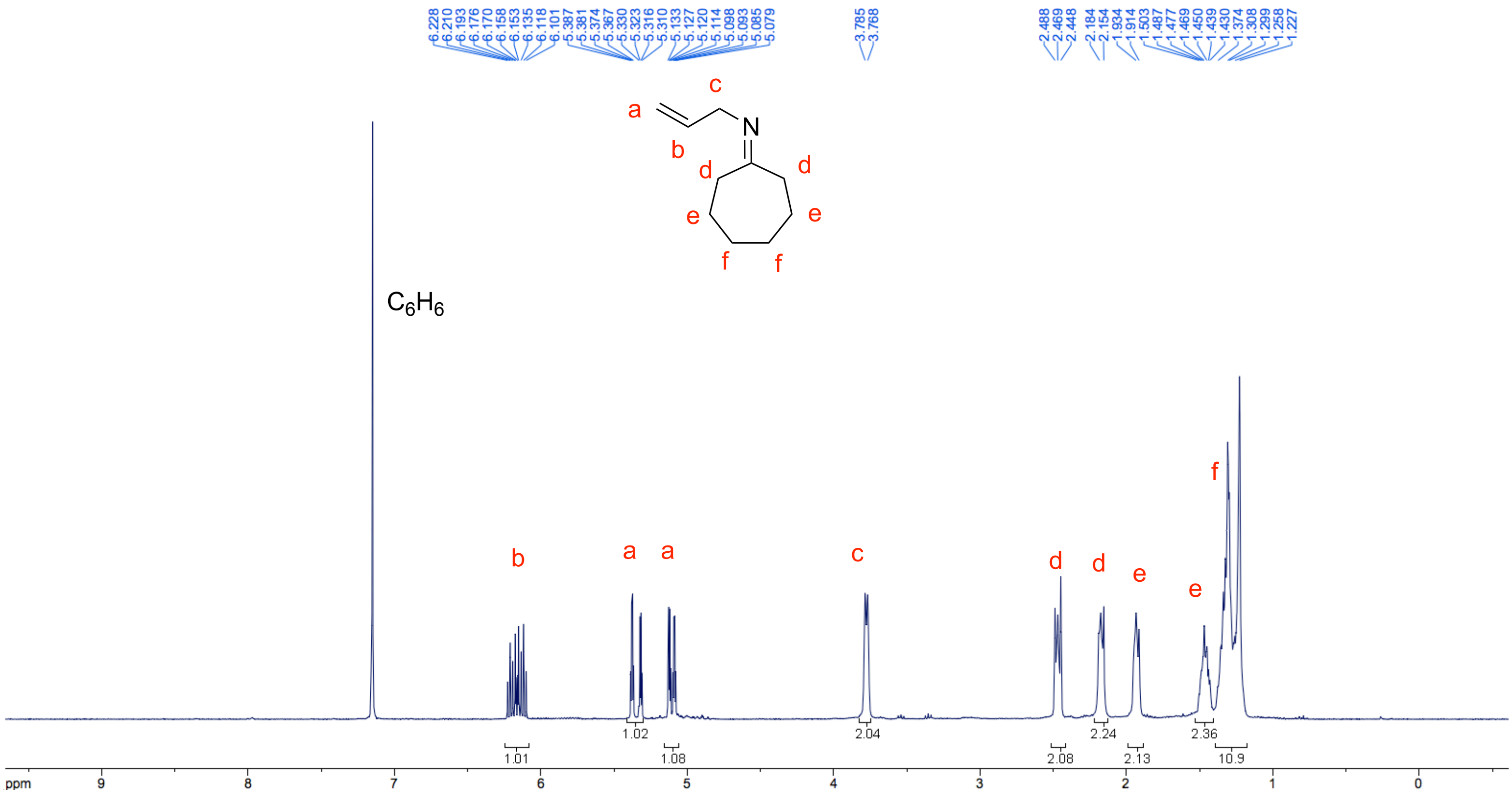

b

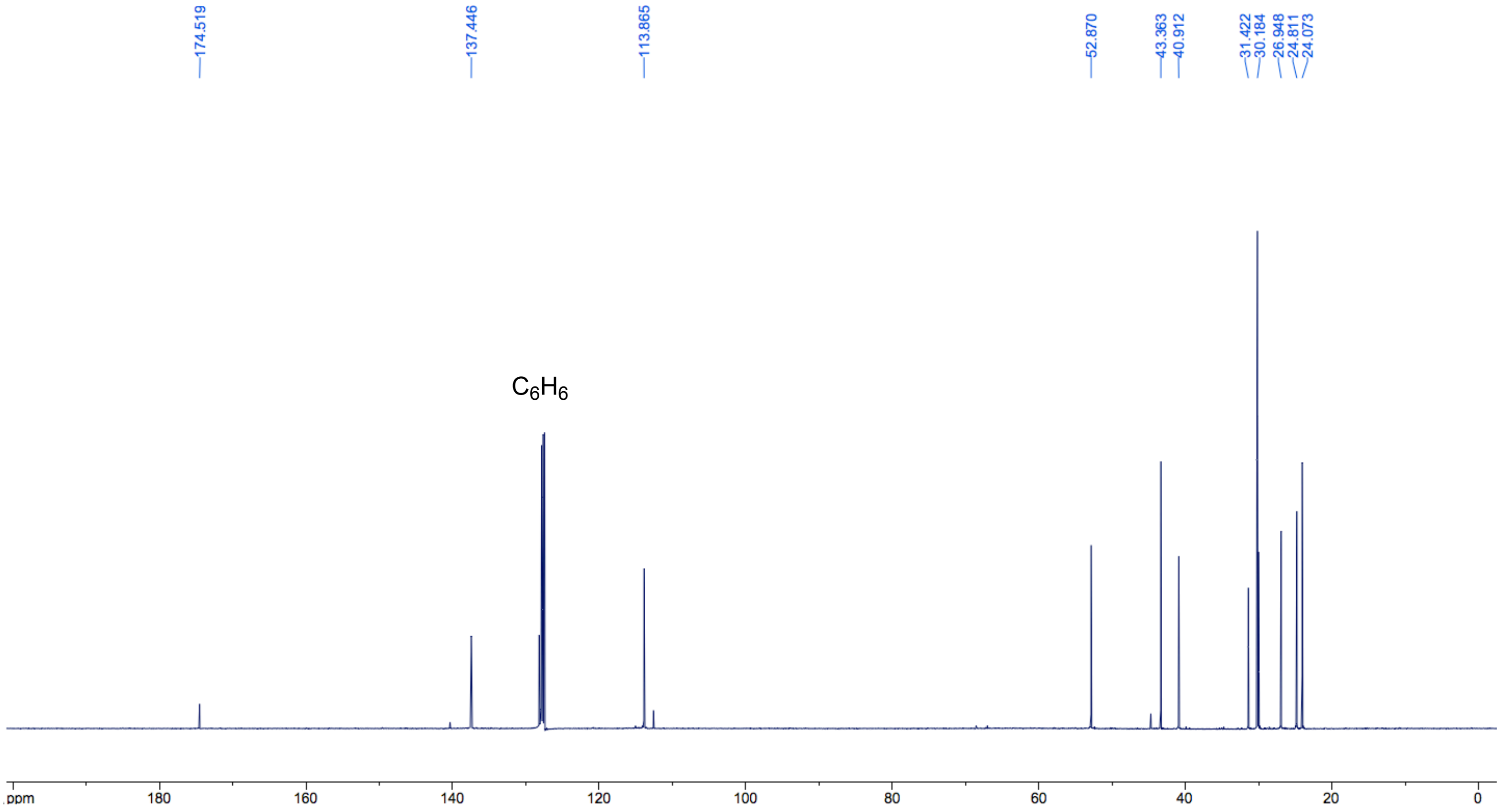

Supplementary Figure 30. NMR spectra of *N*-allylcycloheptylideneamine. **a** ^{1}H NMR (300 MHz, C_6D_6). **b** ^{13}C NMR (126 MHz, C_6D_6).

a

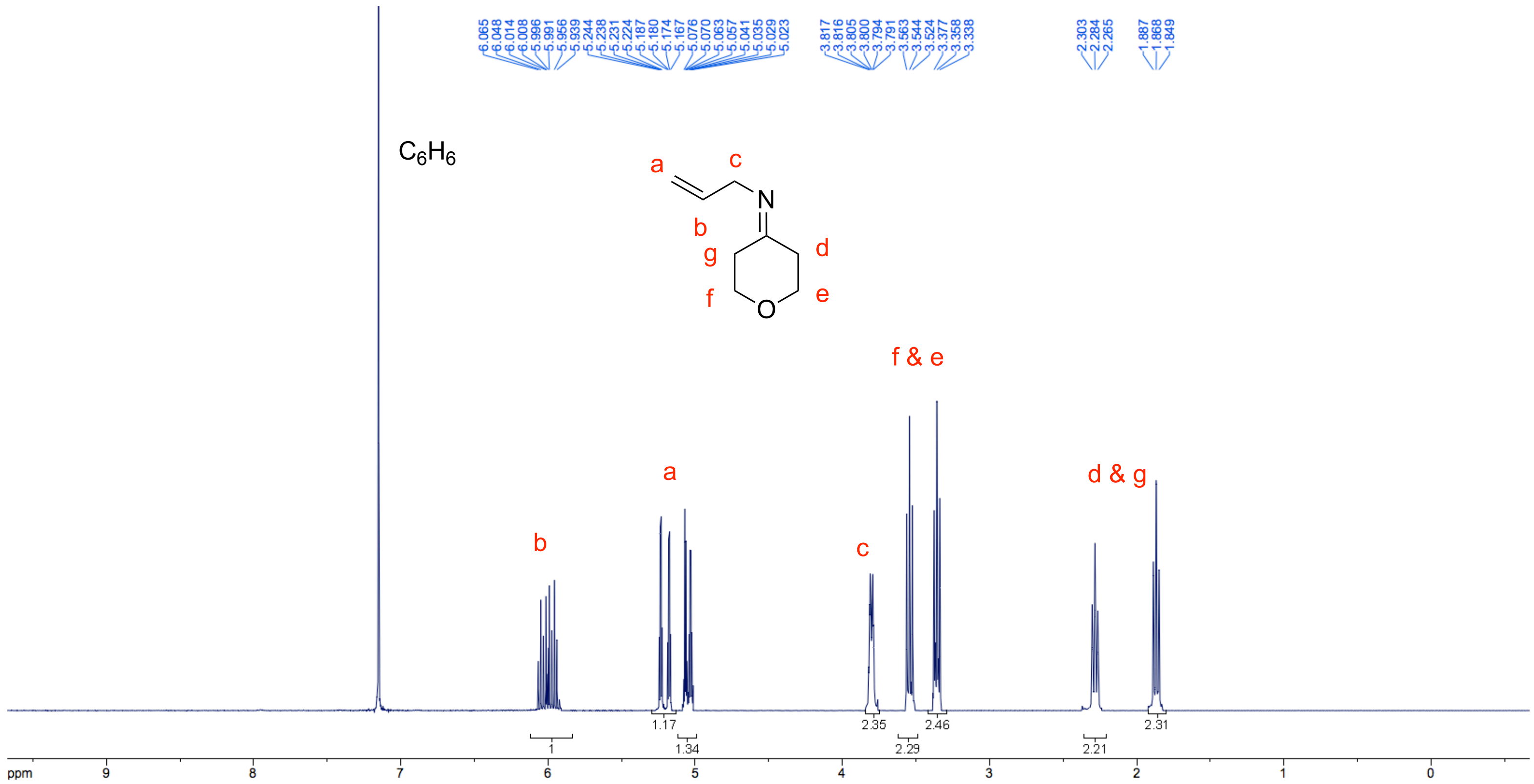

b

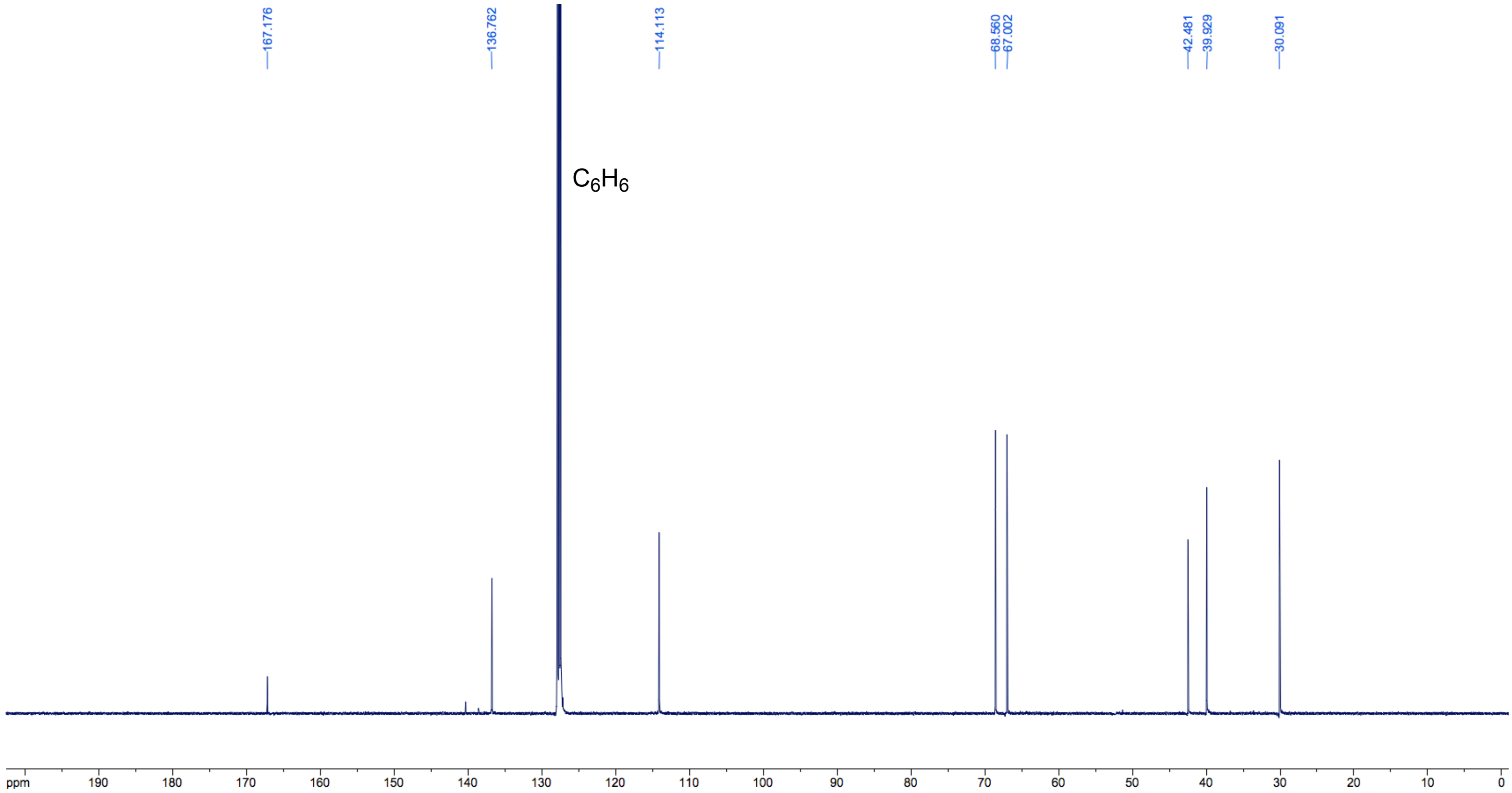

Supplementary Figure 31. NMR spectra of 4-*N*-allyl-tetrahydro-4H-pyran. **a** ^{1}H NMR (300 MHz, C_6D_6). **b** ^{13}C NMR (126 MHz, C_6D_6).

a

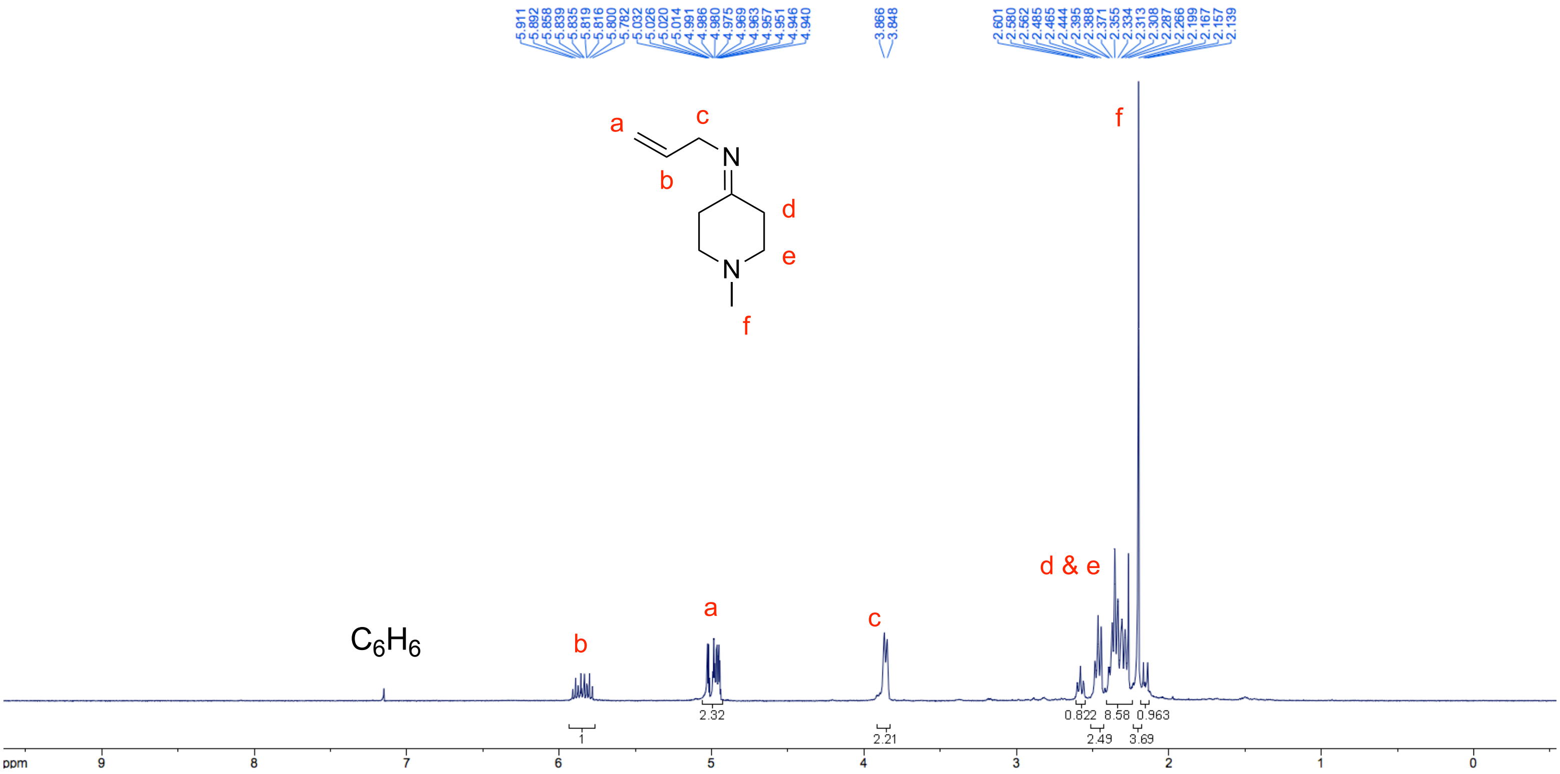

b

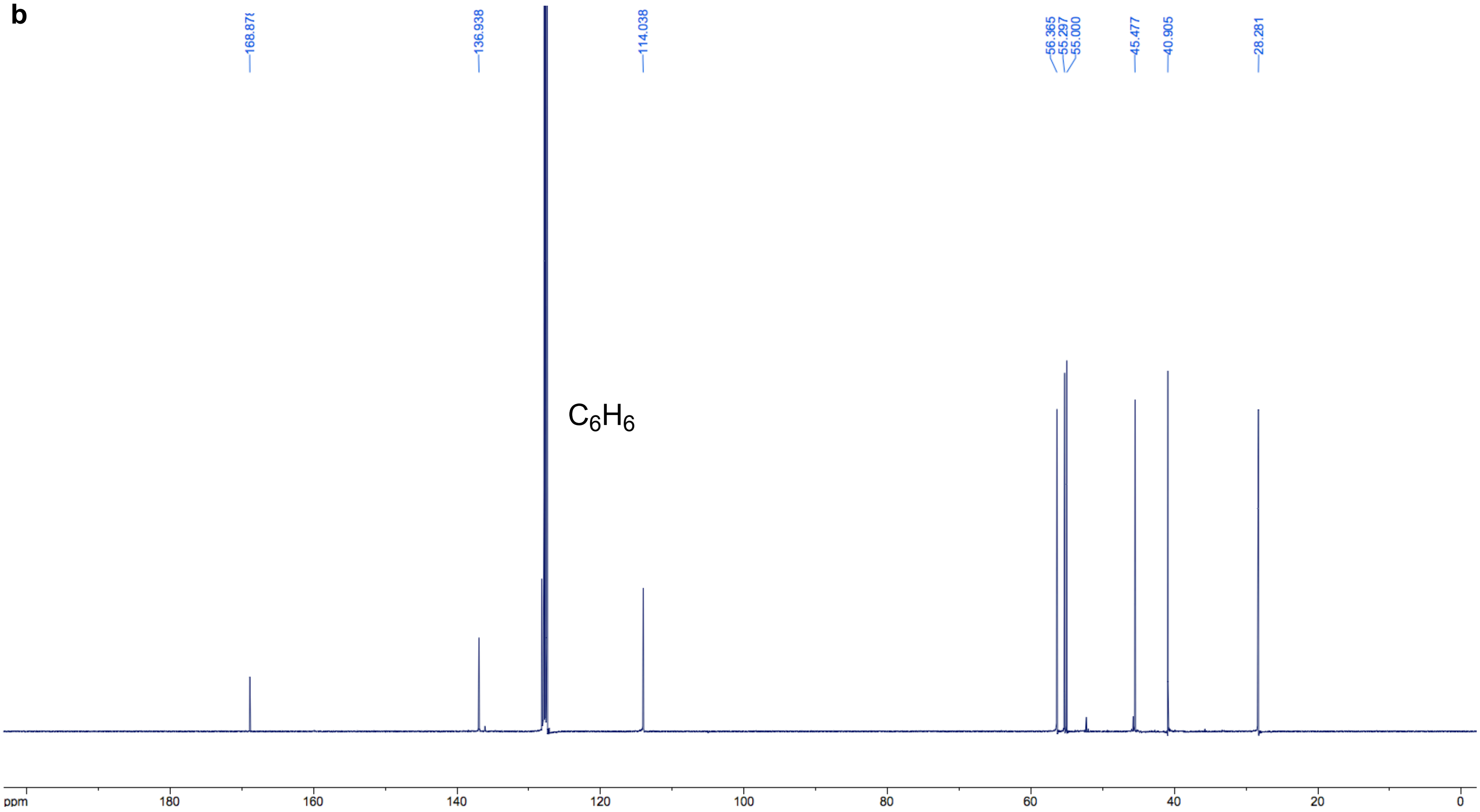

Supplementary Figure 32. NMR spectra of *N*-allyl-1-methyl-4-piperidinylidene. **a** ^{1}H NMR (300 MHz, C_6D_6). **b** ^{13}C NMR (126 MHz, C_6D_6).

a

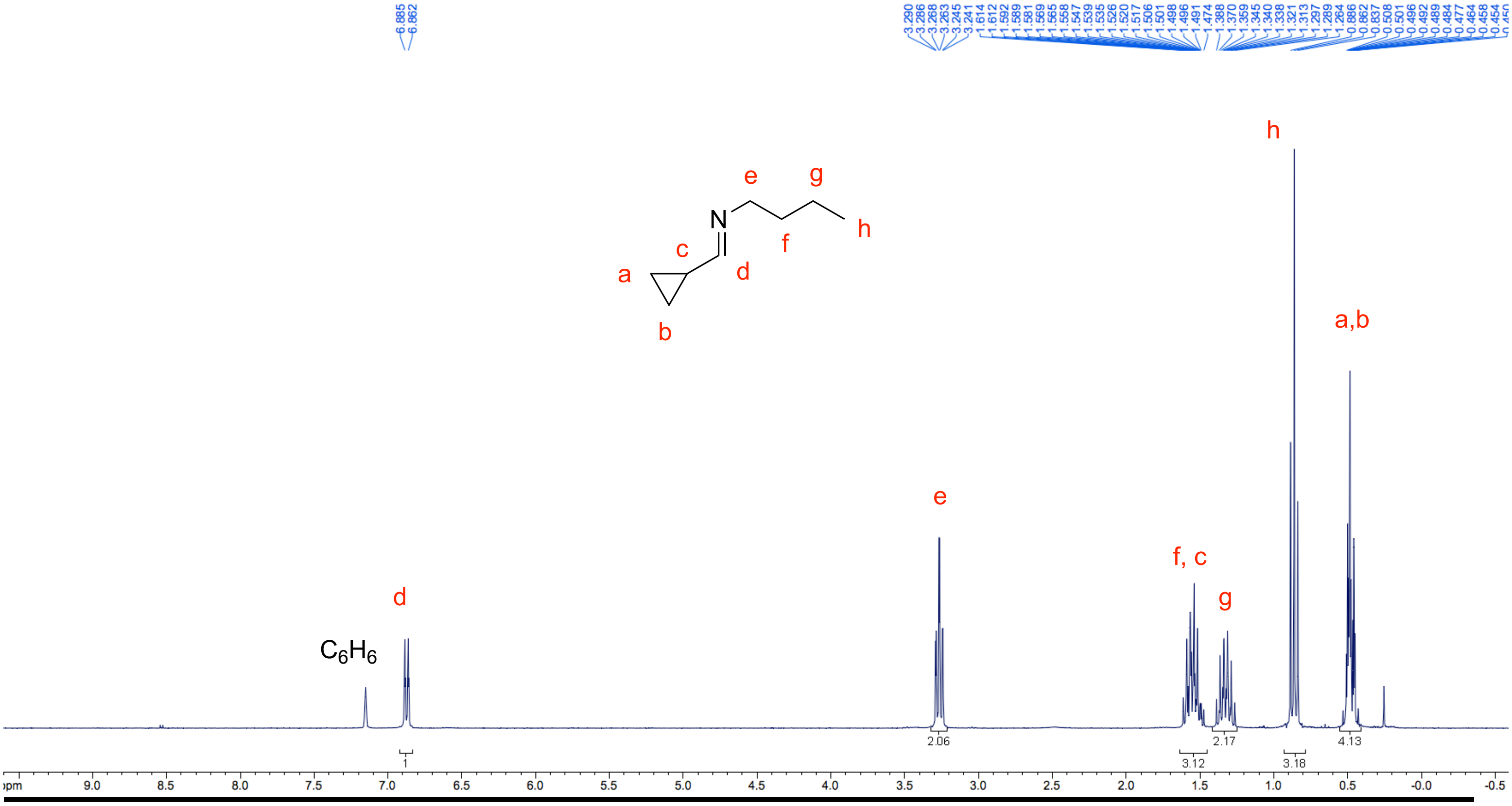

b

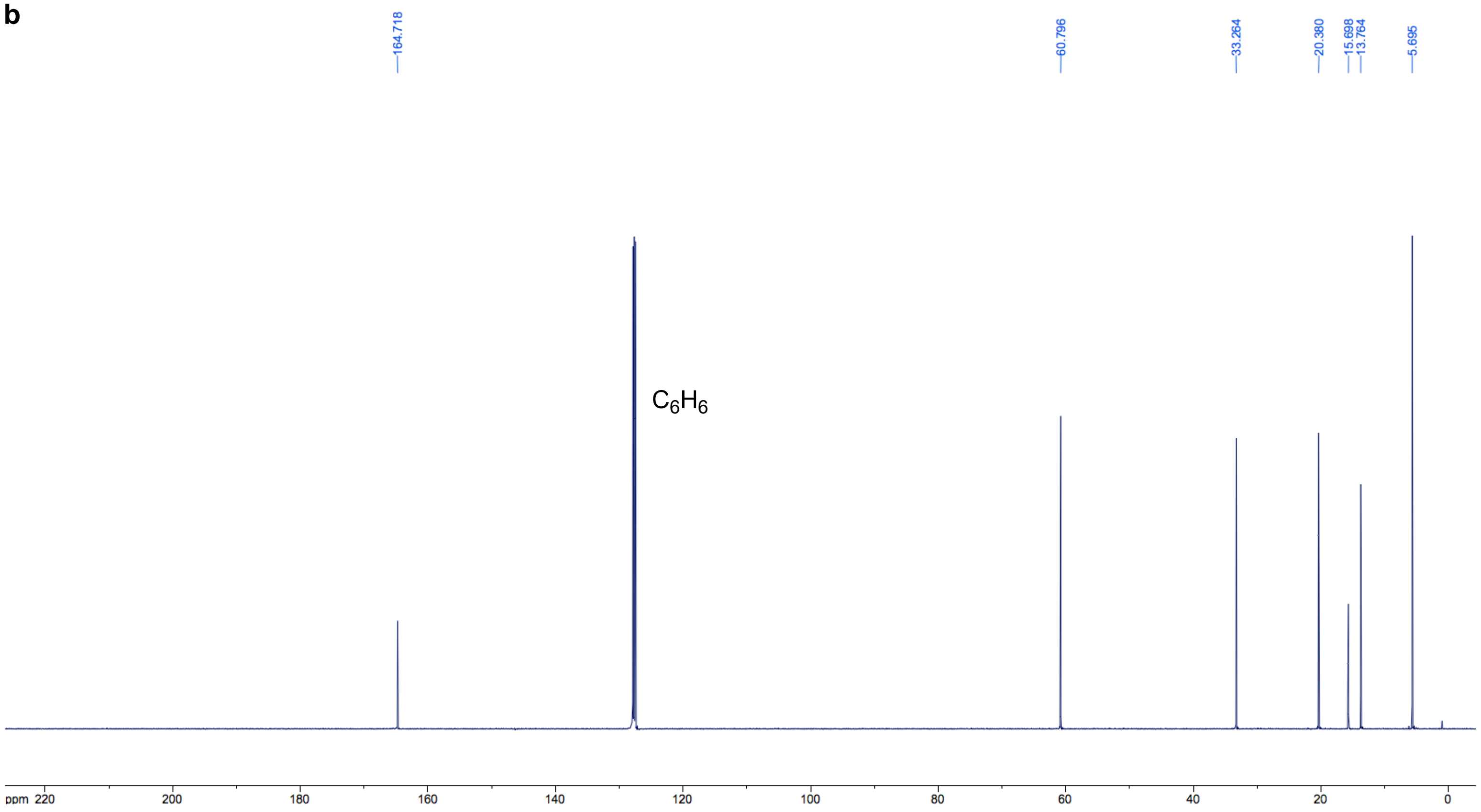

Supplementary Figure 33. NMR spectra of *N*-cyclopropylidenebutylamine . **a** ^{1}H NMR (300 MHz, C_6D_6). **b** ^{13}C NMR (126 MHz, C_6D_6).

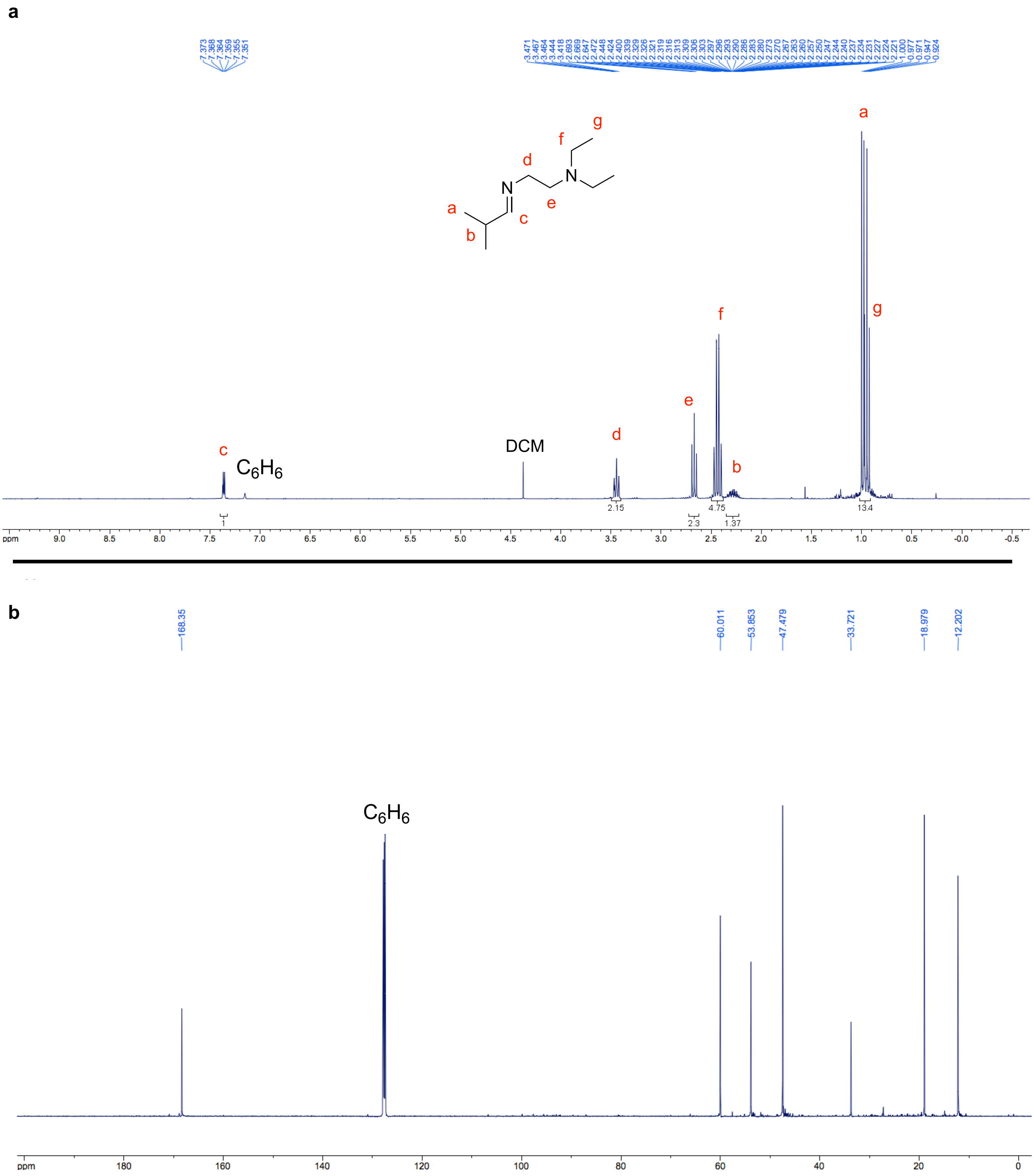

Supplementary Figure 34. NMR spectra of *N'*-isobutylidene-*N*,*N*-diethyl-1,2-ethanediamine. **a** ^{1}H NMR (300 MHz, C_6D_6). **b** ^{13}C NMR (126 MHz, C_6D_6).

a

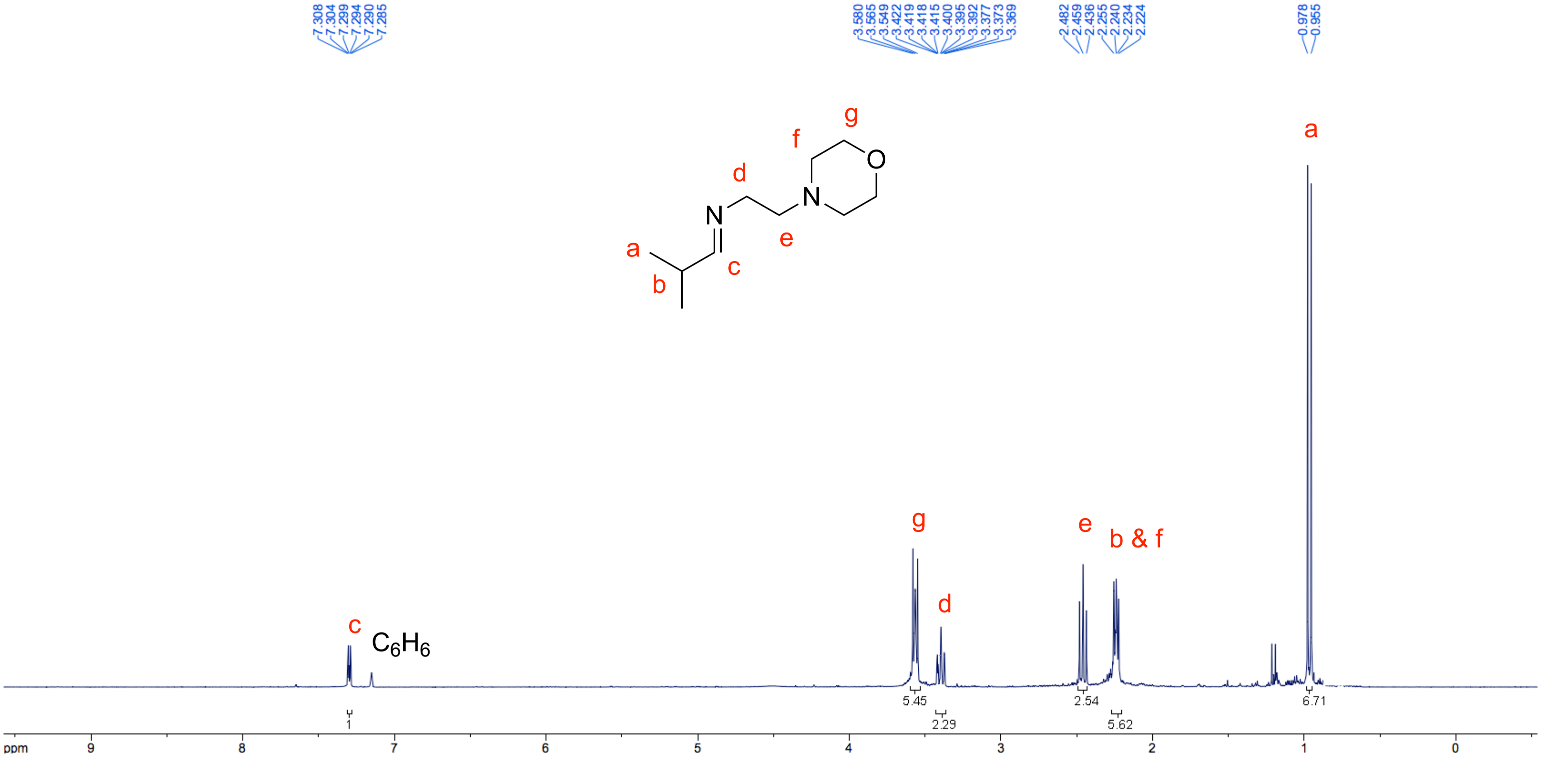

b

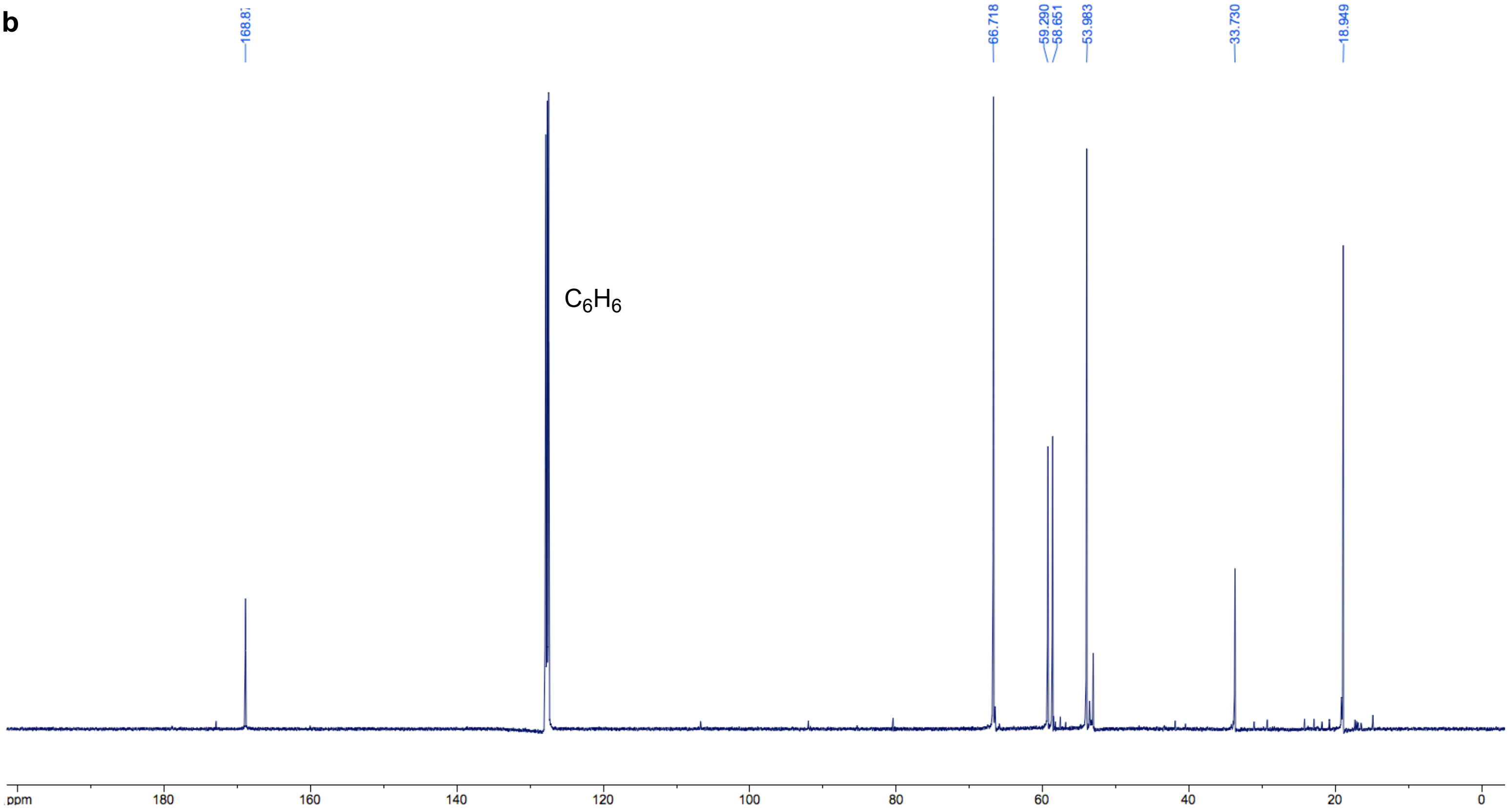

Supplementary Figure 35. NMR spectra of *N*-isobutylidene-1,2-ethanemorpholine. **a** ^{1}H NMR (300 MHz, C_6D_6). **b** ^{13}C NMR (126 MHz, C_6D_6).

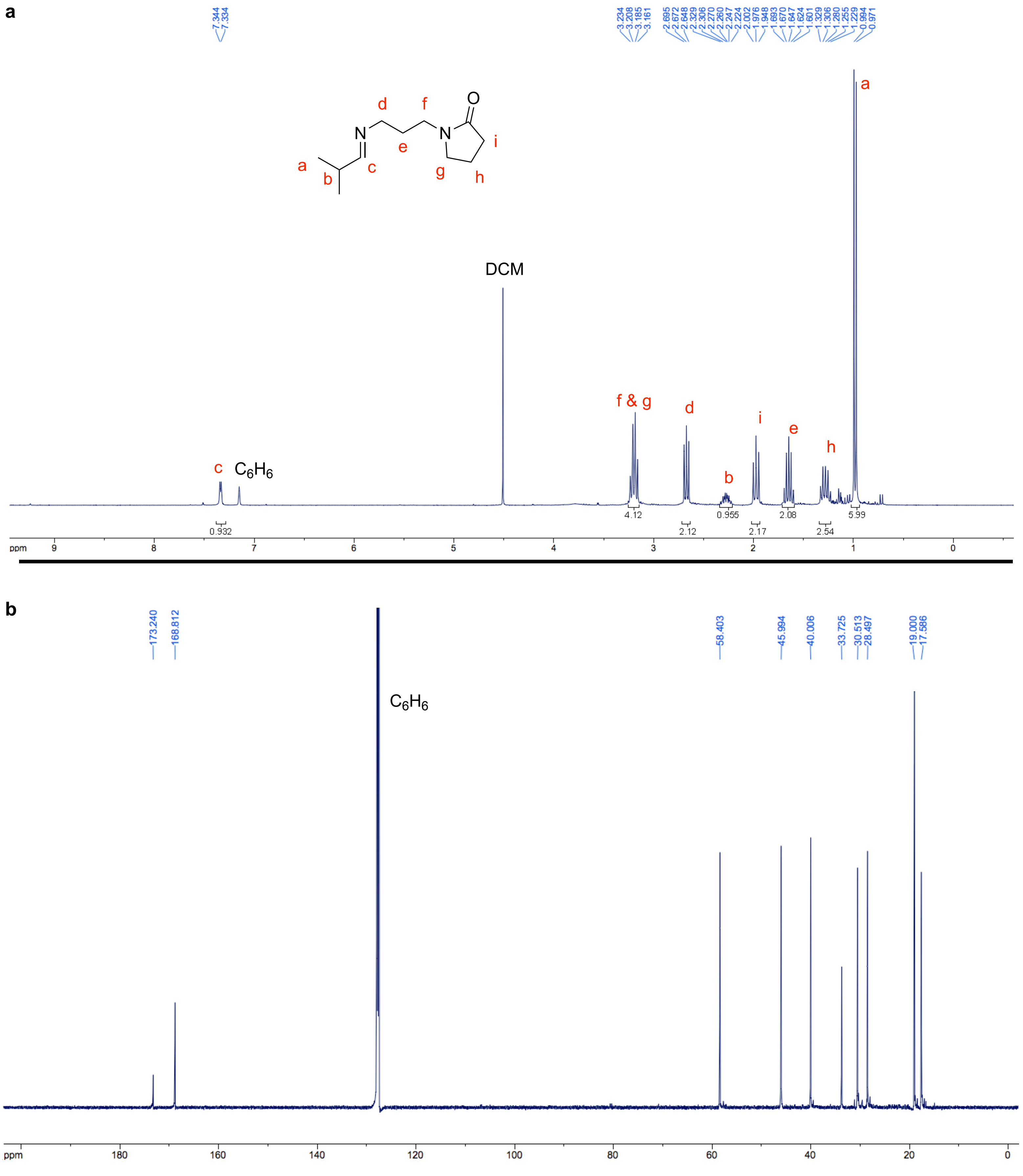

Supplementary Figure 36. NMR spectra of *N*-isobutylidene-1,3-propanepyrrolidone. **a** ^{1}H NMR (300 MHz, C_6D_6). **b** ^{13}C NMR (126 MHz, C_6D_6).

a

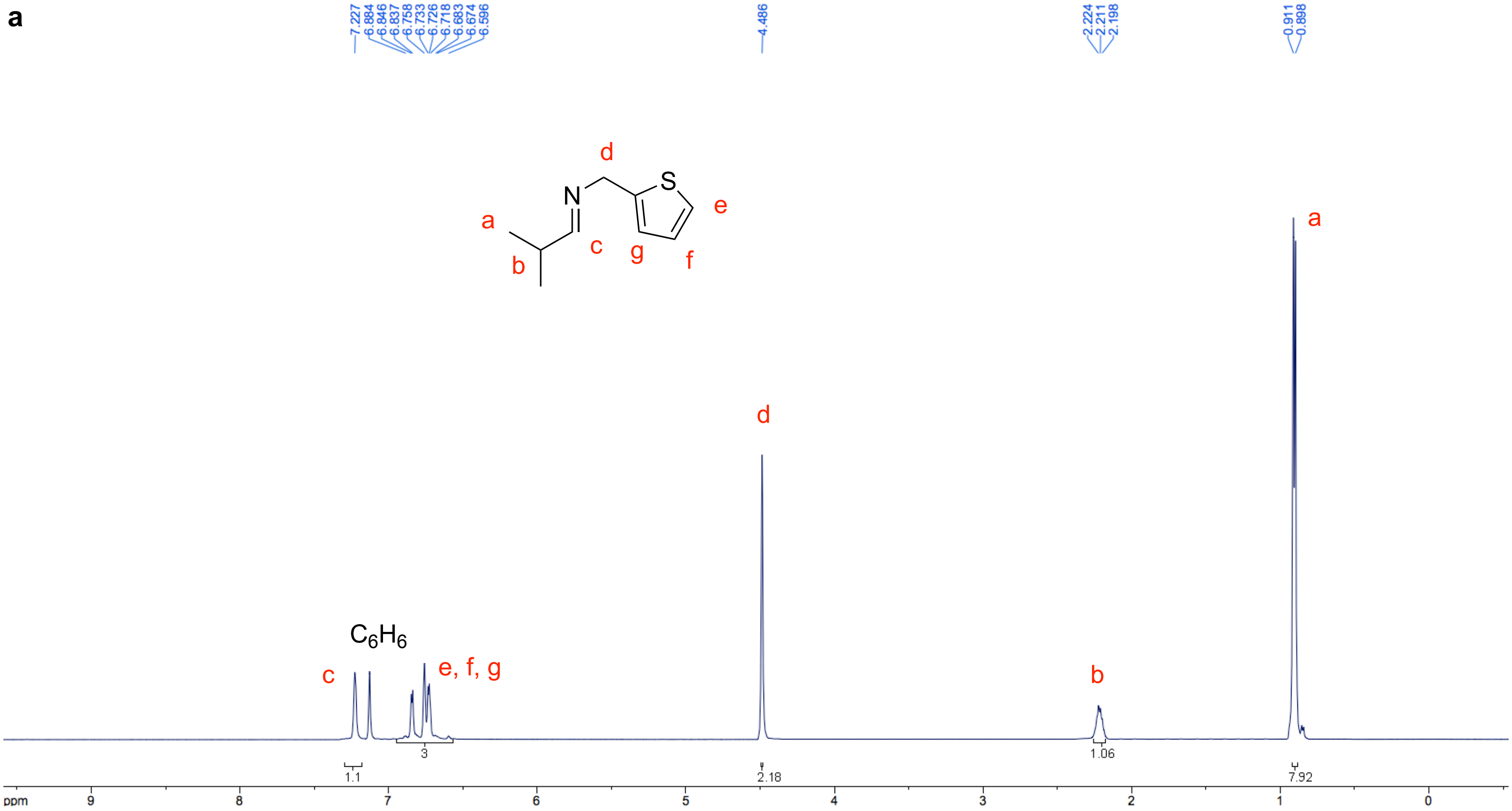

b

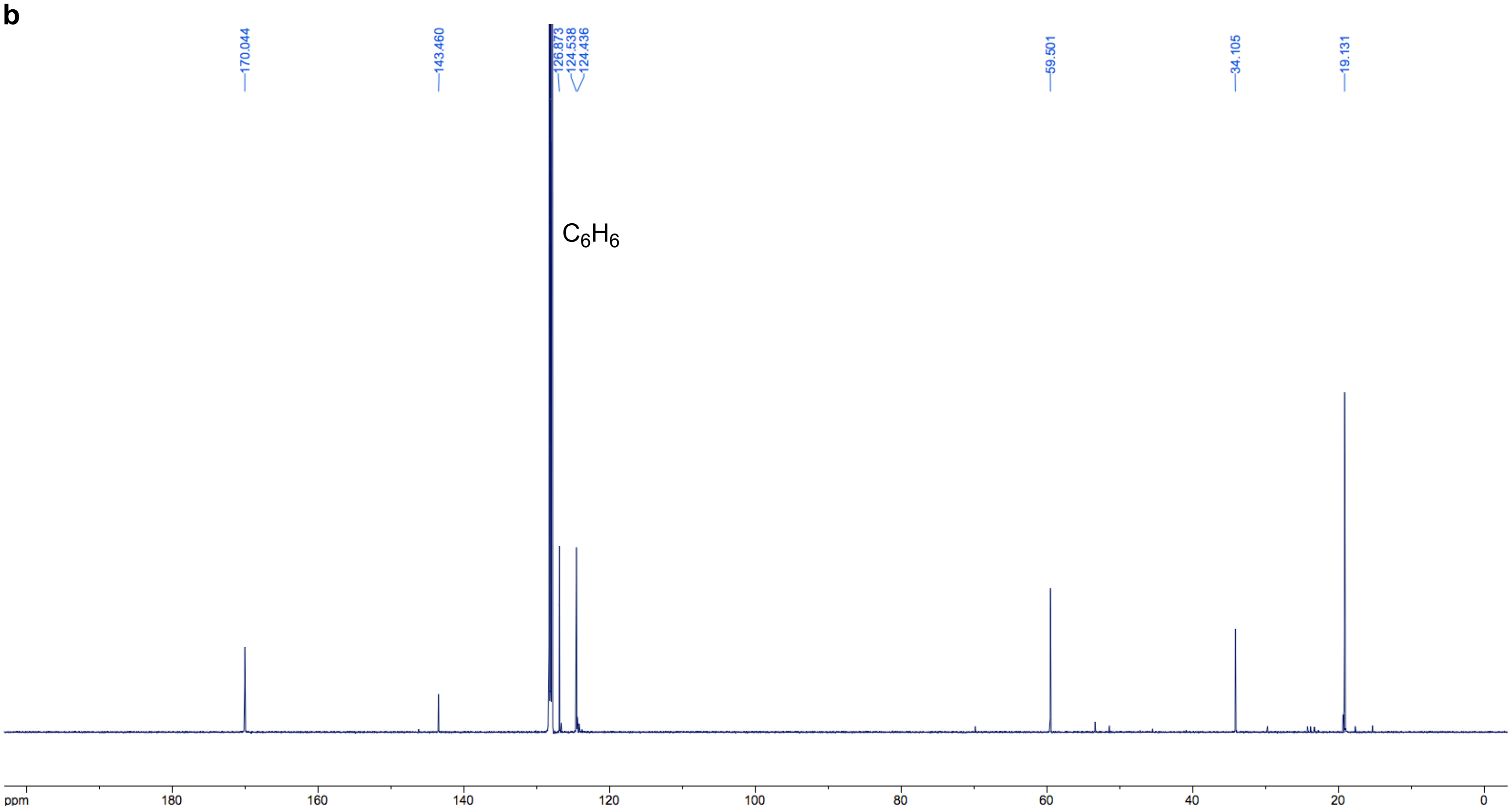

Supplementary Figure 37. NMR spectra of *N*-isobutylidene-methylenethiophene. **a** ^{1}H NMR (300 MHz, C_6D_6). **b** ^{13}C NMR (126 MHz, C_6D_6).

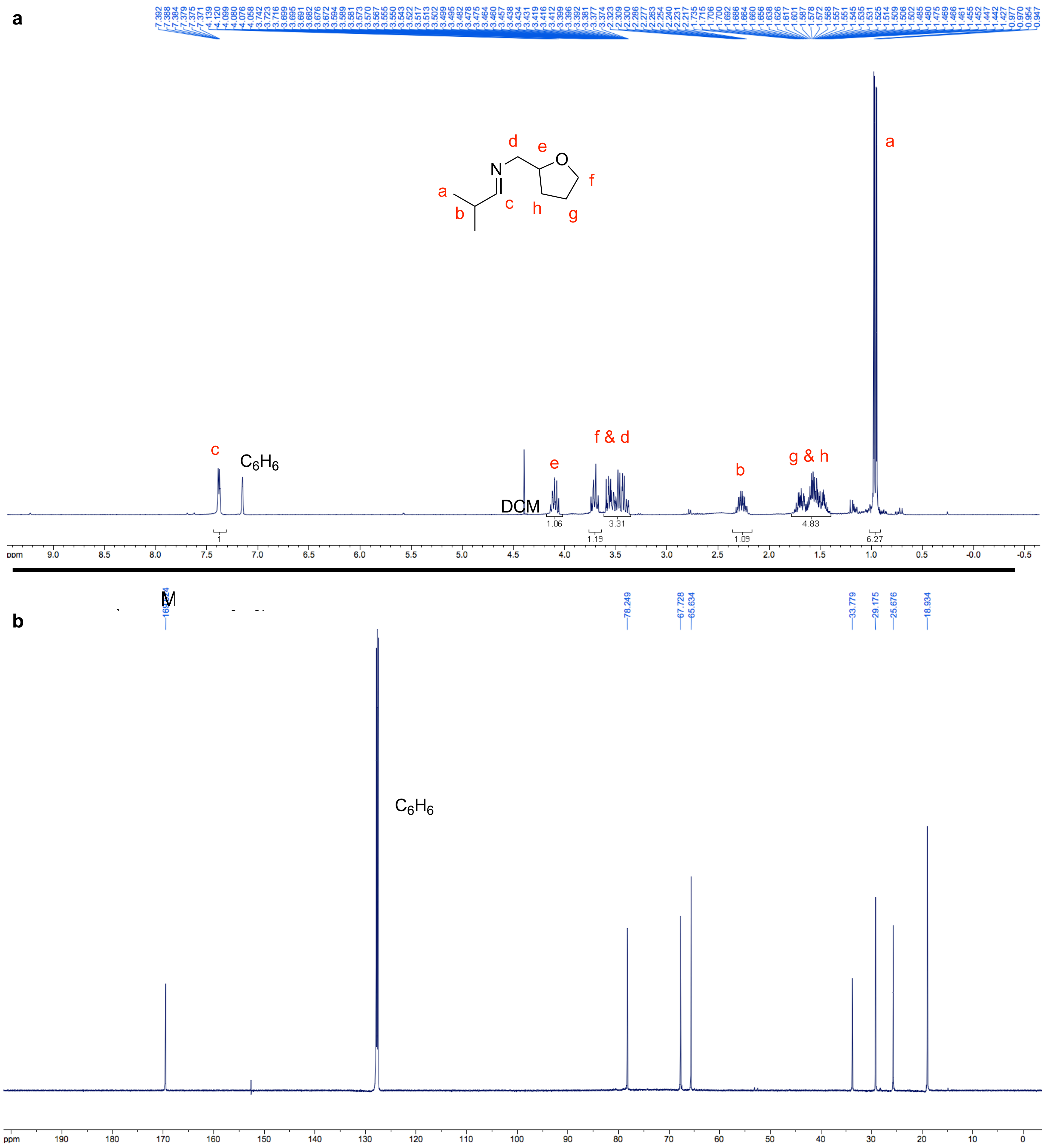

Supplementary Figure 38. NMR spectra of *N*-isobutylidene-methylenefuran. **a** ^{1}H NMR (300 MHz, C_6D_6). **b** ^{13}C NMR (126 MHz, C_6D_6).

a

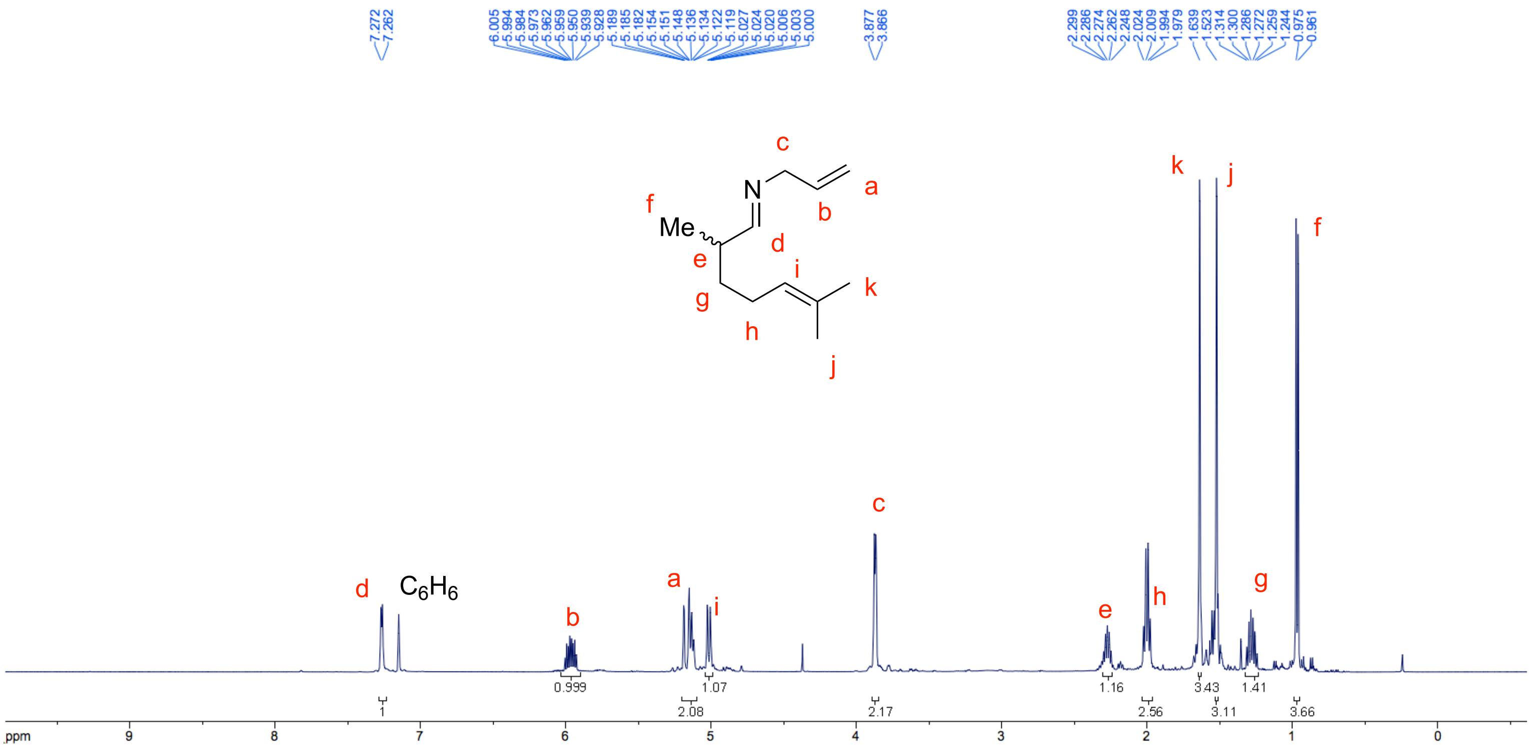

b

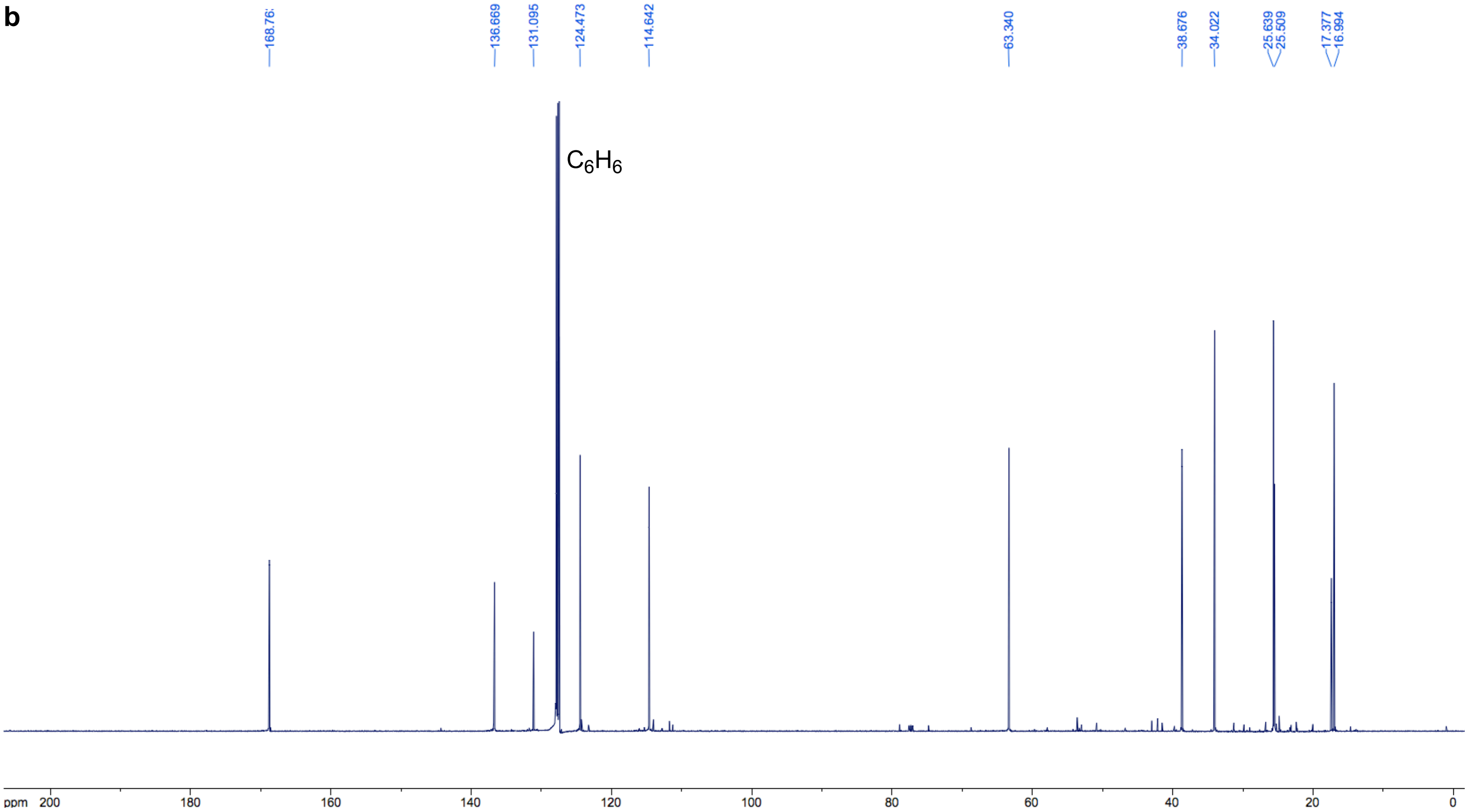

Supplementary Figure 39. NMR spectra of *N*-isobutylidene-melonal. **a** ^{1}H NMR (300 MHz, C_6D_6). **b** ^{13}C NMR (126 MHz, C_6D_6).

a

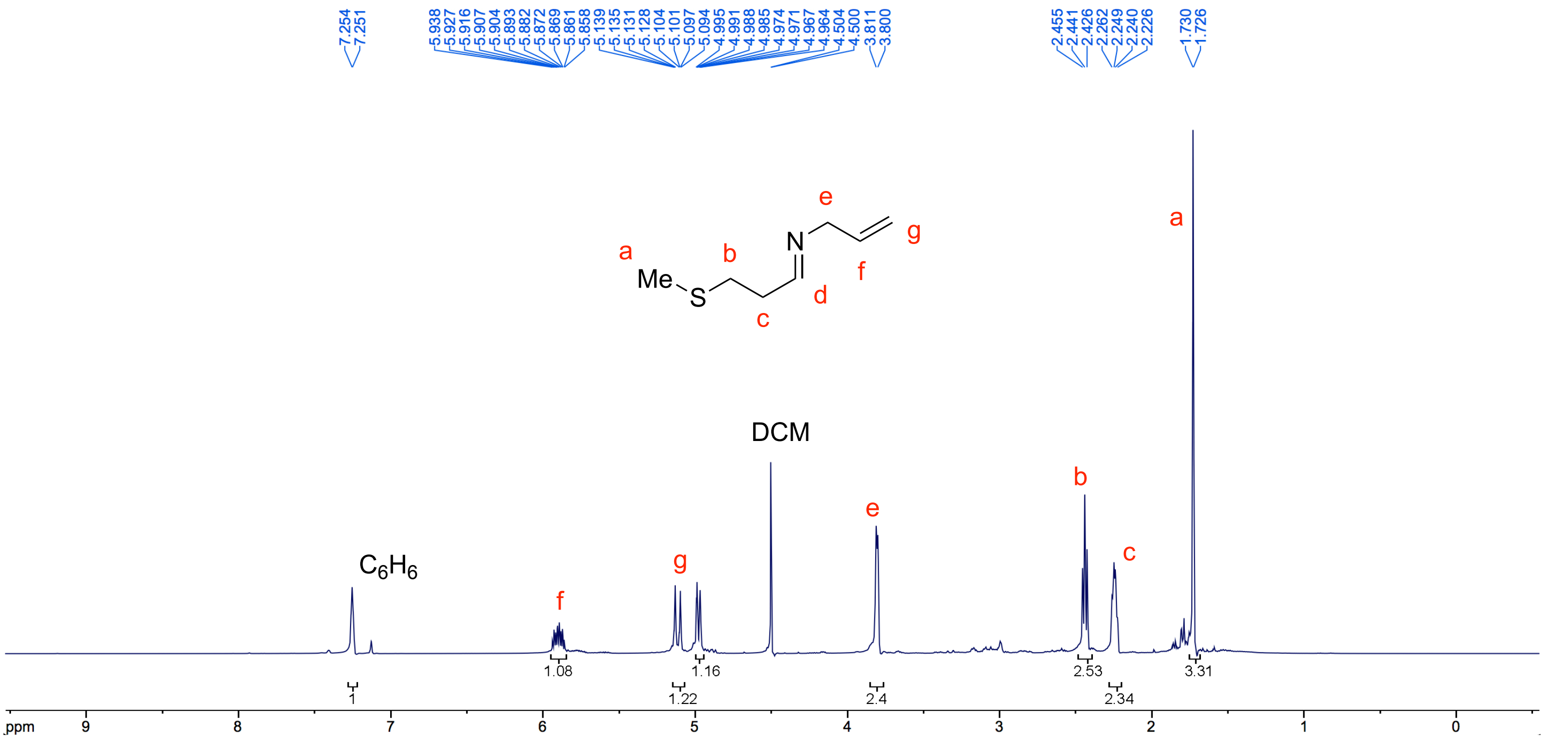

b

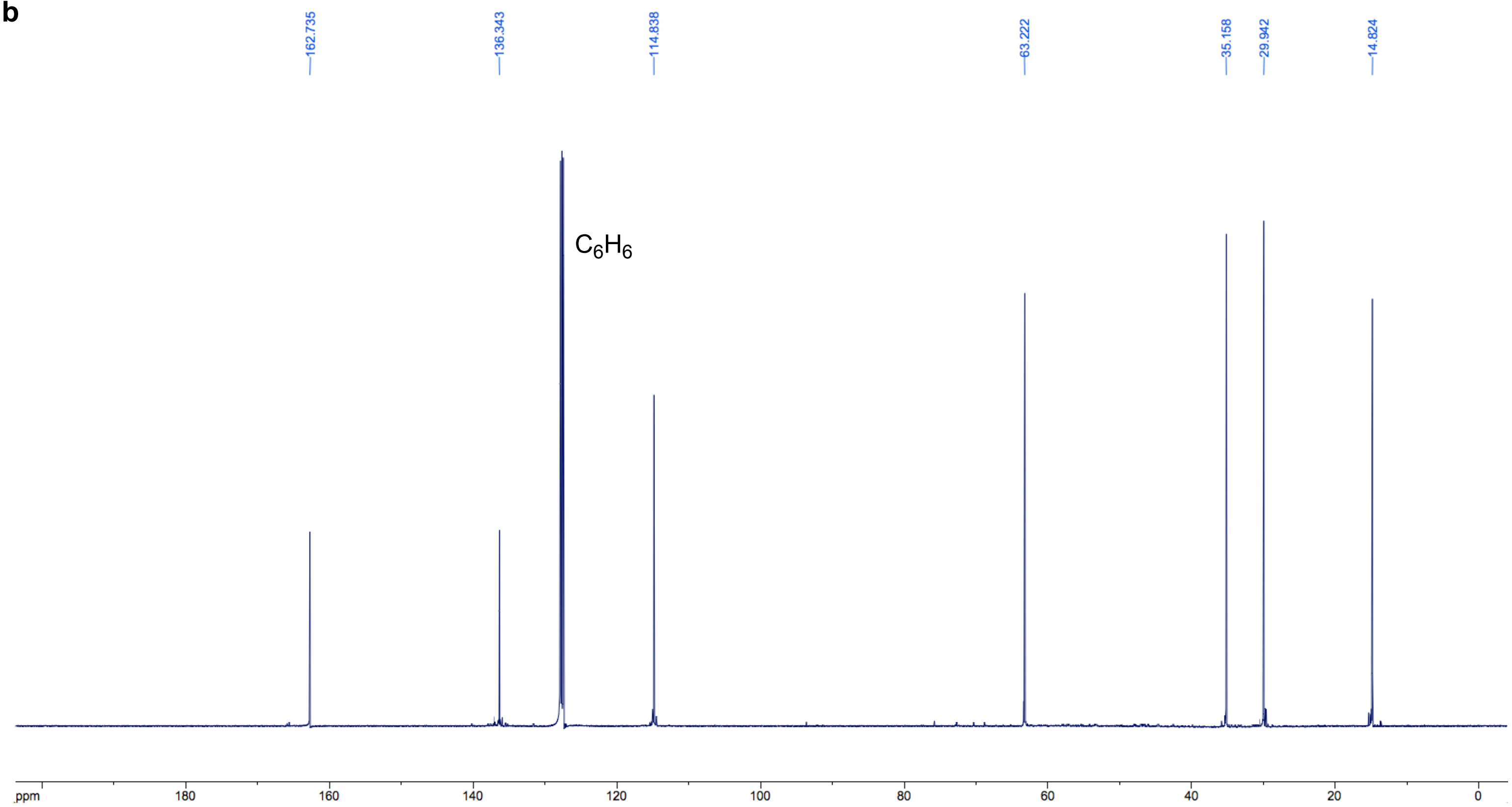

Supplementary Figure 40. NMR spectra of *N*-isobutylidene-methional. **a** ^{1}H NMR (300 MHz, C_6D_6). **b** ^{13}C NMR (126 MHz, C_6D_6).

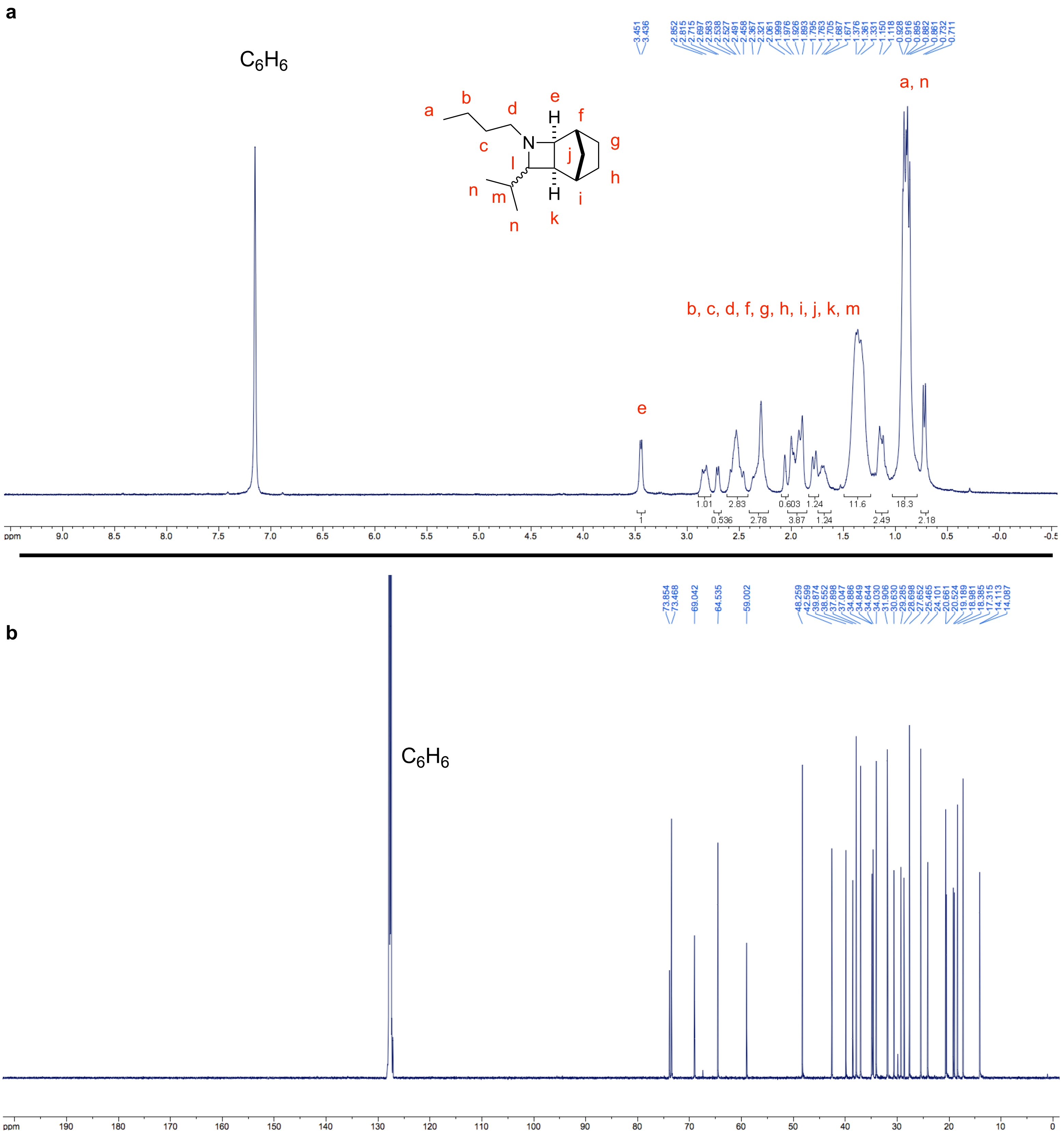

Supplementary Figure 41. NMR spectra of **1A**. **a** ^{1}H NMR (300 MHz, C_6D_6). **b** ^{13}C NMR (126 MHz, C_6D_6).

a

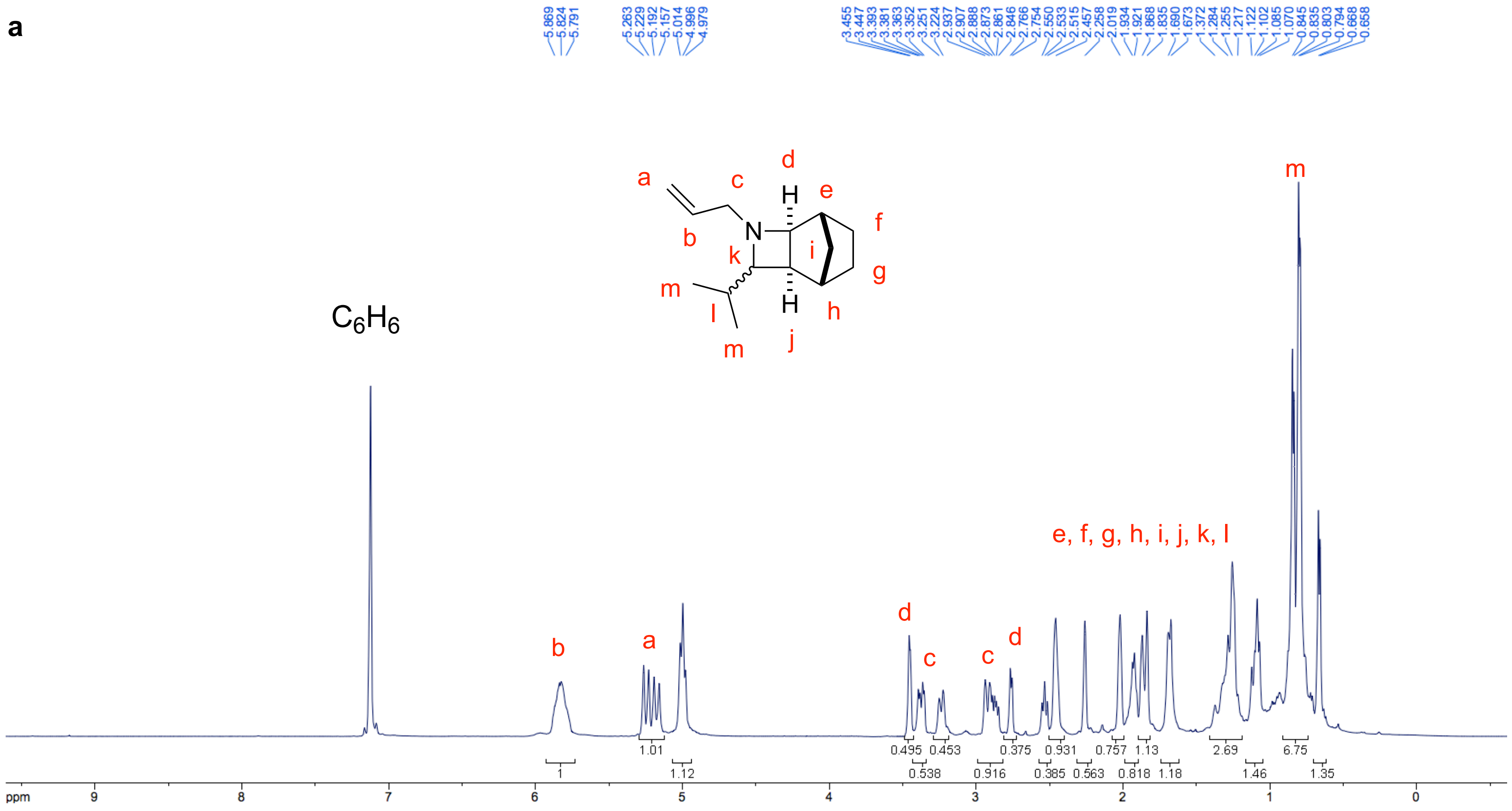

b

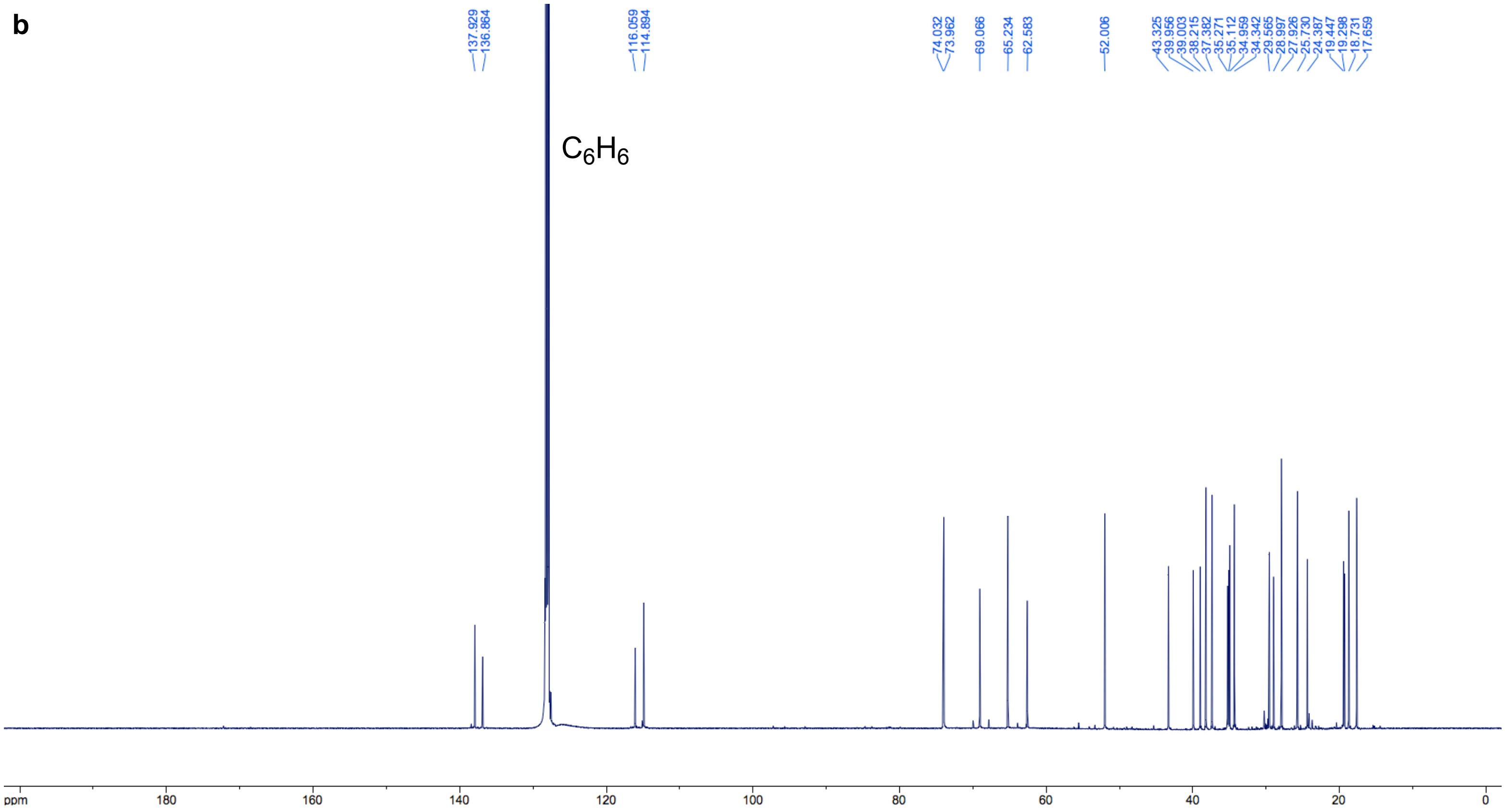

Supplementary Figure 42. NMR spectra of **2**. **a** ^{1}H NMR (300 MHz, C_6D_6). **b** ^{13}C NMR (126 MHz, C_6D_6).

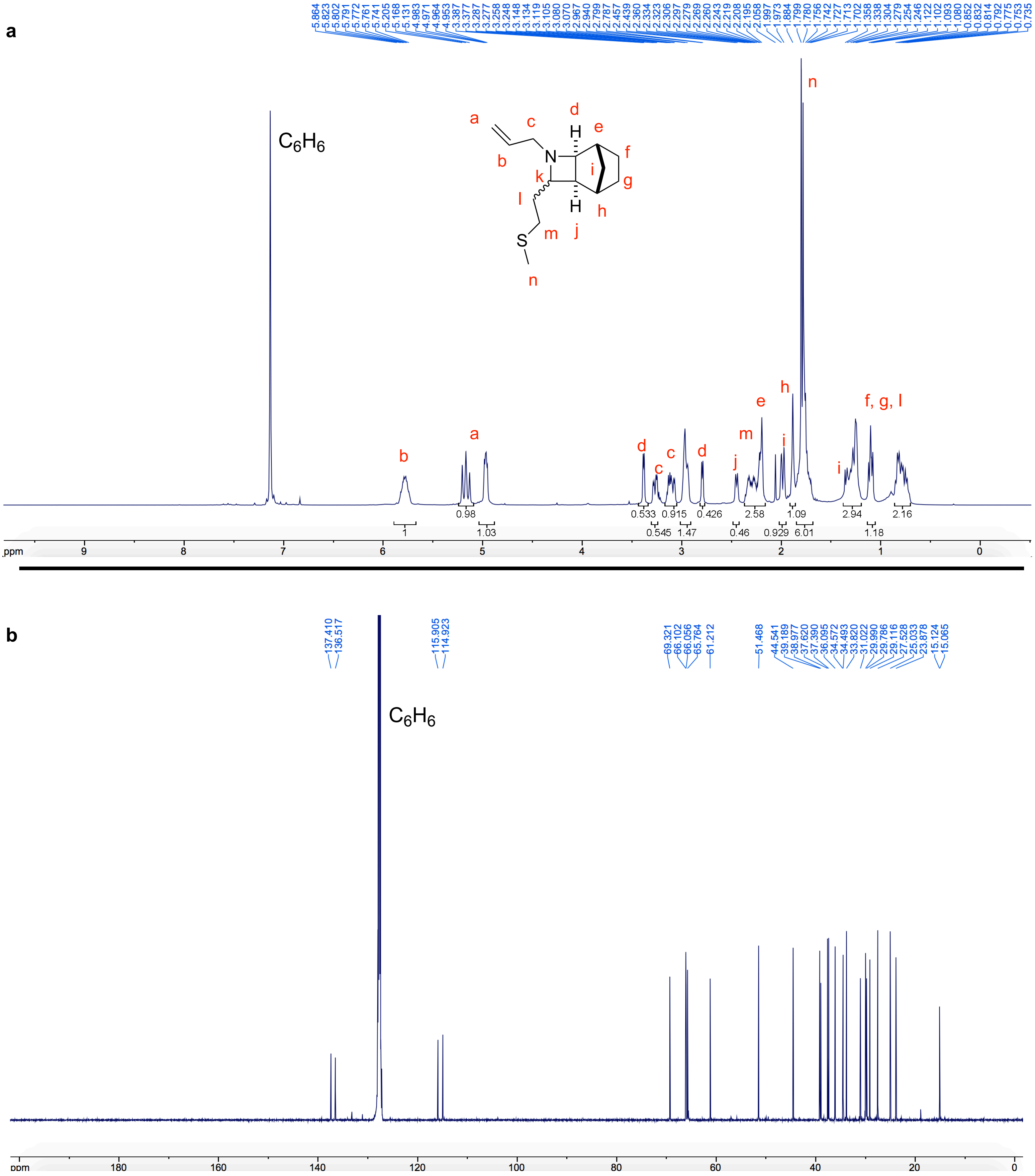

Supplementary Figure 43. NMR spectra of **3**. **a** ^{1}H NMR (300 MHz, C_6D_6). **b** ^{13}C NMR (126 MHz, C_6D_6).

a

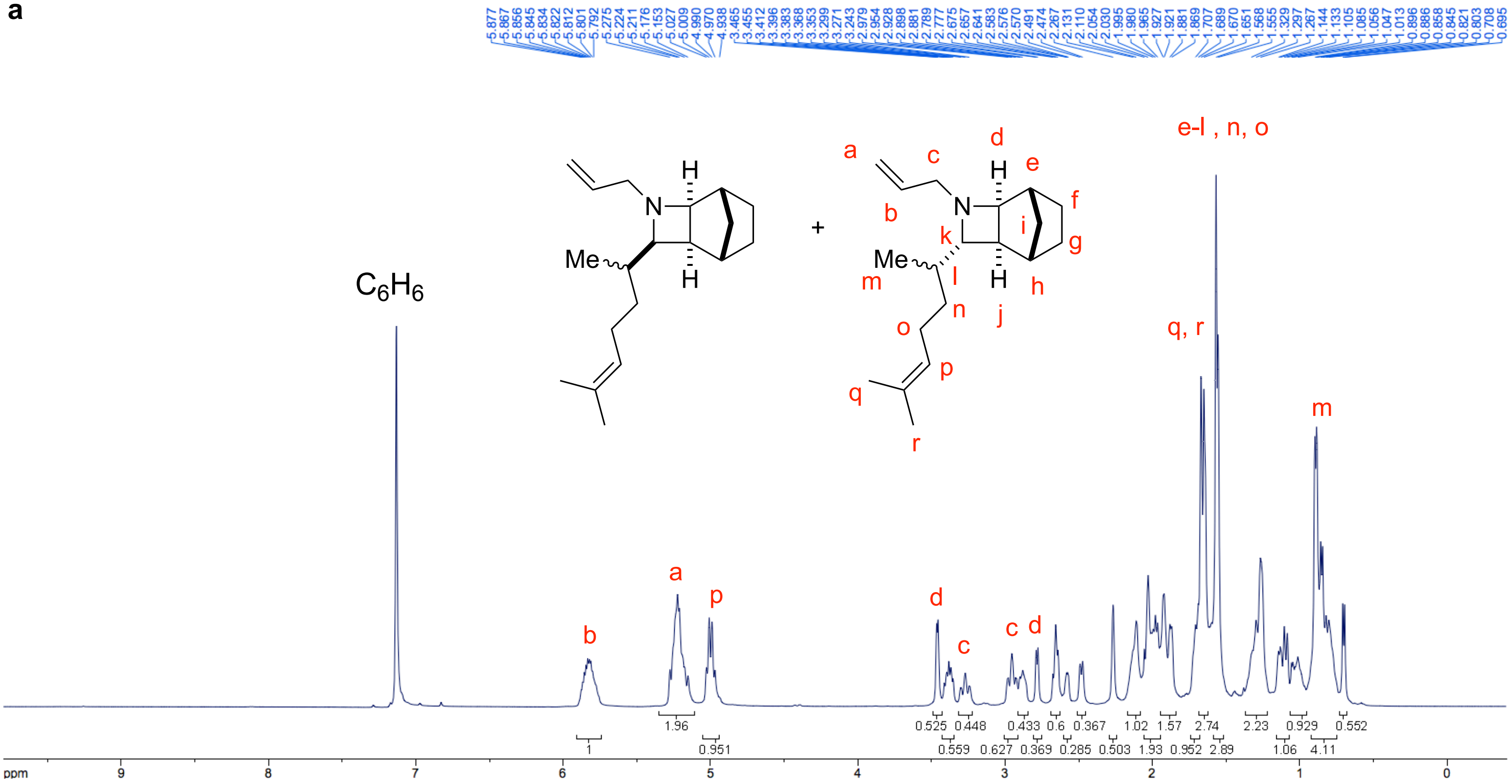

b

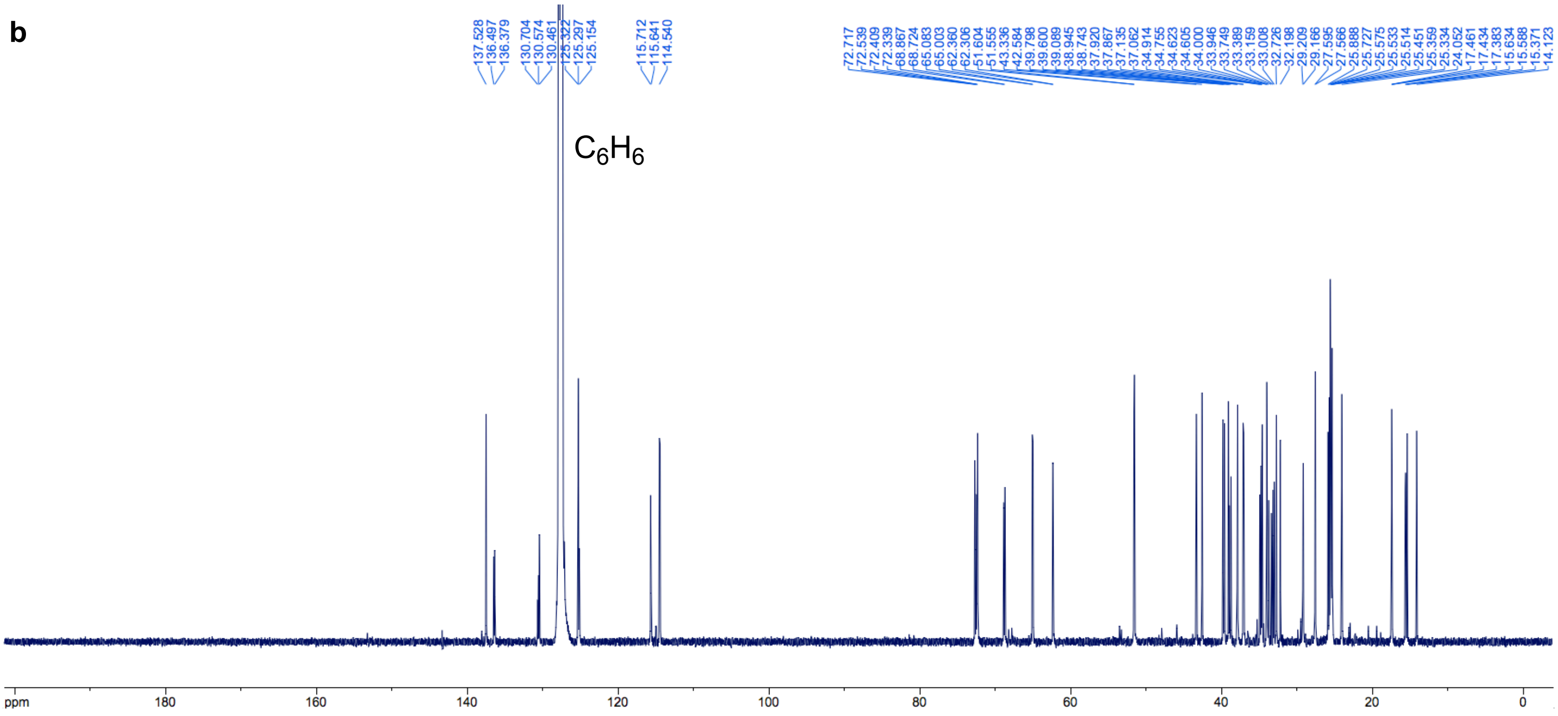

Supplementary Figure 44. NMR spectra of **4**. **a** ^{1}H NMR (300 MHz, C_6D_6). **b** ^{13}C NMR (126 MHz, C_6D_6).

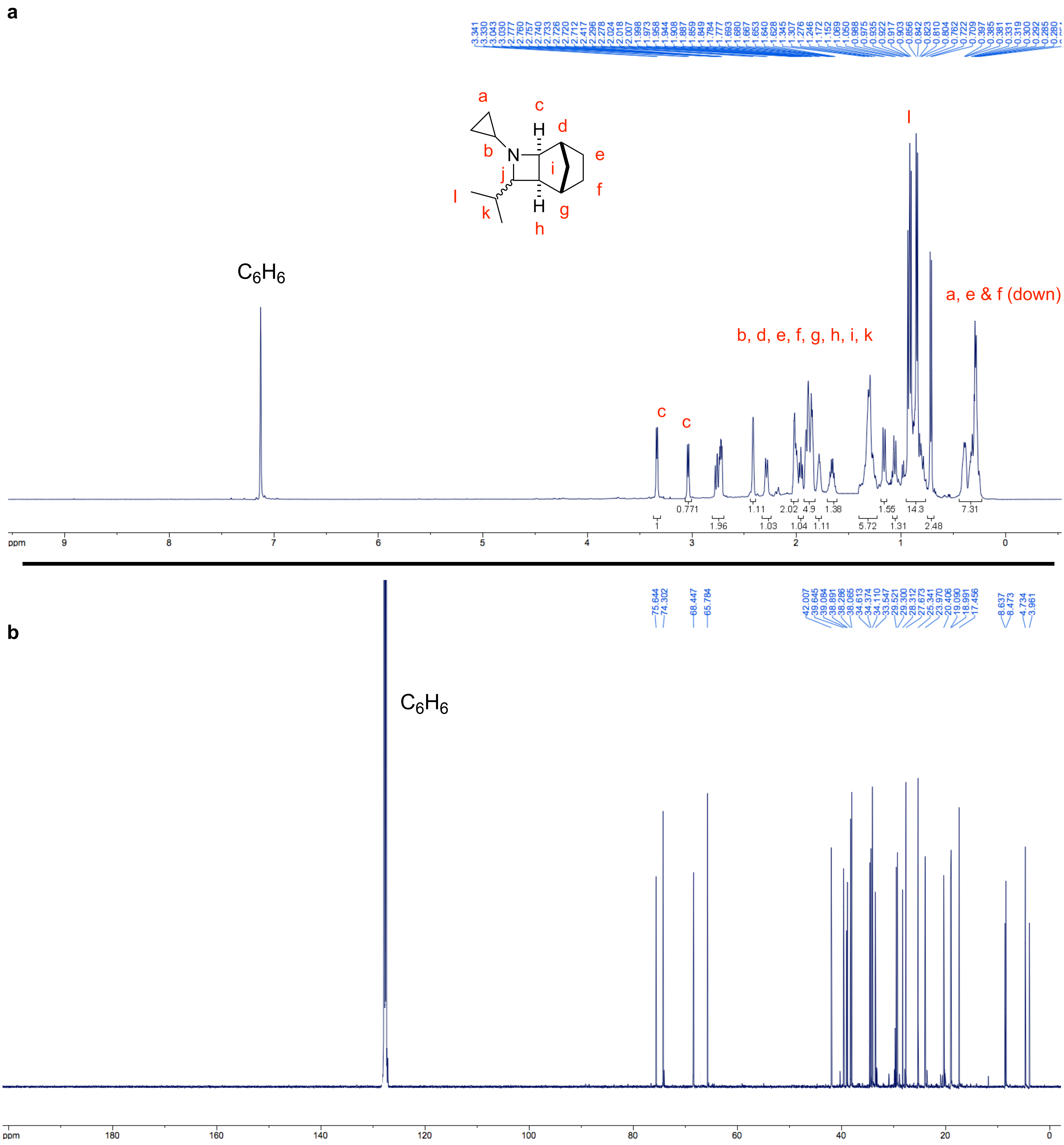

Supplementary Figure 45. NMR spectra of **5**. **a** ^{1}H NMR (300 MHz, C_6D_6). **b** ^{13}C NMR (126 MHz, C_6D_6).

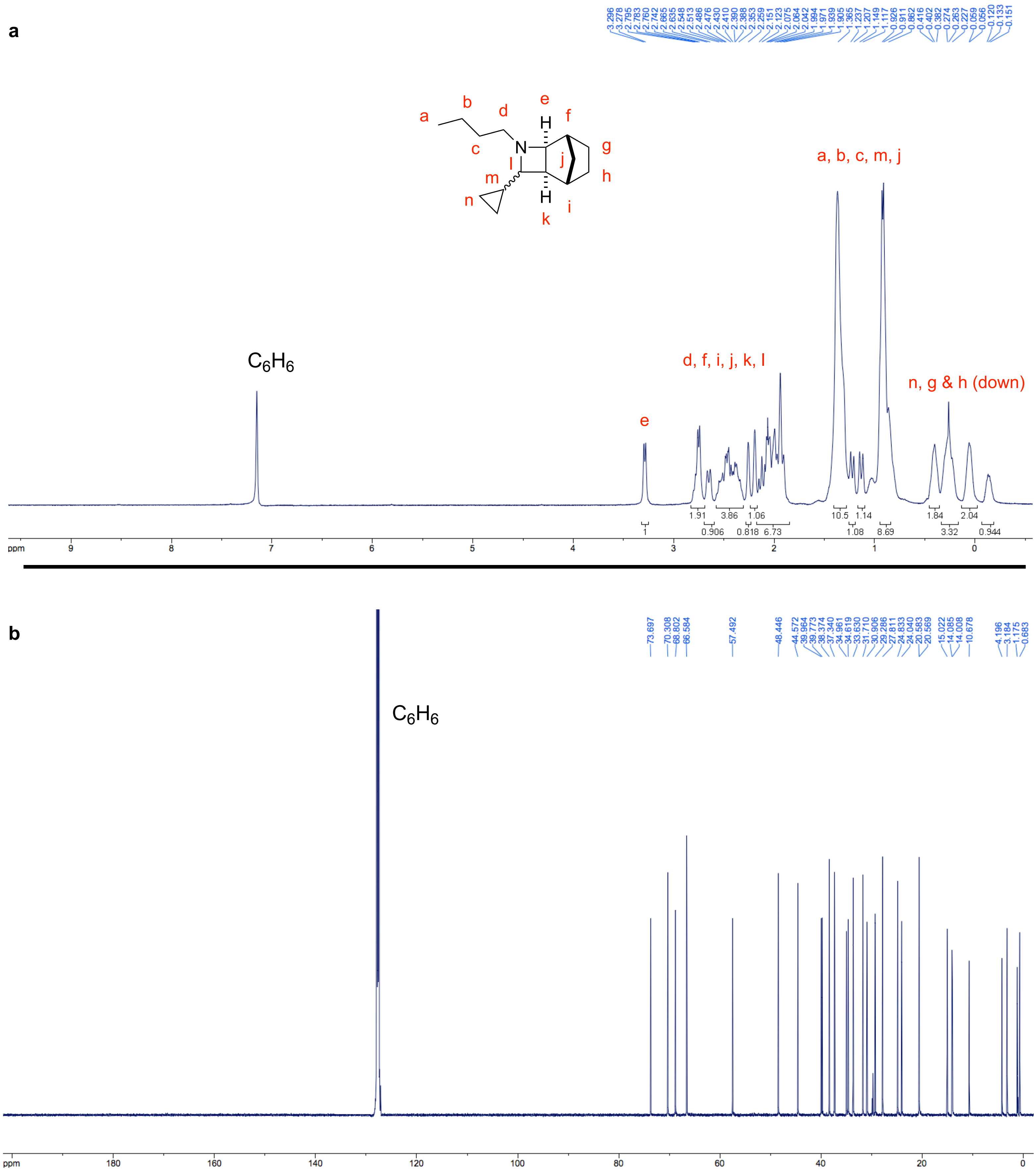

Supplementary Figure 46. NMR spectra of **6**. **a** ^{1}H NMR (300 MHz, C_6D_6). **b** ^{13}C NMR (126 MHz, C_6D_6).

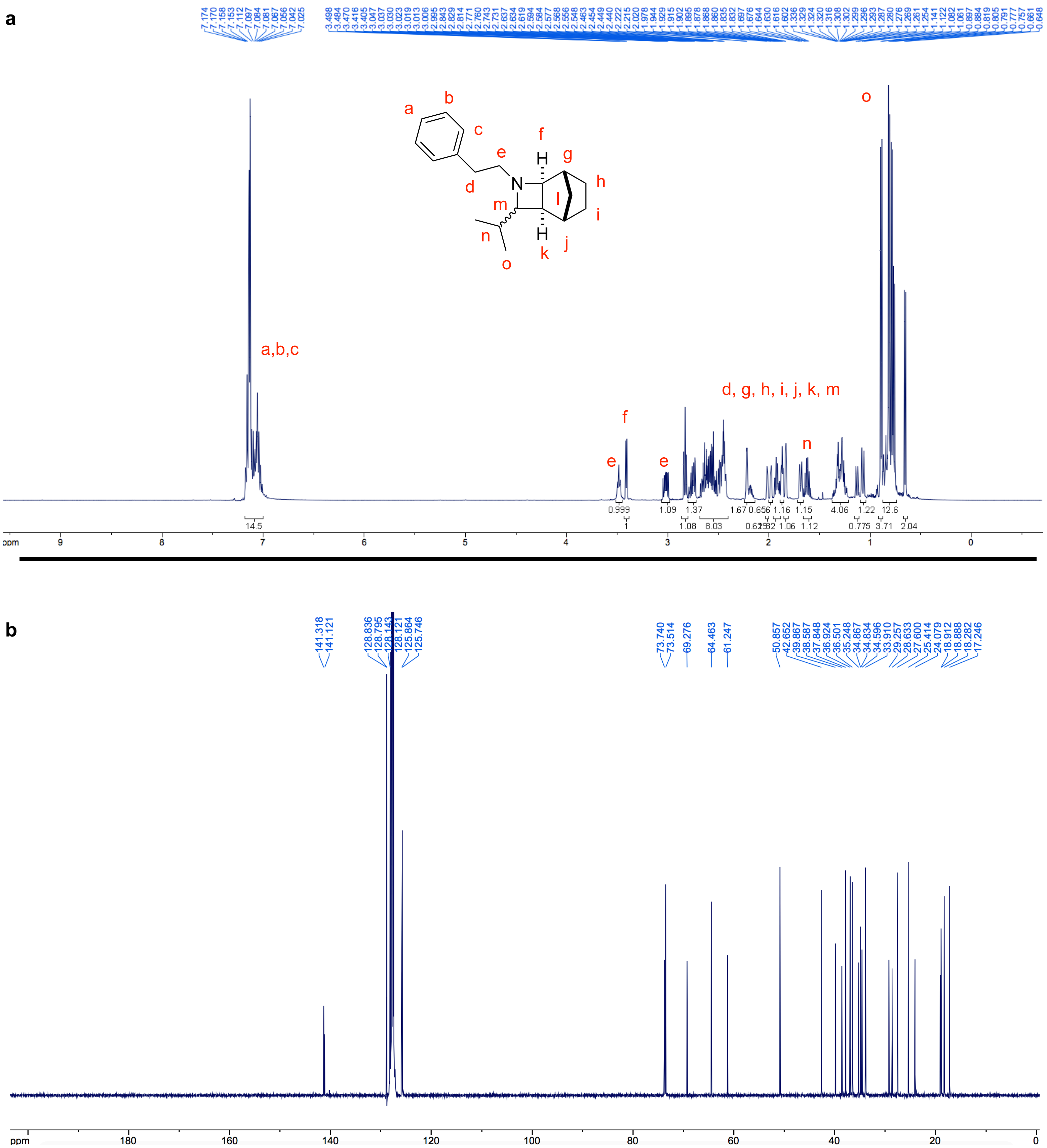

Supplementary Figure 47. NMR spectra of **7**. **a** ^{1}H NMR (300 MHz, C_6D_6). **b** ^{13}C NMR (126 MHz, C_6D_6).

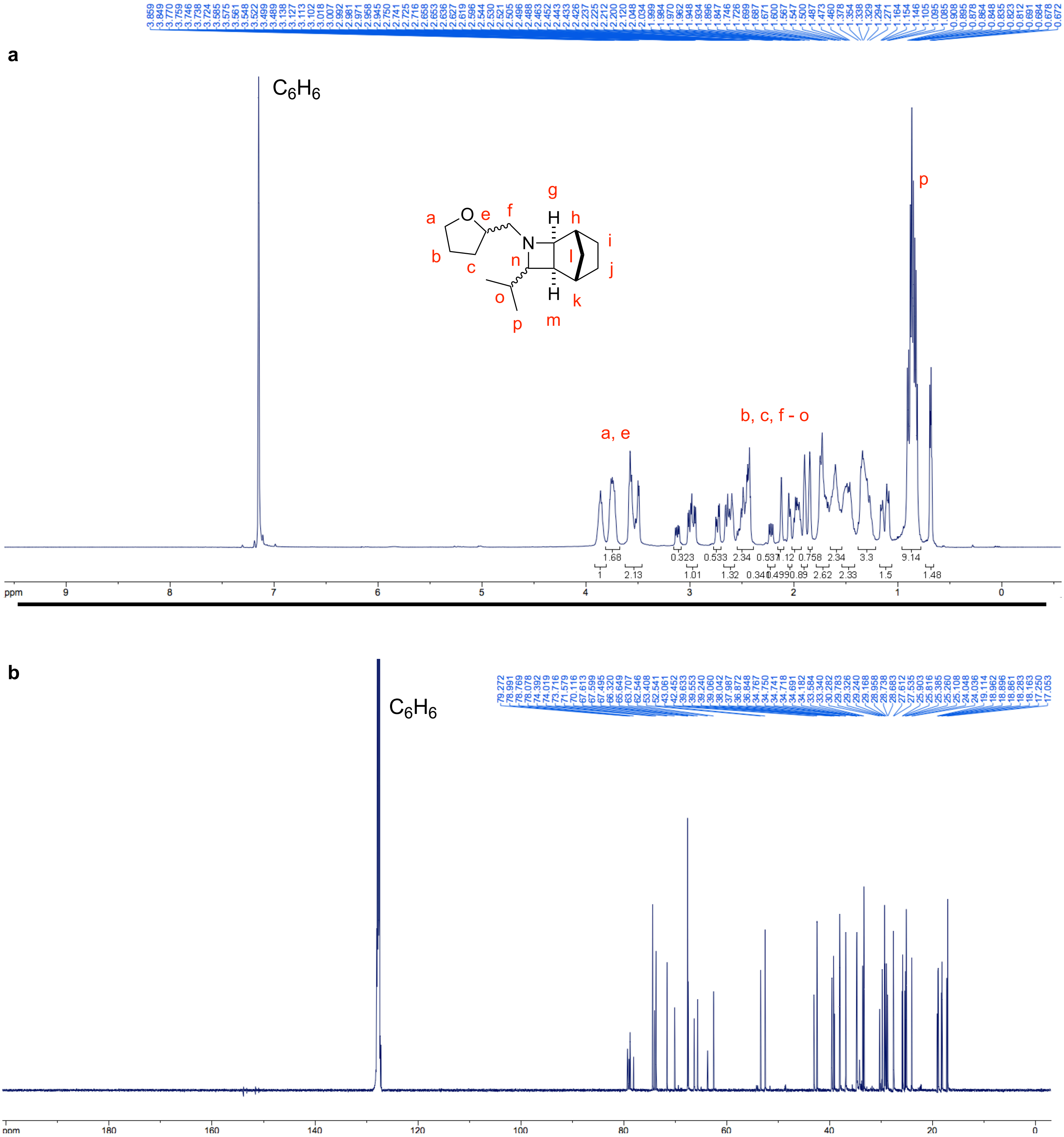

Supplementary Figure 48. NMR spectra of **8**. **a** ^{1}H NMR (300 MHz, C_6D_6). **b** ^{13}C NMR (126 MHz, C_6D_6).

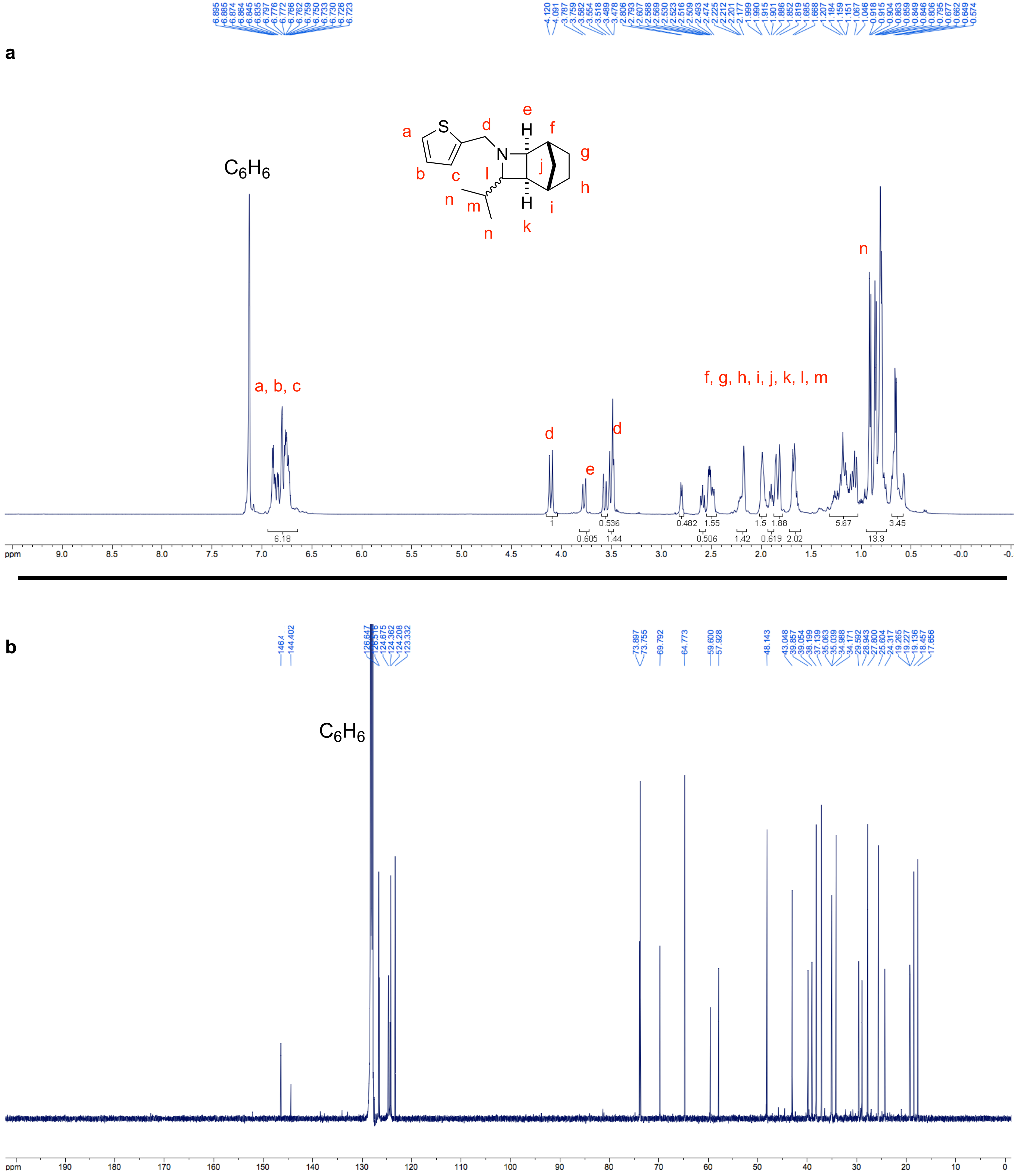

Supplementary Figure 49. NMR spectra of **9**. **a** ^{1}H NMR (300 MHz, C_6D_6). **b** ^{13}C NMR (126 MHz, C_6D_6).

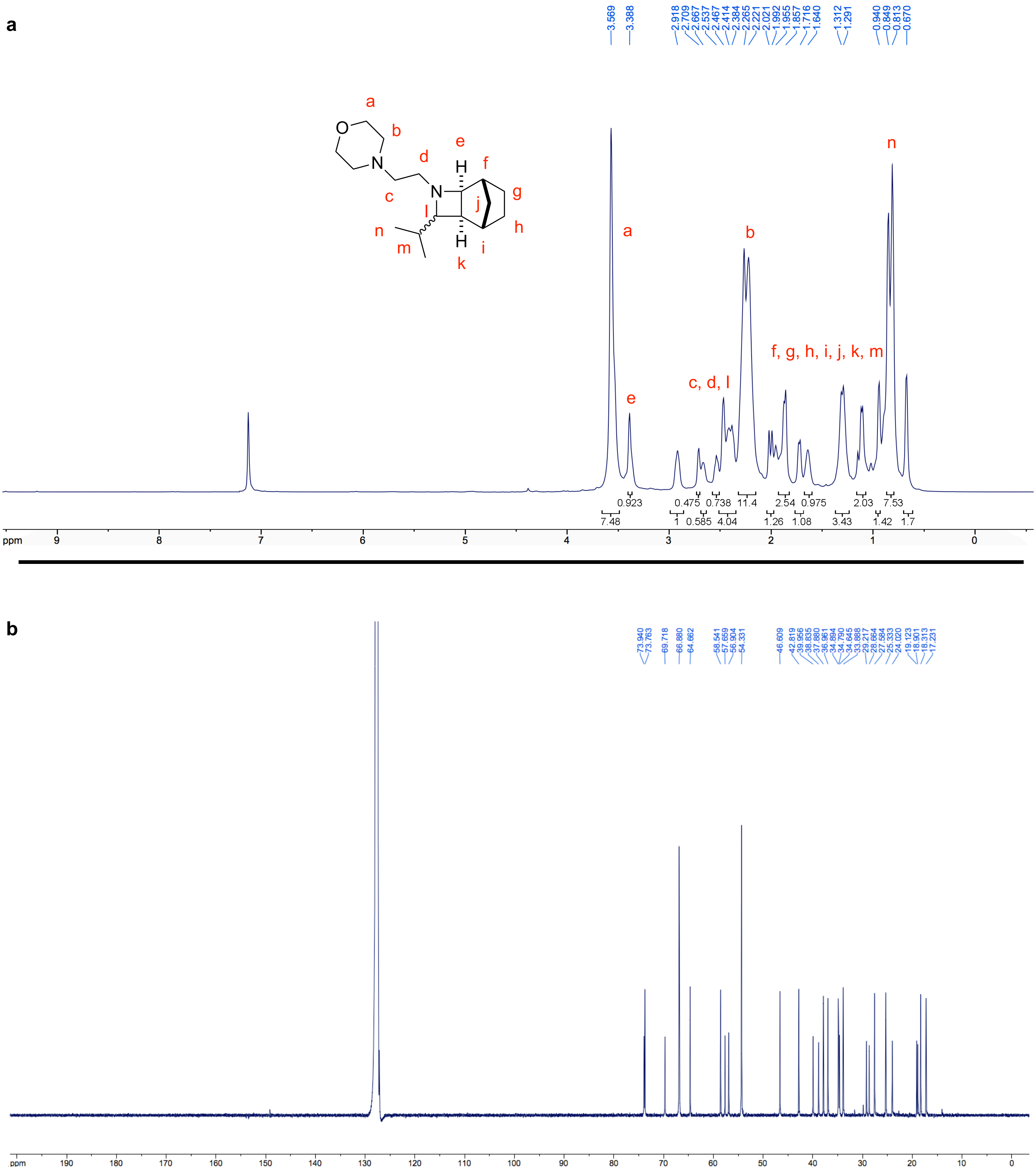

Supplementary Figure 50. NMR spectra of **10**. **a** ^{1}H NMR (300 MHz, C_6D_6). **b** ^{13}C NMR (126 MHz, C_6D_6).

a

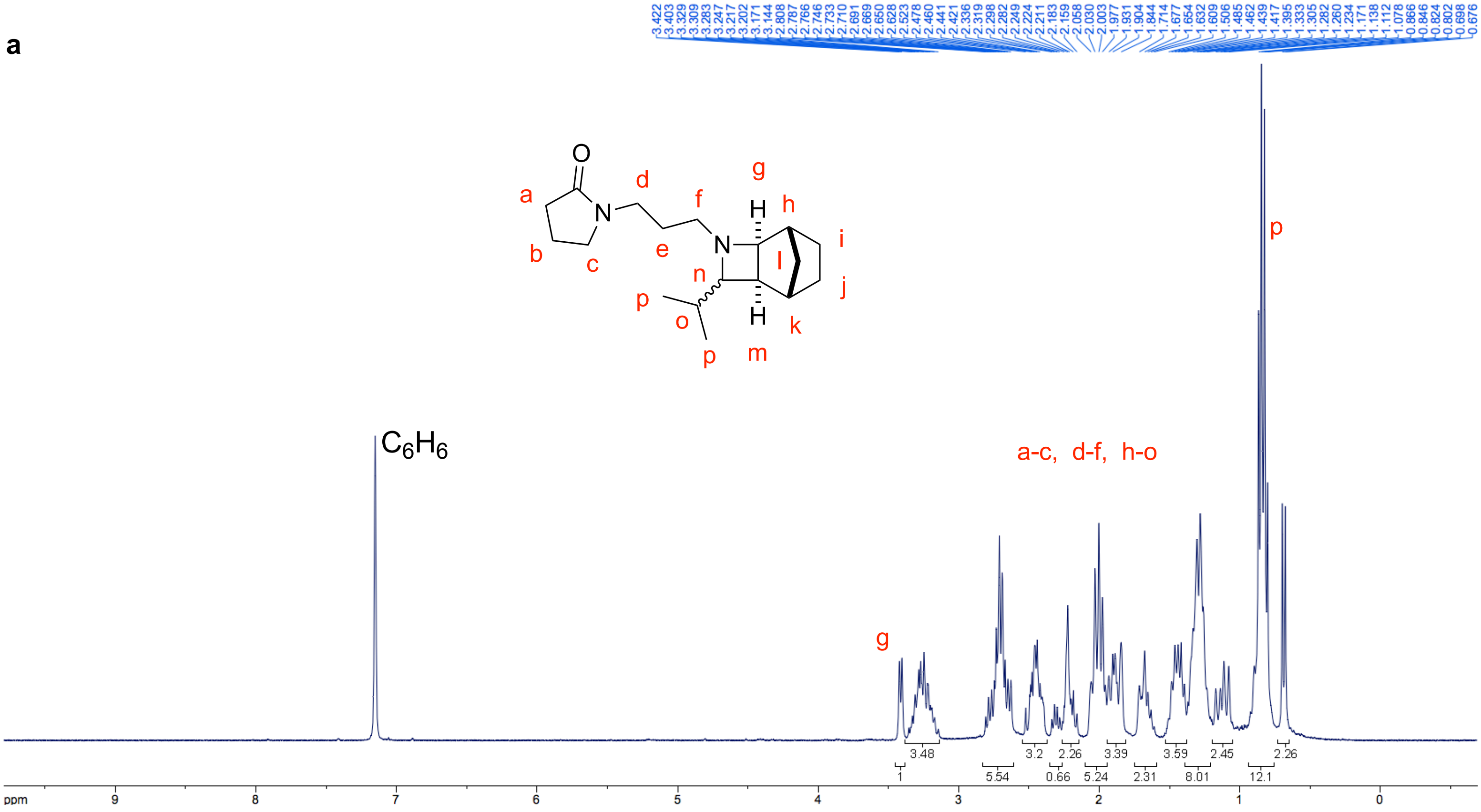

b

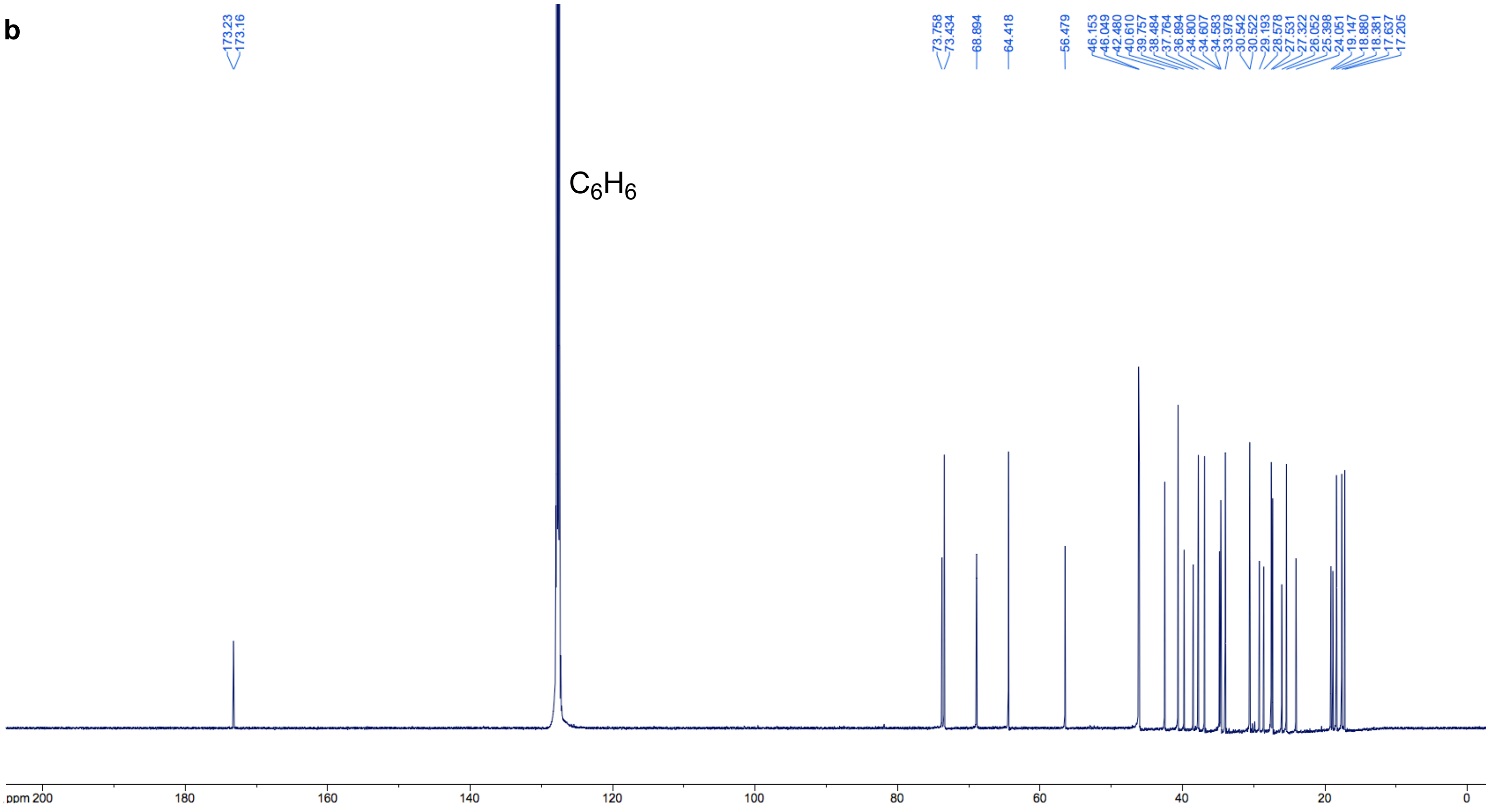

Supplementary Figure 51. NMR spectra of **11**. **a** ^{1}H NMR (300 MHz, C_6D_6). **b** ^{13}C NMR (126 MHz, C_6D_6).

a

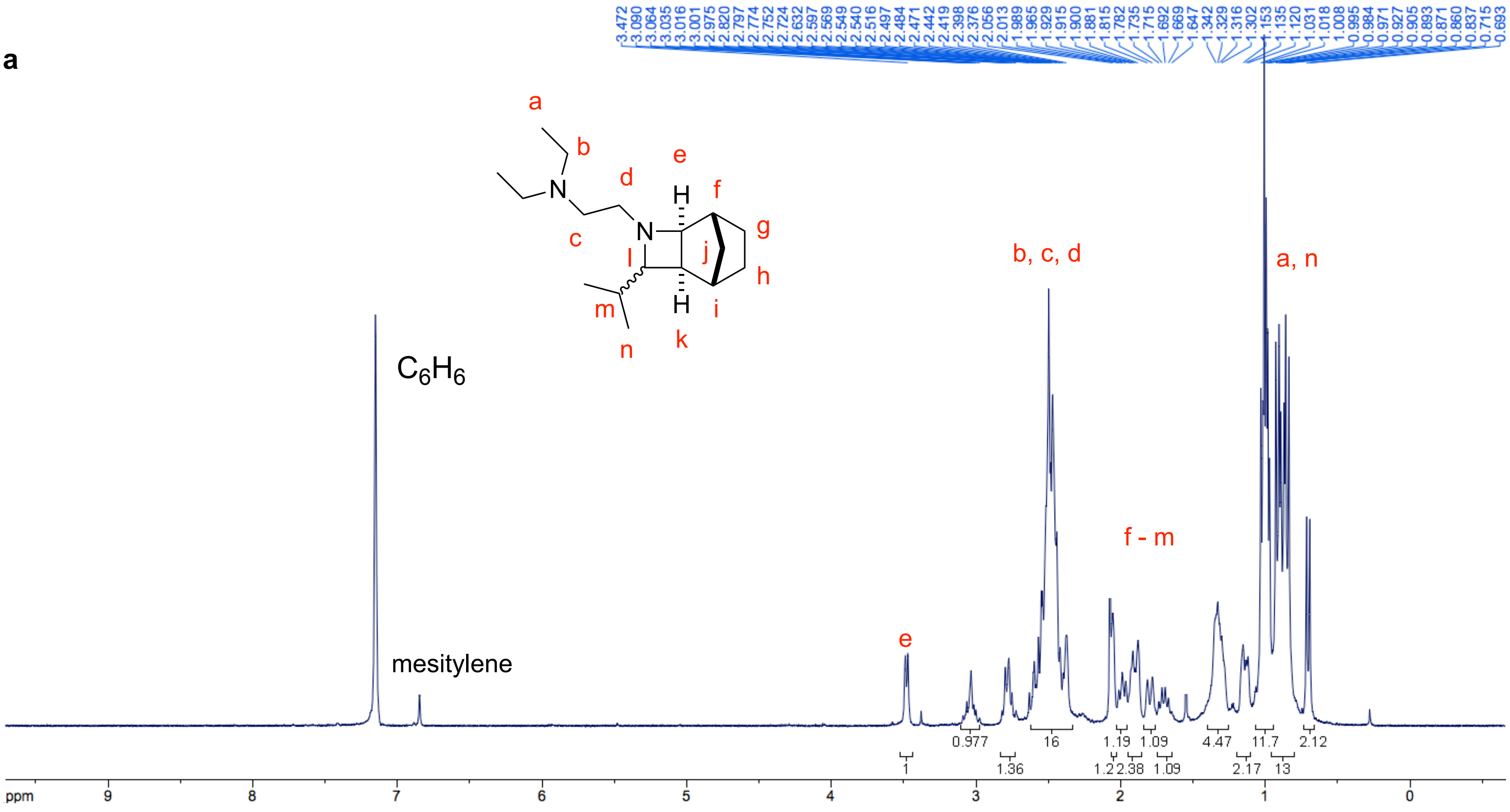

b

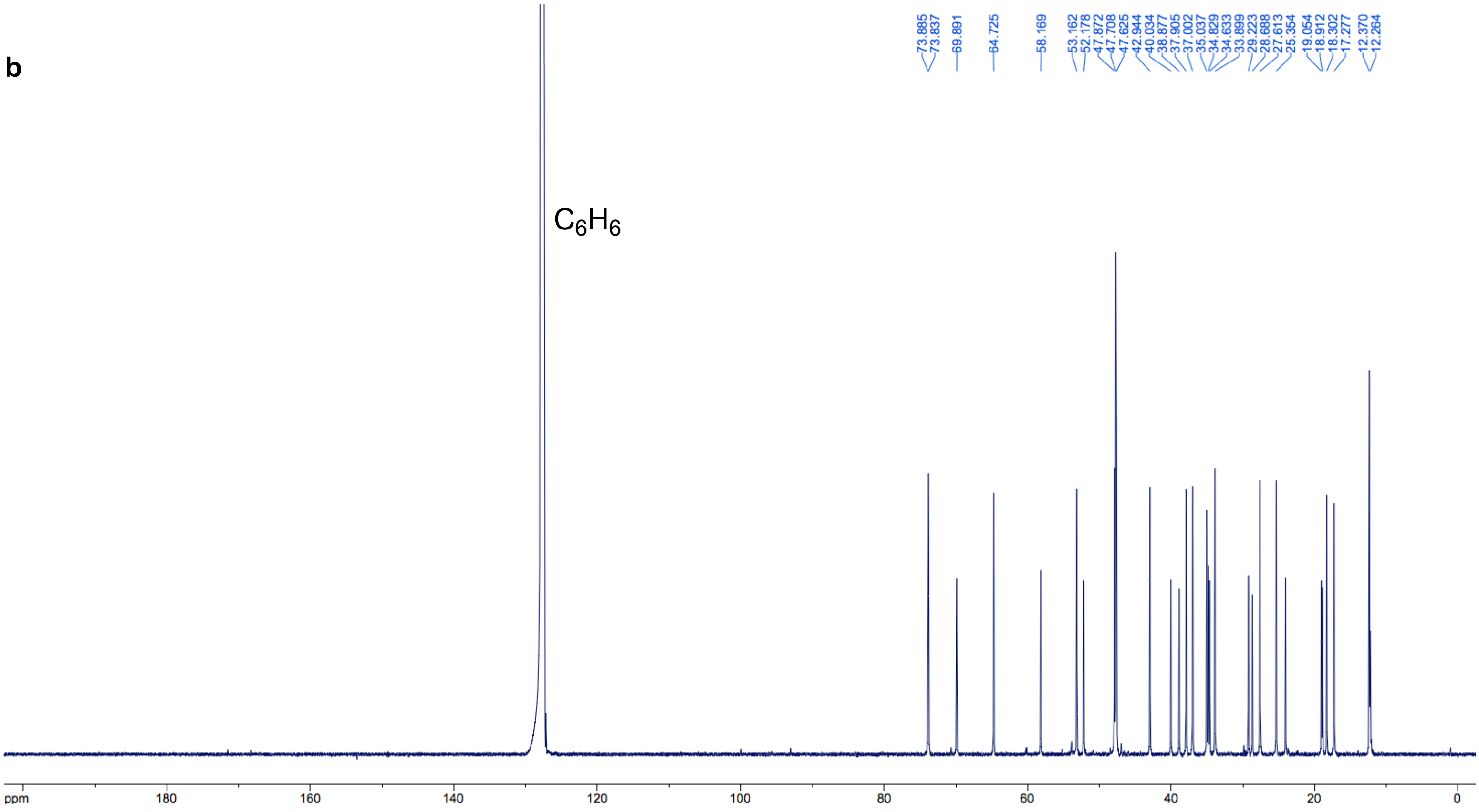

Supplementary Figure 52. NMR spectra of **12**. **a** ^{1}H NMR (300 MHz, C_6D_6). **b** ^{13}C NMR (126 MHz, C_6D_6).

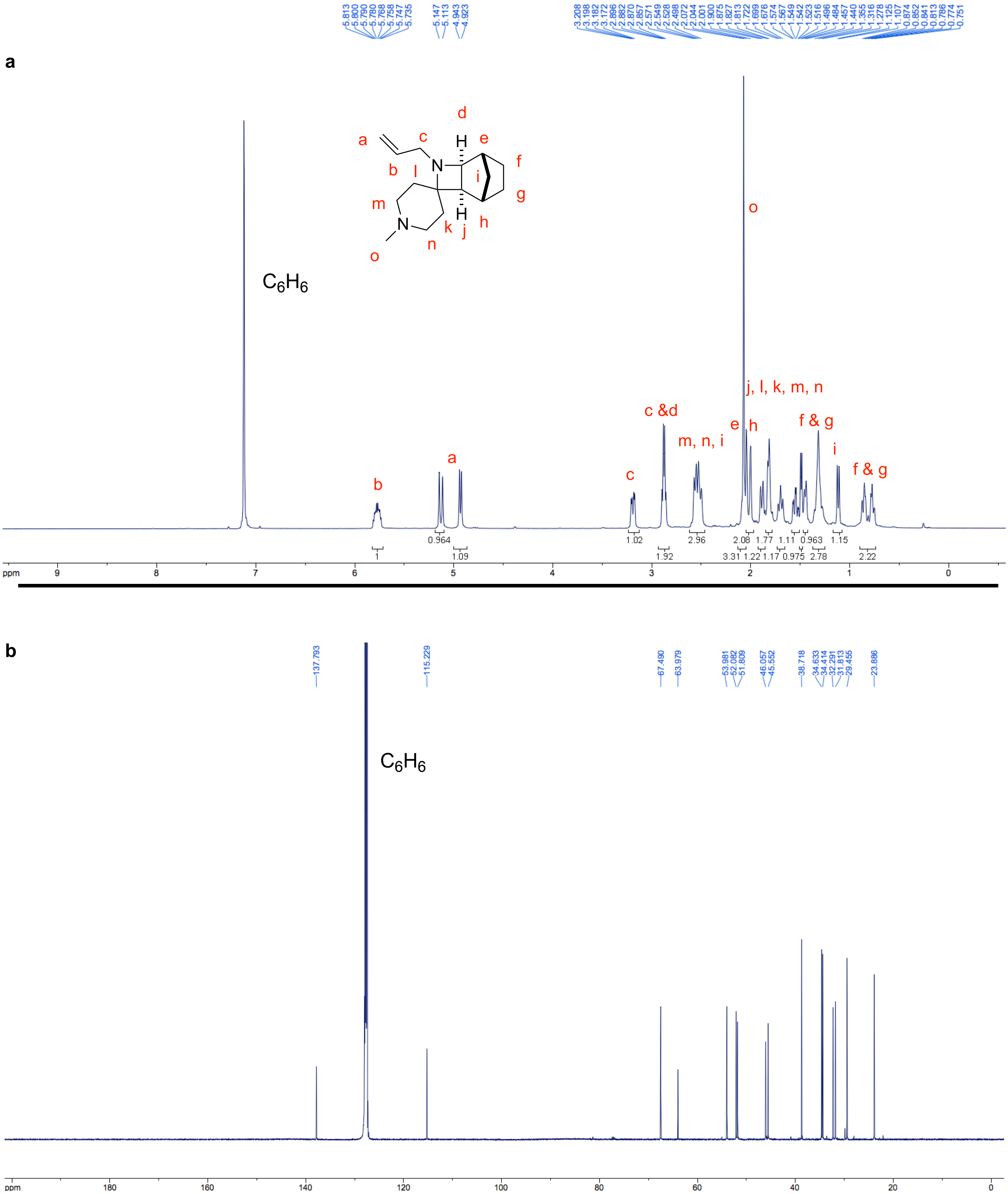

Supplementary Figure 53. NMR spectra of **13**. **a** ^{1}H NMR (300 MHz, C_6D_6). **b** ^{13}C NMR (126 MHz, C_6D_6).

a

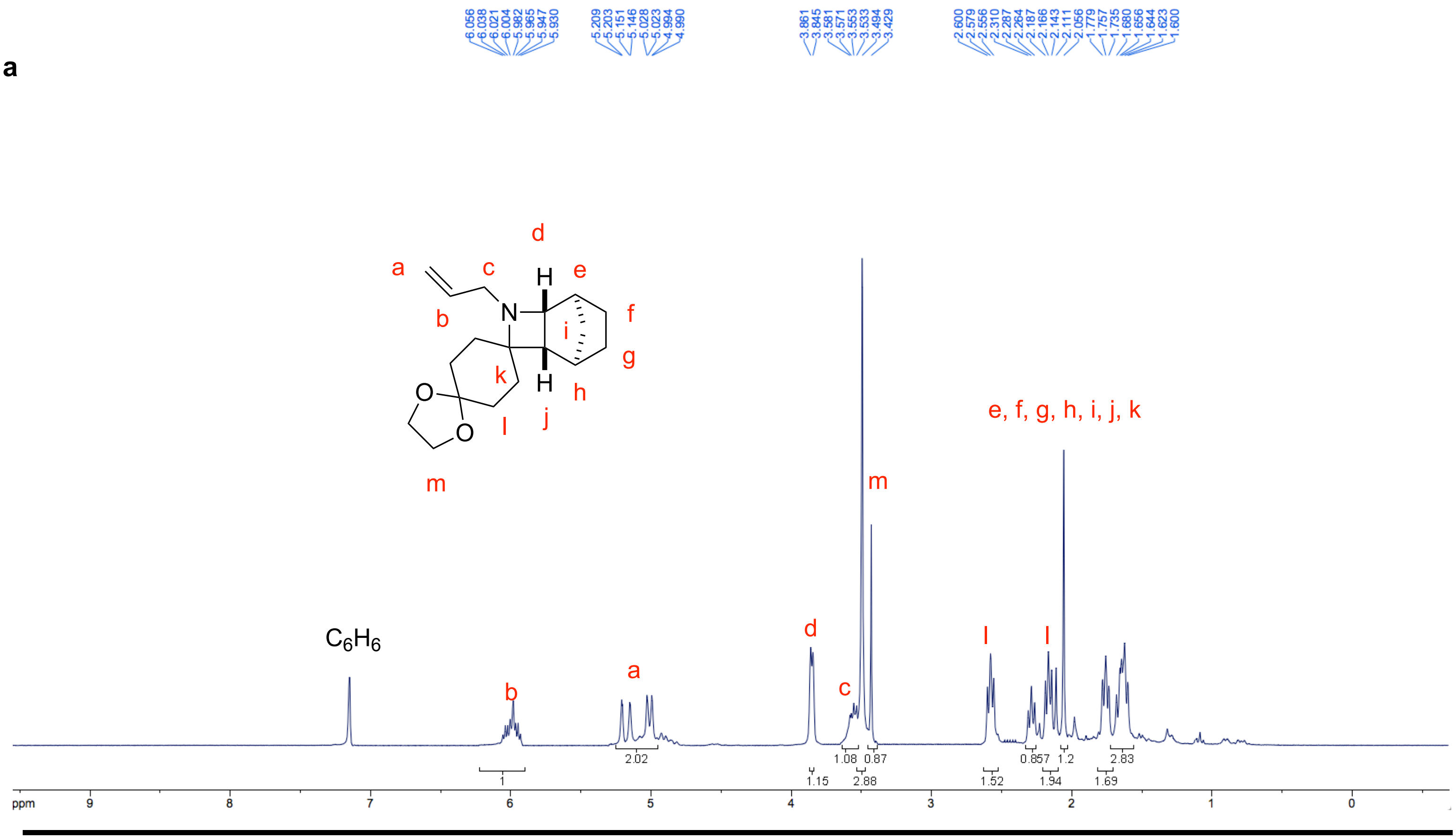

b

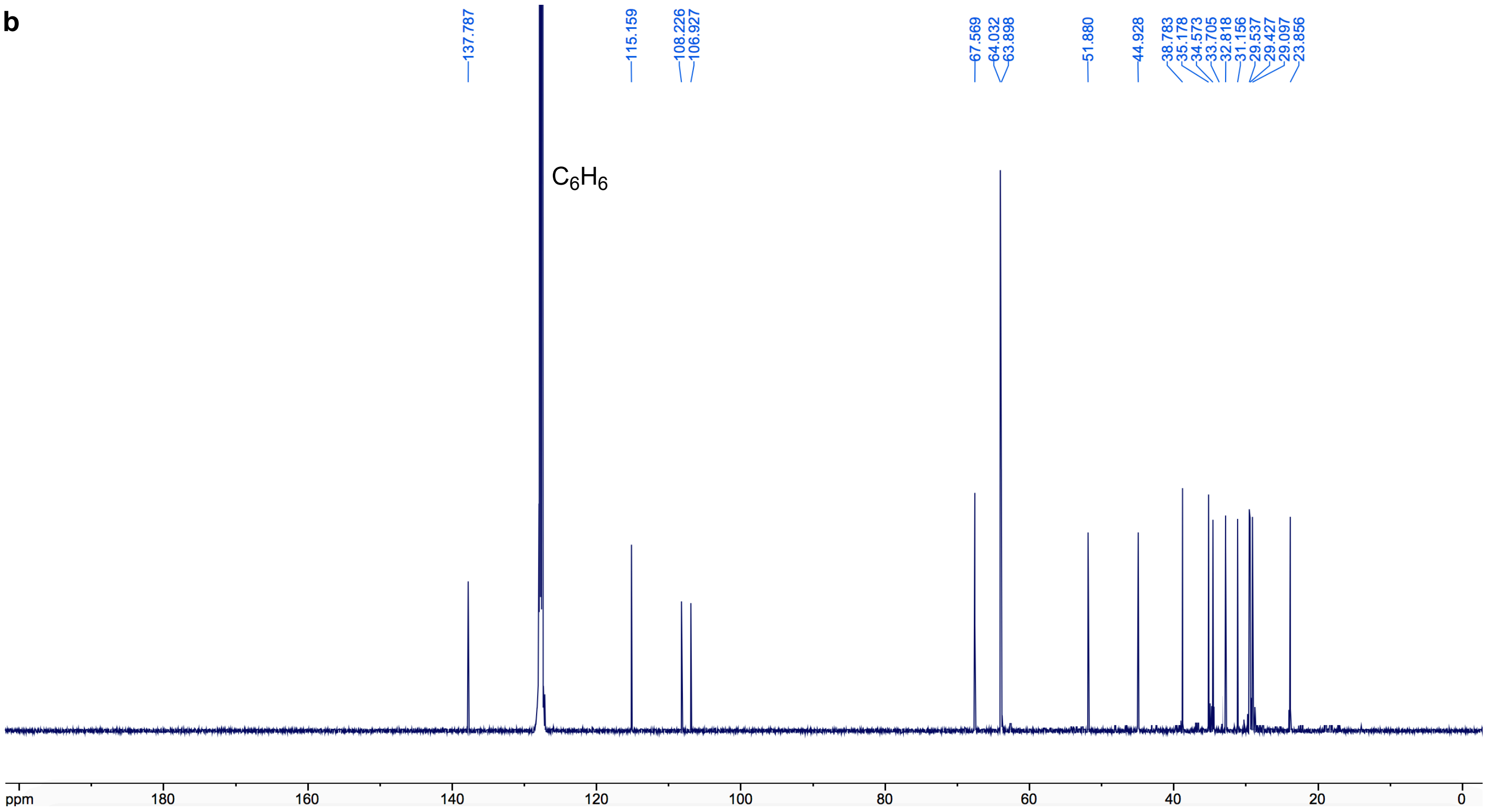

Supplementary Figure 54. NMR spectra of **14**. **a** ^{1}H NMR (300 MHz, C_6D_6). **b** ^{13}C NMR (126 MHz, C_6D_6).

a

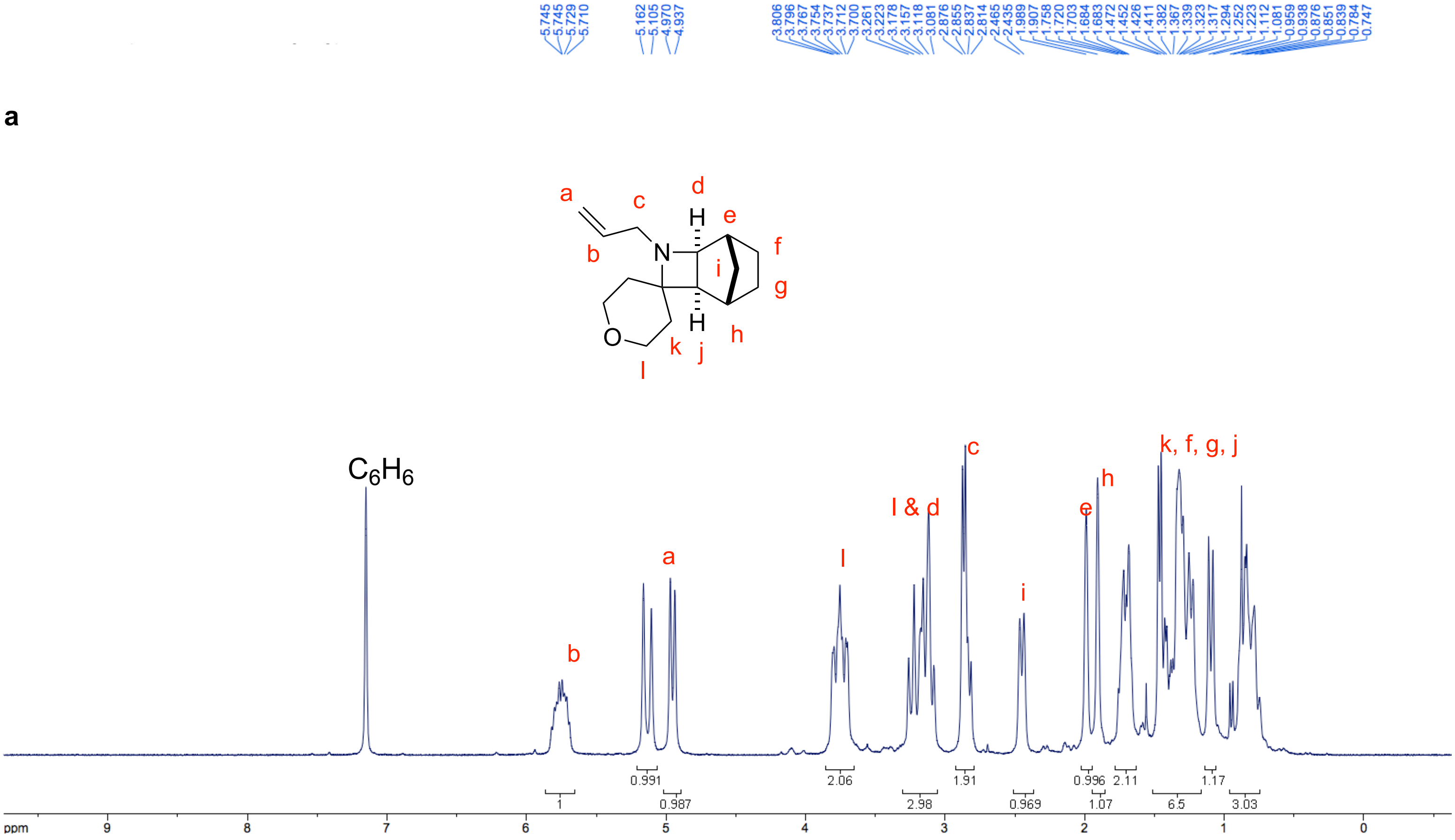

b

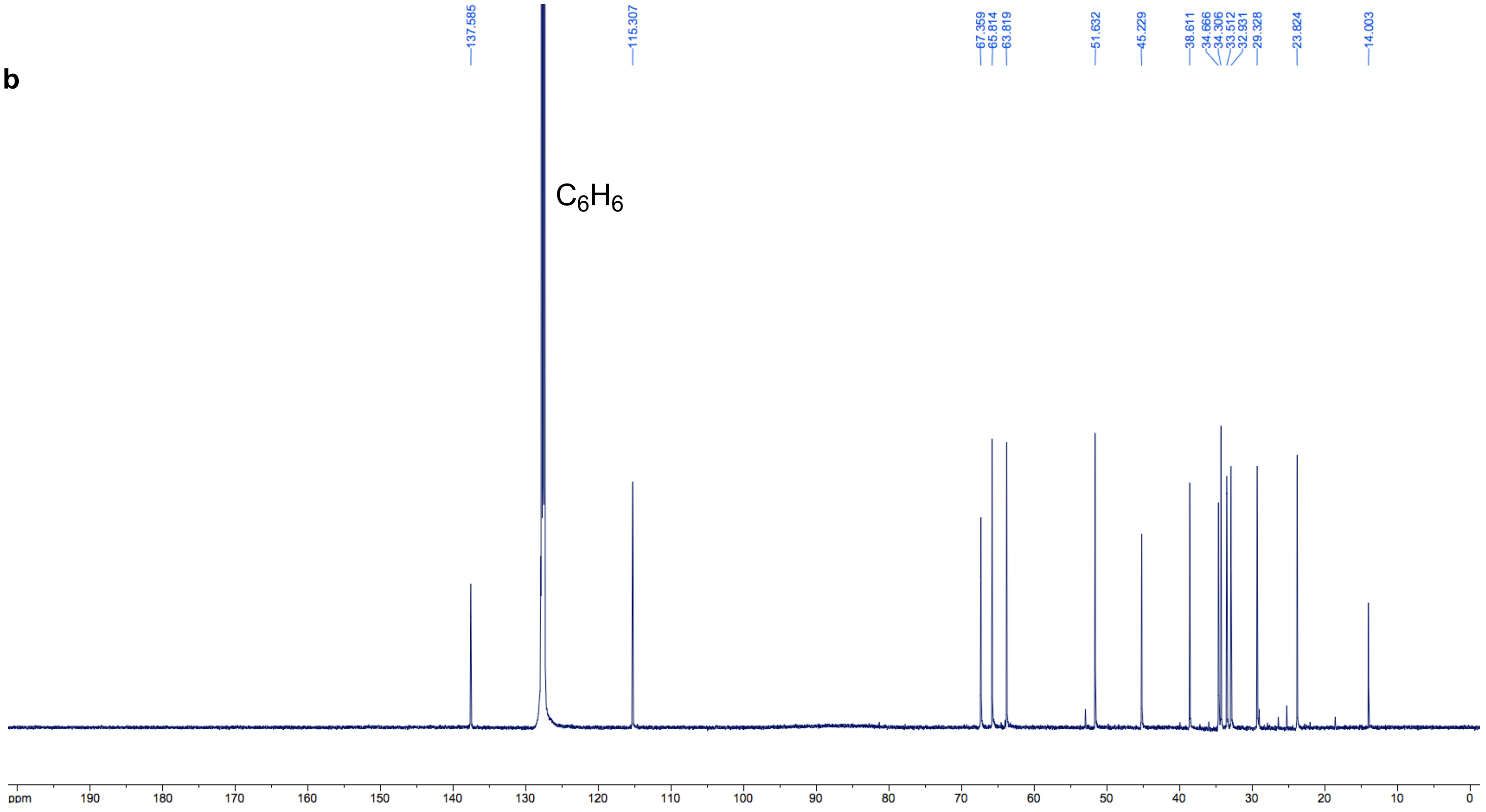

Supplementary Figure 55. NMR spectra of **15**. **a** ^{1}H NMR (300 MHz, C_6D_6). **b** ^{13}C NMR (126 MHz, C_6D_6).

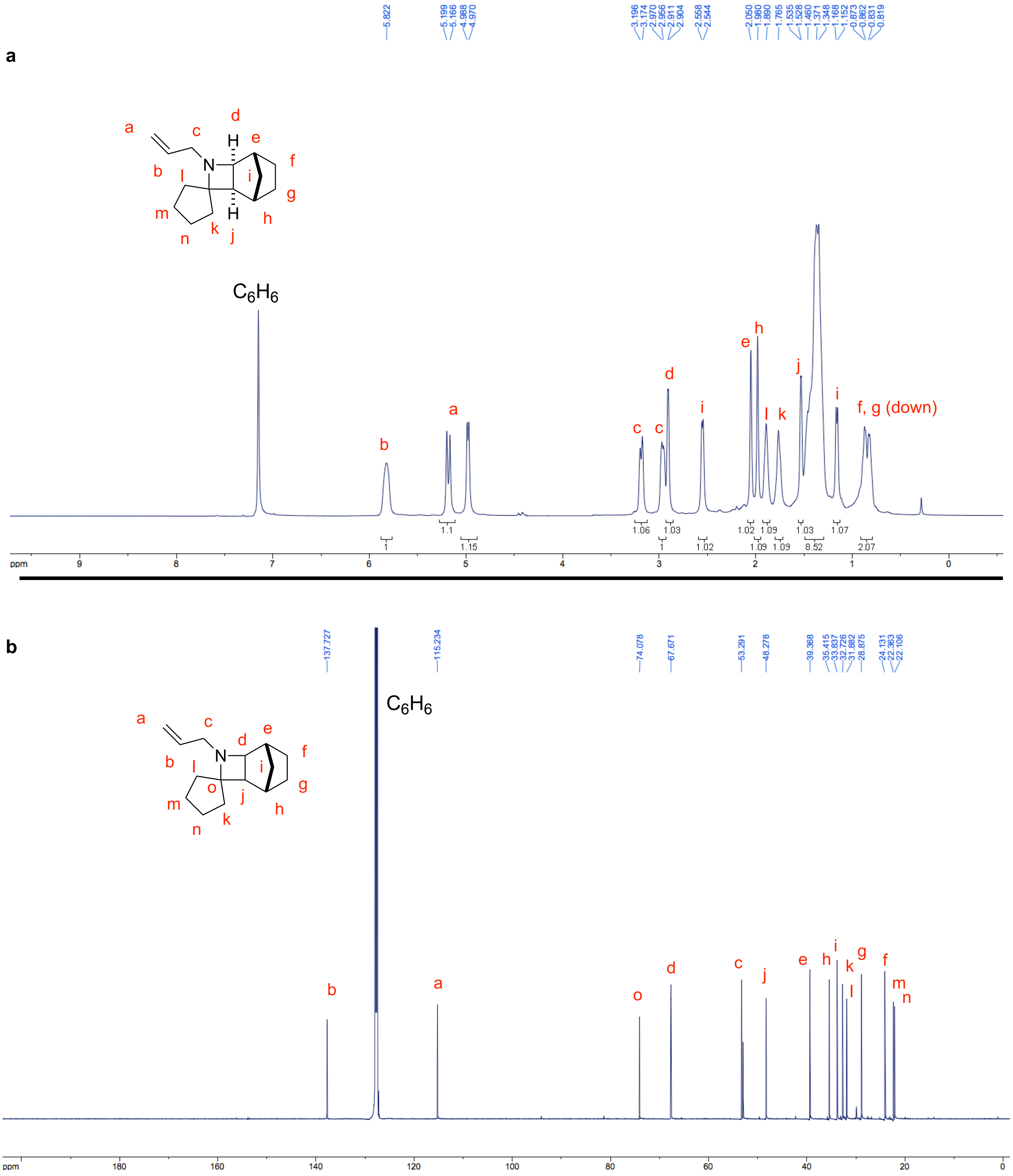

Supplementary Figure 56. NMR spectra of **16**. **a** ^{1}H NMR (300 MHz, C_6D_6). **b** ^{13}C NMR (126 MHz, C_6D_6).

a

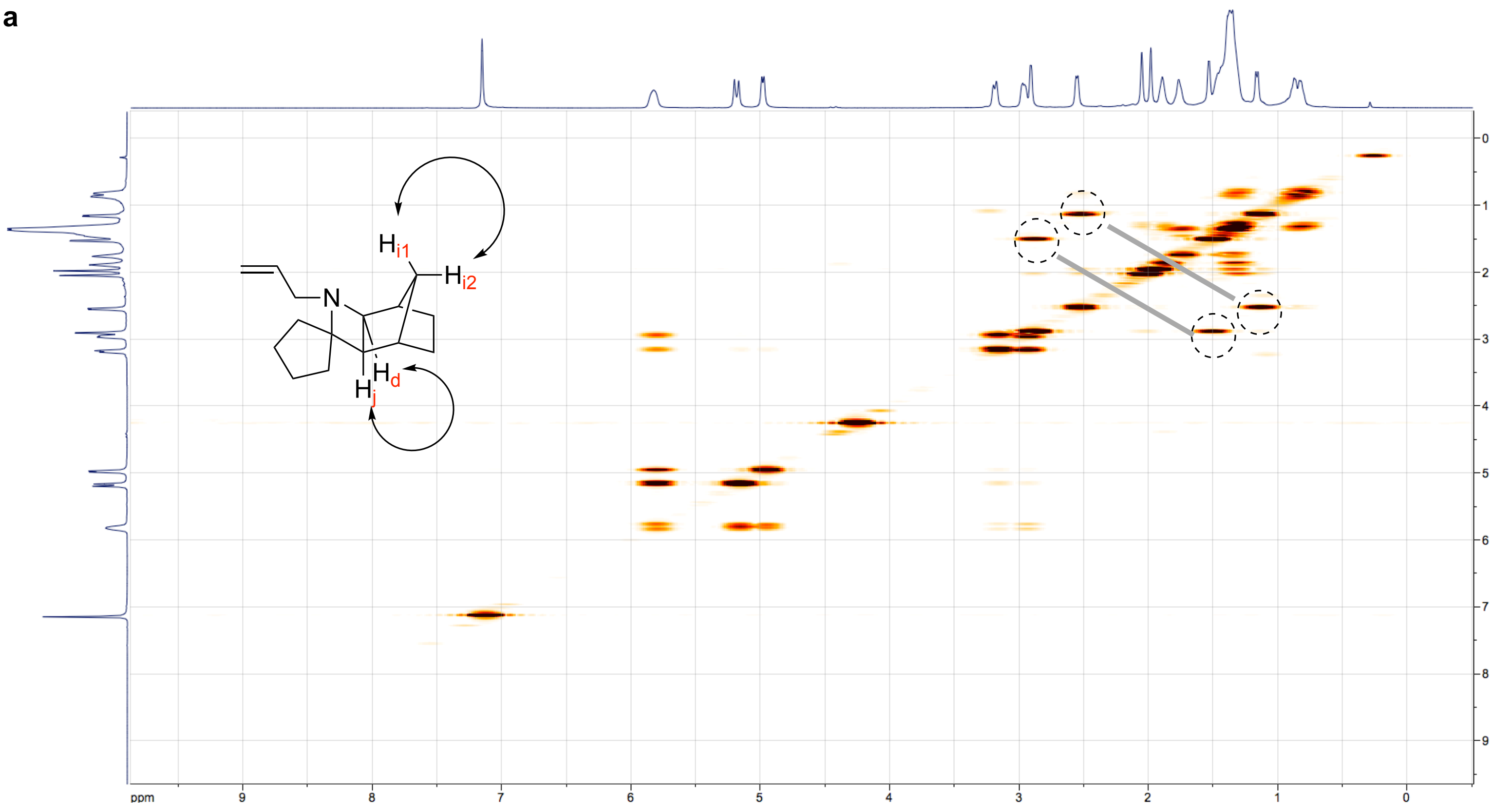

b

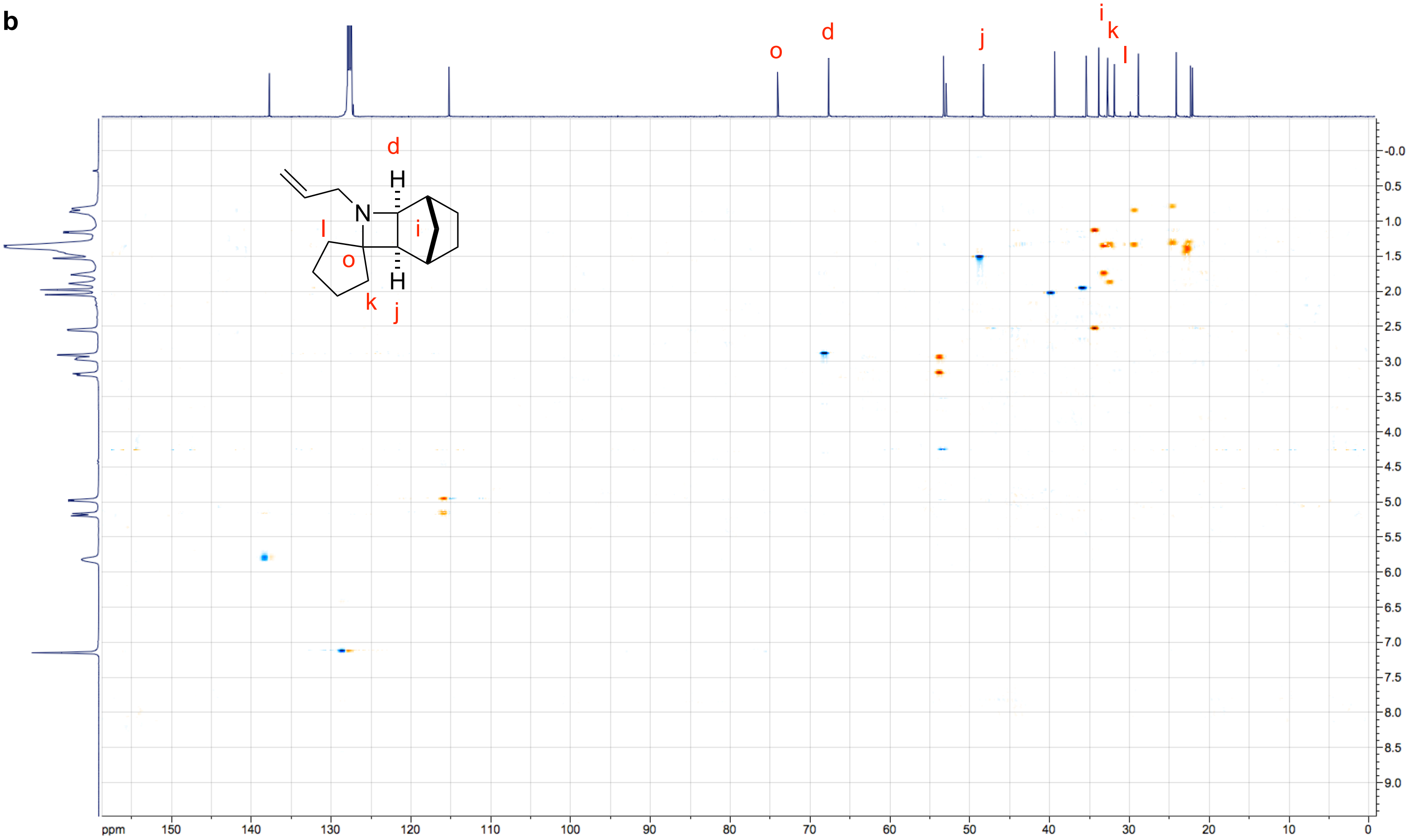

Supplementary Figure 57. NMR spectra of **16**. **a** COSY NMR (^{1}H-^{1}H, 500 MHz, C_6D_6). **b** HSQC NMR (^{13}C-^{1}H, 500 MHz, C_6D_6).

a

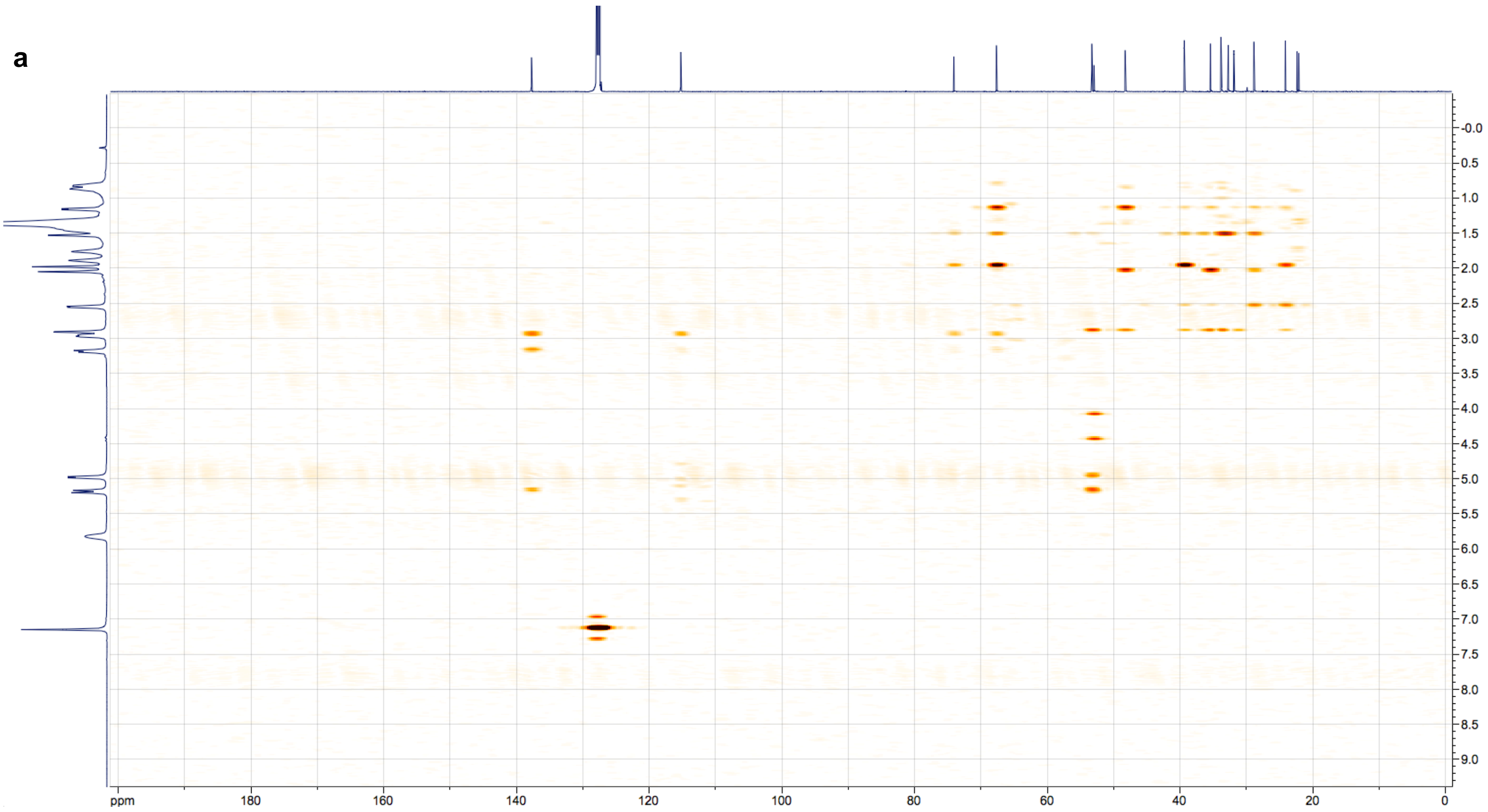

b

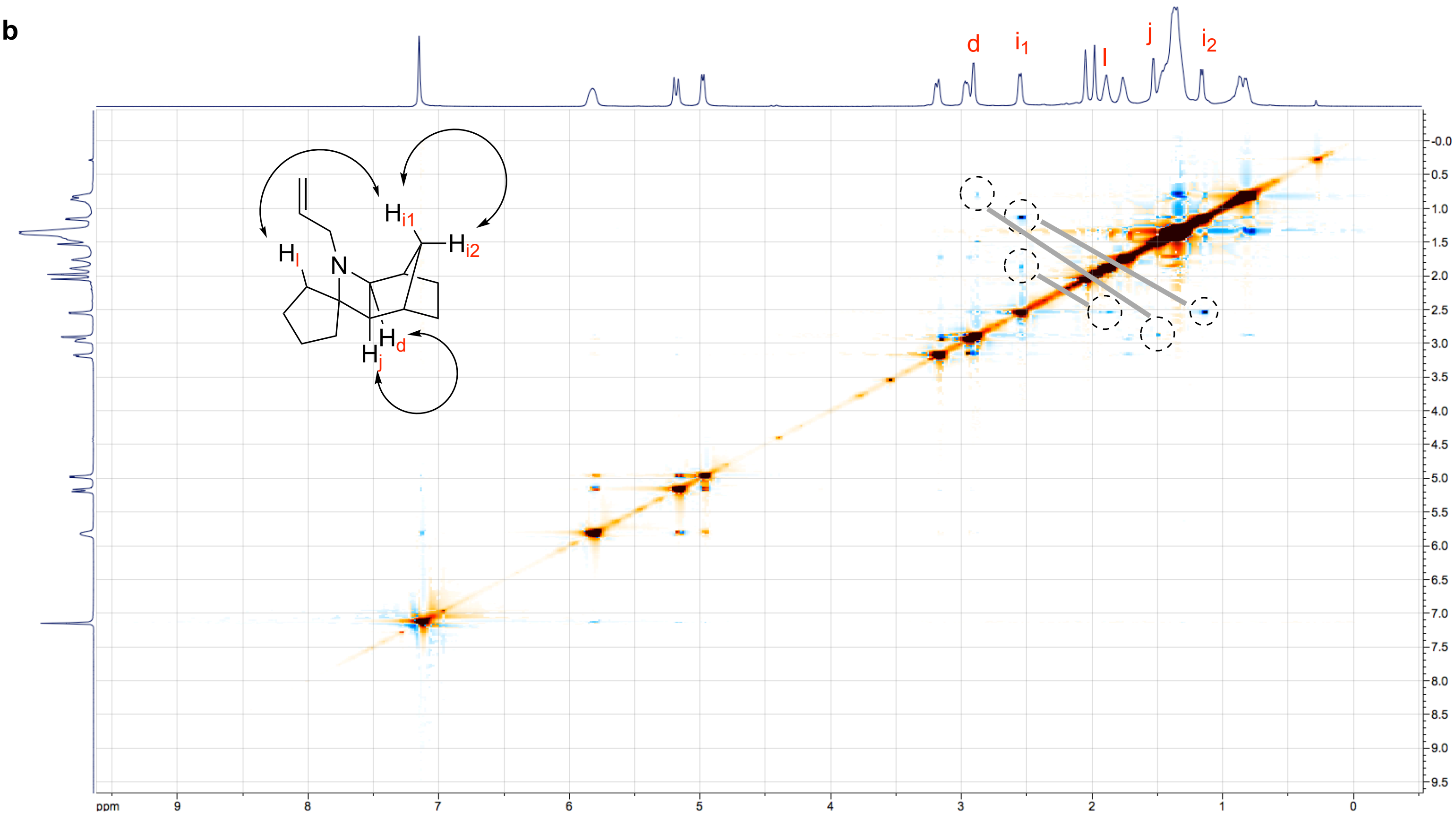

Supplementary Figure 58. NMR spectra of **16**. **a** HMBC NMR (^{13}C-^{1}H, 500 MHz, C_6D_6). **b** NOSEY NMR (^{1}H-^{1}H, 500 MHz, C_6D_6).

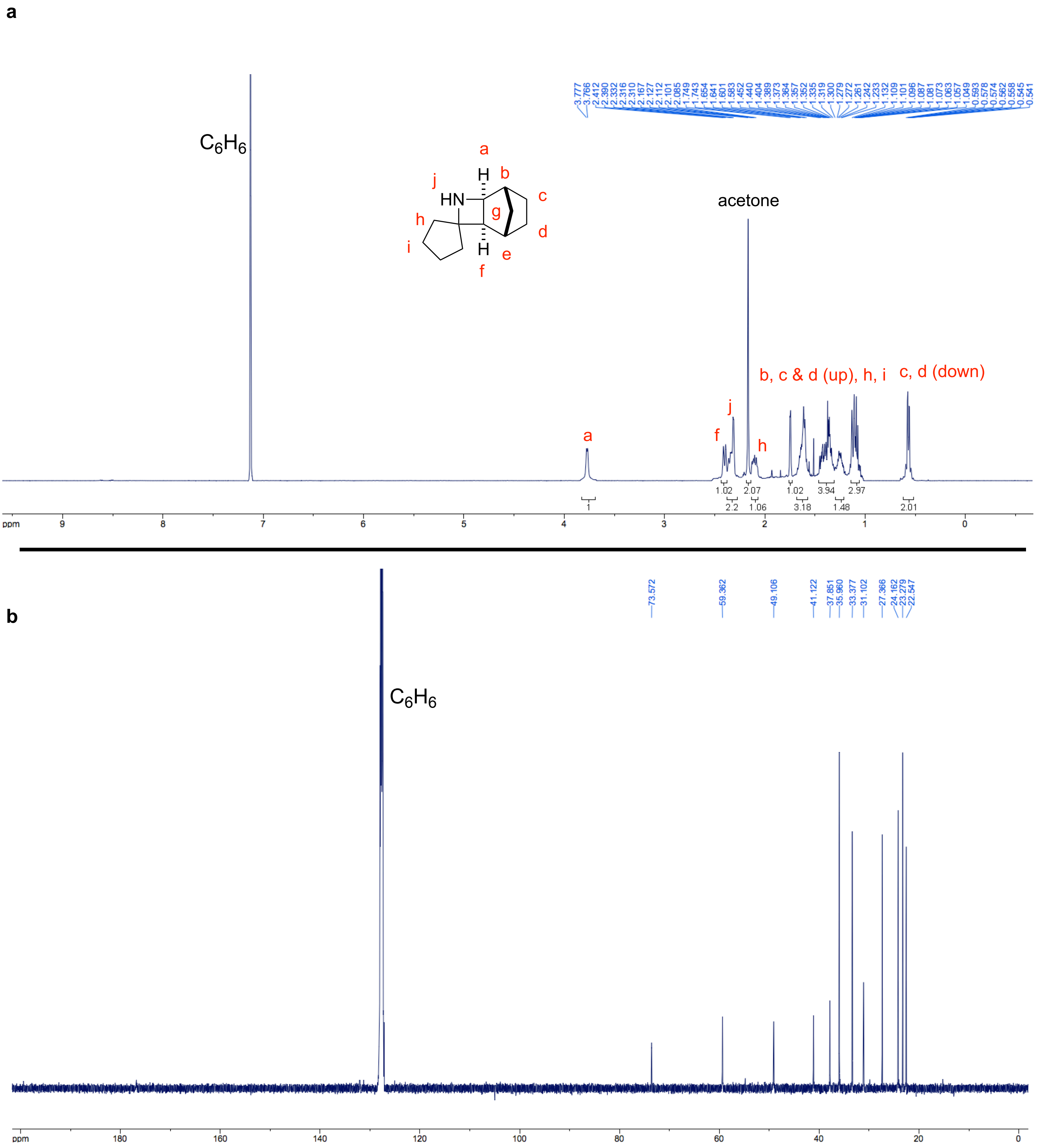

Supplementary Figure 59. NMR spectra of **16a**. **a** ^{1}H NMR (300 MHz, C_6D_6). **b** ^{13}C NMR (126 MHz, C_6D_6).

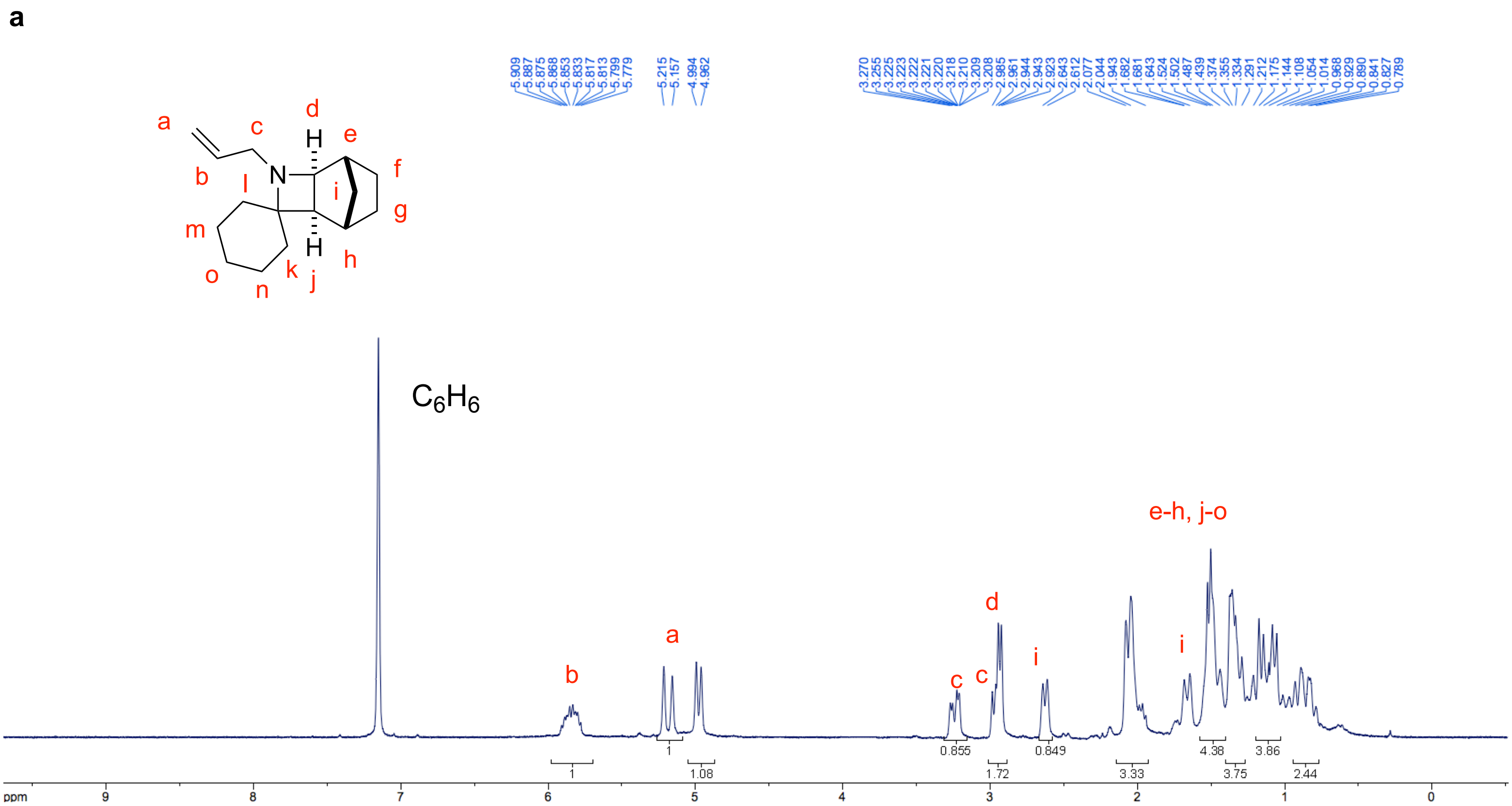

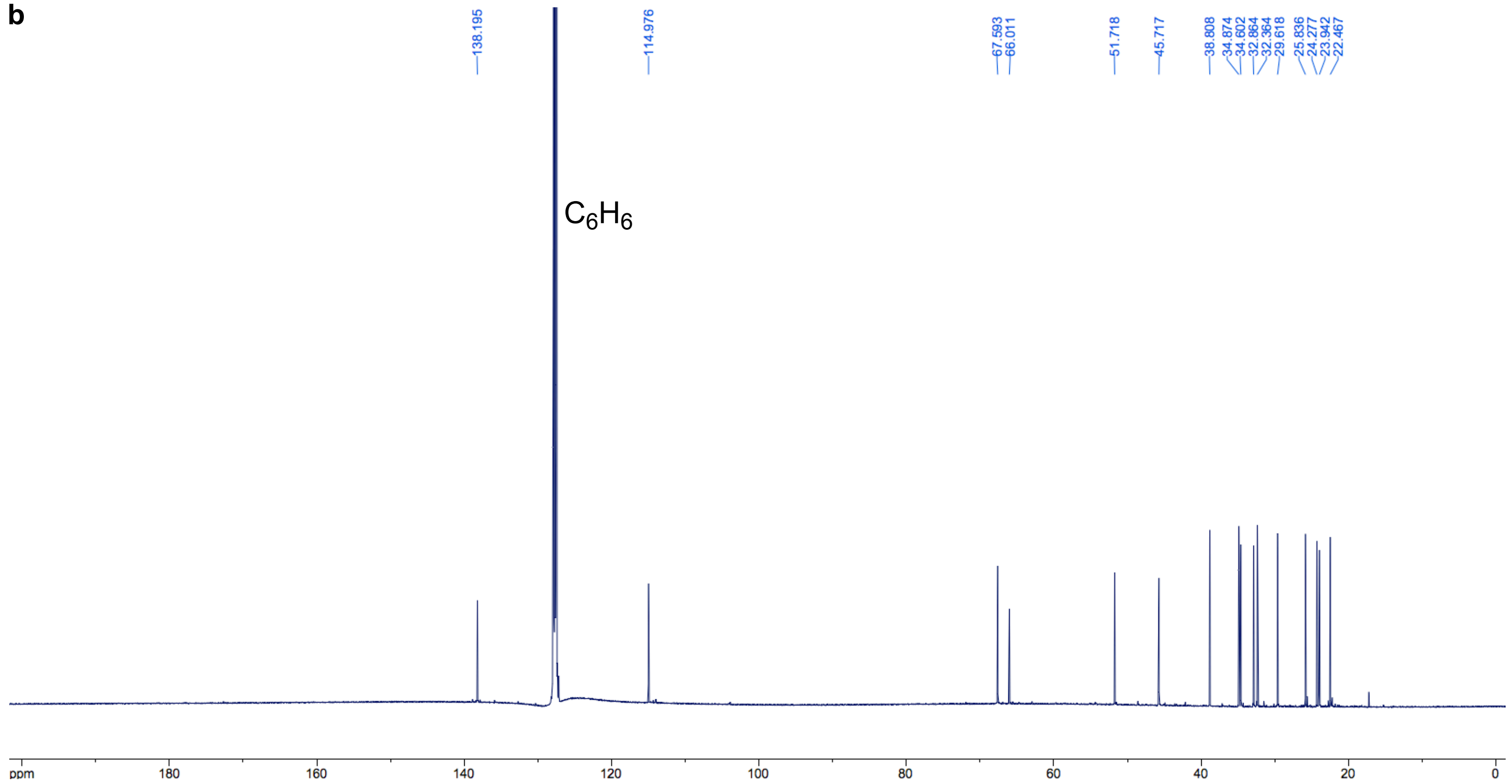

Supplementary Figure 60. NMR spectra of **17**. **a** ^{1}H NMR (300 MHz, C_6D_6). **b** ^{13}C NMR (126 MHz, C_6D_6).

a

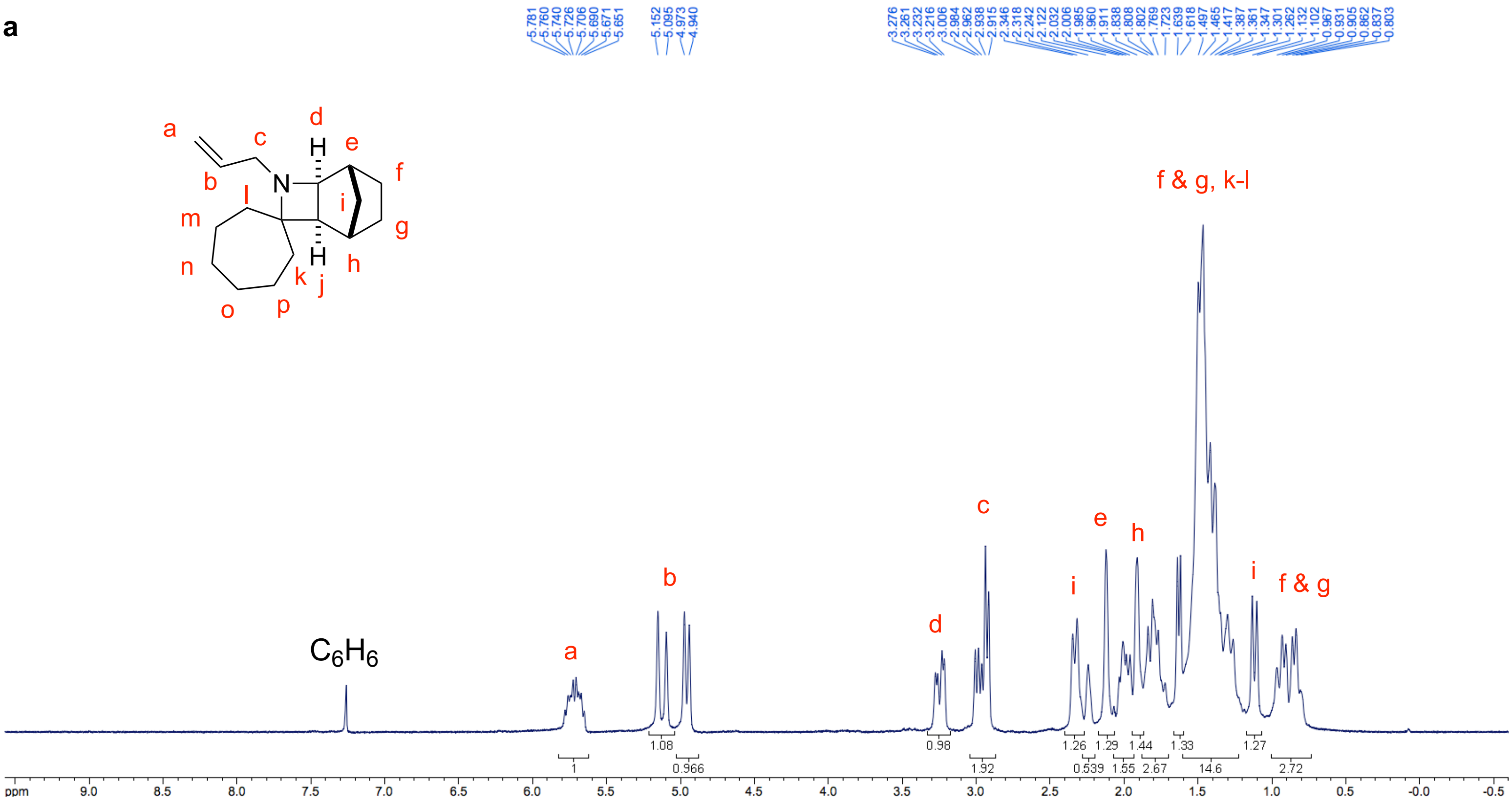

b

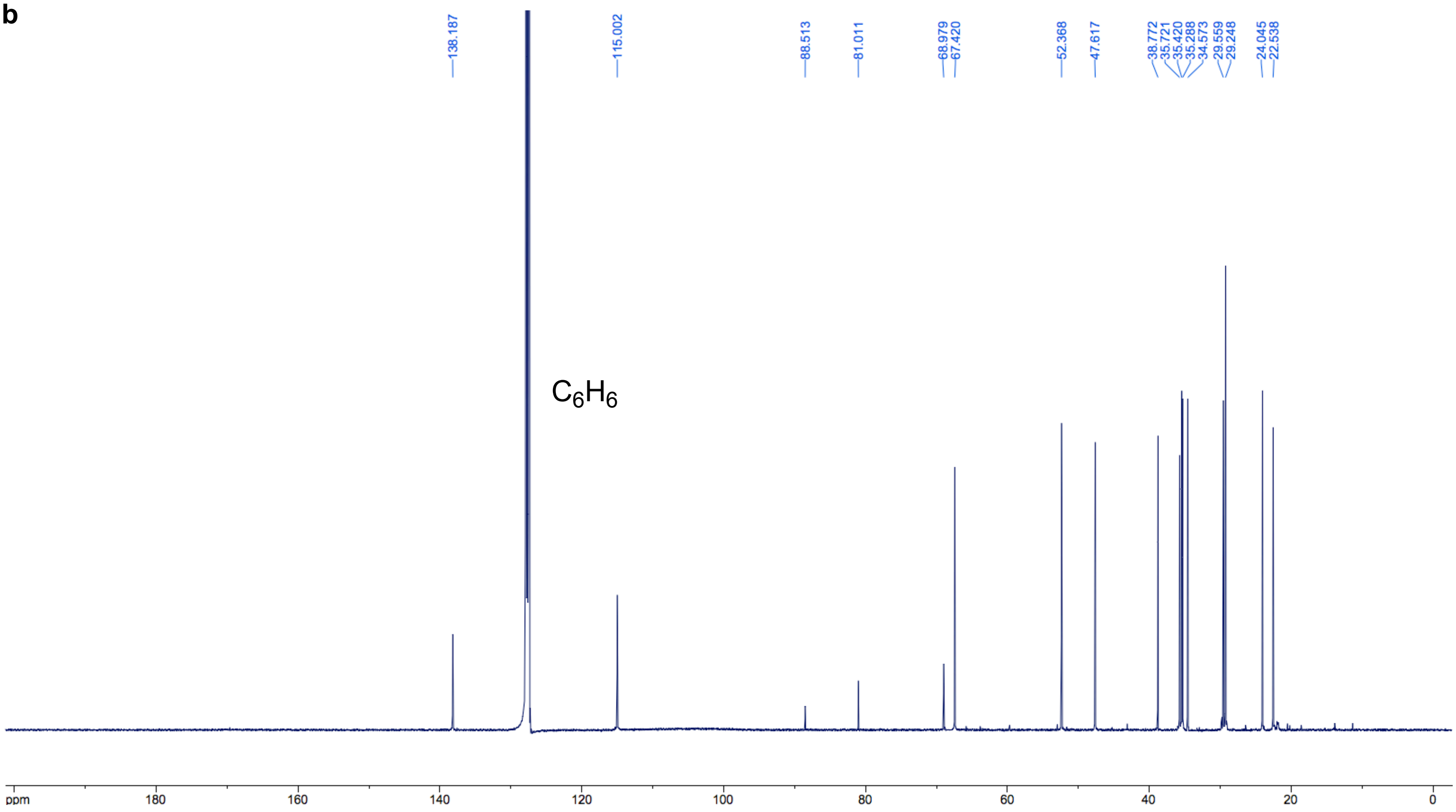

Supplementary Figure 61. NMR spectra of **18**. **a** ^{1}H NMR (300 MHz, C_6D_6). **b** ^{13}C NMR (126 MHz, C_6D_6).

a

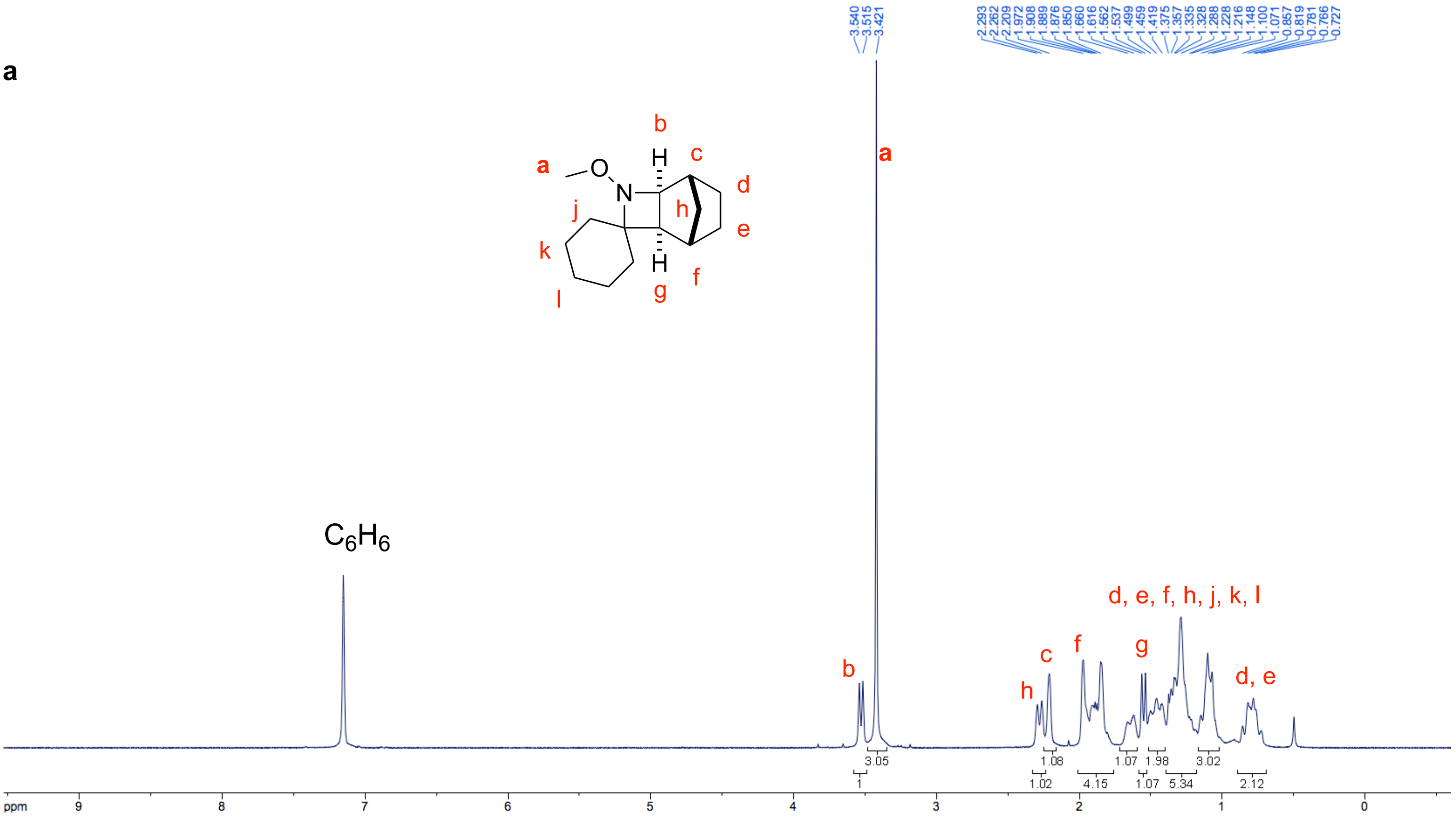

b

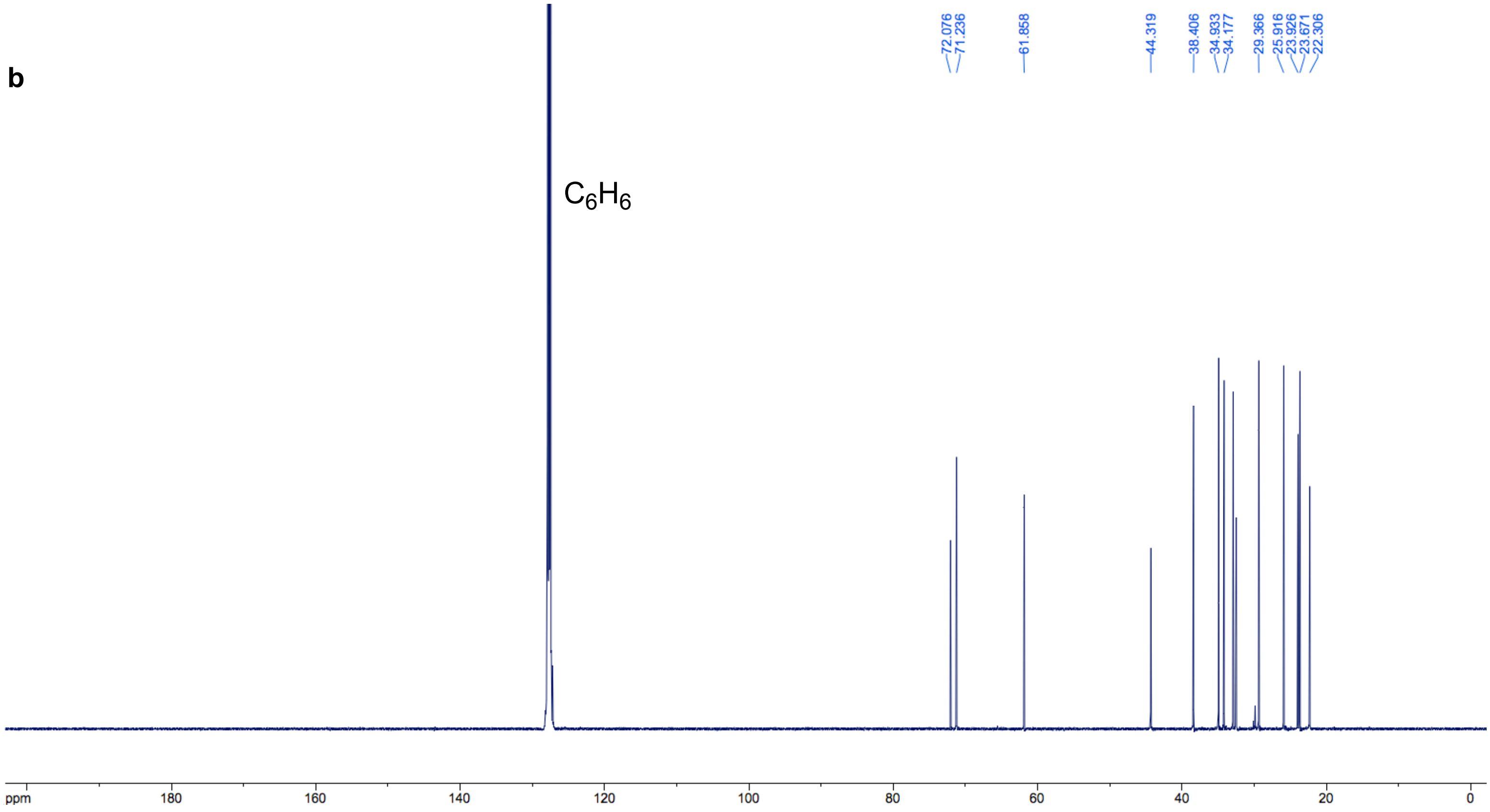

Supplementary Figure 62. NMR spectra of **19**. **a** ^{1}H NMR (300 MHz, C_6D_6). **b** ^{13}C NMR (126 MHz, C_6D_6).

a

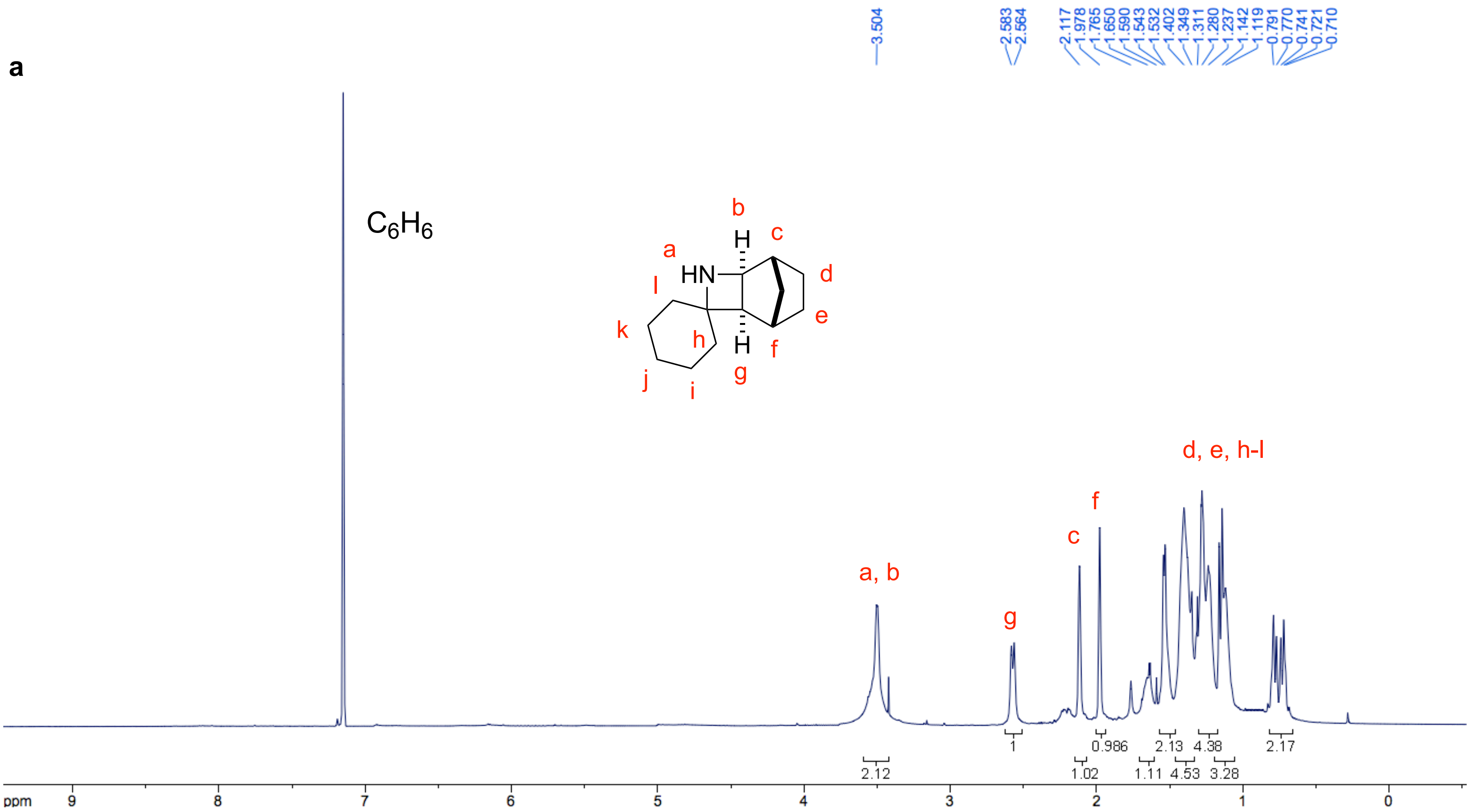

b

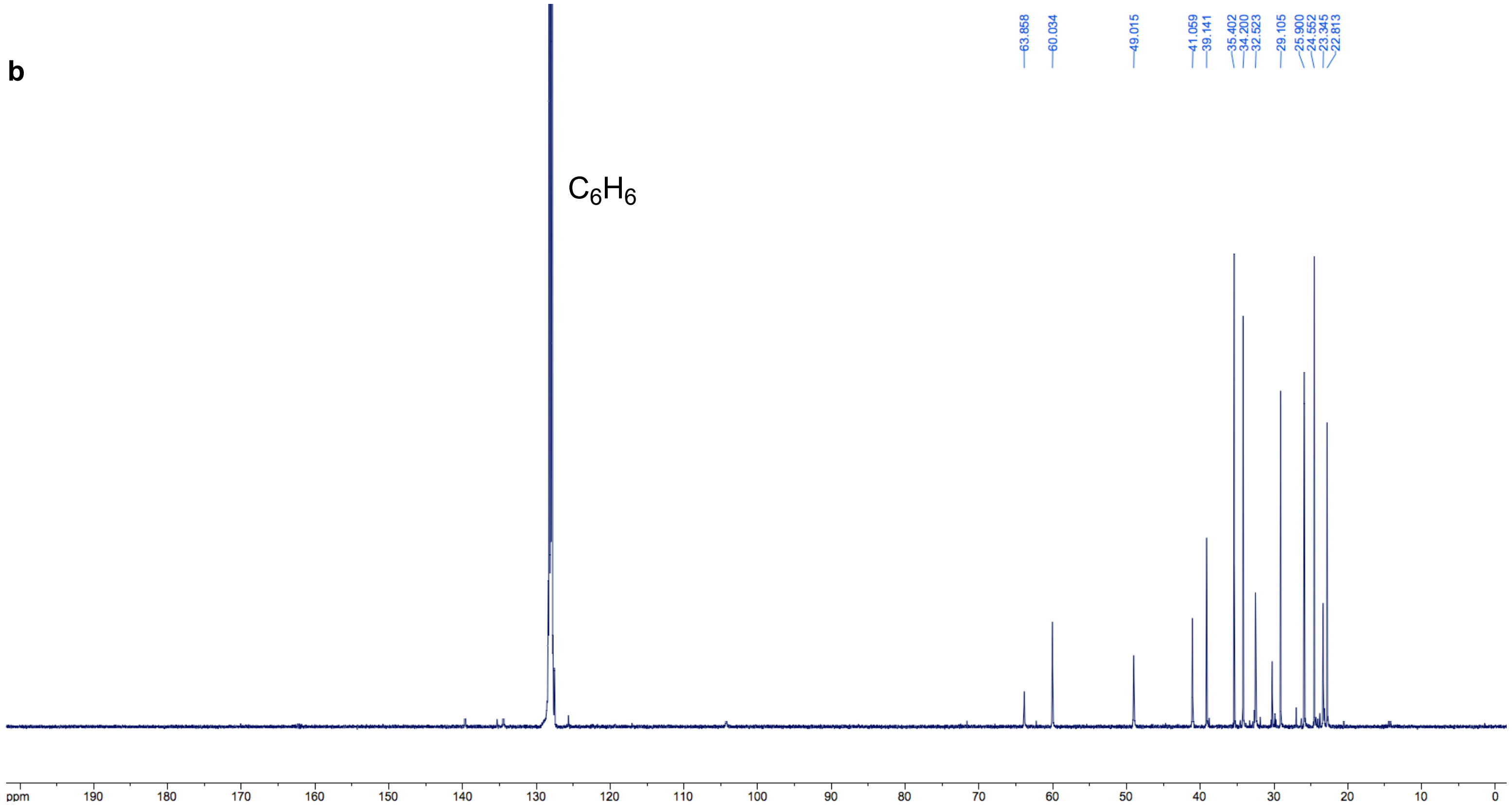

Supplementary Figure 63. NMR spectra of **19a**. **a** ^{1}H NMR (300 MHz, C_6D_6). **b** ^{13}C NMR (126 MHz, C_6D_6).

a

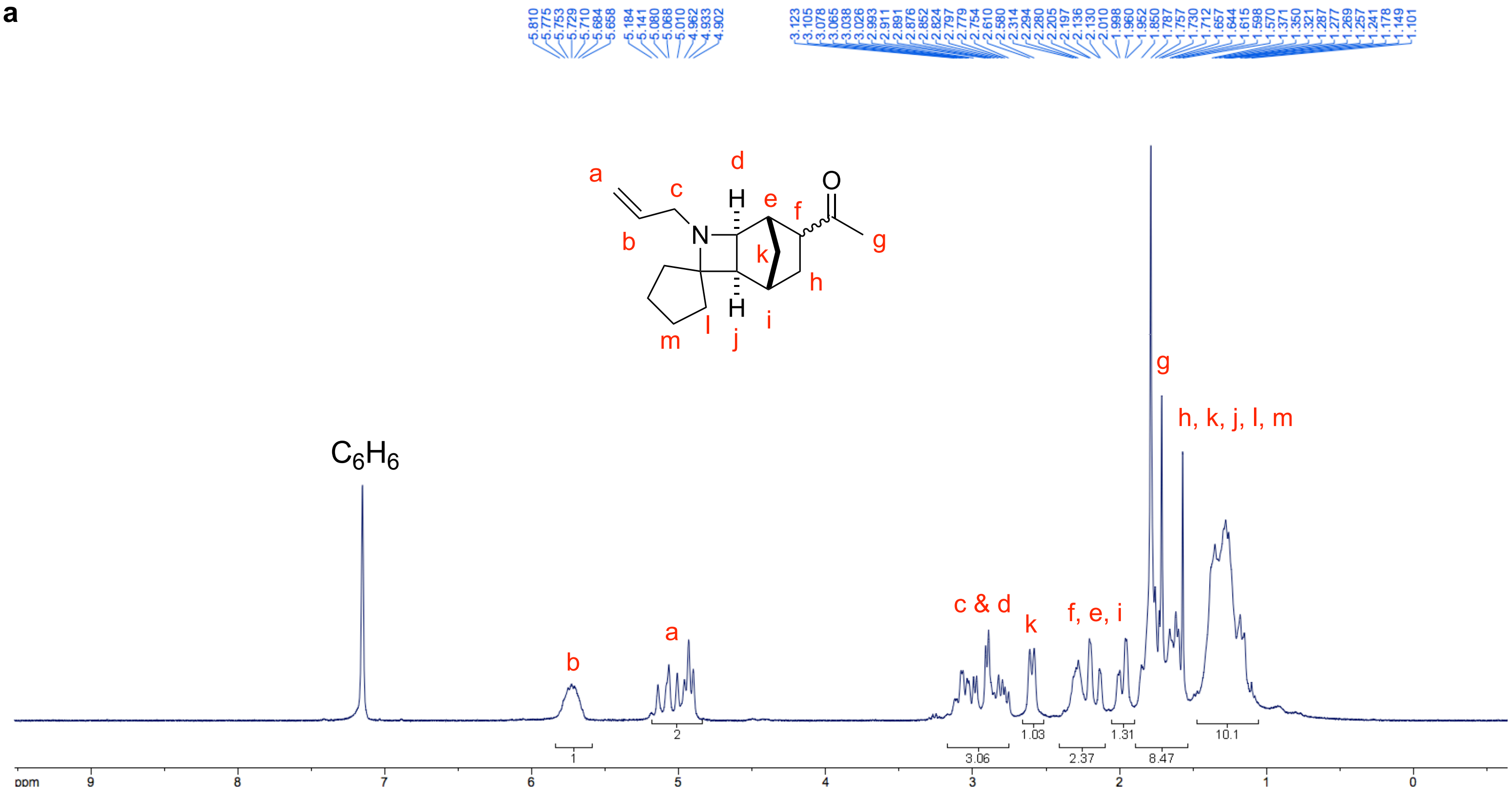

b

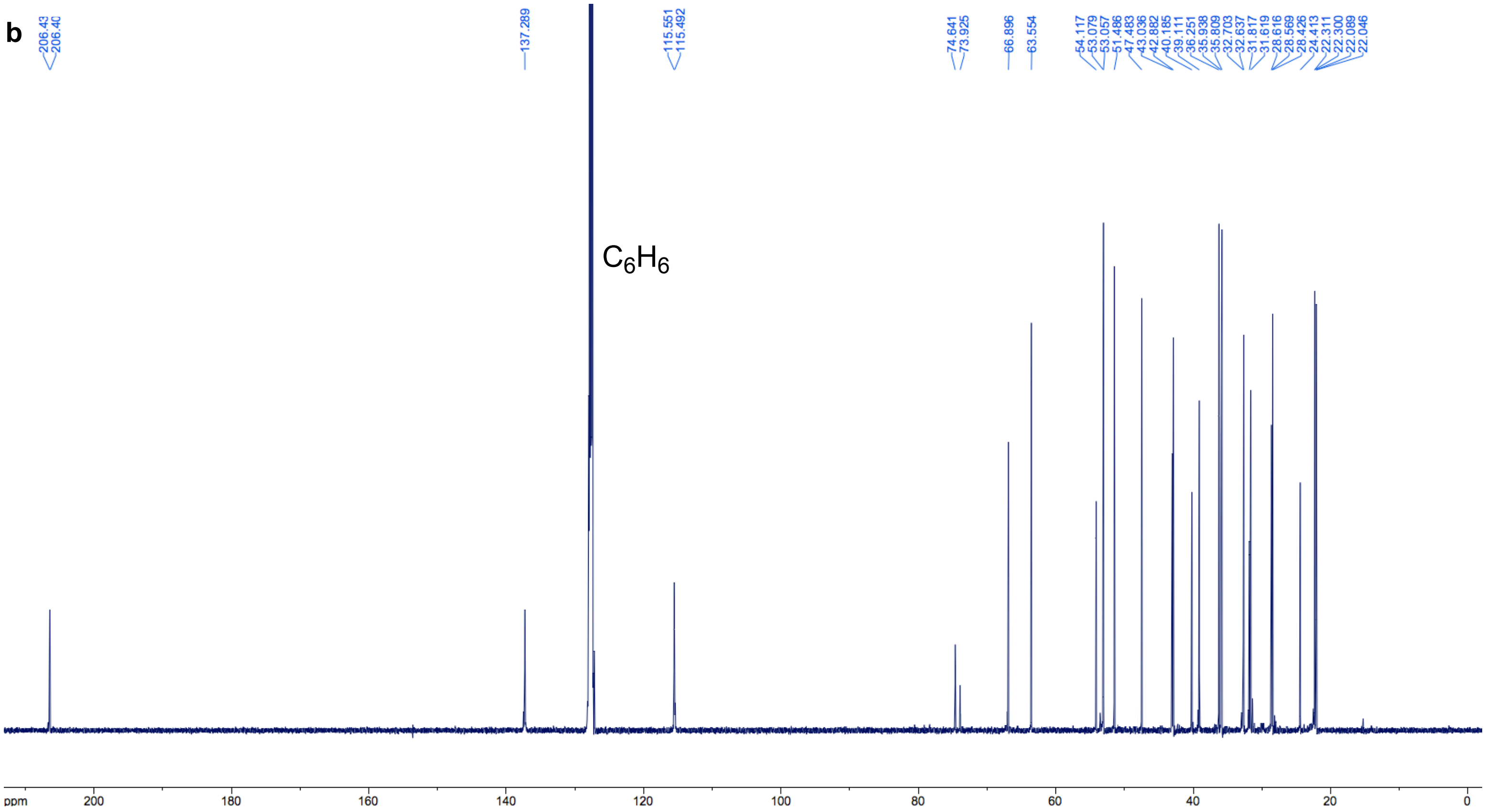

Supplementary Figure 64. NMR spectra of **20**. **a** ^{1}H NMR (300 MHz, C_6D_6). **b** ^{13}C NMR (126 MHz, C_6D_6).

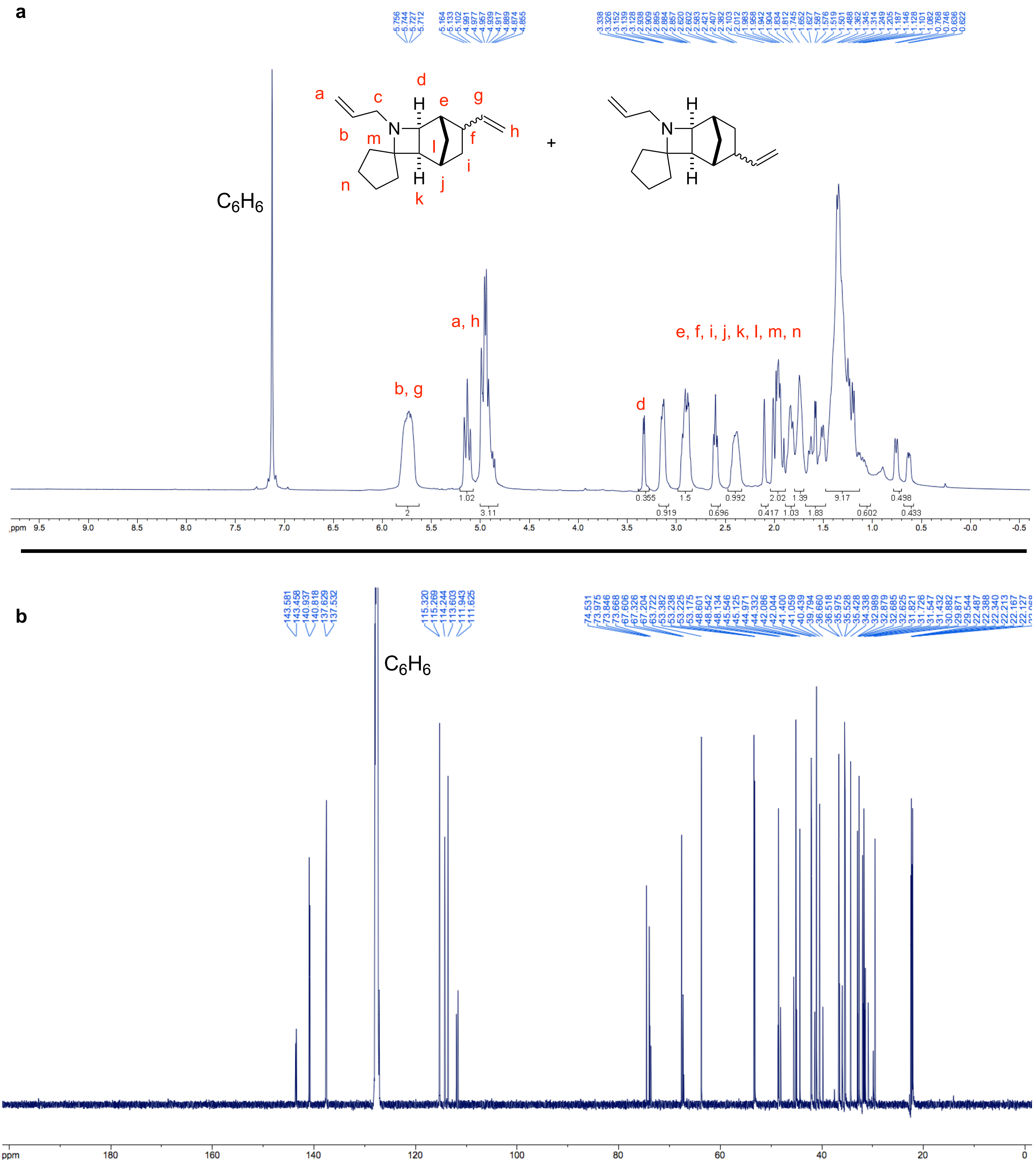

Supplementary Figure 65. NMR spectra of **21**. **a** ^{1}H NMR (300 MHz, C_6D_6). **b** ^{13}C NMR (126 MHz, C_6D_6).

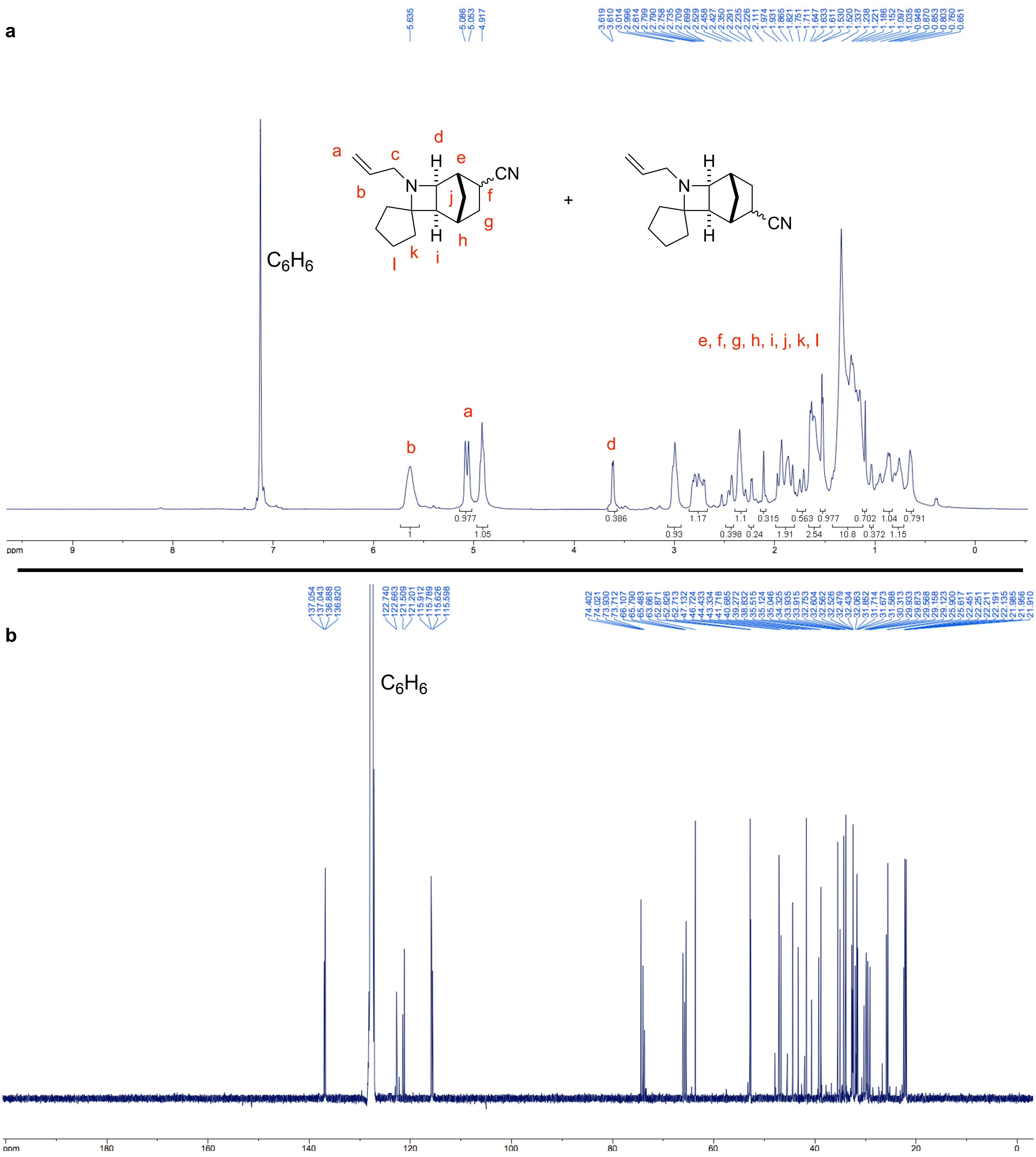

Supplementary Figure 66. NMR spectra of **22**. **a** ^{1}H NMR (300 MHz, C_6D_6). **b** ^{13}C NMR (126 MHz, C_6D_6).

a

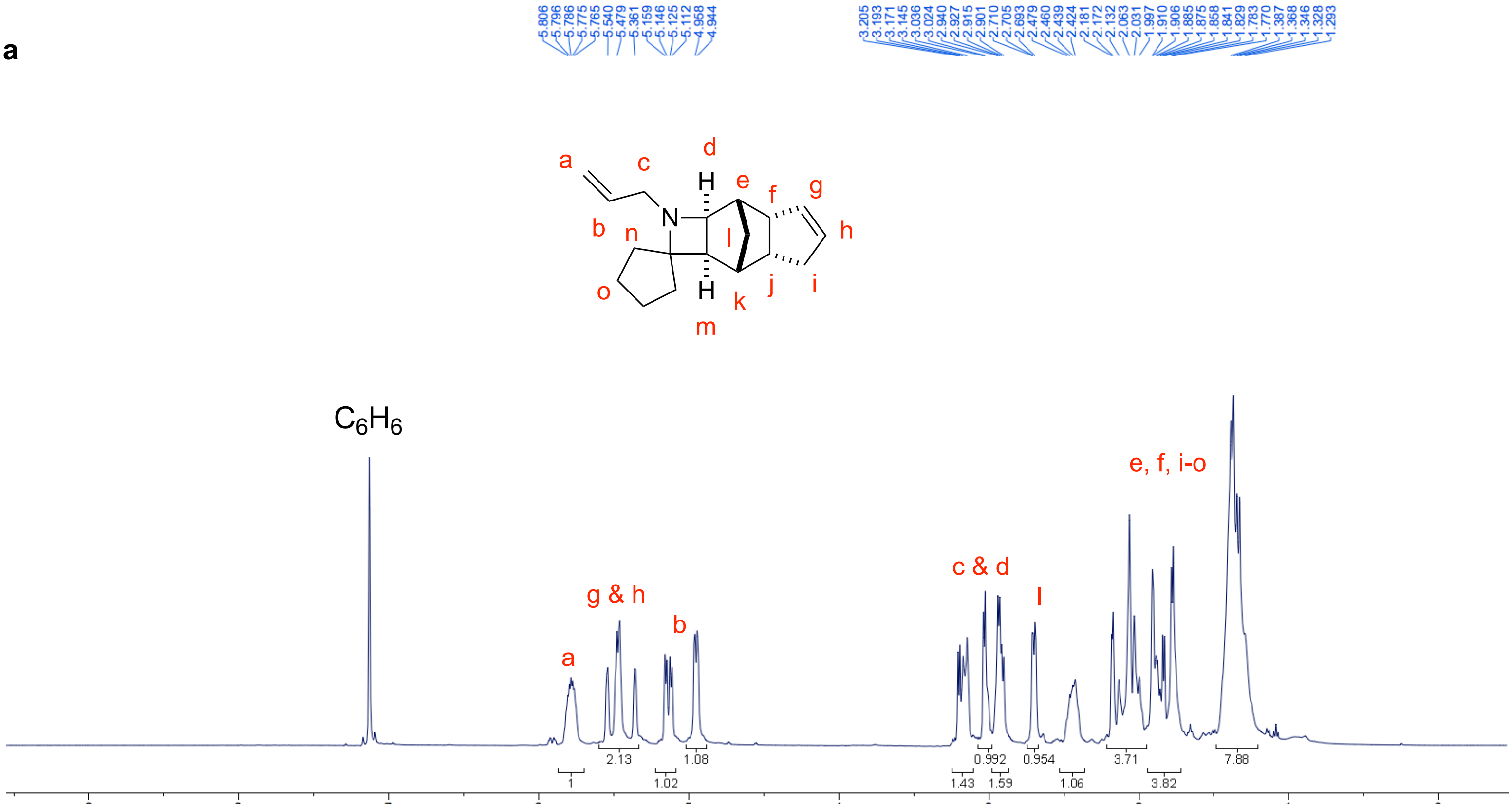

b

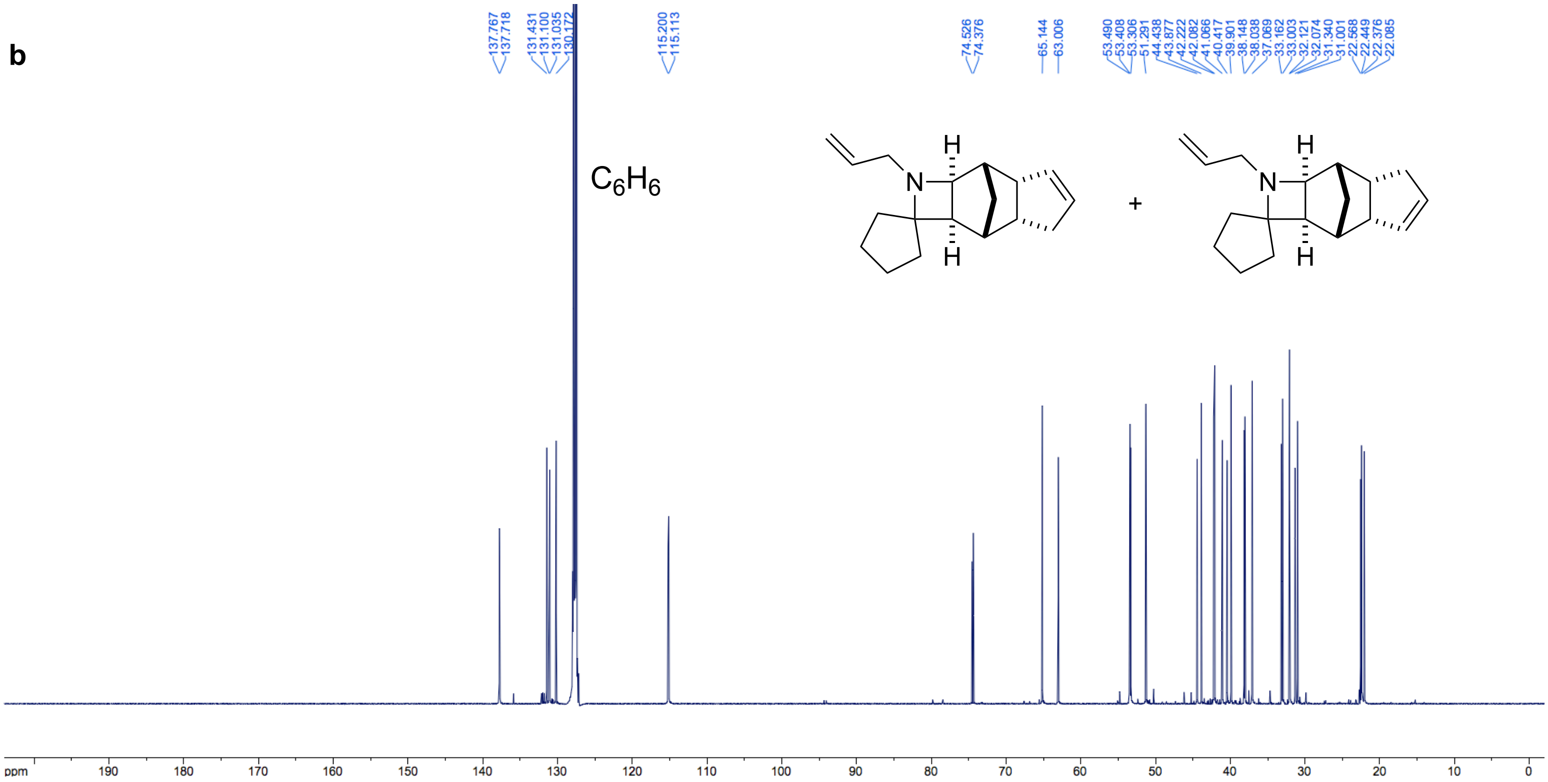

Supplementary Figure 67. NMR spectra of **23**. **a** ^1H NMR (300 MHz, C_6D_6). **b** ^{13}C NMR (126 MHz, C_6D_6).

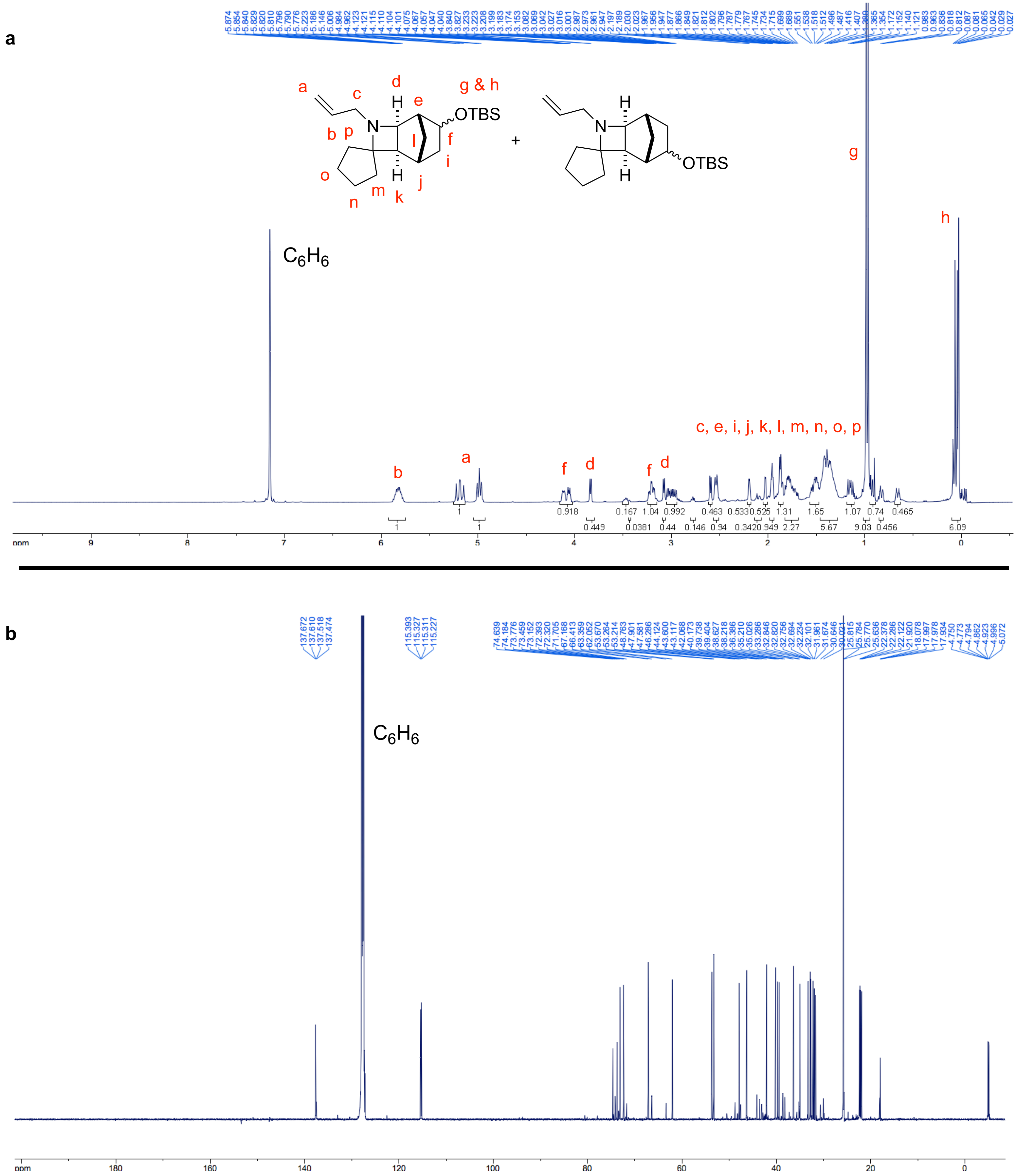

Supplementary Figure 68. NMR spectra of **24**. **a** ^{1}H NMR (300 MHz, C_6D_6). **b** ^{13}C NMR (126 MHz, C_6D_6).

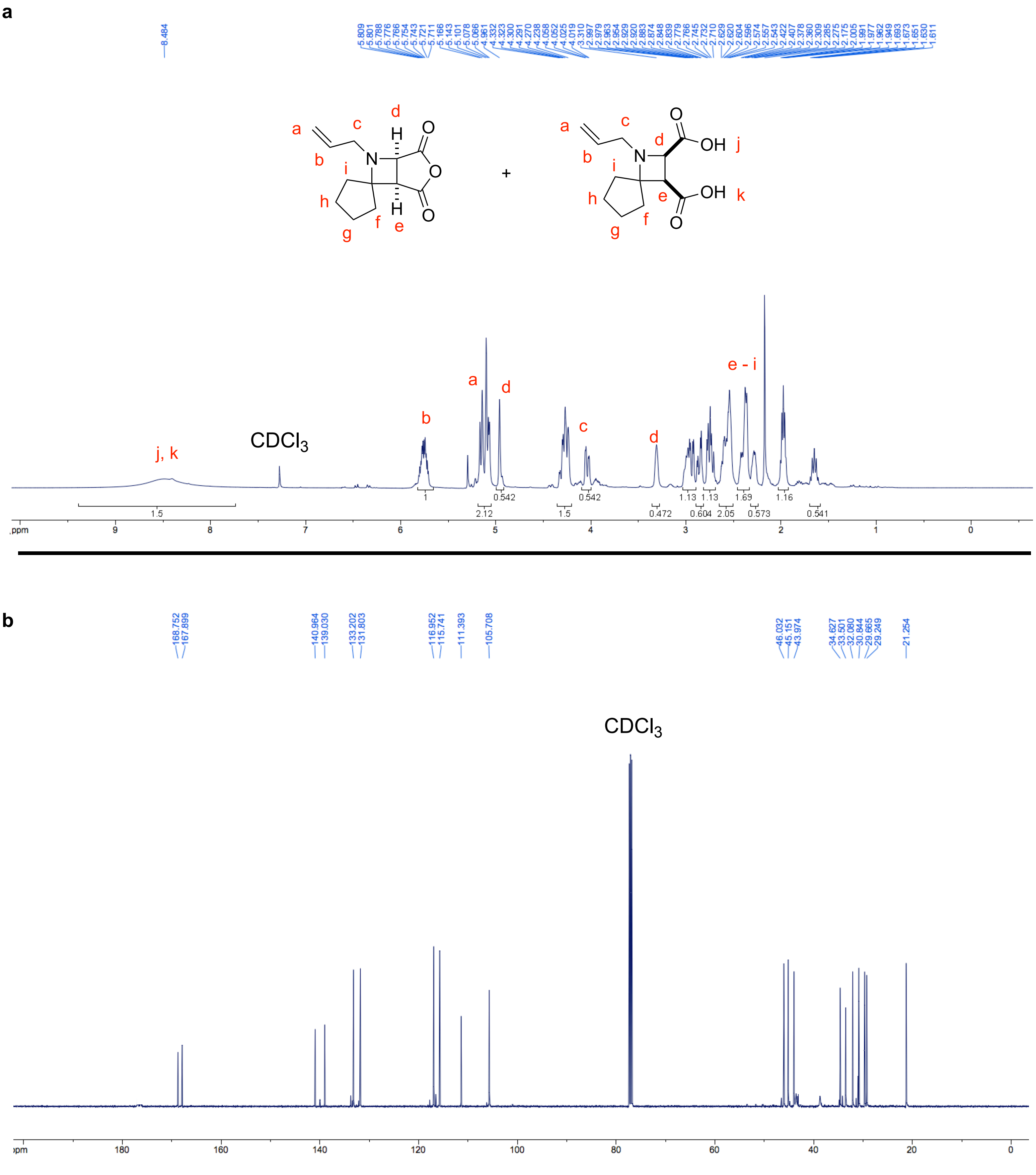

Supplementary Figure 69. NMR spectra of **25**. **a** ^{1}H NMR (300 MHz, C_6D_6). **b** ^{13}C NMR (126 MHz, C_6D_6).

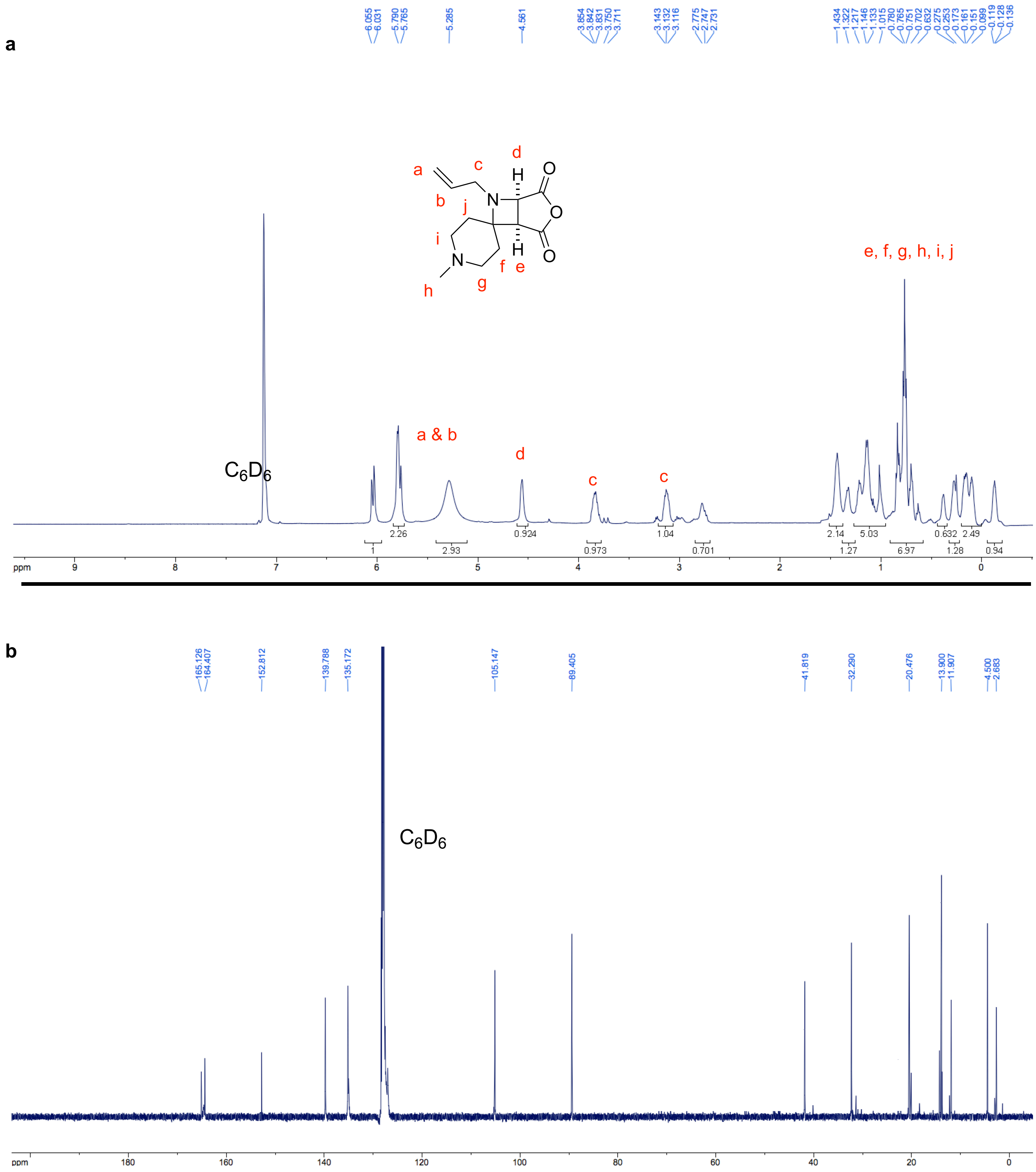

Supplementary Figure 70. NMR spectra of **26**. **a** ^{1}H NMR (300 MHz, C_6D_6). **b** ^{13}C NMR (126 MHz, C_6D_6).

a

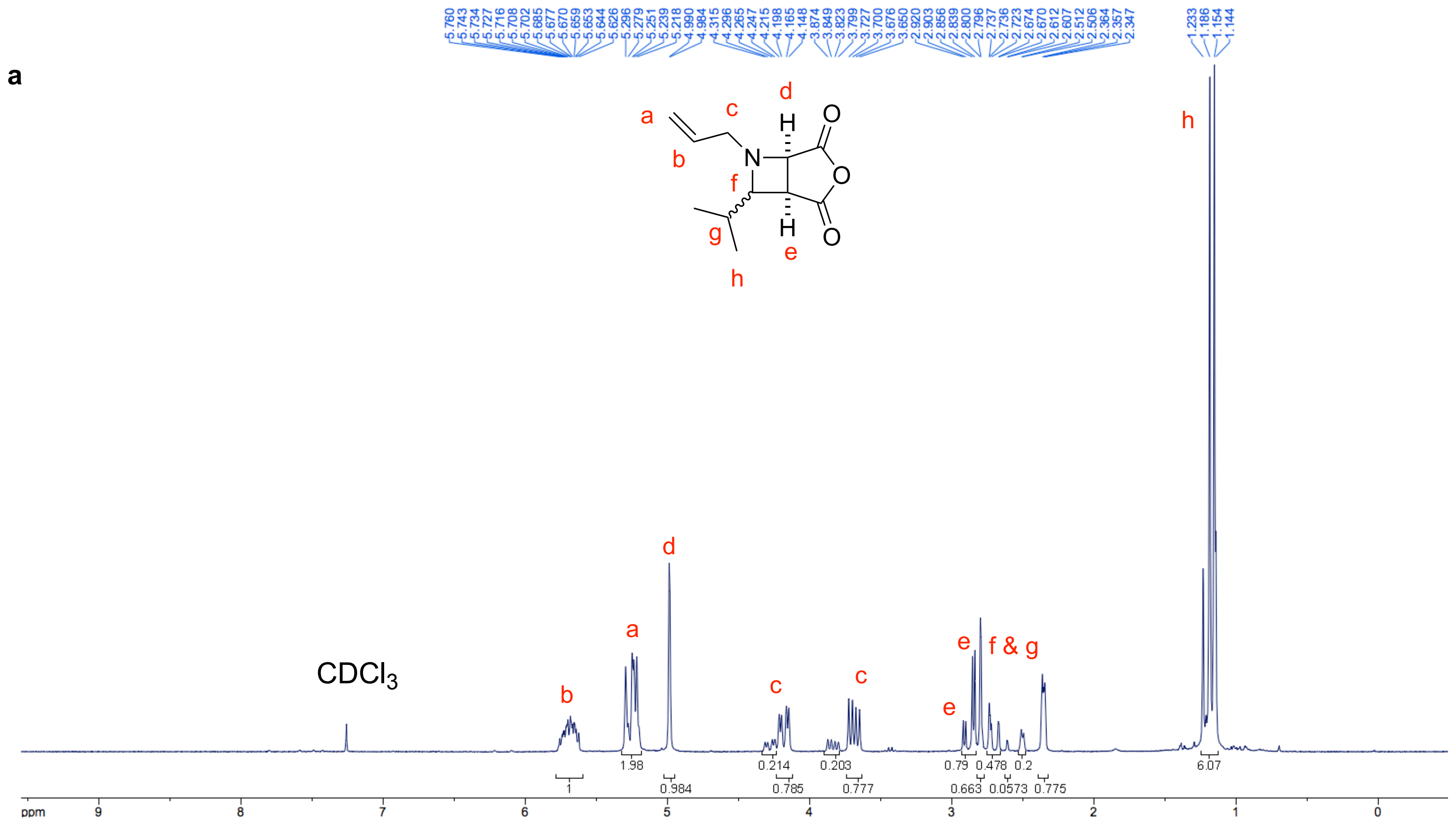

b

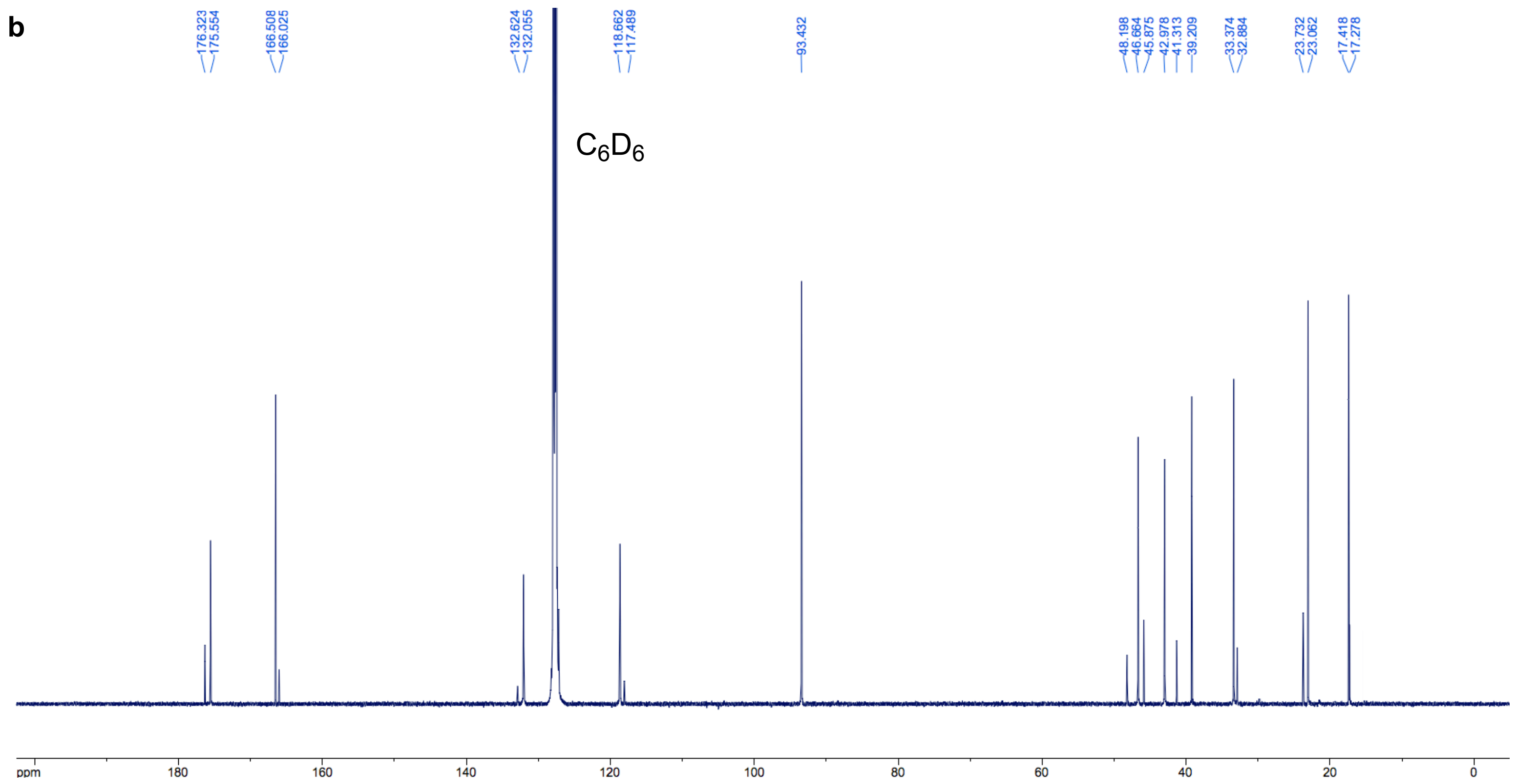

Supplementary Figure 71. NMR spectra of **27**. **a** ^{1}H NMR (300 MHz, C_6D_6). **b** ^{13}C NMR (126 MHz, C_6D_6).

a

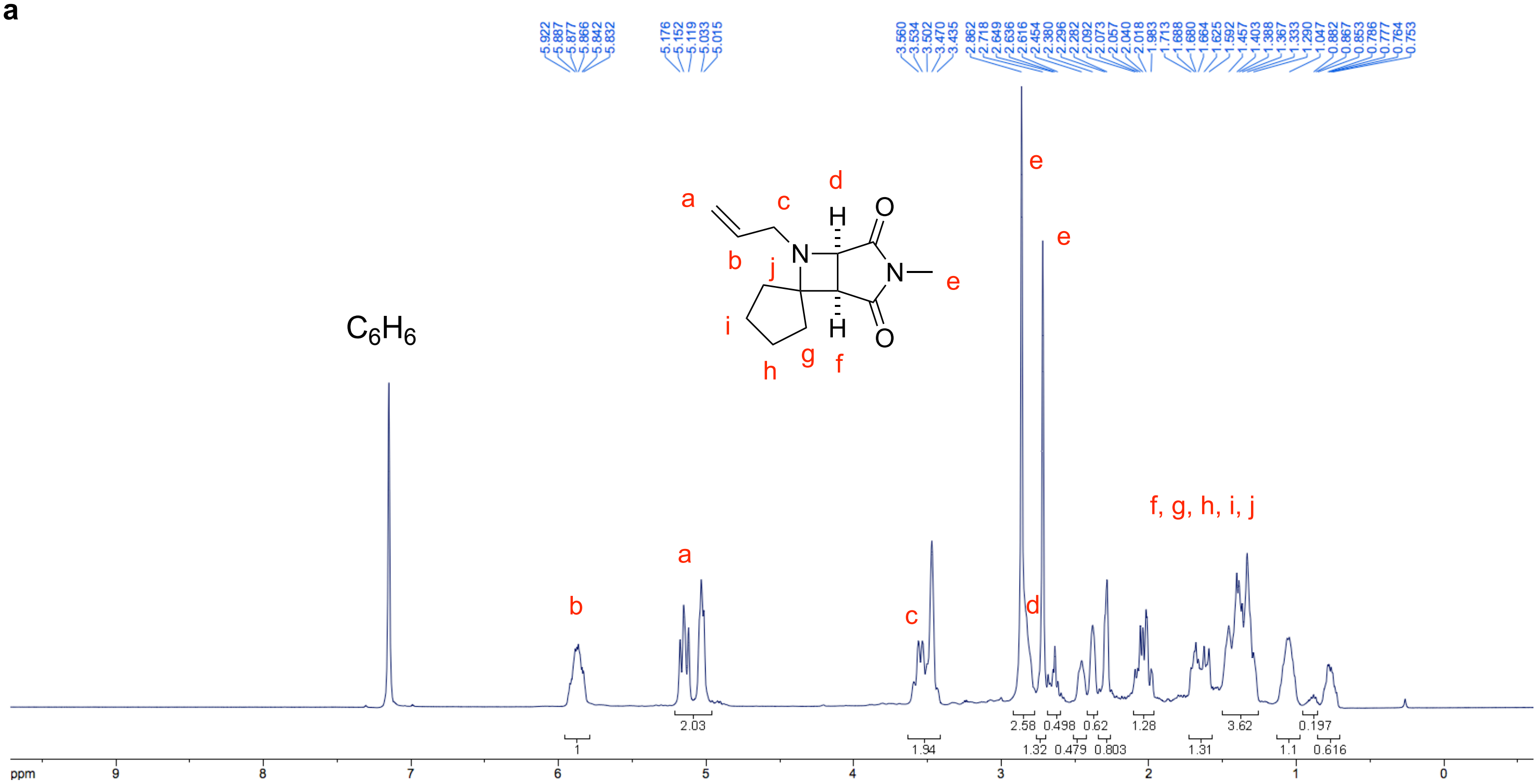

b

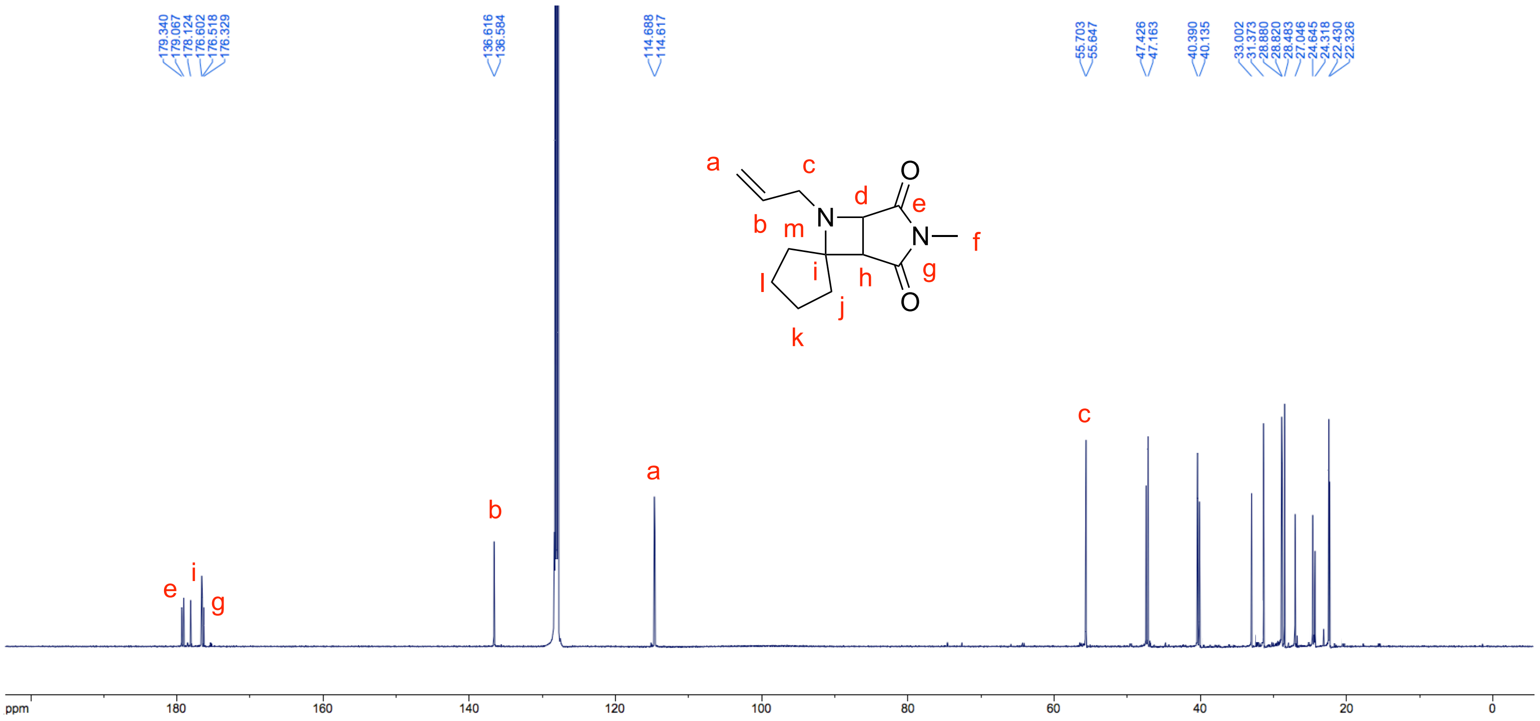

Supplementary Figure 72. NMR spectra of **28**. **a** ^{1}H NMR (300 MHz, C_6D_6). **b** ^{13}C NMR (126 MHz, C_6D_6).

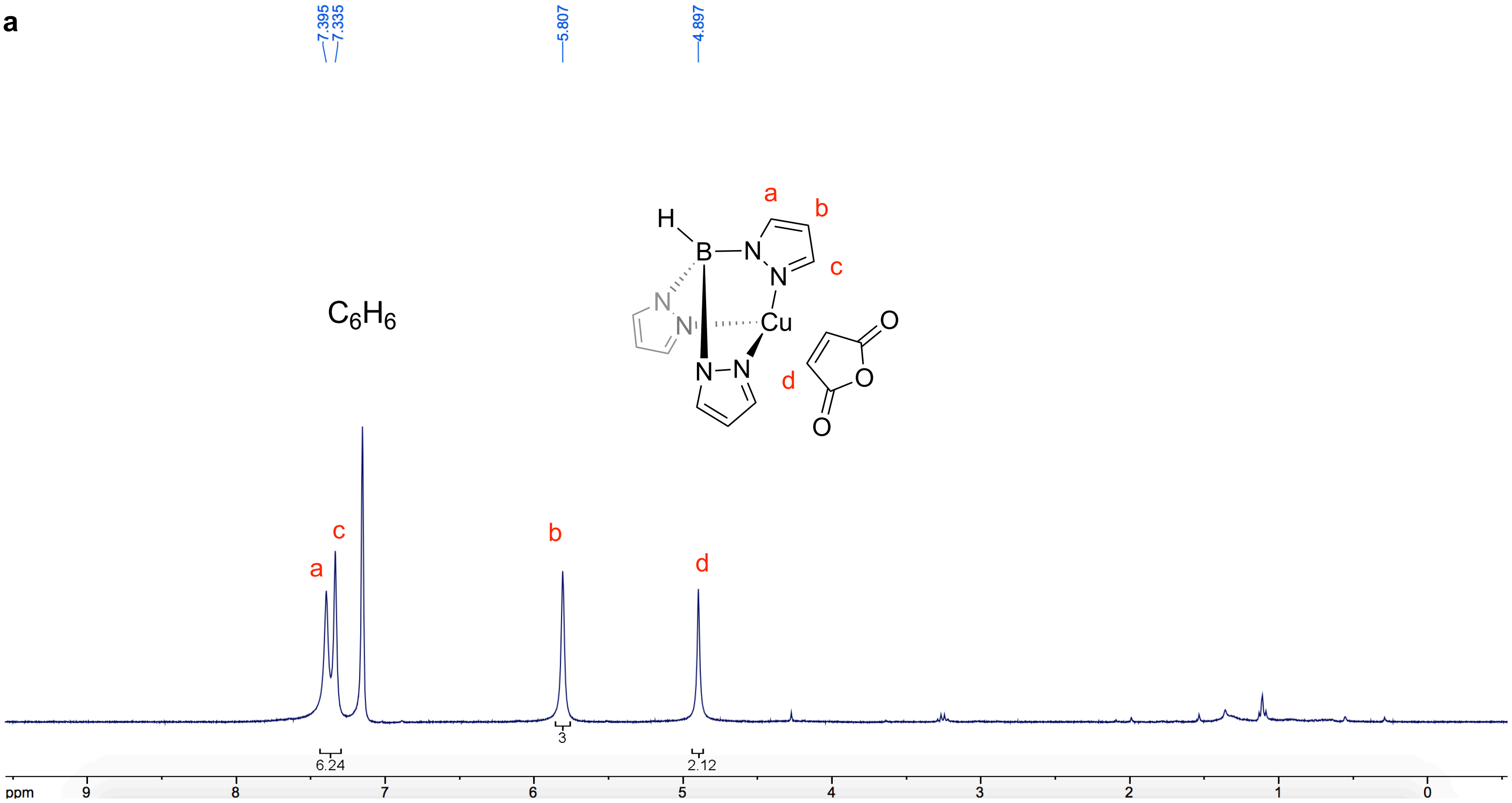

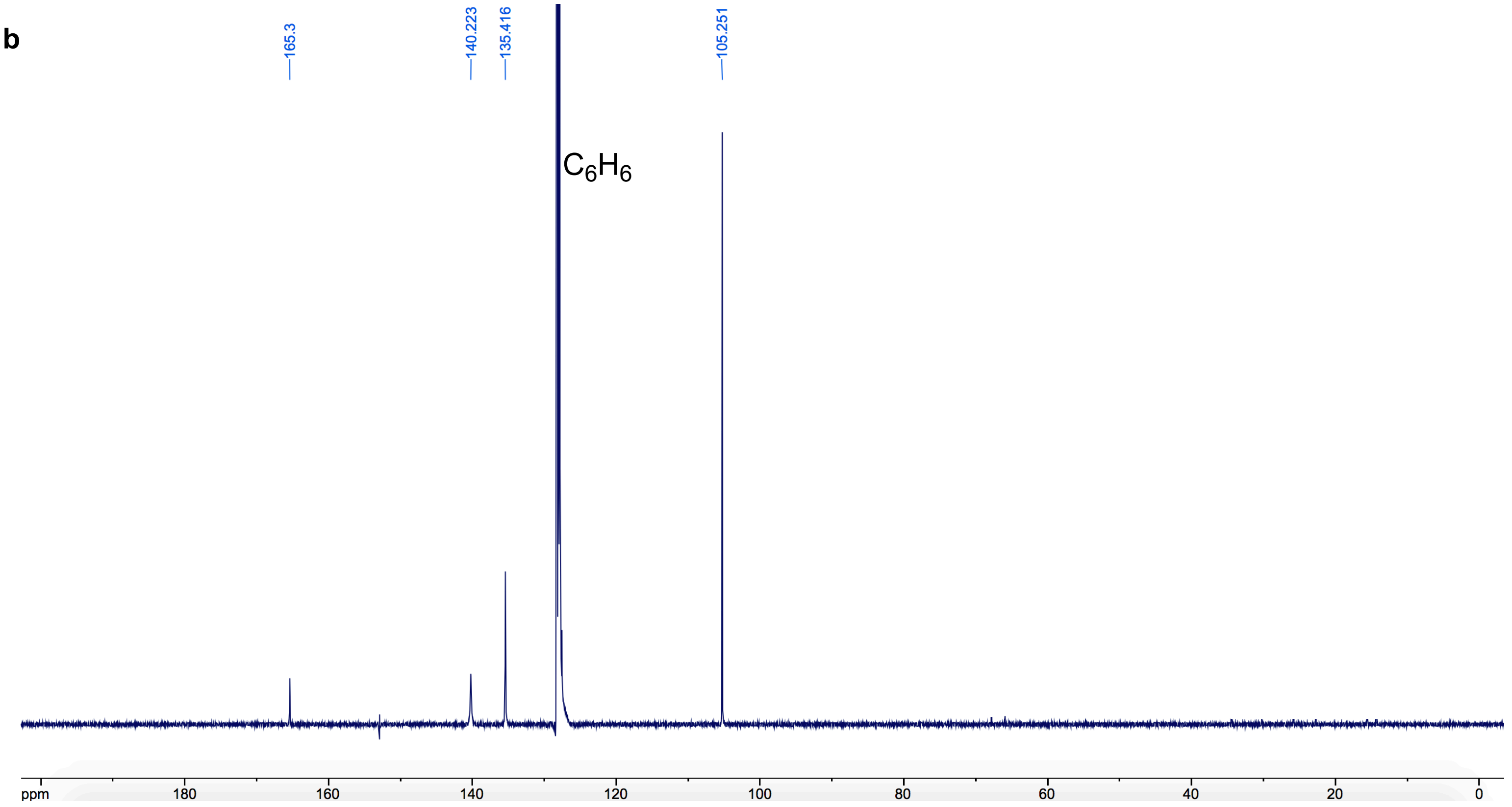

Supplementary Figure 73. NMR spectra of TpCu-MA. **a** ^{1}H NMR (300 MHz, C_6D_6). **b** ^{13}C NMR (126 MHz, C_6D_6).

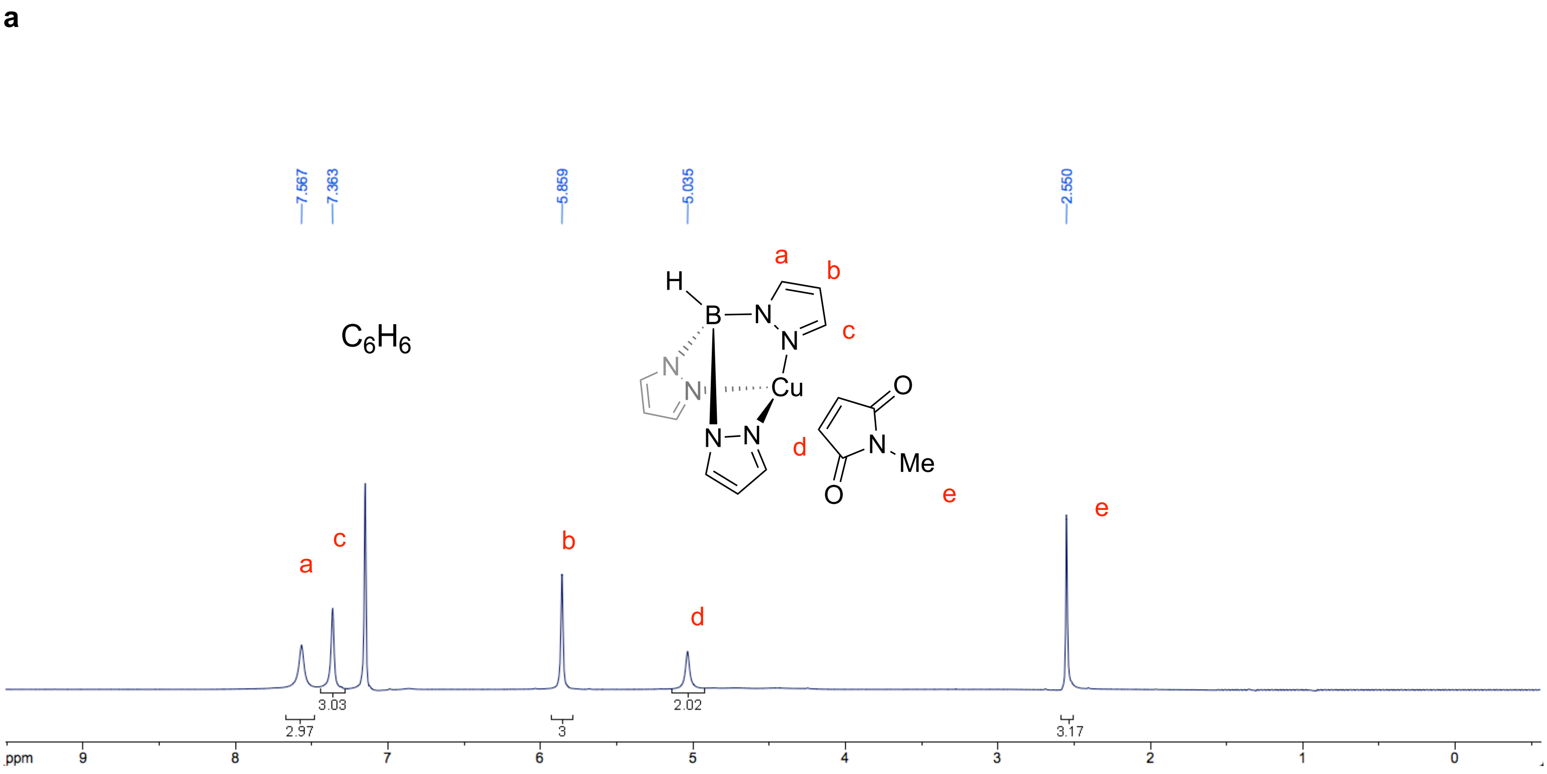

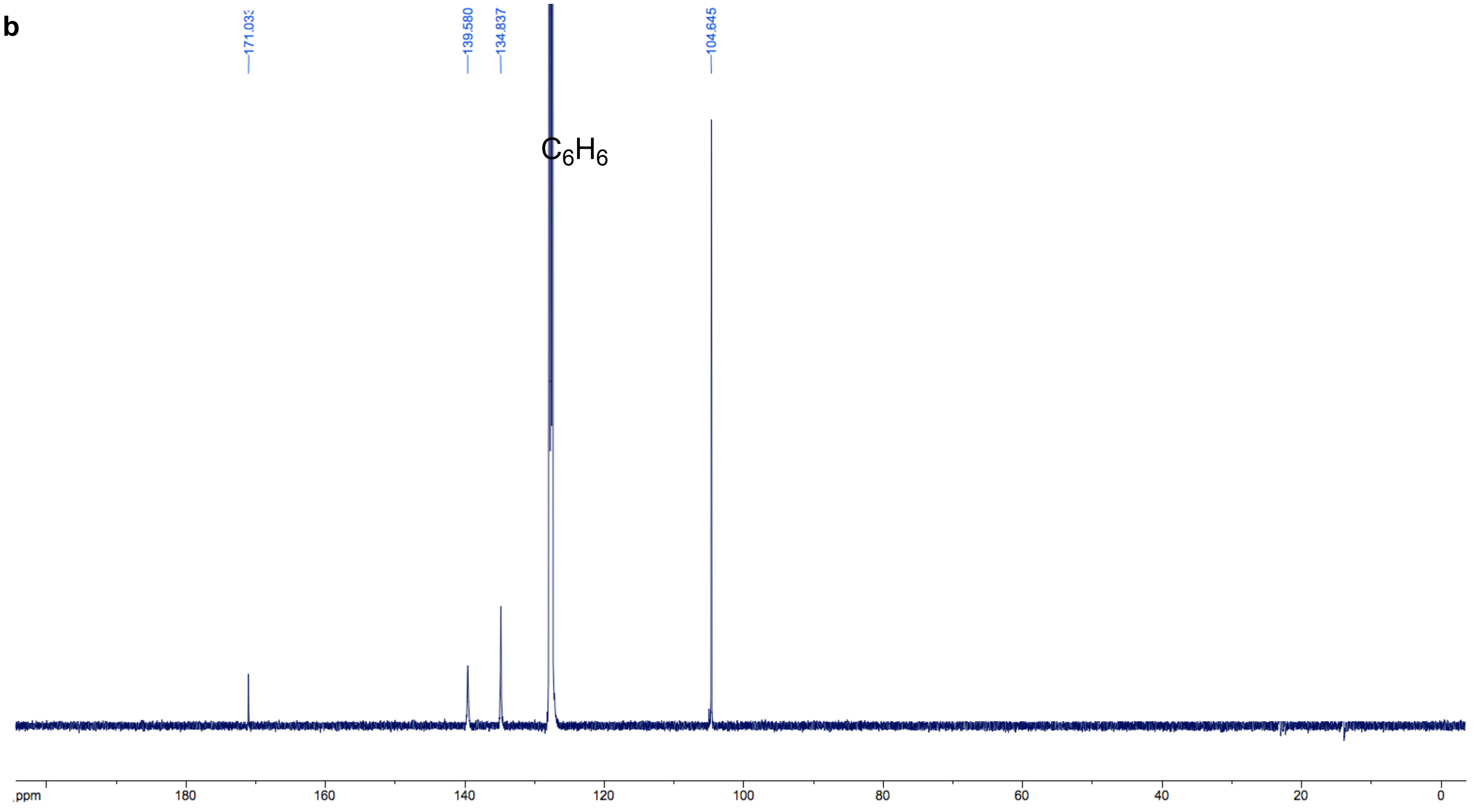

Supplementary Figure 74. NMR spectra of TpCu-MI. **a** ^{1}H NMR (300 MHz, C_6D_6). **b** ^{13}C NMR (126 MHz, C_6D_6).

a

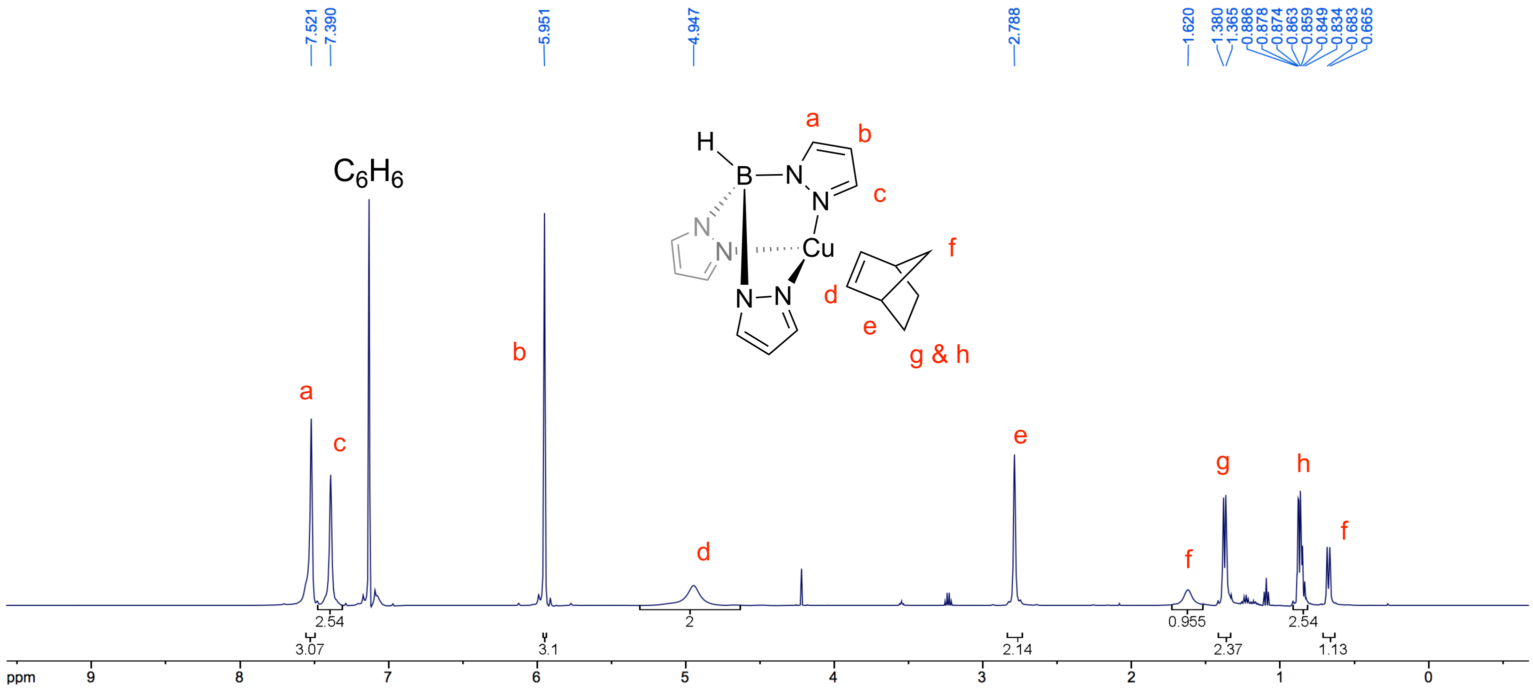

b

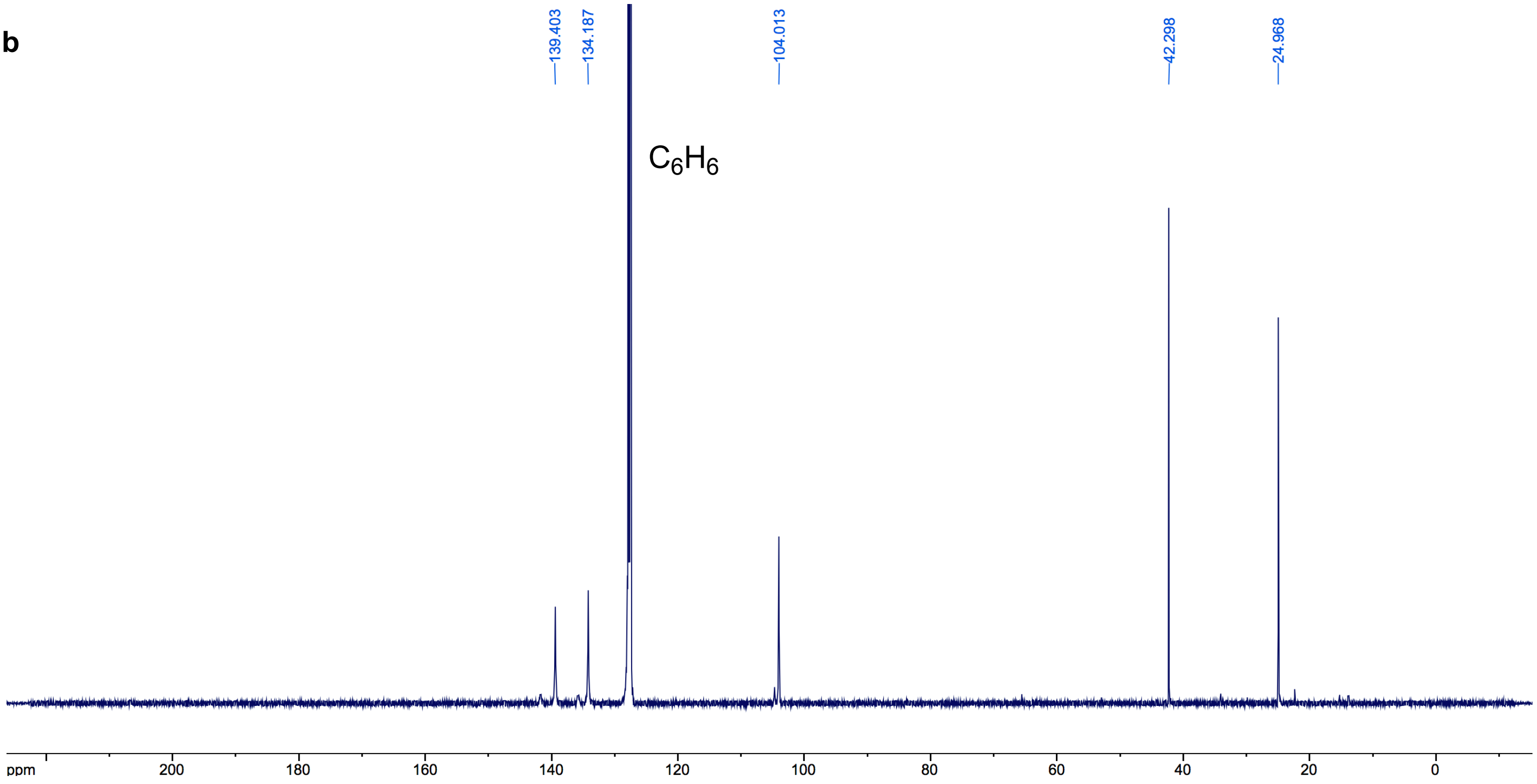

Supplementary Figure 75. NMR spectra of TpCu-NB. **a** ^1H NMR (300 MHz, C_6D_6). **b** ^{13}C NMR (126 MHz, C_6D_6).

II. Supplementary References

[1] Salvi, L., Davis, N. R., Ali, S. Z., Buchwald, S. L. A New Biarylphosphine Ligand for the Pd-Catalyzed Synthesis of Diaryl Ethers under Mild Conditions. *Org. Lett.* 14, 170-173 (2012).

[2] Wright, D. L., Schulte II, J. P., Page, M. A. An Imine Addition/Ring-Closing Metathesis Appproach to the Spirocyclic Core of Halichlorine and Pinnaic Acid. *Org. Lett.* **2**, 1847 (2000).

[3] Diaba, F., Montiel, J. A., Serban, G., Bonjoch, J. Synthesis of Normorphans through an Efficient Intramolecular Carbamoylation of Ketones. *Org. Lett.* **17**, 3860 (2015).

[4] Cook, G. R., Barta, N. S., Stille, J. R. Lewis Acid-promoted 3-aza-Cope rearrangement of N-alklyl-Nallyl enamines. *J. Org. Chem.* **57**, 461 (1992).

[5] Dar'In, D., Bakulina, O., Chizhova, M., Krasavin, M. New Heterocyclic Product Space for the Castagnoli-Cushman Three Component Reaction. *Org. Lett.* **17**, 3930 (2015).

[6] Katritzky, A. R., Hong, Q., Yang, Z. Preparations of Secondary Amines and .beta.-Amino Esters via Additions of Grignard and Reformatsky Reagents to Imines and by One-Pot Reactions of Primary Amines, Aldehydes, and Grignards. *J. Org. Chem.* **60**, 3405 (1995).

[7] Barton, V., Ward, S. A., Hill, J. C. A., O'Neill, P. M. Rationale Design of Biotinylated Antimalarial Endoperoxide Carbon Centered Radical Prodrugs for Applications in Proteomics. *J. Med. Chem.* **53**, 4555 (2010).

[8] Pirrung, M. C., Ghorai, S. Versatile, Fragrant, Convertible Isonitriles. *J. Am. Chem. Soc.* **128**, 11772 (2006).

[9] Capon, B., Wu, Z. P. Comparison of the tautomerization and hydrolysis of some secondary and tertiary enamines. *J. Org. Chem.* **55**, 2317 (1990).

[10] Trost, B. M., Mahapatra, S., Hansen, M. Palladium-Catalyzed C-H Activation of N-Allyl Imines: Regioselective allylic Alkylations to Deliver Substituted Aza-1,3-Dienes. *Angew. Chem. Int. Ed.* **54**, 6036 (2015).

[11] Guizzett, S., Benaglia, M., Celentano, G. 1,1'-Binapthyldiamine-Based Lewis Bases as Readily Available and Efficient Organocatalysts for the Reduction fo N-Aryl and N-Alkyl Ketimines. *Eur. J. Org. Chem.* **22**, 3683 (2009).

[12] Mayo, P, Tam, W. Ring-opening metathesis-cross-metathesis reactions (ROM-CM) of substituted norbornadienes and norbornenes. *Tetrahedron* **58**, 9513 (2002).

[13] Leamire-Audoire, S., Savignac, M., Genet, J. P. Selective deprotection of allyl amines using palladium. *Tetrahedron Lett.* **36**, 1267 (1995).

[14] Shirokane, K., Wada, T., Yoritate, M., Minamikawa, R., Takayama, N., Sato, T., Chida, N. Total Synthesis of Gephyrotoxin by Amide-Selective Reductive Nucleophilic Addition. *Angew. Chem. Int. Ed.* **53**, 512 (2014).

[15] Kubista, M., Sjöback, R., Eriksson, S., Albinsson, B. Experimental correction for the inner-filter effect in fluorescence spectra. *Analyst* **119**, 417-419 (1994).

[16] Zhao, Y., Truhlar, D. G. M06 suite of density functionals for main group thermochemistry, thermochemical kinetics, noncovalent interactions, excited states, and transition elements: two new functionals and systematic testing of four M06-class functionals and 12 other functionals. *Theor. Chem. Acc.* **120**, 215-241 (2008).

[17] Peverati, R., Truhlar, D. G. Improving the Accuracy of Hybrid Meta-GGA Density Functionals by Range Separation. *J. Phys. Chem. Lett.* **2**, 2810-2817 (2011).

[18] Weigend, F., Ahlrichs, R. Balanced basis sets of split valence, triple zeta valence and quadruple zeta valence quality for H to Rn: Design and assessment of accuracy. *Phys. Chem. Chem. Phys.* **7**, 3297-3305 (2005).
